# Atualização da Diretriz Brasileira de Hipercolesterolemia Familiar – 2021

**DOI:** 10.36660/abc.20210788

**Published:** 2021-10-06

**Authors:** Maria Cristina de Oliveira Izar, Viviane Zorzanelli Rocha Giraldez, Adriana Bertolami, Raul Dias dos Santos, Ana Maria Lottenberg, Marcelo Heitor Vieira Assad, José Francisco Kerr Saraiva, Ana Paula M. Chacra, Tania L. R. Martinez, Luciana Ribeiro Bahia, Francisco Antonio Helfenstein Fonseca, Andre Arpad Faludi, Andrei C. Sposito, Antônio Carlos Palandri Chagas, Cinthia Elim Jannes, Cristiane Kovacs Amaral, Daniel Branco de Araújo, Dennys Esper Cintra, Elaine dos Reis Coutinho, Fernando Cesena, Hermes Toros Xavier, Isabela Cardoso Pimentel Mota, Isabela de Carlos Back Giuliano, José Rocha Faria, Juliana Tieko Kato, Marcelo Chiara Bertolami, Marcio Hiroshi Miname, Maria Helane Costa Gurgel Castelo, Maria Sílvia Ferrari Lavrador, Roberta Marcondes Machado, Patrícia Guedes de Souza, Renato Jorge Alves, Valeria Arruda Machado, Wilson Salgado

**Affiliations:** 1 Universidade Federal de São Paulo São PauloSP Brasil Universidade Federal de São Paulo (UNIFESP), São Paulo, SP – Brasil; 2 Faculdade de Medicina da Universidade de São Paulo Instituto do Coração São PauloSP Brasil Instituto do Coração (InCor) da Faculdade de Medicina da Universidade de São Paulo (FMUSP), São Paulo, SP – Brasil; 3 Grupo Fleury São PauloSP Brasil Grupo Fleury, São Paulo, SP – Brasil; 4 Instituto Dante Pazzanese de Cardiologia São PauloSP Brasil Instituto Dante Pazzanese de Cardiologia, São Paulo, SP – Brasil; 5 Universidade de São Paulo São PauloSP Brasil Universidade de São Paulo, São Paulo, SP – Brasil; 6 Faculdade Israelita de Ciências da Saúde Albert Einstein Hospital Israelita Albert Einstein São PauloSP Brasil Hospital Israelita Albert Einstein (HIAE) – Faculdade Israelita de Ciências da Saúde Albert Einstein (FICSAE), São Paulo, SP – Brasil; 7 Faculdade de Medicina da Universidade de São Paulo Laboratório de Lípides São PauloSP Brasil Faculdade de Medicina da Universidade de São Paulo, Laboratório de Lípides (LIM10), São Paulo, São Paulo, SP – Brasil; 8 Instituto Nacional de Cardiologia Rio de JaneiroRJ Brasil Instituto Nacional de Cardiologia, Rio de Janeiro, RJ – Brasil; 9 Sociedade Campineira de Educação e Instrução CampinasSP Brasil Sociedade Campineira de Educação e Instrução, Campinas, SP – Brasil; 10 Hospital Beneficência Portuguesa de São Paulo São PauloSP Brasil Hospital Beneficência Portuguesa de São Paulo, São Paulo, SP – Brasil; 11 Universidade do Estado do Rio de Janeiro Rio de JaneiroRJ Brasil Universidade do Estado do Rio de Janeiro, Rio de Janeiro, RJ – Brasil; 12 Universidade Estadual de Campinas CampinasSP Brasil Universidade Estadual de Campinas (UNICAMP), Campinas, SP – Brasil; 13 Faculdade de Medicina do ABC São PauloSP Brasil Faculdade de Medicina do ABC (FMABC), São Paulo, SP – Brasil; 14 Pontifícia Universidade Católica de Campinas CampinasSP Brasil Pontifícia Universidade Católica de Campinas, Campinas, SP – Brasil; 15 Hospital Israelita Albert Einstein São PauloSP Brasil Hospital Israelita Albert Einstein (HIAE), São Paulo, SP – Brasil; 16 Cardio-Point Serviços Médicos e Assessoria LTDA SantosSP Brasil Cardio-Point Serviços Médicos e Assessoria LTDA, Santos, SP – Brasil; 17 Universidade Federal de Santa Catarina FlorianópolisSC Brasil Universidade Federal de Santa Catarina (UFSC), Florianópolis, SC – Brasil; 18 Pontifícia Universidade Católica do Paraná CuritibaPR Brasil Pontifícia Universidade Católica do Paraná, Curitiba, PR – Brasil; 19 Universidade Federal do Ceará FortalezaCE Brasil Universidade Federal do Ceará (UFC), Fortaleza, CE – Brasil; 20 Hospital do Coração de Messejana FortalezaCE Brasil Hospital do Coração de Messejana, Fortaleza, CE – Brasil; 21 Faculdade Unichristus FortalezaCE Brasil Professora da Faculdade Unichristus, Fortaleza, CE – Brasil; Universidade Federal da Bahia Universitário Professor Edgard Santos SalvadorBA Brasil Hospital Universitário Professor Edgard Santos da Universidade Federal da Bahia (UFBA), Salvador, BA – Brasil; 23 Santa Casa de Misericórdia da São Paulo São PauloSP Brasil Santa Casa de Misericórdia da São Paulo, São Paulo, SP – Brasil

**Table:** 

Atualização da Diretriz Brasileira de Hipercolesterolemia Familiar – 2021	
O relatório abaixo lista as declarações de interesse conforme relatadas à SBC pelos especialistas durante o período de desenvolvimento deste posicionamento, 2020/2021.	
Especialista	Tipo de relacionamento com a indústria
Adriana Bertolami	Nada a ser declarado
Ana Maria Lottenberg	Declaração financeira
	Nada a ser declarado
	Ana Paula M. Chacra	Nada a ser declarado
Andre Arpad Faludi	Nada a ser declarado
Andrei C. Sposito	Outros relacionamentos
	Financiamento de atividades de educação médica continuada, incluindo viagens, hospedagens e inscrições para congressos e cursos, provenientes da indústria farmacêutica, de órteses, próteses, equipamentos e implantes, brasileiras ou estrangeiras:
	- Sanofi: Aula médica.
Antônio Carlos Palandri Chagas	Declaração financeira
	A - Pagamento de qualquer espécie e desde que economicamente apreciáveis, feitos a (i) você, (ii) ao seu cônjuge/ companheiro ou a qualquer outro membro que resida com você, (iii) a qualquer pessoa jurídica em que qualquer destes seja controlador, sócio, acionista ou participante, de forma direta ou indireta, recebimento por palestras, aulas, atuação como proctor de treinamentos, remunerações, honorários pagos por participações em conselhos consultivos, de investigadores, ou outros comitês, etc. Provenientes da indústria farmacêutica, de órteses, próteses, equipamentos e implantes, brasileiras ou estrangeiras:
	- Instituto de Vita: Membro do conselho científico; Novo Nordisk: Diabetes/obesidade; Pfizer/ Upjohn: Hipolipemiante.
Cinthia Elim Jannes	Nada a ser declarado
Cristiane Kovacs Amaral	Nada a ser declarado
Daniel Branco de Araújo	Declaração financeira
	A - Pagamento de qualquer espécie e desde que economicamente apreciáveis, feitos a (i) você, (ii) ao seu cônjuge/ companheiro ou a qualquer outro membro que resida com você, (iii) a qualquer pessoa jurídica em que qualquer destes seja controlador, sócio, acionista ou participante, de forma direta ou indireta, recebimento por palestras, aulas, atuação como proctor de treinamentos, remunerações, honorários pagos por participações em conselhos consultivos, de investigadores, ou outros comitês, etc. Provenientes da indústria farmacêutica, de órteses, próteses, equipamentos e implantes, brasileiras ou estrangeiras:
	- Novo Nordisk/ Merk do Brasil: Diabetes; Novartis: Dislipidemia.
	Outros relacionamentos
	Financiamento de atividades de educação médica continuada, incluindo viagens, hospedagens e inscrições para congressos e cursos, provenientes da indústria farmacêutica, de órteses, próteses, equipamentos e implantes, brasileiras ou estrangeiras:
	- Sanofi: Dislipidemia.
Dennys Esper Cintra	Nada a ser declarado
Elaine dos Reis Coutinho	Declaração financeira
	A - Pagamento de qualquer espécie e desde que economicamente apreciáveis, feitos a (i) você, (ii) ao seu cônjuge/ companheiro ou a qualquer outro membro que resida com você, (iii) a qualquer pessoa jurídica em que qualquer destes seja controlador, sócio, acionista ou participante, de forma direta ou indireta, recebimento por palestras, aulas, atuação como proctor de treinamentos, remunerações, honorários pagos por participações em conselhos consultivos, de investigadores, ou outros comitês, etc. Provenientes da indústria farmacêutica, de órteses, próteses, equipamentos e implantes, brasileiras ou estrangeiras:
	- Novartis: Atividade não promocional.
Fernando Cesena	Declaração financeira
	A - Pagamento de qualquer espécie e desde que economicamente apreciáveis, feitos a (i) você, (ii) ao seu cônjuge/ companheiro ou a qualquer outro membro que resida com você, (iii) a qualquer pessoa jurídica em que qualquer destes seja controlador, sócio, acionista ou participante, de forma direta ou indireta, recebimento por palestras, aulas, atuação como proctor de treinamentos, remunerações, honorários pagos por participações em conselhos consultivos, de investigadores, ou outros comitês, etc. Provenientes da indústria farmacêutica, de órteses, próteses, equipamentos e implantes, brasileiras ou estrangeiras:
	- Libbs/ Novartis: Dislipidemia; Abbott/ Novo Nordisk: Diabetes; Pfizer: Anticoagulação.
	Outros relacionamentos
	Financiamento de atividades de educação médica continuada, incluindo viagens, hospedagens e inscrições para congressos e cursos, provenientes da indústria farmacêutica, de órteses, próteses, equipamentos e implantes, brasileiras ou estrangeiras:
	- Novartis; Novo Nordisk: Diabetes.
Francisco Antonio Helfenstein Fonseca	Declaração financeira
	A - Pagamento de qualquer espécie e desde que economicamente apreciáveis, feitos a (i) você, (ii) ao seu cônjuge/ companheiro ou a qualquer outro membro que resida com você, (iii) a qualquer pessoa jurídica em que qualquer destes seja controlador, sócio, acionista ou participante, de forma direta ou indireta, recebimento por palestras, aulas, atuação como proctor de treinamentos, remunerações, honorários pagos por participações em conselhos consultivos, de investigadores, ou outros comitês, etc. Provenientes da indústria farmacêutica, de órteses, próteses, equipamentos e implantes, brasileiras ou estrangeiras:
	- Novo Nordisk: Semaglutida; AstraZeneca: Rosuvastatina, Metoprolol, Candesartana; Libbs: Rosuvastatina, Ezetimiba; Sanofi.
	Outros relacionamentos
	Financiamento de atividades de educação médica continuada, incluindo viagens, hospedagens e inscrições para congressos e cursos, provenientes da indústria farmacêutica, de órteses, próteses, equipamentos e implantes, brasileiras ou estrangeiras:
	- AstraZeneca/ Novo Nordisk: Congresso virtual.
Hermes Toros Xavier	Declaração financeira
	A - Pagamento de qualquer espécie e desde que economicamente apreciáveis, feitos a (i) você, (ii) ao seu cônjuge/ companheiro ou a qualquer outro membro que resida com você, (iii) a qualquer pessoa jurídica em que qualquer destes seja controlador, sócio, acionista ou participante, de forma direta ou indireta, recebimento por palestras, aulas, atuação como proctor de treinamentos, remunerações, honorários pagos por participações em conselhos consultivos, de investigadores, ou outros comitês, etc. Provenientes da indústria farmacêutica, de órteses, próteses, equipamentos e implantes, brasileiras ou estrangeiras:
	- Torrent do Brasil: Colesterol; Bayer: Aterotrombose.
	Outros relacionamentos
	Financiamento de atividades de educação médica continuada, incluindo viagens, hospedagens e inscrições para congressos e cursos, provenientes da indústria farmacêutica, de órteses, próteses, equipamentos e implantes, brasileiras ou estrangeiras:
	- Torrent do Brasil: Colesterol.
Isabela Cardoso Pimentel Mota	Declaração financeira
	A - Pagamento de qualquer espécie e desde que economicamente apreciáveis, feitos a (i) você, (ii) ao seu cônjuge/ companheiro ou a qualquer outro membro que resida com você, (iii) a qualquer pessoa jurídica em que qualquer destes seja controlador, sócio, acionista ou participante, de forma direta ou indireta, recebimento por palestras, aulas, atuação como proctor de treinamentos, remunerações, honorários pagos por participações em conselhos consultivos, de investigadores, ou outros comitês, etc. Provenientes da indústria farmacêutica, de órteses, próteses, equipamentos e implantes, brasileiras ou estrangeiras:
	- PTC Therapheutics: Doenças raras.
Isabela de Carlos Back Giuliano	Declaração financeira
	B - Financiamento de pesquisas sob sua responsabilidade direta/pessoal (direcionado ao departamento ou instituição) provenientes da indústria farmacêutica, de órteses, próteses, equipamentos e implantes, brasileiras ou estrangeiras:
	- Genzyme do Brasil: Mipomersen.
José Francisco Kerr Saraiva	Nada a ser declarado
José Rocha Faria Neto	Declaração financeira
	A - Pagamento de qualquer espécie e desde que economicamente apreciáveis, feitos a (i) você, (ii) ao seu cônjuge/ companheiro ou a qualquer outro membro que resida com você, (iii) a qualquer pessoa jurídica em que qualquer destes seja controlador, sócio, acionista ou participante, de forma direta ou indireta, recebimento por palestras, aulas, atuação como proctor de treinamentos, remunerações, honorários pagos por participações em conselhos consultivos, de investigadores, ou outros comitês, etc. Provenientes da indústria farmacêutica, de órteses, próteses, equipamentos e implantes, brasileiras ou estrangeiras:
	- AstraZeneca: Diabetes; Boehringer Ingelheim: Diabetes, Fibrilação atrial; Sanofi/ Medley: Dislipidemia; Novartis.
	Outros relacionamentos
	Financiamento de atividades de educação médica continuada, incluindo viagens, hospedagens e inscrições para congressos e cursos, provenientes da indústria farmacêutica, de órteses, próteses, equipamentos e implantes, brasileiras ou estrangeiras:
	- Sanofi/ Medley: Dislipidemia.
Juliana Tieko Kato	Nada a ser declarado
Luciana Ribeiro Bahia	Declaração financeira
	A - Pagamento de qualquer espécie e desde que economicamente apreciáveis, feitos a (i) você, (ii) ao seu cônjuge/ companheiro ou a qualquer outro membro que resida com você, (iii) a qualquer pessoa jurídica em que qualquer destes seja controlador, sócio, acionista ou participante, de forma direta ou indireta, recebimento por palestras, aulas, atuação como proctor de treinamentos, remunerações, honorários pagos por participações em conselhos consultivos, de investigadores, ou outros comitês, etc. Provenientes da indústria farmacêutica, de órteses, próteses, equipamentos e implantes, brasileiras ou estrangeiras:
	- Novo Nordisk: Obesidade; AstraZeneca: Diabetes.
	Outros relacionamentos
	Financiamento de atividades de educação médica continuada, incluindo viagens, hospedagens e inscrições para congressos e cursos, provenientes da indústria farmacêutica, de órteses, próteses, equipamentos e implantes, brasileiras ou estrangeiras:
	- Novo Nordisk: Obesidade.
Marcelo Chiara Bertolami	Declaração financeira
	A - Pagamento de qualquer espécie e desde que economicamente apreciáveis, feitos a (i) você, (ii) ao seu cônjuge/ companheiro ou a qualquer outro membro que resida com você, (iii) a qualquer pessoa jurídica em que qualquer destes seja controlador, sócio, acionista ou participante, de forma direta ou indireta, recebimento por palestras, aulas, atuação como proctor de treinamentos, remunerações, honorários pagos por participações em conselhos consultivos, de investigadores, ou outros comitês, etc. Provenientes da indústria farmacêutica, de órteses, próteses, equipamentos e implantes, brasileiras ou estrangeiras:
	- Abbott: Lipidil; Sanofi: Zinpass e Zinpass eze, Libbs: Plenance eze.
	Outros relacionamentos
	Financiamento de atividades de educação médica continuada, incluindo viagens, hospedagens e inscrições para congressos e cursos, provenientes da indústria farmacêutica, de órteses, próteses, equipamentos e implantes, brasileiras ou estrangeiras:
	- Novo Nordisk: Ozempic; EMS: Hipolipemiantes; Aché: Trezate.
Marcelo Heitor Vieira Assad	Declaração financeira
	A - Pagamento de qualquer espécie e desde que economicamente apreciáveis, feitos a (i) você, (ii) ao seu cônjuge/ companheiro ou a qualquer outro membro que resida com você, (iii) a qualquer pessoa jurídica em que qualquer destes seja controlador, sócio, acionista ou participante, de forma direta ou indireta, recebimento por palestras, aulas, atuação como proctor de treinamentos, remunerações, honorários pagos por participações em conselhos consultivos, de investigadores, ou outros comitês, etc. Provenientes da indústria farmacêutica, de órteses, próteses, equipamentos e implantes, brasileiras ou estrangeiras:
	- AstraZeneca: Prevenção cardiovascular; Boehringerr: Diabetes/anticoagulação; Novo Nordisk: Diabetes.
	Outros relacionamentos
	Financiamento de atividades de educação médica continuada, incluindo viagens, hospedagens e inscrições para congressos e cursos, provenientes da indústria farmacêutica, de órteses, próteses, equipamentos e implantes, brasileiras ou estrangeiras:
	- Boehringer; Novo Nordisk: Diabetes
Marcio Hiroshi Miname	Declaração financeira
	A - Pagamento de qualquer espécie e desde que economicamente apreciáveis, feitos a (i) você, (ii) ao seu cônjuge/ companheiro ou a qualquer outro membro que resida com você, (iii) a qualquer pessoa jurídica em que qualquer destes seja controlador, sócio, acionista ou participante, de forma direta ou indireta, recebimento por palestras, aulas, atuação como proctor de treinamentos, remunerações, honorários pagos por participações em conselhos consultivos, de investigadores, ou outros comitês, etc. Provenientes da indústria farmacêutica, de órteses, próteses, equipamentos e implantes, brasileiras ou estrangeiras:
	- Amgen: Repatha; Novo Nordisk: Ozempic; Libbs.
	B - Financiamento de pesquisas sob sua responsabilidade direta/pessoal (direcionado ao departamento ou instituição) provenientes da indústria farmacêutica, de órteses, próteses, equipamentos e implantes, brasileiras ou estrangeiras:
	- Kowa: Pemafibrato.
	Outros relacionamentos
	Financiamento de atividades de educação médica continuada, incluindo viagens, hospedagens e inscrições para congressos e cursos, provenientes da indústria farmacêutica, de órteses, próteses, equipamentos e implantes, brasileiras ou estrangeiras:
	- Novo Nordisk: Ozempic.
Maria Cristina de Oliveira Izar	Declaração financeira
	A - Pagamento de qualquer espécie e desde que economicamente apreciáveis, feitos a (i) você, (ii) ao seu cônjuge/ companheiro ou a qualquer outro membro que resida com você, (iii) a qualquer pessoa jurídica em que qualquer destes seja controlador, sócio, acionista ou participante, de forma direta ou indireta, recebimento por palestras, aulas, atuação como proctor de treinamentos, remunerações, honorários pagos por participações em conselhos consultivos, de investigadores, ou outros comitês, etc. Provenientes da indústria farmacêutica, de órteses, próteses, equipamentos e implantes, brasileiras ou estrangeiras:
	- Amgen: Evolocumabe; Amryt: Lomitapide; Aché: Rosuvastatina, Ezetimiba; Libbs.
	B - Financiamento de pesquisas sob sua responsabilidade direta/pessoal (direcionado ao departamento ou instituição) provenientes da indústria farmacêutica, de órteses, próteses, equipamentos e implantes, brasileiras ou estrangeiras:
	- Novartis; PTC Bio; Amgen: Dislipidemia.
	C - Financiamento de pesquisa (pessoal), cujas receitas tenham sido provenientes da indústria farmacêutica, de órteses, próteses, equipamentos e implantes, brasileiras ou estrangeiras:
	- PTC Bio; Amgen: Dislipidemia.
Maria Helane Costa Gurgel Castelo	Declaração financeira
	A - Pagamento de qualquer espécie e desde que economicamente apreciáveis, feitos a (i) você, (ii) ao seu cônjuge/ companheiro ou a qualquer outro membro que resida com você, (iii) a qualquer pessoa jurídica em que qualquer destes seja controlador, sócio, acionista ou participante, de forma direta ou indireta, recebimento por palestras, aulas, atuação como proctor de treinamentos, remunerações, honorários pagos por participações em conselhos consultivos, de investigadores, ou outros comitês, etc. Provenientes da indústria farmacêutica, de órteses, próteses, equipamentos e implantes, brasileiras ou estrangeiras:
	- PTC: Palestra e Advisory board sobre SQF; Amgen: Estuco clinico nos últimos 2 anos - Houser;
	B - Financiamento de pesquisas sob sua responsabilidade direta/pessoal (direcionado ao departamento ou instituição) provenientes da indústria farmacêutica, de órteses, próteses, equipamentos e implantes, brasileiras ou estrangeiras:
	- Amgen: Estudo Houser; Novertis: Estudo Orion.
Maria Sílvia Ferrari Lavrador	Nada a ser declarado
Patrícia Guedes de Souza	Nada a ser declarado
Raul Dias dos Santos Filho	Declaração financeira
	A - Pagamento de qualquer espécie e desde que economicamente apreciáveis, feitos a (i) você, (ii) ao seu cônjuge/ companheiro ou a qualquer outro membro que resida com você, (iii) a qualquer pessoa jurídica em que qualquer destes seja controlador, sócio, acionista ou participante, de forma direta ou indireta, recebimento por palestras, aulas, atuação como proctor de treinamentos, remunerações, honorários pagos por participações em conselhos consultivos, de investigadores, ou outros comitês, etc. Provenientes da indústria farmacêutica, de órteses, próteses, equipamentos e implantes, brasileiras ou estrangeiras:
	- Abbott; Amgen: Dislipidemia; AstraZeneca: Diabetes; EMS; GETZ Pharma; Kowa; Merck; MSD; Novo Nordisk; Novartis; PTC; Pfizer; Hypera; Sanofi.
	B - Financiamento de pesquisas sob sua responsabilidade direta/pessoal (direcionado ao departamento ou instituição) provenientes da indústria farmacêutica, de órteses, próteses, equipamentos e implantes, brasileiras ou estrangeiras:
	- Amgen, Sanofi; Esperion: Dislipidemia; Kowa.
Renato Jorge Alves	Declaração financeira
	A - Pagamento de qualquer espécie e desde que economicamente apreciáveis, feitos a (i) você, (ii) ao seu cônjuge/ companheiro ou a qualquer outro membro que resida com você, (iii) a qualquer pessoa jurídica em que qualquer destes seja controlador, sócio, acionista ou participante, de forma direta ou indireta, recebimento por palestras, aulas, atuação como proctor de treinamentos, remunerações, honorários pagos por participações em conselhos consultivos, de investigadores, ou outros comitês, etc. Provenientes da indústria farmacêutica, de órteses, próteses, equipamentos e implantes, brasileiras ou estrangeiras:
	- Amgen: Evolocumabe; PTC: Volanesorsen; Pfizer: Apixaban.
Roberta Marcondes Machado	Declaração financeira
	Nada a ser declarado
	Outros relacionamentos
	Financiamento de atividades de educação médica continuada, incluindo viagens, hospedagens e inscrições para congressos e cursos, provenientes da indústria farmacêutica, de órteses, próteses, equipamentos e implantes, brasileiras ou estrangeiras:
	- PTC: Nutricionista.
	Tania L. R. Martinez	Nada a ser declarado
Valeria Arruda Machado	Nada a ser declarado
Viviane Zorzanelli Rocha Giraldez	Declaração financeira
	A - Pagamento de qualquer espécie e desde que economicamente apreciáveis, feitos a (i) você, (ii) ao seu cônjuge/ companheiro ou a qualquer outro membro que resida com você, (iii) a qualquer pessoa jurídica em que qualquer destes seja controlador, sócio, acionista ou participante, de forma direta ou indireta, recebimento por palestras, aulas, atuação como proctor de treinamentos, remunerações, honorários pagos por participações em conselhos consultivos, de investigadores, ou outros comitês, etc. Provenientes da indústria farmacêutica, de órteses, próteses, equipamentos e implantes, brasileiras ou estrangeiras:
	- Novo Nordisk: Agonistas de receptor de GLP1; AstraZeneca: Dapagliflozina; Amgen: Inibidores de PCSK9.
	Outros relacionamentos
	Financiamento de atividades de educação médica continuada, incluindo viagens, hospedagens e inscrições para congressos e cursos, provenientes da indústria farmacêutica, de órteses, próteses, equipamentos e implantes, brasileiras ou estrangeiras:
	- Novo Nordisk: Agonistas de receptor de GLP1.
Wilson Salgado Filho	Nada a ser declarado

**Definição de graus de recomendação e níveis de evidência**

**Classes (graus) de recomendação:**

Classe I – Condições para as quais há evidências conclusivas, ou, na sua falta, consenso de que o procedimento é seguro e útil/eficaz.

Classe II – Condições para as quais há evidências conflitantes e/ou divergência de opinião sobre segurança e utilidade/eficácia do procedimento.

Classe IIA – Peso ou evidência/opinião a favor do procedimento. A maioria aprova.

Classe IIB – Segurança e utilidade/eficácia menos bem estabelecida, não havendo predomínio de opiniões a favor.

Classe III – Condições para as quais há evidências e/ou consenso de que o procedimento não é útil/eficaz e, em alguns casos, pode ser prejudicial.

**Níveis de evidência:**

Nível A – Dados obtidos a partir de múltiplos estudos randomizados de bom porte, concordantes, e/ou de meta-análise robusta de estudos clínicos randomizados.

Nível B – Dados obtidos a partir de meta-análise menos robusta, de um único estudo randomizado ou de estudos não randomizados (observacionais).

Nível C – Dados obtidos de opiniões consensuais de especialistas.

## Introdução

A hipercolesterolemia familiar (HF) é uma causa genética comum de doença coronariana prematura, especialmente de infarto do miocárdio, devido à exposição ao longo da vida a concentrações elevadas de colesterol da lipoproteína de baixa densidade (LDL-c). Caracteriza-se por ser uma forma grave de dislipidemia de base genética, em que aproximadamente 85% dos homens e 50% das mulheres podem ter um evento coronariano antes de completar os 65 anos de idade, se não tratados adequadamente.

A HF é considerada um problema de saúde pública, devido à sua alta prevalência (em torno de 1:200 a 1:300 indivíduos da população geral) e à sua associação com doença arterial coronariana (DAC) precoce, com redução da expectativa de vida observada em várias famílias. Além disso, cerca de 200.000 pessoas no mundo vão a óbito a cada ano por ataques cardíacos precoces devido à doença, os quais poderiam ser evitados com tratamentos apropriados. Se a HF não for tratada, homens e mulheres com a forma heterozigótica desenvolverão DAC antes dos 55 e 60 anos, respectivamente. Já os homozigotos comumente desenvolvem DAC muito cedo na vida e, se não tratados, podem morrer antes dos 20 anos de idade. No entanto, quando o diagnóstico é feito e o tratamento é instituído, pode-se modificar a história natural da doença aterosclerótica.

O diagnóstico precoce é fundamental, pois torna possível o início antecipado da medicação hipolipemiante e a mudança na história natural da doença, devendo ser guiado por diretrizes e podendo ser facilitado pelo uso de algoritmos. A identificação dos casos de maior gravidade e o cuidado integrado são estratégias para minimizar o impacto da HF na doença cardiovascular. A abordagem diagnóstica, as medidas nutricionais e o emprego de fármacos potentes, como o tratamento com estatinas de alta intensidade, a combinação de medicamentos e o uso de novos agentes hipolipemiantes, podem modificar a história natural da doença nesses indivíduos.

É importante também o reconhecimento da HF como condição genética autossômica dominante, pois o rastreamento em cascata de familiares de indivíduos afetados é imperativo, uma medida custo-efetiva e que propicia o reconhecimento precoce e a instituição de terapêutica que vise retardar ou impedir o aparecimento da doença aterosclerótica. Atenção especial a crianças e adolescentes, gestantes e HF grave são assuntos abordados nesta diretriz.

O Departamento de Aterosclerose e os maiores especialistas do país reuniram-se com o objetivo de transmitir as melhores informações disponíveis para melhoria da prática clínica no Brasil, de forma clara e objetiva, para a prevenção e o tratamento da doença aterosclerótica cardiovascular prematura e para tranquilizar famílias afetadas por essa condição.

Sinceramente,

Profa. Dra. Maria Cristina de Oliveira Izar, MD, PhD

## 1. História Natural da Hipercolesterolemia Familiar

### 1.1. Definição

A HF é uma doença genética do metabolismo das lipoproteínas, cujo modo de herança é autossômico codominante. Caracteriza-se por níveis muito elevados do LDL-c e pela presença de sinais clínicos específicos, como xantomas tendíneos, arco corneal e doença aterosclerótica cardiovascular (DASCV) antes dos 45 anos. ^1^^,^^2^

As primeiras observações sobre a doença foram feitas pelo patologista Harbitz em meados do século XVIII, quando relatou casos de morte súbita em portadores de xantomas. E em 1938, Müller ^3^ descreveu a HF como uma entidade clínica e observou que a associação de hipercolesterolemia, xantomas e manifestações de DAC eram achados comuns em algumas famílias e herdados como um traço dominante. Cerca de 50 anos mais tarde, Brown e Goldstein, ^4^^-^^6^ ao estudarem pacientes e culturas de células, elucidaram a complexa via da síntese endógena do colesterol e identificaram o defeito na internalização da lipoproteína de baixa densidade (LDL, do inglês, *low density lipoprotein* ) ligada ao seu receptor. Em 1983, esse gene foi clonado e mapeado no braço curto do cromossomo 19, ^7^ sendo então denominado gene do receptor da LDL, ou gene *LDLR* , em 1989. ^8^

A HF tinha uma prevalência “histórica” estimada de 1:500 indivíduos afetados na forma heterozigótica e de 1:1.000.000 na forma homozigótica. ^9^^,^^10^ Khachadurian ^10^ foi quem discriminou essas duas manifestações da HF. No entanto, estudos mais recentes sugerem que a prevalência da doença seja maior, 1:200 a 1:300 na HF heterozigótica (HFHe) e 1:160.000 a 1:300.000 na HF homozigótica (HFHo), com base em critérios clínicos e moleculares. ^11^^,^^12^

As concentrações plasmáticas de LDL-c nos indivíduos com HFHe são, em geral, duas a três vezes maiores do que em pessoas sem a doença, e os portadores da afecção apresentam maior probabilidade de desenvolver DASCV prematura na segunda ou terceira décadas de vida. Já aqueles com HFHo têm concentrações de LDL-c cerca de seis a oito vezes maiores e desenvolvem DASCV muito cedo em sua vida, frequentemente morrendo até a idade de 20 anos, se não tratados. ^9^^,^^13^

O fenótipo clínico de HF é geralmente decorrente de defeitos no gene *LDLR* , que codifica o LDLR, ^5^^,^^10^ sede de mais de 2.251 mutações descritas até o momento. ^14^ Mutações pontuais, ou por substituição de uma única base (polimorfismo de nucleotídeo único [SNP, do inglês, *single nucleotide polymorphism* ]), são responsáveis por mais de 84% das mutações, enquanto rearranjos maiores ocorrem em 16% de todas as mutações descritas no gene *LDLR* .

O fenótipo clínico da HF pode também ser secundário a defeitos no gene APOB, que codifica a apolipoproteína B-100 (Apo B-100) ^15^ – quando defeituosa, apresenta menor afinidade pelo LDLR –, ou ainda quando existe catabolismo acelerado do LDLR devido a mutações com ganho de função no gene proproteína convertase subutilisina/kexina tipo 9 ( *PCSK9* ), que codifica a proteína NARC-1, ^16^ a qual participa do catabolismo do LDLR.

Na maioria dos casos, a HF é causada por mutações em genes que codificam proteínas envolvidas na captação e no catabolismo do LDLR. Os genes *LDLR* , apolipoproteína-B ( *APOB* ) e *PCSK9* são considerados genes ligados ao desenvolvimento de HF, resultando na homeostase defeituosa das partículas de LDL e, consequentemente, na elevação das concentrações plasmáticas de LDL-c. Desse modo, frequentemente, pacientes com diagnóstico molecular de HF apresentam variantes patogênicas no gene LDLR, ^17^ enquanto as mutações dos genes *APOB* e *PCSK9* respondem por menor percentual da HF na forma autossômica dominante (ADH). ^18^ A HF autossômica recessiva (ARH), por outro lado, é uma forma rara e ocorre quando os indivíduos herdam mutações patogênicas em ambas as cópias do gene *low-density lipoprotein adaptor protein 1* ( *LDLRAP1* ), que codifica a proteína adaptadora do receptor de LDL. ^19^ No entanto, sabe-se ainda que a HF pode ser decorrente de variantes patogênicas em genes não conhecidos, ou mesmo de vários genes, sendo conhecida como HF poligênica. ^20^

O fenótipo clínico é muito semelhante entre as formas mais comuns de HF; porém, os defeitos do gene *APOB* são mais comuns entre algumas populações europeias (1:300 a 1:700 na Europa Central), enquanto mutações do gene *PCSK9* não têm uma frequência estabelecida (em geral, ~1%). A HF apresenta penetração elevada; ^20^^-^^22^ assim, a maioria dos portadores de mutações causais para a doença apresentam o fenótipo clínico. Pelo seu modo de herança autossômico codominante, a metade dos descendentes em primeiro grau de um indivíduo afetado serão portadores do defeito genético e apresentarão níveis elevados de LDL-c desde o nascimento e ao longo de sua vida, sendo homens e mulheres igualmente afetados. ^9^^,^^22^

Os heterozigotos possuem metade dos receptores de LDL funcionantes, enquanto, nos homozigotos, por defeito no *LDLR* , ambos os receptores têm perda de função ou função nula ^23^ . A importância do diagnóstico genético reside no fato de que os critérios clínicos/laboratoriais muitas vezes não são conhecidos pelos pacientes, dificultando a confirmação diagnóstica.

De acordo com normatização recente, ^23^ a HF inclui múltiplos fenótipos, devido a diferentes etiologias moleculares e fatores genéticos adicionais. Os níveis de LDL-c, o número de mutações e fatores adicionais protetores ou patogênicos determinam o risco de DAC; portanto, indivíduos sob risco pela história familiar, bem como aqueles com fenótipo de HF, devem ser genotipados. Os resultados desse teste podem fornecer três categorias de indivíduos:

1. Genótipo positivo, fenótipo negativo
2. Genótipo positivo, fenótipo positivo
3. Genótipo negativo, fenótipo positivo.

Em alguns casos, outras etiologias moleculares devem ser pesquisadas, ^23^ como mutações no gene *apo (a)* , *LIPA* , que codifica a lipase ácida lisossomal, além da forma poligênica. O risco de DAC é maior em portadores de mutações patogênicas, se comparado àqueles sem mutações para qualquer valor de LDL-c. Acima de 190 mg/dl, o risco de DAC chega a ser mais de 3 vezes maior para um mesmo nível de LDL-c nos portadores de mutações causais, comparado aos não portadores de mutações; isso provavelmente em função da exposição ao longo da vida a níveis muito elevados de LDL-c. ^24^

A HF é considerada um problema de saúde pública devido à elevada prevalência de doença coronariana precoce e à redução da expectativa de vida observada em várias famílias. Aproximadamente 85% dos homens e 50% das mulheres podem ter um evento coronariano antes de completarem os 65 anos de idade, se não tratados adequadamente. Estudos revelam que cerca de 200.000 pessoas no mundo vão a óbito a cada ano por ataques cardíacos precoces devido à HF, os quais poderiam ser evitados com tratamentos apropriados. ^20^

### 1.2. Epidemiologia da Doença Aterosclerótica Cardiovascular

A DASCV e suas complicações no Brasil e no mundo são um grave problema de saúde pública. Segundo dados fornecidos pelo Departamento de Informática do Sistema Único de Saúde (DATASUS), as doenças cardiovasculares (DCV) são a principal causa de morte no país, com aproximadamente 27,65% do total de óbitos. ^25^ Analisando-se a mortalidade específica por doenças do aparelho circulatório, as afecções isquêmicas do coração são responsáveis por 32% das mortes. ^25^ Dados publicados em 2016 por Ribeiro et al. ^26^ mostram que o sistema público financiou 940.323 hospitalizações para DCV em 2012. No período de 2008 a 2012, as taxas de internações por insuficiência cardíaca congestiva e hipertensão arterial foram reduzidas, enquanto aquelas motivadas por angioplastia e infarto agudo do miocárdio (IAM) aumentaram. ^26^

No mundo, dentre as dez principais causas de morte, estão as doenças isquêmicas do coração e o acidente vascular encefálico (AVE), que ocupam o primeiro e segundo lugares, respectivamente, e juntos são responsáveis por mais de 15,2 milhões de óbitos. Essas afecções permanecem líderes globais de morte nos últimos 15 anos. ^27^ Em estudo realizado nos Estados Unidos de 1989 a 1998, 51% das mulheres e 41% dos homens com morte súbita cardíaca faleceram antes do primeiro contato médico. As síndromes coronarianas agudas foram responsáveis por 27% dessas mortes. ^28^

A maioria dos óbitos por IAM ocorre nas primeiras horas de manifestação da doença, sendo 40 a 65% na primeira hora e aproximadamente 80% nas primeiras 24 horas. ^29^ Entre os sobreviventes, 19% em média evoluem com insuficiência cardíaca, importante causa de internações e morbidade. ^30^^,^^31^

Embora conhecidos fatores de risco cardiovascular sejam responsáveis pela maioria dos casos de DASCV e suas complicações, ^32^^-^^36^ existem condições clínicas que aumentam o risco e antecipam sua ocorrência, como a HF. ^37^^-^^40^

### 1.3. Aspectos Epidemiológicos da Hipercolesterolemia Familiar no Mundo e no Brasil

A HF tem uma prevalência dita *histórica* de 1:500 na população geral. ^22^ No entanto, atualmente, sabe-se que, com base nos dados do *Copenhagen General Population Study* , a prevalência estimada da doença é de 1:223 por critérios clínicos ^37^ e de 1:217 utilizando teste genético. ^38^ Um relato do governo da Dinamarca concluiu que, com uma prevalência de 1:200 a 250, apenas 11 a 13% dos portadores de HFHe seriam identificados (a falha no diagnóstico é particularmente importante nas crianças).

Na forma homozigótica, estimava-se sua prevalência em 1:1.000.000 de indivíduos; porém, hoje se sabe que ela pode acometer 1 em cada 300.000 pessoas, podendo ser maior (1:160.000) quando há um efeito “fundador”. Isso significa que a HFHo pode ser mais prevalente em algumas populações, como os sul-africanos (1:100.000), libaneses (1:170.000), franco-canadenses (1:270.000) e finlandeses, devido à presença de casamentos consanguíneos. ^13^^,^^14^

Assim, dada a estimativa do estudo dinamarquês, uma prevalência de HFHe de 1:220 se traduz em uma frequência alélica de 1:440, assumindo-se uma frequência de HFHo de 1:193.600 casos. Com base nessas estimativas, são preditos cerca de 28 casos de HFHo na Dinamarca; porém, na verdade, bem poucos são reconhecidos, ^38^^,^^39^ e na maioria dos países, a entidade permanece não diagnosticada (menos de 1% no Brasil). ^14^ Estima-se que no mundo todo existam mais de 34.000.000 indivíduos portadores de HF. ^9^^,^^14^ No entanto, menos de 10% tem diagnóstico conhecido, e menos de 25% recebe tratamento hipolipemiante. ^38^ Assumindo-se a mesma prevalência no Brasil, o país deve ter cerca de 1.033 casos de HFHo.

Não existem dados objetivos sobre a prevalência da HF no Brasil. Com base nos dados clínicos e laboratoriais e na história familiar, obtidos a partir do estudo ELSA-Brasil, da população adulta das instituições participantes, e adotando-se os critérios da *Dutch Lipid Clinic Network* (DLCN), há uma estimativa de casos de HF de 1:263, o que corresponde a uma população afetada de 766.000 indivíduos no Brasil. ^41^ Essa prevalência ainda varia com o gênero (0,38% em mulheres e 0,30% em homens), a raça (0,25% em brancos, 0,47% em etnia mista e 0,67% em negros) e com a idade (0,10% de 35 a 45 anos; 0,42% de 46 a 55 anos; 0,60% de 56 a 65 anos; e 0,26% de 66 a 75 anos). ^41^ Recentemente, dados de meta-análise, demonstraram a prevalência global da HF na população geral de 1:311, sendo 18 vezes maior entre os indivíduos com doença cardiovascular aterosclerótica. ^42^ Outra meta-análise evidenciou maior prevalência da HF naqueles com doença isquêmica do coração, doença cardíaca isquêmica prematura e hipercolesterolemia grave, em 10, 20 e 23 vezes, respectivamente. ^43^

### 1.4. Impacto da Hipercolesterolemia Familiar na Doença Aterosclerótica Cardiovascular

A HF é uma causa genética comum de doença coronariana prematura, especialmente de infarto do miocárdio (IAM) e angina *pectoris* , devido à exposição a concentrações elevadas de LDL-c ao longo da vida. ^44^^,^^45^ Se não for tratada, homens e mulheres com HFHe e colesterol total de 310 a 580 mg/dl desenvolverão DAC antes dos 55 e 60 anos, respectivamente. Os homozigotos com colesterol total entre 460 a 1.160 mg/dl geralmente desenvolvem DAC muito cedo na vida e, se não tratados, podem morrer antes dos 20 anos de idade. No entanto, quando o diagnóstico é feito e o tratamento é instituído, pode-se modificar a história natural da doença aterosclerótica. ^46^

Embora não existam dados a respeito do risco de DASCV ou da taxa de tratamento hipolipemiante na HF, em uma grande amostra da população geral de Copenhage, na Dinamarca, a prevalência de DAC entre aqueles com diagnóstico provável ou certeza de HF (segundo a DLCN) foi de 33%, ^37^ dos quais apenas 48% recebiam estatinas. O risco de DAC aumentou em 13 vezes (IC 95%: 10 a 17 vezes) em indivíduos com HF provável ou com certeza que não recebiam estatinas. Dados semelhantes foram observados em outras coortes com HF. ^47^

Por outro lado, o aumento do risco de DASCV em portadores de HF recebendo estatinas é 10 vezes maior (IC 95%: 8 a 14 vezes), o que sugere que as doses de estatinas resultaram em tratamento hipolipemiante insuficiente, ou foram introduzidas tarde na vida, quando a aterosclerose já se desenvolvia de maneira grave. Outros estudos sugerem os mesmos dados sobre o tratamento. ^48^^-^^50^

Na HF, o risco de DASCV prematura é muito elevado, e 5 a 10% dos eventos coronarianos ocorrem antes dos 50 anos. ^47^^,^^51^ Sem tratamento, portadores de HF jovens apresentam um risco de morte 90 vezes maior. ^47^^,^^51^ A doença é ainda responsável por número significativo de internações hospitalares e perda de produtividade, em função da alta incidência de DASCV. ^47^

Por isso, o diagnóstico precoce é fundamental, pois torna possível o início antecipado da medicação hipolipemiante e a mudança na história natural da doença, devendo ser guiado por diretrizes ^52^^-^^54^ e podendo ser facilitado pelo uso de algoritmos. ^55^ Além disso, a identificação dos casos de maior gravidade ^56^^,^^57^ e o cuidado integrado à HF ^58^ são estratégias para minimizar o impacto da HF na doença cardiovascular.

## 2. Metabolismo Lipídico na Hipercolesterolemia Familiar

A quantidade de colesterol circulante depende, por um lado, do balanço, principalmente, entre sua síntese hepática e sua absorção intestinal, e por outro, de sua excreção, especialmente pelas vias biliares. Quando ocorre desequilíbrio nesse processo, como ocorre na HF, o colesterol pode elevar-se significativamente e formar depósitos como xantomas e aterosclerose mais precoce. ^22^ A entrada e a saída do colesterol corpóreo são reguladas por sistema de retroalimentação, em que o aumento da sua absorção na dieta determina diminuição da síntese hepática. Ao contrário das gorduras alimentares, que são absorvidas pelo intestino quase completamente, o colesterol é absorvido de modo parcial, e quando sua quantidade na dieta aumenta, a absorção diminui proporcionalmente. No homem, a LDL transporta a maior parte do colesterol. As LDL são produto de metabolismo das lipoproteínas de densidade muito baixa (VLDL, do inglês, *very low density lipoprotein* ), ricas em triglicerídeos, mas que, sobretudo como remanescentes (lipoproteína de densidade intermediária [IDL, do inglês, *intermediate-density lipoprotein* ]), fornecem também colesterol para formação das placas. Além disso, ao serem deslipidadas em seu conteúdo de triglicerídeos, originam LDL menores e mais densas, consideradas muito aterogênicas. As LDL são removidas da circulação para o interior das células por receptores da membrana celular que reconhecem a Apo B-100, única proteína existente na LDL. Remanescentes e IDL são removidos também por esses receptores, mas de maneira bem mais rápida que a LDL. Isso acontece porque essas partículas, além da Apo B-100, têm Apo E na superfície, a qual apresenta afinidade bem maior pelos receptores do que a Apo B-100.

Na HF também ocorrem defeitos genéticos que afetam o receptor da LDL e que resultam em diminuição da endocitose da lipoproteína. ^59^ A existência da endocitose da LDL mediada por receptor e os defeitos que resultam em deficiência da função dos receptores e em hipercolesterolemia foram descritos por Brown e Goldstein na década de 1970. As várias centenas de polimorfismos no gene do receptor podem afetar tanto a estrutura do receptor que liga a Apo B-100 da LDL quanto outros domínios da proteína e até mesmo a recirculação dos receptores que normalmente são reciclados após a endocitose, voltando à membrana celular. Entretanto, apenas parte desses polimorfismos do receptor LDL se associam ao fenótipo da HF. Defeitos da Apo B e aqueles relacionados com ganho de função da PCSK9 que participa do catabolismo do receptor da LDL constituem aproximadamente 5%, e menos de 1% do fenótipo de HF. ^2^

Outra possibilidade ainda muito mais rara é o defeito em homozigose da proteína adaptadora do receptor de LDL, uma vez que esse polimorfismo é recessivo. Entretanto, estima-se entre 5 e 30% os pacientes com fenótipo de HF em que não se encontra o gene causal, sugerindo uma origem a partir de genes não identificados ou pela combinação (poligênica). Assim, a HF resulta da incapacidade de remoção eficiente do colesterol das LDL, determinando sua elevação plasmática e depósitos nos vasos e tecidos. ^59^

A HF resulta geralmente da transmissão de gene de um dos pais, como herança monogênica autossômica dominante, determinando mais frequentemente sua forma heterozigótica, estimada em 1:200 a 1:250 na Europa e ao redor de 1:250 no Brasil. Entretanto, não é infrequente a concomitância de aumento da lipoproteína (a) (Lp[a]) ou ainda a concomitância de defeito no metabolismo de triglicerídeos, determinando gravidade ainda maior da dislipidemia.

A ocorrência de xantomas na infância ou adolescência, junto com níveis muito elevados de LDL-c (> 500 mg/dl), doença aterosclerótica prematura e estenose valvar aórtica, sugere a forma homozigótica da HF, de muito maior gravidade e dificuldade no tratamento. ^13^ Nessa situação, a maioria dos indivíduos tem os pais com HFHe, geralmente por mutações no gene *LDLR* , mas que também podem ocorrer nos outros genes ( *APOB* ou *PCSK9* ). Pode haver ainda a combinação de polimorfismos de diferentes genes ( *LDLR* , *APOB* , *PCSK9* ou *LDLRAP-1* ). Na forma homozigótica também é frequente a concomitância de baixos níveis de colesterol da lipoproteína de alta densidade (HDL-c), possivelmente por remoção acelerada da Apo A-I ou defeito no efluxo de colesterol. Manifestações homozigóticas também devem ser suspeitadas por elevações menos marcantes de LDL-c (> 300 mg/dl) na ocorrência de xantomas antes dos 10 anos. ^13^

## 3. Diagnóstico Clínico da Hipercolesterolemia Familiar

Os critérios clínicos e laboratoriais para o diagnóstico da HF são arbitrários e baseiam-se nos seguintes dados:

- Sinais clínicos de depósitos extravasculares de colesterol
- Taxas elevadas de LDL-c ou colesterol total no plasma
- História familiar de hipercolesterolemia e/ou doença aterosclerótica prematura
- Identificação de mutações e polimorfismos genéticos que favoreçam o desenvolvimento da HF.

Alguns critérios têm sido propostos na tentativa de uniformizar e formalizar o diagnóstico de HF, como os do *US Make Early Diagnosis Prevent Early Death Program* ( *USA MEDPED* ), ^60^ os da *DLCN* (Dutch MEDPED, ver Tabela 1 ), ^61^ e os do *Simon Broome Register Group.*^62^ No Brasil é utilizado o *Dutch MEDPED* .

**Tabela 1:** Critérios diagnósticos de HF heterozigótica com base nos critérios da *Dutch Lipid Clinic Network* (Dutch MEDPED) ^61^

Parâmetro	Pontos
**História familiar**	
Parente de primeiro grau portador de doença vascular/coronariana prematura (homens com menos de 55, mulheres com menos de 60 anos) OU Parente adulto com colesterol total > 290 mg/dl *	1
Parente de primeiro grau portador de xantoma tendíneo e/ou arco corneano OU Parente de primeiro grau < 16 anos com colesterol > 260 mg/dl *	2
**História clínica**	
Paciente portador de doença coronariana prematura (homens com menos de 55, mulheres com menos de 60 anos)	2
Paciente portador de doença cerebral ou periférica prematura (homens com menos de 55, mulheres com menos de 60 anos)	1
**Exame físico**	
Xantoma tendíneo	6
Arco corneano < 45 anos	4
**Níveis de LDL-c (mg/dl)**	
≥ 330	8
250 a 329	5
190 a 249	3
155 a 189	1
**Análise do DNA**	
Presença de mutação funcional do gene do receptor de LDL, Apo B-100 ou PCSK9 *	8
**Diagnóstico de HF**	
Certeza se	> 8
Provável se	6 a 8
Possível se	3 a 5

* Modificado de Dutch Lipid Clinic Network, adotando um critério do Simon Broome Register Group. ^62^ LDL-c: colesterol da lipoproteína de baixa densidade; DNA: ácido desoxirribonucleico; HF: hipercolesterolemia familiar.

Essa diretriz recomenda a utilização de critérios simples para a suspeita diagnóstica de HF e para a decisão de se iniciar o tratamento (ver adiante). Um algoritmo com base no *Dutch MEDPED* pode ser empregado para melhor precisão diagnóstica, embora não esteja disponível até o momento validação para a população brasileira.

### 3.1. Anamnese

Dada a alta prevalência de HF na população geral e o seu grande impacto nas taxas de doença cardiovascular e mortalidade, toda anamnese deve incluir a pesquisa de histórico familiar de hipercolesterolemia, de uso de medicamentos hipolipemiantes e de doença aterosclerótica prematura, incluindo a idade de acometimento. A possibilidade de HF é sempre reforçada com história familiar de hipercolesterolemia e/ou doença aterosclerótica prematura.

### 3.2. Exame Físico

A pesquisa pelos sinais clínicos da HF (xantomas, xantelasmas e arco córneo) deve fazer parte do exame físico rotineiro e pode ser complementada por exames subsidiários, como o ultrassom de tendão, em casos selecionados. Tais sinais clínicos não são muito sensíveis, mas podem ser bastante específicos, ou seja, embora não haja necessidade deles para o diagnóstico da HF, quando identificados, sugerem fortemente essa etiologia.

**Figura 1:**
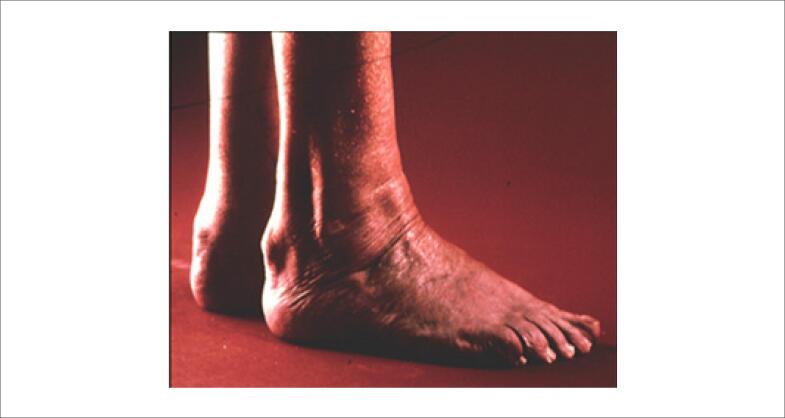
Xantoma tendinoso em tendão calcâneo.

**Figura 2:**
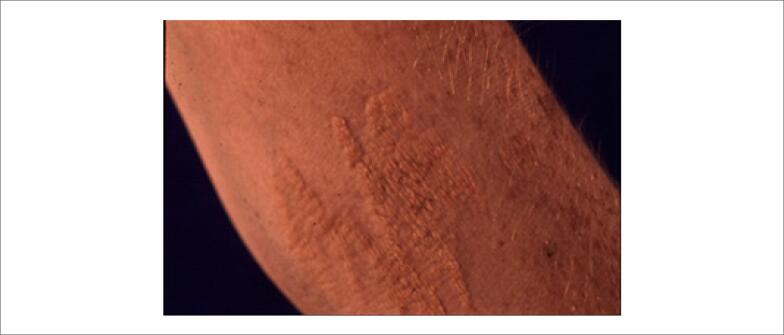
Xantoma plano.

Os xantomas tendinosos ( Figura 1 ) são mais comumente observados no tendão de Aquiles e nos tendões extensores dos dedos, mas também podem ser encontrados nos tendões patelar e do tríceps. Eles devem ser pesquisados não só pela inspeção visual, mas também pela palpação. São praticamente patognomônicos de HF, mas ocorrem em menos de 50% dos casos. ^63^ Podem ocorrer também xantomas planares intertriginosos, especialmente na HF homozigótica ( Figura 2 ).

**Figura 3 (A e B):**
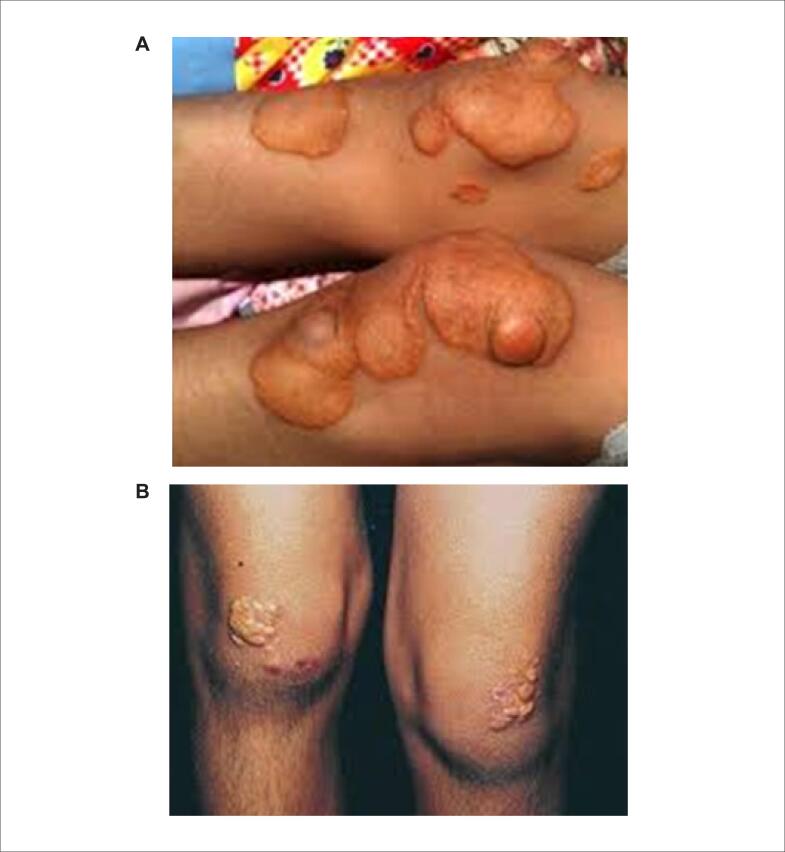
Xantomas tuberosos em joelhos.

Os xantomas tuberosos amarelo-alaranjados (Figuras 3 e 4 ) e os xantelasmas de pálpebras não são específicos de HF e devem ser valorizados quando encontrados em pacientes com idade em torno de 20 a 25 anos. A presença de arco córneo, parcial ou total, sugere HF quando observada antes dos 45 anos de idade ( Figura 5 ). Portadores da forma homozigótica da HF podem apresentar também sopro sistólico ejetivo decorrente de estenose da valva aórtica e da região supra-aórtica.

**Figura 4:**
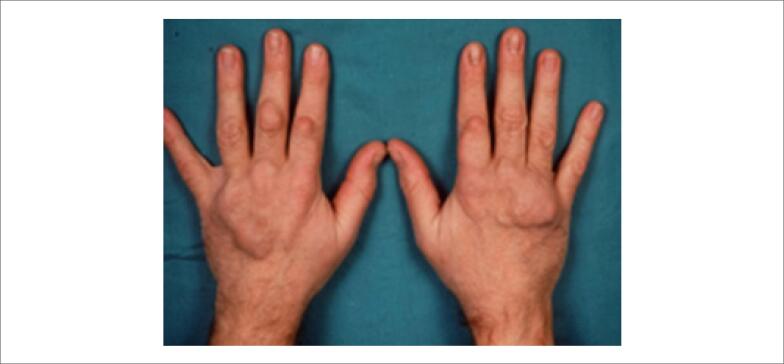
Xantomas tuberosos em mãos.

**Figura 5:**
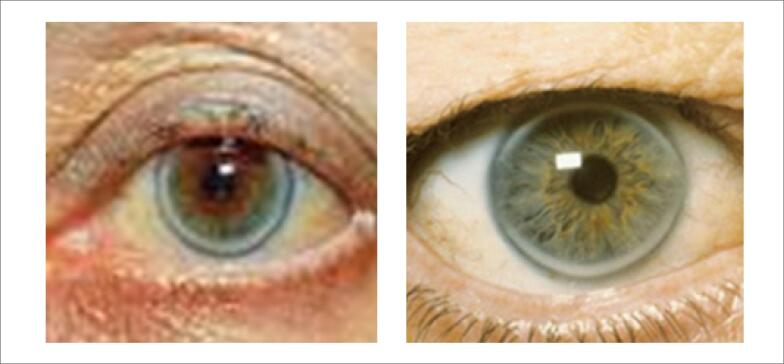
Arco córneo.

### 3.3. Rastreamento e Níveis Lipídicos

A coleta de sangue para determinação das taxas de colesterol total e LDL-c visando rastrear a HF é de fundamental importância para o diagnóstico do maior número possível de casos e, consequentemente, para reduzir o impacto da doença sobre a morbimortalidade cardiovascular na população geral. Esse rastreamento pode ser realizado por meio de dois métodos: o chamado rastreamento universal e o rastreamento em cascata. ^23^^,^^52^

#### 3.3.1. Rastreamento Universal

Todas as pessoas acima dos 10 anos de idade devem ser submetidas à análise do perfil lipídico. ^52^ A obtenção dos lípides plasmáticos também deve ser considerada a partir dos 2 anos de idade nas seguintes situações: ^52^

Quando houver história familiar de doença aterosclerótica prematura (homens com menos de 55 anos ou mulheres com menos de 65 anos) e/ou dislipidemia.

Se a própria criança apresentar xantomas ou arco córneo, fatores de risco (hipertensão arterial, diabetes melito, obesidade) ou doença aterosclerótica.

A periodicidade recomendada para a determinação dos lípides plasmáticos é motivo de debate. Em geral, se o perfil lipídico for normal, mas existirem outros critérios de possível HF, como história familiar de doença aterosclerótica precoce ou hipercolesterolemia significativa, o exame poderá ser repetido após um ano. Na ausência desses fatores, o exame pode ser repetido em até cinco anos. Alguns dados, como idade, presença de outros fatores de risco para aterosclerose, grau de controle dos fatores de risco, hábitos de vida e eventual uso de medicamentos que possam interferir no metabolismo lipídico, podem ser considerados para individualizar a periodicidade das dosagens lipídicas.

O diagnóstico positivo de HF deve sempre ser suspeitado em adultos (≥ 20 anos) com valores de LDL-c ≥ 190 mg/dl. Na população geral, a probabilidade de ter a doença é de aproximadamente 80% no caso de LDL-c ≥ 250 mg/dl em indivíduos com 30 anos ou mais, ou LDL-c ≥ 220 mg/dl em pessoas entre 20 e 29 anos, ou LDL-c ≥ 190 mg/dl nos que têm menos de 20 anos. ^61^ O diagnóstico de HF é também mais provável em portadores de LDL-c ≥ 190 mg/dl cujas famílias são caracterizadas por distribuição bimodal do LDL-c, nas quais alguns membros apresentam taxas tipicamente baixas (LDL-c < 130 mg/dl), enquanto outros (os afetados por HF) exibem taxas tipicamente elevadas, ≥ 190 mg/dl. ^62^

Antes do diagnóstico de HF, no entanto, devem ser afastadas causas secundárias de hipercolesterolemia, incluindo hipotireoidismo e síndrome nefrótica. Deve-se ressaltar também que a presença de hipertrigliceridemia não exclui o diagnóstico de HF.

No Brasil, desde 2017 os laudos laboratoriais destacam valores de colesterol total ≥ 310 mg/dl em adultos e ≥ 230 mg/dl em crianças e adolescentes como sugestivos de HF. ^64^

Por fim, deve-se considerar que a determinação do perfil lipídico está sujeita a uma série de variações relacionadas tanto ao método e aos procedimentos utilizados como a fatores peculiares do indivíduo, como estilo de vida, uso de medicações e doenças associadas. Desse modo, a confirmação de alteração laboratorial com nova amostra, idealmente coletada com intervalo mínimo de uma semana após a primeira coleta, aumenta a precisão diagnóstica.

#### 3.3.2. Rastreamento em Cascata

O rastreamento em cascata envolve a determinação do perfil lipídico em todos os parentes de primeiro grau (pai, mãe e irmãos) dos pacientes diagnosticados com HF. As chances de identificação de outros portadores da doença a partir de um caso-índice são: 50% nos familiares de primeiro grau, 25% nos de segundo grau e 12,5% nos de terceiro grau. ^63^ À medida que novos casos vão sendo identificados, novos parentes vão sendo recomendados para o rastreamento. Essa medida é considerada a que tem a melhor relação custo-eficácia para a identificação de portadores de HF.

##### 3.3.2.1. Rastreamento Genético em Cascata

O rastreamento genético é custo-efetivo e pode ser realizado em todos os pacientes e familiares em primeiro grau das pessoas com diagnóstico de HF. O rastreamento em cascata mais custo-efetivo é o que utiliza informação genética de indivíduos afetados nos quais uma mutação causadora da doença tenha sido identificada. ^63^

##### 3.3.2.2. Cascata Reversa

Trata-se da investigação de familiares de primeiro, segundo e terceiro graus a partir de uma criança como caso-índice e, portanto, de maneira reversa. Muitas vezes, a criança com HF é a primeira a ser diagnosticada pelo pediatra, e seus pais desconhecem se são também portadores dessa condição. Portanto, é uma oportunidade de identificação e de tratamento de pai(s) afetados assintomáticos e que nunca utilizaram medicação. ^23^

##### 3.3.2.3. Diagnóstico Oportunístico

É a situação em que o rastreamento do perfil lipídico se faz no momento da imunização. No Brasil, essa não é uma prática comum, mas seria uma oportunidade de diagnóstico precoce de crianças assintomáticas. ^23^^,^^59^

#### 3.3.3. Hipercolesterolemia Familiar Homozigótica

Historicamente, a prevalência da HFHo é muito rara, estimada em 1:1.000.000 de indivíduos na população ao redor do mundo. Entretanto, atualmente, são registradas prevalências maiores do que as inferidas em uma população geral, que variam de 1:160.000 a 1:300.000. ^13^^,^^52^ Os critérios diagnósticos de HFHo são apresentados no Quadro 1 .

**Quadro 1:** Critérios diagnósticos na hipercolesterolemia familiar homozigótica (HFHo)

1. Confirmação genética de dois alelos mutantes nos genes *LDLR* , *APOB* , *PSCK9* , ou no lócus do gene *LDLRAP1* OU
2. LDL-c sem tratamento > 500 mg/dl ou LDL-c tratada > 300 mg/dl mais algum dos seguintes critérios:
xantomas cutâneos ou tendinosos antes dos 10 anos OU
valores de LDL-c elevados consistentes com HF heterozigótica em ambos os pais *

* Exceto no caso de hipercolesterolemia autossômica recessiva. HF: hipercolesterolemia familiar; LDL-c: colesterol da lipoproteína de baixa densidade. Os valores de LDL-c são apenas indicativos de HF homozigótica, mas devem ser considerados valores menores para o diagnóstico de heterozigotos compostos ou duplos, na presença de outros critérios.

### 3.4. Recomendações

Sinais clínicos de HF e história familiar de doença aterosclerótica precoce e/ou dislipidemia devem ser pesquisados em todos os indivíduos (recomendação classe I, nível de evidência C).

O perfil lipídico deve ser obtido em todos os indivíduos acima dos 10 anos de idade (recomendação classe I, nível de evidência C).

A determinação do perfil lipídico deve ser considerada a partir dos 2 anos de idade na presença de fatores de risco, sinais clínicos de HF ou doença aterosclerótica, bem como no caso de história familiar de doença aterosclerótica prematura e/ou de dislipidemia (recomendação classe I, nível de evidência C).

O perfil lipídico deve ser obtido em todos os parentes de primeiro grau dos indivíduos diagnosticados como portadores de HF (recomendação classe I, nível de evidência C).

## 4. Teste Genético para Hipercolesterolemia Familiar

A HF é uma doença autossômica codominante. É causada, principalmente, por alterações genéticas capazes de provocar perda de função no receptor da LDL, na APOB e, com menos frequência, quando ocorrem alterações que promovem ganho de função na proteína PCSK9, responsável pela degradação do receptor da LDL.

### 4.1. Receptor da LDL, Apo B, PCSK9 e Remoção da LDL Circulante

O receptor da LDL está localizado na superfície das células hepáticas e de outros órgãos, ligando-se à LDL via Apo B. Isso leva à sua captação, realizada por um mecanismo de internalização e endocitose do complexo LDL/Apo B/LDLR. Esse processo é mediado pela proteína adaptadora do LDLR tipo 1 (LDLRAP1) presente nas depressões revestidas com clatrina ( *clathrin-coated pits* ). Após internalização, a partícula de LDL e o LDLR separam-se no endossoma, e o LDLR pode sofrer degradação lisossomal facilitada pela PCSK9 ou ser transferido de volta à superfície da célula, sendo o colesterol liberado na célula para metabolismo ou eliminação. Alternativamente, o LDLR pode ser degradado via ligação da PCSK9 exógena ao LDLR na superfície celular, na qual é internalizada e processada para degradação lisossomal. ^16^ Quando os LDLR apresentam alguma alteração genética que modifique sua estrutura ou função, o nível de remoção de LDL do plasma diminui e, consequentemente, o nível plasmático de LDL-c aumenta em proporção inversa ao número de receptores funcionais presentes. ^65^

### 4.2. Herança Autossômica Dominante

Classicamente a HF é causada por variantes patogênicas nos genes *LDLR* , *APOB* e *PCSK9* . O gene que codifica o receptor de LDL ( *LDLR* ) compreende aproximadamente 45.000 pares de bases de DNA e localiza-se no cromossomo 19, sendo formado por 18 éxons e 17 íntrons. O LDLR é uma proteína composta de 839 aminoácidos, incluindo um peptídeo sinal de 21 aminoácidos com vários domínios funcionais.

A análise das mutações descritas no gene *LDLR* demonstra que não existem regiões principais em sua sequência ( *hot spots* ) para o aparecimento de alterações. ^66^^,^^67^ Apesar disso, mutações no éxon 4, responsável pela ligação à LDL via Apo B, parecem estar correlacionadas a fenótipos mais graves da doença. ^66^^-^^70^ De modo interessante,, mutações “de novo” no gene *LDLR* parecem ser raras. ^71^ A produção é finamente regulada por um mecanismo de retroalimentação sofisticado, que controla a transcrição do gene *LDLR* em resposta a variações no conteúdo intracelular de esteróis e da demanda celular de colesterol. ^72^

Existem cerca de 2.900 alterações genéticas associadas à HF, ^73^ e aproximadamente 85 a 90% ocorrem no gene *LDLR* . A HF é mais comumente atribuível a alterações no gene *LDLR* (incluindo missense, nonsense e inserções e deleções), resultando em LDLR com reduções funcionais (parcial a completa) em sua capacidade de remover a LDL da circulação. Dependendo do impacto da mutação sobre a proteína resultante, o indivíduo pode ser receptor-negativo, que expressa pouco ou nenhum LDLR, ou receptor-defeituoso, que expressa isoformas de LDLR com afinidade reduzida para LDL na superfície dos hepatócitos. ^70^^,^^74^^-^^77^

Em indivíduos heterozigotos, um alelo com alteração patogênica é herdado de um dos pais, e um alelo normal, do outro. Como dois alelos funcionais são necessários para manter o nível plasmático normal de LDL-c, a ausência de um funcional pode causar um aumento no nível de LDL para aproximadamente duas vezes o normal já na infância. ^72^ Os indivíduos homozigotos herdam dois alelos com variantes patogênicas; consequentemente, os LDLR têm funcionalidade muito reduzida, e os pacientes são portadores de uma hipercolesterolemia muito grave (400 a 1.000 mg/dl). ^72^

Existem cinco principais classes de alterações no gene *LDLR*: ^70^^,^^76^

- Classe I (mutações nulas): essas alterações afetam a região promotora ou a região codificante do gene, o que resulta na total ausência de síntese do LDLR ou na síntese de um receptor não funcional.
- Classe II: ocasionadas por defeitos no processamento pós-tradução ou falha no transporte do LDLR do retículo endoplasmático para o complexo de Golgi, resultando em menor expressão na superfície celular.
- Classe III: a LDL não se liga corretamente ao LDLR na superfície da célula, graças a um defeito no domínio de ligação do substrato ou no domínio que apresenta homologia estrutural ao Fator de Crescimento Epidérmico (EGF), presentes no LDLR.
- Classe IV: o LDLR liga-se normalmente à LDL, mas esta não é internalizada eficientemente pelo mecanismo de endocitose via depressões revestidas com clatrina ( *clathrin-coated pits* ).
- Classe V: o LDLR não é reciclado de volta para a superfície celular.

O gene *APOB* tem 42 kb, é formado por 29 éxons e 28 íntrons, e dá origem a duas isoformas de proteínas: uma pequena, denominada Apo B-48, e uma grande, chamada de Apo B-100. A primeira é produzida no intestino, sendo um componente dos quilomícrons e seus remanescentes; a segunda é produzida no fígado e é um componente de várias lipoproteínas, como VLDL, IDL, LDL e lipoproteína(a) [Lp(a)]. A hipercolesterolemia, devido à mutação no gene *APOB* , resulta em um fenótipo clínico de HF semelhante ao causado por mutações em outros genes, sendo referida classicamente como defeito familiar da APOB (FDB, do inglês, *familial defective Apo B* ). ^15^ Entretanto, é importante enfatizar que, atualmente, o FDB é considerado um dos tipos de HF, e sua distinção é feita apenas do ponto de vista acadêmico.

Em contraste com o gene *LDLR* , apenas 353 variantes estão descritas para o gene APOB, ^80^ e a maioria delas encontra-se no éxon 26. ^78^^-^^80^ A mutação mais comum no gene *APOB* é a substituição Arg3500Gln, que causa o rompimento da estrutura da proteína. Essa variante corresponde a 5 a 10% dos casos de HF nas populações do norte da Europa, sendo, porém, rara em outras populações. ^79^^,^^80^

Outra possível condição que leva a um fenótipo da HF é o aumento da atividade de PCSK9, também chamada de HF3, na qual mutações com ganho de função levam a maior degradação do LDLR. ^16^^,^^80^^,^^81^ Essa é a causa menos comum de HF, representando 1 a 3% dos casos de HF clinicamente diagnosticados. ^80^^,^^81^ O gene *PCSK* 9 tem 25 kb, contém 12 éxons e dá origem a uma proteína de 692 aminoácidos.

### 4.3. Hipercolesterolemia Autossômica Recessiva

Além dos genes descritos anteriormente, tem-se considerado como uma das causas do fenótipo da HFHo alterações da LDLRAP1. Diferentemente da HF clássica, esses distúrbios têm herança autossômica recessiva, sendo essa forma denominada hipercolesterolemia autossômica recessiva (ARH, do inglês, *autosomal recessive hypercholesterolemia* ). Nela a expressão reduzida da LDLRAP1 dificulta a associação do LDLR nas depressões revestidas com clatrina da superfície celular, ^82^^,^^83^ consequentemente reduzindo ou impedindo a internalização do complexo LDL/LDLR no hepatócito. O gene *LDLRAP1* tem 25 kb, é composto por nove éxons e dá origem a uma proteína de 308 aminoácidos. Apenas indivíduos com mutações no gene em homozigose ou heterozigose composta são afetados; os heterozigotos simples são considerados apenas portadores, pois geralmente não apresentam hipercolesterolemia. Entretanto, existem casos descritos na literatura de portadores com níveis de LDL-c mais altos que outros membros da família e que não apresentam nenhuma alteração. ^84^

### 4.4. Outros Genes Candidatos

Além dos genes apresentados, outros candidatos a serem causadores de HF são: *APOE, IDOL (MYLIP), HCHOLA4, STAP1* e *LIP* A. ^85^

Formas raras de ARH (também denominadas fenocópias da HF) incluem sitosterolemia ou fitosterolemia, em razão de mutações em dois genes adjacentes e com orientações opostas ( *ABCG5* e *ABCG8* ), que codificam proteínas transportadoras da família ABC ( *ATP-binding cassette* ), denominadas esterolina-1 e esterolina-2. ^86^ Elas estão envolvidas na eliminação de esteróis de plantas, que não podem ser utilizados pelas células humanas, e na deficiência de colesterol 7-alfa hidroxilase (CYP7A1), que é a enzima da primeira etapa na síntese de ácidos biliares, resultando em colesterol intra-hepático e aumentado e expressão reduzida de LDLR na superfície do hepatócito. A deficiência de CYP7A1 é a menos comum das condições autossômicas recessivas que podem causar graves hipercolesterolemias. ^87^

### 4.5. Variabilidade do Fenótipo na Hipercolesterolemia Familiar

Estudos atuais mostram que a HF engloba um espectro de fenótipos clínicos com base, em parte, na gama de variantes patogênicas. Assim, indivíduos com mais de uma alteração no mesmo gene e em alelos diferentes (heterozigotos compostos em trans, geralmente do *LDLR* ) podem ter um fenótipo similar ao de um homozigoto verdadeiro (mesma variante em dois alelos). ^23^^,^^88^ A Tabela 2 mostra a variabilidade da distribuição dos valores pré-tratamento de LDL-c para vários genótipos de HF. ^23^

**Tabela 2:** Variabilidade no fenótipo da hipercolesterolemia familiar por ordem decrescente de concentrações de LDL-c

Valores de LDL-c	Genótipos possíveis
400 a 1.000 mg/dl	Variantes patogênicas em homozigose
	Homozigoto *LDLR* “nulo”
	Verdadeiro homozigoto *LDLR*
	Heterozigoto composto *LDLR*
130 a 450 mg/dl	Variantes patogênicas em heterozigose
	*LDLR* “nulo”
	*LDLR* defeituoso
	Ganho de função da *PCSK9*
	APOB
	Formas poligênicas (múltiplos SNP que elevam o LDL-c)
	Lipoproteína (a) elevada
130 a 200 mg/dl	Hipercolesterolemia comum

Adaptada de Sturm et al. ^23^ LDL-c: colesterol da lipoproteína de baixa densidade; LDLR: receptor da LDL; PCSK9: proproteína convertase subutilisina/kexina tipo 9; APOB: apolipoproteína B; SNP: polimorfismo de nucleotídeo único.

É importante ressaltar que já foram descritos níveis normais de LDL-c em indivíduos com alterações patogênicas em famílias portadoras de HF, e nem sempre é identificada alteração patogênica em pessoas com fenótipo da doença. Desse modo, a presença de um fenótipo compatível com HF sem identificação de alteração patogênica nos clássicos genes *LDLR* , *APOB* e *PCSK9* pode estar ligada à herança poligênica. Talmud et al. ^89^ descreveram conjuntos de 12 polimorfismos em diferentes genes em indivíduos hipercolesterolêmicos em que não foi encontrada uma alteração causal. ^89^ Segundo os autores, a herança poligênica explicaria até 88% dos casos de hipercolesterolemia em geral e cerca de 20% daqueles com fenótipo de HF na ausência de causas monogênicas clássicas. ^90^

### 4.6. Racional para Realização do Rastreamento em Cascata

**Figura 6:**
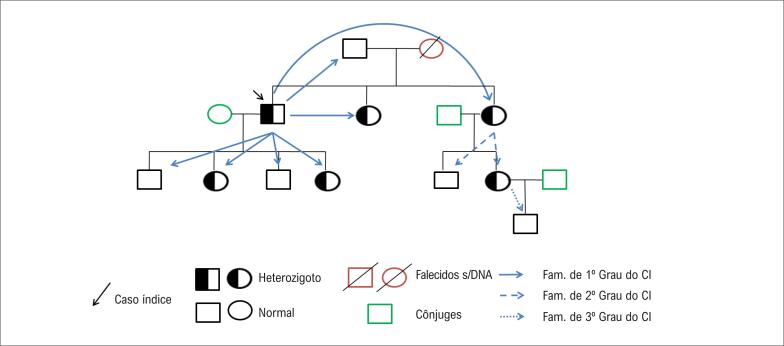
Exemplo de rastreamento genético em cascata. DNA: ácido desoxirribonucleico.

Na HF, o rastreamento genético em cascata vem sendo utilizado como ferramenta para a identificação de novos indivíduos afetados. As alterações patogênicas correlacionadas à doença podem ser identificadas em 30 a 80% dos pacientes, dependendo dos critérios de inclusão e da sensibilidade dos métodos utilizados para o rastreamento. ^91^^,^^92^ A técnica nada mais é do que o sequenciamento das alterações em parentes de primeiro grau de indivíduos identificados com HF. ^93^ Em rodadas de rastreamento, os parentes de primeiro grau identificados com a afecção passam a ser os casos-índices, e seus respectivos parentes começam a ser rastreados ( Figura 6 ).

A cascata genética é a estratégia mais custo-efetiva para a identificação de indivíduos portadores de HF. ^93^^-^^95^ Marks et al. ^93^ analisaram essa custo-efetividade, e foi determinado que o custo incremental por ano de vida adquirido era de £ 3.300 por vida ao ano. O programa foi o mais custo-efetivo na Dinamarca, e o custo por vida ao ano foi de US$ 8.700,00, demonstrando uma estimativa de custos menor que o gasto com prevenção secundária em indivíduos não portadores de HF. ^96^

Estudos mostram que pouquíssimos indivíduos com HF são diagnosticados. Em geral, estima-se que aproximadamente 20% dos pacientes com a doença recebem diagnóstico, e menos de 10% têm tratamento adequado. ^10^ Assim, o rastreamento em cascata aumenta o número de diagnósticos e diminui a idade com que o indivíduo é diagnosticado, oferecendo-lhe maior chance de tratamento precoce e diminuição do risco cardiovascular global.

O teste genético geralmente não é necessário para diagnóstico ou tratamento clínico de um caso-índice, mas pode ser útil quando o diagnóstico é incerto e para a identificação de familiares de um indivíduo afetado. O método de rastreamento em cascata tem sido utilizado por vários países, como Espanha, ^96^ Inglaterra, Holanda, ^97^ Portugal ^98^ e, mais recentemente, no Brasil, ^99^ como ferramenta bastante custo-efetiva na identificação de novos portadores de HF.

Em publicação realizada em um consórcio de estudos genéticos, ^24^ a presença de uma variante patogênica causadora da HF foi encontrada em 2% dos casos graves (LDL-c > 190 mg/dl encontrado em cerca de 7% da população). Indivíduos com a variante monogênica causadora da HF tinham um risco 22 vezes maior de eventos cardiovasculares do que os normolipidêmicos sem alterações genéticas e 4 vezes maior do que os hipercolesterolêmicos sem alterações. ^24^ Essa elevação foi atribuída principalmente à exposição dos portadores de HF a colesterol alto desde o nascimento, diferentemente da hipercolesterolemia poligênica, que pode manifestar-se mais tardiamente. Esses dados sugerem fortemente que a presença de variante genética patogênica causadora da HF tem implicação prognóstica.

Além disso, a identificação de uma alteração causal pode fornecer uma motivação adicional para alguns pacientes iniciarem o tratamento adequado, e o teste genético é padrão-ouro para o diagnóstico de certeza de HF. Pode ser particularmente útil nos casos de familiares com diagnóstico clínico equivocado ou apenas com nível de LDL-c sugestivo da doença. Testes genéticos também podem ser importantes para a identificação de uma alteração causal em famílias recém-identificadas ou com forte suspeita de HF. Ademais, quando encontrada a alteração, o teste fornece uma resposta simples e definitiva para o diagnóstico da HF, tornando-se ferramenta incontestável para a doença como traço familiar. ^23^

No entanto, os testes genéticos têm limitações. Entre os pacientes hipercolesterolêmicos com diagnóstico de possível HF, a taxa de identificação de uma alteração causal por meio do teste genético é de 50% ou menos, enquanto, em pacientes com HF definitiva segundo critérios clínicos, a taxa de identificação da mutação pode ser tão alta quanto 86%. ^23^^,^^100^ Desse modo, é importante ressaltar que um teste genético negativo não exclui a HF. Além disso, indivíduos com LDL-c elevado permanecem em alto risco cardiovascular e devem ser tratados de acordo com diretrizes aceitas, independentemente dos resultados dos testes genéticos.

### 4.7. Metodologias para Diagnóstico Genético

O defeito no gene causal do fenótipo da HF, se *LDLR* , *APOB* , *PCSK9* ou *LDLRAP1* , além dos outros mais raros já citados, não pode ser determinado clinicamente, sendo necessário um teste genético para sua verificação. Assim, por conta da variabilidade de genes e do grande número de mutações possíveis, o método de diagnóstico genético deve incluir o sequenciamento da região codificadora de todos os genes possivelmente ligados à etiologia da doença. ^101^

Para que seja possível esse sequenciamento em grande escala, de modo que um grupo de genes seja sequenciado (painel de genes-alvo), é necessária a utilização da tecnologia de sequenciamento de nova geração (NGS, do inglês, *next-generation sequencing* ). Nessa técnica, é feito um painel contendo todos os genes a serem sequenciados, os quais são colocados em um chip. Outro enfoque mais amplo é a utilização do sequenciamento de exomas, o qual possibilita determinar a sequência da região codificante de praticamente todos os genes presentes no genoma em questão. Contudo, apesar de esse enfoque fornecer uma extensa cobertura do genoma, muitos genes podem não ser sequenciados perfeitamente. Assim, em casos específicos de doenças monogenéticas, como é o caso da HF, painéis contendo os genes-alvo configuram-se como uma alternativa mais custo-efetiva, além de mais precisa.

A tecnologia NGS apresenta muitas vantagens em relação ao sequenciamento Sanger, considerado padrão-ouro nessa técnica. Dentre as vantagens, podem ser citados: a velocidade de obtenção de resultados, a quantidade de material necessário utilizado na reação, o custo do sequenciamento por base, a quantidade de informação gerada e a precisão dos resultados obtidos. Resumidamente, para o estudo genético, é efetuada coleta de sangue periférico em tubo contendo ácido etilenodiamino tetra-acético (EDTA, do inglês, *ethylenediamine tetraacetic acid* ), obtendo-se o DNA genômico de leucócitos. A primeira etapa na preparação do material consiste na geração de uma biblioteca de fragmentos de DNA flanqueados por adaptadores específicos. As regiões de interesse dos genes em estudo são amplificadas por meio da reação em cadeia da polimerase em larga escala, em reações multiplexadas, com centenas de pares de oligonucleotídeos em um mesmo tubo de reação. A partir destas reações, são construídas bibliotecas com códigos de barras para identificar cada paciente analisado. Os fragmentos gerados são amplificados, por clonagem, em esferas por reação em cadeia da polimerase em emulsão, as quais são aplicadas em um chip e inseridas no equipamento de NGS. Uma vez gerados, os dados são transferidos para uma plataforma, na qual as leituras são mapeadas com o genoma humano (hg19/GRCh37) e é realizada a interpretação das variantes.

Cerca de 10% das alterações genéticas no gene do *LDLR* não são pontuais, ^99^ mas sim grandes deleções ou duplicações de éxons do *LDLR* . Portanto, caso não seja identificada nenhuma alteração por NGS, é importante realizar a técnica de amplificação multiplex de sondas dependente de ligação ^102^ (MLPA, do inglês, *multiplex ligation-dependent probe amplification* ) (MCR-Holland) para identificar prováveis deleções e ou duplicações.

O rastreamento em cascata é custo-efetivo e deve ser realizado em todos os pacientes e familiares em primeiro grau de indivíduos com diagnóstico de HF. O mais custo-efetivo é o que utiliza informação genética de pessoas nas quais uma mutação causadora da doença tenha sido identificada. O rastreamento clínico/bioquímico deve ser realizado mesmo quando a realização de teste genético não é possível. ^103^^-^^105^

Um resumo dos benefícios e das limitações para realização do teste genético em cascata pode ser visto no Quadro 2 , adaptado de Sturm et al. ^23^

**Quadro 2:** Benefícios e limitações do teste genético em cascata. ^23^

**Benefícios**
1. Fornece diagnóstico definitivo para a HF.
2. Fornece informações prognósticas e a capacidade de realizar estratificação de risco refinado, porque a detecção de uma variante patogênica indica maior risco cardiovascular.
3. Resultados de testes genéticos positivos mostraram aumentar a iniciação da terapia hipolipemiante, a adesão à terapia e as reduções nos níveis de LDL-c.
4. A detecção precoce oferece a oportunidade para modificações mais precoces no tratamento e no estilo de vida.
5. Quando o teste genético no probando é positivo, leva a testes genéticos em cascata em membros da família em risco com alta sensibilidade e especificidade.
6. Pode excluir HF nos membros da família em risco que não herdam a(s) variante(s) patogênica(s).
7. O teste genético proporciona discriminação a nível molecular entre indivíduos com HFHe, HFHe composta, HF de duplo heterozigoto, HFHo, HF autossômica recessiva e os sujeitos sem uma variante patogênica identificável, mas com o fenótipo de HF. Os riscos de recorrência para parentes e as implicações para o planejamento familiar diferem entre esses cenários.
8. O teste genético possibilita a identificação potencial de “fenocópias” de HF que podem requerer terapias específicas e ter padrões de herança diferentes dos da HF.
9. Pode fornecer motivação adicional para os indivíduos terem maior aderência aos medicamentos prescritos.
10. Fornece uma explicação para o fracasso da dieta e exercício de controle para controlar níveis elevados de lipídios
11. Fornece uma explicação útil para a história familiar de doença cardíaca prematura e níveis de LDL-c difíceis de tratar.
**Limitações**
1. O teste genético para a HF não é completamente sensível ou específico.
2. Nem todos os pacientes com diagnóstico clínico de HF terão variante(s) patogênica(s) identificável(s)
3. Alguns pacientes terão uma variante de significância incerta (VUS, do inglês, *variant of uncertain significance* ) identificada, que pode ser reclassificada como patogênica ou benigna ao longo do tempo, à medida que mais informações são obtidas.
**Custo**
1. Indivíduos podem querer passar por testes genéticos, mas o custo deles pode ser um fator limitante.

Adaptado de Sturm et al. ^23^

HF: hipercolesterolemia familiar; HFHe: HF heterozigótica; HFHo: HF homozigótica; LDL-c: colesterol da lipoproteína de baixa densidade.

### 4.8. Recomendação

1. Triagem laboratorial: Todo indivíduo com suspeita de HF (caso-índice) deverá ter seus familiares de primeiro grau testados para hipercolesterolemia. Caso o resultado seja positivo, deverá ser realizada uma triagem em cascata em outros familiares (de segundo e terceiro graus). Grau de recomendação: I, nível de evidência: A.
2. Triagem genética: O teste genético deverá ser oferecido para o caso-índice; se positivo, deverá ser realizado em seus familiares de primeiro grau. Caso o resultado seja positivo, deverá ser realizada uma triagem em cascata em outros familiares (de segundo e terceiro graus). Grau de recomendação: II, nível de evidência: A.

## 5. Estratificação de Risco Cardiovascular

### 5.1. Epidemiologia do Risco Cardiovascular na Hipercolesterolemia Familiar

A associação entre HFHe e DAC está bem estabelecida. ^51^^,^^105^ isso porque, na ausência de terapia hipolipemiante, existe um risco cumulativo de doença coronariana fatal e não fatal de aproximadamente 50% em homens e 33% em mulheres até 60 anos. ^51^ No estudo do *Simon Broome Register Group* , realizado no período de 1980 até 1995, apesar do tratamento, constatou-se aumento do risco relativo de evento coronariano fatal de 125 vezes entre mulheres com HF e de 20 a 39 anos (mortalidade anual de 0,17%) em relação à população geral da Inglaterra e do País de Gales. Em homens com HF e idade entre 20 e 39 anos, o risco relativo aumentou em 48 vezes (mortalidade anual de 0,46%). ^4^

Estudos mais recentes corroboram o risco aumentado de DAC entre indivíduos com HF (LDL-c ≥ 190 mg/dl), seja de causa monogênica ou poligênica. No estudo de Khera et al., ^24^ observou-se risco aumentado de eventos cardiovasculares entre indivíduos com LDL-c ≥ 190 mg/dl, mesmo na ausência de mutação para HF identificada, em relação àqueles com colesterol normal. ^24^ De 1.386 pessoas com LDL-c ≥ 190 mg/dL (6,7% do total), apenas 24 (1,7%) tinham mutação detectada. Aquelas com LDL-c ≥ 190 mg/dL e sem mutação apresentaram seis vezes mais risco de DAC em relação ao grupo-controle (LDL-c < 130 mg/dL e sem mutação), enquanto as com LDL-c ≥ 190 mg/dL e com mutação demonstraram 22 vezes mais risco. ^24^

Em outro estudo recente, entre pacientes com HF de diagnóstico clínico, a presença de uma causa monogênica da doença se mostrou associada a risco cardiovascular significativamente aumentado (HR ajustado 1,96; IC 95% 1,24 a 3,12; p = 0,004), enquanto o risco cardiovascular em pacientes com hipercolesterolemia poligênica não foi diferente em comparação àqueles sem causa genética identificada. No entanto, a presença de escore poligênico em indivíduos com HF monogênica aumentou ainda mais o seu risco cardiovascular (HR ajustado 3,06; IC 95% 1,56 a 5,99; p = 0,001). ^106^

A despeito do risco aumentado de DAC nos pacientes com HF, o tratamento com estatina associa-se a significativa redução do risco de eventos cardiovasculares. O estudo de Versmissen et al. ^46^ mostrou que indivíduos tratados com a substância apresentaram uma redução de 76% (HR 0,24; IC 95% 0,18 a 0,30; p < 0,001) no risco de eventos coronarianos em relação aos “não tratados” (com atraso no início do tratamento). No entanto, apesar da importante queda nas chances de eventos cardiovasculares com terapia hipolipemiante, pesquisas recentes evidenciam risco residual de eventos nesses pacientes. Em estudo com 821 pacientes com HF (mediana de idade 47,4; 35,3 a 58,3) em uso de terapia hipolipemiante por período de 9,5 anos (5,1 a 14,2), 102 pacientes (12%) apresentaram DCV. Os pacientes mais propensos tinham histórico de evento cardiovascular prévio, história familiar prematura de DCV, hipertensão, maior LDL-c e menor HDL-c sob tratamento, além de serem mais tabagistas em relação a indivíduos sem evento cardiovascular. ^107^

O registro CASCADE FH avaliou desfechos cardiovasculares de pacientes com HF nos EUA. Em uma coorte de 1.900 indivíduos com idade média de 56 ±15 anos, seguimento médio de 20 ±11 anos e prevalência de DCV aterosclerótica prévia de 37%, apenas 48% alcançaram LDL-c < 100 mg/dL e 22% alcançaram LDL-c < 70 mg/dL, apesar do uso de terapia hipolipemiante em 92,8%. Um total de 107 eventos ateroscleróticos ocorreram em 69 (3,6%) do total de pacientes durante o seguimento, correspondendo a uma incidência anual de eventos de 2,2/100 pacientes-ano. ^108^ Desse modo, portadores de HF apresentam risco aumentado de eventos ateroscleróticos mesmo sob tratamento hipolipemiante. Esse risco é variável, a depender do controle do LDL-c e da presença de diversos outros fatores, sugerindo a importância da estratificação desses pacientes.

#### 5.1.1. Recomendações para Estratificação de Risco na HF

O risco cardiovascular na HF é maior. No entanto, embora aumentado, ele é variável de acordo com a presença de fatores de risco. Portanto, recomenda-se a estratificação de risco nesses pacientes (recomendação classe I, nível de evidência B).

### 5.2. Papel dos Fatores de Risco na Hipercolesterolemia Familiar

Os fatores de risco clássicos para DAC na HF são de grande importância na estratificação dessa população. Por exemplo, assim como na população geral, o risco cardiovascular entre pacientes com HF é maior em homens do que em mulheres, como demonstrado em diferentes pesquisas. O estudo de coorte holandês de Jansen et al. ^109^ observou que o risco de um evento cardiovascular foi quase três vezes maior em homens em comparação às mulheres (RR 2,82, IC 95% 2,37 a 3,36). ^109^ Uma meta-análise recente que incluiu 27 estudos e 41.831 participantes quantificou a associação entre vários fatores de risco e DCV em indivíduos com HF. Nela, o risco para homens com DCV foi de cerca de 2 vezes maior (OR: 1,95; CI 95% 1,68 a 2,23). ^110^ Nessa meta-análise e em outros estudos, o tabagismo também apresenta forte associação com o desenvolvimento de DAC em pacientes com HF, com risco em torno de 1,7 a 1,8 vezes maior. ^109^^-^^111^

O diabetes melito é um importante fator de risco cardiovascular na população geral. Em meta-análise do *Emerging Risk Factors Collaboration* , com 102 estudos prospectivos, a doença foi associada a um aumento duas vezes maior de DCV, independentemente de outros fatores de risco. ^112^ Como esperado, o diabetes em indivíduos com HF também está ligado a risco aumentado em comparação a pacientes com HF sem a doença. A meta-análise de Akioyamen et al. ^110^ encontrou uma relação duas vezes maior entre diabetes e DCV nesses pacientes (OR: 1,95; IC 95% 1,33 a 2,57), assim como a hipertensão arterial (OR: 2,11; IC 95% 1,64 a 2,58). O tabagismo, a hipertensão e o diabetes foram responsáveis por mais de um quarto do risco cardiovascular em indivíduos com HF nesse estudo. ^110^

Além de fatores de risco tradicionais, outros também aumentam o risco de eventos em indivíduos com HF, como os antecedentes familiares de DCV, que se mostraram associados a maior risco de DCV em indivíduos com HF. Na meta-análise de Akioyamen et al., ^110^ o risco foi quase duas vezes maior entre indivíduos com história familiar de DCV (OR: 1,83; IC 95% 1,58 a 2,07).

Alguns estudos prévios falharam em demonstrar a associação entre LDL-c e DCV em HF, apesar de o LDL-c elevado ser a principal característica dessa condição. Contudo, existem várias explicações para isso. Por exemplo, a comparação entre indivíduos com LDL-c alto e aqueles com LDL-c similarmente elevado pode não ter eficácia para mostrar efeitos das diferenças de LDL-c, em especial quando a comparação envolve pequeno número de participantes. Além disso, pacientes com LDL-c mais elevado são, em geral, tratados mais agressivamente, introduzindo um fator de confusão nas análises. No entanto, na meta-análise recente de Akioyamen et al., ^110^ análises de metarregressão demonstraram que níveis mais altos de colesterol total e LDL-c não tratados estavam associados a maior risco de DCV, com aumento de 51% para cada aumento de 1 mmol/L de colesterol. Além disso, níveis baixos de HDL-c (< 1 mmol/L) também estiveram relacionados a risco cardiovascular aumentado em indivíduos com HF, ao contrário dos triglicerídeos séricos e das apolipoproteínas A-I e B.

#### 5.2.1. Recomendação sobre o Papel dos Fatores de Risco na HF

Vários fatores apresentam importante papel no risco cardiovascular de pacientes com HF e devem ser pesquisados ativamente nessa população (recomendação classe I, nível de evidência A).

### 5.3. Papel de Outros Fatores no Risco Cardiovascular da Hipercolesterolemia Familiar: Lipoproteína (a), Xantoma de Tendão de Aquiles, Proteína C Reativa

A lipoproteína (a), ou Lp(a), consiste em uma lipoproteína composta por partícula semelhante a LDL, cuja Apo B se encontra covalentemente ligada a uma apolipoproteína (a).

De acordo com evidência acumulada ao longo de vários anos, a Lp(a) elevada é considerada um fator de risco cardiovascular independente na população geral, inclusive com implicação causal. ^112^^-^^114^ Em indivíduos com HF, a condição também consiste em fator de risco adicional, observação de grande relevância nesse grupo, considerando-se o potencial para níveis elevados de Lp(a) nessa população.

#### 5.3.1. Recomendação

A dosagem de Lp(a) deve ser considerada em indivíduos com HF (recomendação classe IIa, nível de evidência B).

O xantoma de tendão de Aquiles é um sinal peculiar da FH e faz parte dos seus critérios diagnósticos. Cerca de 30 a 50% dos indivíduos com HFHe e diagnóstico genético apresentam xantoma tendinoso. Estudo de Civeira et al. ^115^ mostrou que pessoas com HF e portadores de xantomas apresentam maior prevalência de DCV prematura em comparação àquelas sem xantomas (36,7% *versus* 13,8%, p = 0,001). ^115^ Na meta-análise de Oosterveer et al., ^116^ em indivíduos com HF geneticamente confirmada, observou-se um risco três vezes maior de DCV entre os portadores de xantoma tendinoso. ^116^

Um estudo brasileiro mais recente também avaliou a associação de xantomas de tendão de Aquiles com a presença e carga de aterosclerose subclínica em indivíduos com HFHe. Nele, os indivíduos com xantomas (21%) apresentavam concentrações mais elevadas de LDL-c e Lp(a), assim como escore de cálcio mais elevado. Além disso, a associação de xantoma com escore de cálcio persistiu positiva e independente após ajustes para idade, sexo, tabagismo, hipertensão arterial, uso prévio de estatina, HDL-C, LDL-c e Lp(a). ^117^

Apesar da associação positiva entre xantomas e DCV em estudos prévios, na recente meta-análise de Akioyamen et al., ^110^ os dados disponíveis não apontaram os xantomas de tendão como fatores de risco na HF.

Outras recomendações incluem:

1. O xantoma de tendão de Aquiles parece estar relacionado com risco cardiovascular mais alto na HF. Como sua pesquisa se baseia geralmente apenas no exame físico, deve ser estimulada (recomendação classe IIA, nível de evidência B).
2. A associação da proteína C reativa com doença cardiovascular na HF é pautada em estudos pequenos de sua associação com aterosclerose subclínica e com resultados controversos. ^118^^,^^119^
3. Não existe evidência para dosagem rotineira de PCR na HF (recomendação classe IIB, nível de evidência C).

### 5.4. Estratificação de Risco Cardiovascular na Hipercolesterolemia Familar: Uso dos Escores Clínicos para Estratificação de Risco

A estratificação de risco habitual utilizando escores clínicos amplamente empregados, como o escore de Framingham, o de Framingham Global, o da AHA/ACC, entre outros, não foi elaborada para pacientes portadores de HF. ^22^ De fato, um indivíduo com longa exposição a elevados níveis de colesterol ao longo do tempo (“ *cholesterol years score* ”) não pode ser abordado como eventual baixo risco cardiovascular ao se utilizar um desses escores tradicionais.

Portanto, estudos atuais sobre estratificação de risco na HF precisam considerar, se possível, um desenho prospectivo, o diagnóstico molecular e os efeitos atenuantes pelo uso prévio de estatina. Nessa perspectiva, Paquete et al. ^120^ estabeleceram *o escore de risco de Montreal* para portadores de HF, avaliando 670 indivíduos com diagnóstico molecular confirmado e submetidos a tratamento prévio com estatina. ^120^ Os fatores sexo masculino, idade, hipertensão e tabagismo foram associados de maneira independente com a incidência da DCV aterosclerótica. Mais recentemente, os autores validaram sua equação de risco em outra população de 718 portadores de diagnóstico molecular de HF com boa discriminação estatística; ^121^ entretanto, esses estudos são ainda considerados limitados pelo seu desenho retrospectivo e pela incidência relativamente pequena de eventos.

Perez de Isla et al., ^122^ utilizando o registro prospectivo SAFEHEART ( *Spanish Familial Hypercholesterolemia* ), estabeleceram uma nova equação, acrescentando aos marcadores de risco definidos no escore de Montreal: presença de evento cardiovascular aterosclerótico, índice de massa corporal (IMC) elevado (> 30 kg/m^2^ ), concentração de LDL-c residual elevada (> 100 ou > 160 mg/dl) e níveis de Lp(a) acima de 50 mg/dL. ^122^ O uso desses parâmetros revelou associação independente com um primeiro evento cardiovascular aterosclerótico ou sua recorrência. De fato, o SAFEHEART escore mostrou um bom índice de discriminação (0,85 global e 0,81 na prevenção primária), com excelente calibração para as prevenções primárias e secundárias. Entretanto, ele foi limitado pela incidência relativamente baixa de eventos (5,6%), pela existência de um possível efeito confundidor, pelo uso prévio de estatina e por um período relativamente curto de seguimento. Finalmente, de modo semelhante ao escore de Montreal para HF, a equação do registro SAFEHEART se mostrou limitada pela falta de validação em outras populações com HF. ^123^

#### 5.4.1. Recomendação

1. Uso do escore de Montreal para HF: recomendação classe IIb, nível de evidência B.
2. Uso do escore SAFEHEART: recomendação classe IIa, nível de evidência B.
3. Uso do escore de Framingham ou de outros escores clínicos na HF: recomendação classe III, nível de evidência B.

### 5.5. Escore de Cálcio Coronário

O escore de cálcio coronário (CAC) é uma forma de quantificar a carga total de placa aterosclerótica coronariana: quanto maior o escore de cálcio, maior a carga de placa que o indivíduo apresenta. A maneira mais difundida de avaliar o CAC é pelo método de Agatston, que corresponde à soma ponderada das lesões com densidade acima de 130 unidades Hounsfied (UH). Então, multiplica-se a área do cálcio por um fator relacionado à atenuação máxima da placa: fator 1 – se atenuação máxima < 200 UH; fator 2 – se atenuação máxima entre 200 e 300 UH; fator 3 – se atenuação máxima entre 300 e 400 UH; fator 4 – se atenuação máxima ≥ 400 UH. ^124^

Existem diversos estudos que demonstraram a associação de CAC elevado com evento coronariano. ^125^^,^^126^ Uma meta-análise publicada em 2004 por Pletcher et al. ^127^ mostra uma relação linear entre a quantidade de CAC e o risco de eventos coronarianos. Aqueles com uma pontuação CAC > 400 unidades Agatston (UA) apresentavam risco mais alto de eventos cardiovasculares. Os estudos mais emblemáticos a respeito da associação de CAC com a predição de DAC são o MESA ^125^ e o Heinz Nixdorf Recall Study (HNR), ^126^ os quais mostraram que CAC é um marcador independente de morte e infarto do miocárdio. O escore não apenas adicionou poder discriminativo, mas também melhorou a reclassificação de risco para DAC comparado aos fatores de risco clássicos. No estudo MESA, uma avaliação prospectiva de 6.814 pacientes seguidos por uma média de 3,8 anos, as taxas de risco para um evento coronariano foram 7,73 para aqueles com pontuação CAC 101 a 300 UA e 9,67 para CAC ≥ 300 UA (P < 0,001) em comparação àqueles com CAC ausente. ^125^ Em contrapartida, CAC zero está associado a baixa taxa de eventos coronarianos mesmo em médio prazo (11 anos). ^128^

Não há estudos randomizados para tratamento do perfil lipídico guiado por CAC, mas existem estudos observacionais demonstrando que indivíduos portadores de CAC mais elevado apresentam maior benefício da terapêutica com estatinas. ^129^^,^^130^ A Diretriz Brasileira de Dislipidemia de 2017 recomenda que indivíduos de prevenção primária com escore de cálcio elevado (> 100 UA) sejam considerados como de alto risco cardiovascular e tratados com metas lipídicas pertinentes para tal categoria de risco. ^52^ A diretriz do American College of Cardiology/American Heart Association de 2018 dá um passo adiante e coloca a possibilidade de adiar tratamento com estatina em pacientes de prevenção primária, idade entre 40 e 75 anos, sem diabetes melito, com LDL-c entre 70 e 189 mg/dl e que apresentam CAC zero. ^53^

Os pacientes portadores de HF apresentam CAC mais elevado em comparação aos não HF pareados por sexo e idade. ^118^ Os determinantes da calcificação coronariana na HF são os fatores de risco clássicos para aterosclerose. De fato, Martinez et al. ^118^ demonstraram que a carga de exposição ao LDL-c correspondente ao LDL *years score* (LDL-c multiplicado pela idade), escore de Framingham e sexo masculino estavam associados ao CAC. Um estudo francês também mostrou associação de CAC com colesterol *years score* (colesterol total multiplicado pela idade). ^131^

As diretrizes de tratamento da dislipidemia consideram os pacientes portadores de HF como pelo menos de alto risco cardiovascular por apresentarem LDL-c elevado desde a infância. ^52^^,^^132^ Desse modo, um questionamento que ficaria nesse contexto seria a utilidade do CAC na estratificação de risco da HF, em se tratando de uma população de alto risco cardiovascular. Uma subanálise do estudo MESA mostra associação de CAC com DCV mesmo no contexto de LDL-c elevado (LDL-c > 190 mg/dl). Os pacientes com CAC zero apresentaram baixa taxa de eventos cardiovasculares (risco em 10 anos: 3,7%, risco por ano: 0,4%) quando comparados aos portadores de CAC > 0 (risco em 10 anos: 20%; risco por ano: 2,0%). Os fatores associados a CAC zero foram: idade < 65 anos, sexo feminino e ausência de diabetes melito. A análise da associação de CAC com evento cardiovascular foi feita em uma coorte brasileira de indivíduos portadores de HF com diagnóstico molecular comprovado e em prevenção primária. Esse estudo mostra associação de CAC com evento cardiovascular na HF, mesmo sob tratamento com estatinas de alta potência. ^133^ A média de idade foi de 45 anos, e foram documentados 15 eventos cardiovasculares. A taxa anual de eventos por 1.000 pacientes para CAC zero, 1 a 100 e > 100 foi, respectivamente: 0; 26,4 (IC 95% 12,9 a 51,8) e 44,1 (IC 95% 26,0 a 104,1). Apesar de ser um estudo com tamanho amostral pequeno (n = 206) e tempo de seguimento relativamente curto (mediana 3,7 anos), demonstra que possivelmente CAC zero também pode ser utilizado como marcador de bom prognóstico na população com HF. ^133^

Desse modo, o CAC pode ser considerado uma ferramenta adicional na estratificação de risco de pacientes com HFHe de prevenção primária (classe IIb, nível de evidência B).

### 5.6. Angiotomografia de Coronárias

Comparada ao escore de cálcio, a angiotomografia de coronárias é um exame que apresenta como vantagens a visualização de placas não calcificadas e estimativas do grau de estenose luminal. ^134^ Contudo, as desvantagens seriam: necessidade de infusão de contraste endovenoso, custo mais elevado e dose maior de radiação.

O uso da angiotomografia de coronárias apresenta claros benefícios em pacientes sintomáticos de baixo risco para DAC, tanto na emergência como no âmbito ambulatorial. Isso porque afasta a doença como causadora dos sintomas. ^135^ Entretanto, o benefício dessa metodologia sobre o CAC na estratificação de risco de pacientes assintomáticos é bem discutível. O registro CONFIRM, por exemplo, não demonstrou vantagem da angiotomografia de coronárias sobre o CAC nos indivíduos assintomáticos. ^136^ Porém, dois “subestudos” do CONFIRM mostraram que o exame poderia melhorar a estratificação em relação ao CAC em pacientes de maior risco, em particular nos mais idosos ^137^ e naqueles com CAC de valor intermediário. ^138^

Estudos prévios demonstraram que portadores de HF apresentam maior carga de placas ateroscleróticas na angiotomografia de coronárias, o que é representado por maior número de pacientes com placas, estenose luminal e segmentos com placas, quando comparado a controles normolipidêmicos. ^139^^,^^140^ Essa condição está associada a maior risco de desfechos cardiovasculares em população não portadora de HF. ^141^ A dúvida, então, seria a utilidade da angiotomografia de coronárias nos portadores de HF assintomáticos de prevenção primária. Assim, um pequeno estudo japonês com 101 pacientes portadores de HF demonstrou que um escore tomográfico com base em segmentos com estenose na angiotomografia de coronárias estava associado a eventos cardíacos maiores. ^142^ No entanto, esse estudo não deu uma resposta definitiva devido ao pequeno tamanho da amostra, ao desenho retrospectivo e ao fato de muitos eventos terem ocorrido próximo à realização do exame, o que possivelmente leva à possibilidade de muitos eventos de revascularização terem sido “provocados” por ele. O único estudo clínico randomizado para avaliar influência da angiotomografia de coronárias em desfechos clínicos em pacientes assintomáticos foi realizado em uma população diabética e não demonstrou benefício. ^143^ Diante disso, por enquanto, não é recomendada a realização de angiotomografia de coronárias em portadores de HFHe assintomáticos de prevenção primária (classe III, nível de evidência B).

Entretanto, a utilidade da angiotomografia de coronárias na HFHo merece outro ponto de vista. Isso porque a doença está associada a aterosclerose acelerada e pode cursar com evento cardiovascular e estenose aórtica supravalvar de modo muito precoce. ^13^ Existem estudos com tamanho amostral pequeno demonstrando que a HFHo pode evoluir com aterosclerose coronariana e acometimento aterosclerótico de raiz de aorta detectada pela angiotomografia de coronárias em idade prematura. ^144^^,^^145^

O exame pode ser realizado em portadores de HFHo mesmo assintomáticos desde o diagnóstico, para melhor avaliação do seu perfil de risco cardiovascular, e repetido a critério clínico (recomendação classe IIa, nível de evidência B).

### 5.7. Espessura Íntima Média Carotídea

A espessura íntima média carotídea (EIMC) é definida como a distância entre a interface lúmen-íntima e média-adventícia. Está ligada a fatores de risco cardiovasculares, prevalência e incidência de DCV e grau de aterosclerose em diferentes sítios arteriais. Sua progressão pode ser revertida ou atenuada com intervenção em fatores de risco, em associação a redução de eventos cardiovasculares, ^146^ achados que colocam a EIMC como potencial marcador substituto de aterosclerose. A EIMC já foi estudada na população com HF por Martinez et al., ^118^ que demonstrou maior valor dela no grupo com HF em relação aos controles.

A EIMC foi utilizada na população com HF como um marcador substituto de aterosclerose para avaliação de progressão de aterosclerose com medicação hipolipemiante. ^147^^,^^148^ Mesmo na infância, foi demonstrada maior EIMC nas crianças com HF, se comparadas às não afetadas. Além disso, estudo prévio com pessoas de 8 a 18 anos mostra uma tendência à regressão da EIMC com pravastatina, enquanto uma tendência à progressão foi observada no grupo placebo. ^149^ O uso da rosuvastatina em crianças a partir de 6 anos de idade foi capaz de desacelerar a progressão da EIMC, chegando a igualar os valores após 2 anos de tratamento, em comparação às crianças não afetadas. ^150^

Entretanto, a potencial utilização da EIMC na prática clínica esbarra na variabilidade da metodologia para sua aferição, que inclui: local da medida, influência do ciclo cardíaco (não houve padronização se medida na sístole ou diástole nos diferentes estudos), utilização de medida média ou máxima, definição de EIMC alterada, entre outras dificuldades na uniformização do método. ^151^ Além disso, o estudo MESA comparou a reclassificação de risco entre diversos biomarcadores em pacientes de risco intermediário e encontrou baixo poder de reclassificação da EIMC em comparação ao CAC: *net reclassification improvement* (NRI) da EIMC foi de 0,060, e de CAC foi de 0,406, demonstrando a superioridade deste último método. ^152^

Outra informação que o ultrassom de carótidas pode fornecer é se há placas carotídeas. O risco relativo da presença delas varia de maneira ampla, de 1,16 a 6,71 em diferentes estudos, possivelmente em decorrência das suas diferentes definições: EIMC >1,2 mm; EIMC >1,0 mm com protrusão para lúmen; análise subjetiva; espessamento focal > 50% da EIMC ao redor ou >1,5 mm; entre outras. Além disso, considerar apenas presença ou ausência de placa carotídea pode ser simplista demais frente à diversidade dos fenótipos de placa (calcificada, não calcificada, focal etc.). ^153^ Desse modo, o ultrassom doppler de carótidas em portadores de HF assintomáticos para avaliação da EIMC e pesquisa de placa carotídea pode ser considerado para otimizar sua estratificação de risco cardiovascular (classe IIb, nível de evidência B).

### 5.8. Pesquisa de Isquemia Miocárdica

A pesquisa de isquemia miocárdica com teste ergométrico é indicada em indivíduos que iniciarão atividade esportiva de alta intensidade e/ou competitiva. Estudo prévio com 639 portadores de HFHe diagnosticados por critério clínico encontrou 9% de testes positivos para isquemia miocárdica. Ele também demonstrou que parâmetros do teste ergométrico, como menor capacidade de exercício, atraso na redução da frequência cardíaca no primeiro minuto da recuperação e aumento no pico da pressão de pulso, foram preditores de eventos coronarianos. ^154^ Outro estudo com 194 indivíduos com HF detectou 21% de testes ergométricos positivos. ^155^

Apesar de não haver estudos randomizados com teste ergométrico na HF e de os poucos que existem utilizarem critério diagnóstico clínico, a realização de teste ergométrico periodicamente na HFHe pode ser útil para os indivíduos assintomáticos que desejam iniciar atividade física recreativa ou competitiva, bem como para aqueles que apresentam fatores de risco adicional para doença coronariana ou com início de tratamento hipolipemiante tardio, podendo ser repetido a cada 3 a 5 anos (classe IIb, nível de evidência C). Com base nessas informações, podem ser considerados fatores ou marcadores adicionais de risco em portadores de HF os apresentados na Tabela 3 .

**Tabela 3:** Fatores/marcadores de risco cardiovascular na hipercolesterolemia familiar.

Fator/marcador de risco	Grau de recomendação	Nível de evidência
Diabetes melito	I	B
Hipertensão arterial sistêmica	I	B
Tabagismo	I	B
História familiar de DAC prematura em parentes de 1^º^ grau (homens < 55 anos e mulheres < 60 anos)	I	B
Início de tratamento hipolipemiante após os 40 anos de idade	IIa	B
HDL-c < 40 mg/dl	I	B
Lipoproteína (a) > 50 mg/dL (ou > 125 nmol/L)	IIa	B
Xantoma de tendão de Aquiles	IIb	B
Escore de cálcio > 100 UA ou > percentil 75	IIa	B
Presença de placa aterosclerótica com obstrução > 50% em qualquer território arterial	IIa	C

DAC: doença arterial coronariana; HDL-c: colesterol da lipoproteína de alta densidade.

### 5.9. Como Fazer a Estratificação de Risco Cardiovascular dos Pacientes com Hipercolesterolemia Familiar na Prática Clínica

Conforme discutido previamente, os pacientes com HF apresentam elevado risco de evento cardiovascular na prevenção primária, comparados aos não portadores da doença. Contudo, a presença dos fatores de risco clássicos para DAC aumenta mais ainda esse risco, o que acaba por contribuir para a sua heterogeneidade. Mesmo no cenário da prevenção secundária, existe evidência de que o risco de recorrência de evento cardiovascular é mais do que duas vezes maior na HF após evento-índice, quando comparado ao não portador de HF. ^156^

Santos et al. ^56^ consideraram os portadores de HF de acordo com a presença de fatores de risco adicionais: idade > 40 anos e sem tratamento, tabagismo, sexo masculino, lipoproteína (a) > 50 mg/dl (ou > 125 nmol/L), HDL-c < 40 mg/dL, hipertensão arterial, diabetes melito, história familiar de DAC prematura em parentes de 1^o^ grau (homens < 55 anos e mulheres < 60 anos), doença renal crônica (taxa de filtração glomerular [TFG] < 60 ml/min) e IMC > 30 kg/m^2^ –, que caracterizam situação de maior risco.

Com base na presença de doença aterosclerótica manifesta ou subclínica significativa, fatores de risco adicionais, LDL-c muito elevado, os indivíduos portadores de HF poderão ser classificados em três categorias de risco, conforme a seguir. ^132^

#### 5.9.1. Muito Alto Risco

- Quando diante de doença aterosclerótica clinicamente manifesta, assim definida como: infarto prévio do miocárdio, angina *pectoris* , revascularização miocárdica prévia, acidente vascular isquêmico ou transitório ou claudicação intermitente.
- Na presença de doença subclínica aterosclerótica avançada diagnosticada com: escore de cálcio superior a 100 UA ou 75% do percentil para a idade e sexo, ou angiotomografia computadorizada coronariana apresentando obstruções coronárias em mais de 50% ou a presença de placas não obstrutivas em mais de um vaso.

#### 5.9.2. Alto Risco

- Na prevenção primária da HFHe, com LDL-c > 400 mg/dl, mesmo sem fatores de risco.
- Na prevenção primária da HFHe, mas com fatores adicionais de risco.

Obs.: se o LDL-c for > 310 mg/dl com uma das situações de alto risco; LDL-c > 190 mg/dl com duas das condições de alto risco.

#### 5.9.3. Risco Intermediário

- Na prevenção primária da HFHe, sem fatores adicionais de risco.

## 6. Recomendações Nutricionais

O seguimento de padrão alimentar saudável é fundamental no tratamento da HF, uma vez que a dieta inadequada pode amplificar o risco cardiovascular já estabelecido nesses pacientes. ^157^ A última diretriz para o tratamento dietético da doença, elaborada em conjunto com o American College of Cardiology (ACC), a American Heart Association (AHA) e outras sociedades americanas, ^53^ é embasada na diretriz publicada por essas mesmas sociedades em 2013. ^158^ Recomenda-se o seguimento de padrões alimentares saudáveis, com valor calórico adequado, exclusão de ácidos graxos *Trans* , adequação de ácidos graxos saturados (SAT) e estímulo ao consumo de ácidos graxos monoinsaturados (MONO) e poli-insaturados (POLI) em quantidades adequadas. ^158^ As ações desses ácidos graxos sobre o colesterol plasmático foram exaustivamente avaliadas em diversos estudos experimentais, clínicos e epidemiológicos. ^158^^,^^159^ Muitos dos resultados controversos encontrados foram devido às diferenças quanto à duração dos estudos, ao tamanho da amostra e ao tipo de nutriente utilizado para comparação. ^132^ Além disso, os diferentes ácidos graxos são provenientes de diversas matrizes alimentares, como carnes, leite, óleos ou alimentos industrializados, podendo, assim, induzir efeitos distintos sobre os lípides do plasma. ^160^

Nos últimos anos, o tipo de alimento consumido tem sido mais valorizado que o nutriente utilizado isoladamente. Desse modo, padrões alimentares como a dieta do Mediterrâneo ^161^^,^^162^ e a dieta DASH, ^163^ que contemplam a inclusão de alimentos como grãos, frutas, hortaliças, carnes magras, produtos lácteos com menor teor de gorduras e frutas oleaginosas (nozes e castanhas), passaram a ser recomendados pelas principais diretrizes internacionais. Além disso, indica-se para o preparo dos alimentos o uso moderado de óleos vegetais ricos em POLI (ômega 3 e ômega 6) e MONO (ômega 9). ^53^ A recomendação deles no preparo dos alimentos é evidenciada em dois importantes estudos. O recente *Cohorts for Heart and Aging Research in Genomic Epidemiology* (CHARGE) mostrou que a concentração plasmática e tecidual de biomarcadores de ácidos graxos ômega 6 associou-se a menos eventos cardiovasculares. ^164^ O mesmo resultado foi observado com biomarcadores plasmáticos das séries ômega 3 e ômega 6, que se associaram a menor risco cardiovascular. ^165^

A Diretriz da European Society of Cardiology (ESC), elaborada em conjunto com a European Atherosclerosis Society (EAS), ^132^ reitera essas recomendações e adverte que alimentos como óleo de palma, óleo de coco, bacon, biscoitos, produtos de panificação ricos em gordura e produtos lácteos integrais devem ser consumidos ocasionalmente e em quantidades mínimas por indivíduos com HF. ^53^

### 6.1. Colesterol Alimentar

Nos últimos anos, tanto as diretrizes da AHA ^53^^,^^158^ como o Guia Alimentar Americano (2015-2020) ^166^ não mais estabelecem limite máximo de consumo de colesterol, em razão das poucas evidências associando sua ingestão a doença ateroslerótica ^167^ e DAC. ^168^ Apesar disso, no Guia Alimentar Americano, é sugerida a importância de considerar o colesterol da dieta no sentido de estabelecer padrões alimentares saudáveis, o que vai ao encontro das recomendações do Instituto de Medicina, ^169^ que ressaltaram o benefício do baixo consumo de colesterol.

O colesterol alimentar apresenta menor ação hipercolesterolêmica quando comparado aos ácidos graxos saturados, razão pela qual os guias alimentares enfatizaram suas orientações para redução do consumo desse ácido graxo. Assim, a diminuição de saturados de origem animal garante menor taxa de colesterol, uma vez que ambos se encontram nos mesmos alimentos. De modo geral, o balanço entre ingestão e síntese endógena de colesterol é responsável pela homeostase da colesterolemia; ^170^ entretanto, o aumento da sua ingestão pode contribuir significativamente para a elevação da concentração plasmática de LDL-c, ^171^ resposta sujeita a grande variabilidade interpessoal, dependente de fatores metabólicos e genéticos. ^170^^,^^172^^,^^173^

De fato, a Diretriz 2018 AHA/ACC/Guideline on the Management of Blood Cholesterol, que referencia o mesmo documento publicado em 2014, informa não haver evidência para determinar se a redução do colesterol alimentar reduz o LDL-c. ^158^

Os estudos observacionais e as meta-análises que avaliaram a influência do colesterol da dieta no risco de desenvolvimento de diabetes tipo 2, DAC e AVE não são claros, ^168^^,^^171^^,^^173^^-^^177^ ou mostram ausência de associação com DCV e mortalidade. ^178^ No entanto, uma pesquisa que avaliou o banco de dados dos estudos Atherosclerosis Risk in Communities (ARIC), Coronary Artery Risk Development in Young Adults (CARDIA), Framingham Heart Study (FHS), Framingham Offspring Study (FOS), Jackson Heart Study (JHS) e Multi-ethnic Study of Atherosclerosis (MESA) mostrou que o aumento do consumo de colesterol está ligado de forma dose-dependente ao aumento de DCV e de mortalidade total, ^179^ possivelmente em razão de seu impacto nas concentrações de LDL-c. ^180^

Com relação ao ovo, apesar de ser fonte de colesterol, é também altamente nutritivo, com perfil excelente de proteínas de alto valor biológico, vitaminas e minerais, além de grande viabilidade econômica. Diante de tais qualidades nutricionais, recomenda-se sua inclusão na dieta, desde que integrante de um padrão alimentar saudável.

O aumento de mortalidade e de eventos cardiovasculares em decorrência de maior consumo de colesterol total e de ovo é independente da qualidade da dieta consumida. Portanto, recomenda-se moderação no consumo de ovos e outras fontes de colesterol, ^179^ especialmente entre indivíduos com maior concentração de lipídios plasmáticos e entre aqueles hiper-responsivos ao consumo de colesterol.

### 6.2. Ação dos Ácidos Graxos sobre a Colesterolemia

#### 6.2.1. Saturados

Os ácidos graxos classificam-se em SAT, MONO, POLI ou *Trans* e influenciam de forma distinta a concentração plasmática de colesterol total e LDL-c. Dentre os principais ácidos graxos saturados presentes nos alimentos em geral, têm-se: láurico (12:0), mirístico (14:0), palmítico (16:0) e esteárico (18:0), mas apenas o palmítico é encontrado amplamente na natureza. O óleo de coco é fonte exuberante dos ácidos láurico e mirístico, enquanto as carnes apresentam conteúdo elevado de palmítico, e o leite, de esteárico e mirístico. Produtos de origem vegetal, como a palma e o cacau, também apresentam, de forma respectiva, conteúdo elevado dos ácidos graxos palmítico e esteárico. ^181^^,^^182^ Os ácidos graxos pentadecílico (15:0) e magárico (17:0) são encontrados, em pequena quantidade, em derivados lácteos, e a sua concentração plasmática é um marcador de consumo deles. ^183^^,^^184^ Além disso, os alimentos também fornecem ácidos graxos de cadeia mais longa, como araquídico (20:0), behênico (22:0) e lignocérico (24:0), os quais são encontrados em frutas oleaginosas, como amendoim e macadâmia. ^185^^,^^186^

Diversos estudos clínicos e epidemiológicos mostraram aumento do risco cardiovascular com maior consumo de SAT, ^186^^-^^188^ por induzirem aumento nas concentrações plasmáticas de colesterol total e LDL-c. ^159^ Vários mecanismos foram propostos para justificar essa ação, como: (a) apresentam cadeia retilínea de carbono, empacotando-se no centro das lipoproteínas, o que possibilita a acomodação de maior quantidade de colesterol; ^189^ (b) em associação ao colesterol, SAT reduz a atividade, a proteína e o mRNA do LDLR, ^190^^-^^192^ alteração que diminui a metabolização das partículas de LDL. ^193^^,^^194^

A Organização Mundial da Saúde (OMS) publicou uma revisão sistemática de estudos clínicos (consumo médio de SAT: 9,8%) e mostrou que a substituição isocalórica de ácidos graxos saturados por POLI ou MONO reduziu as concentrações plasmáticas de colesterol total e de LDL-c. ^159^ Além disso, foi demonstrado que a substituição de carboidratos por ácido palmítico, mirístico ou láurico também induziu uma elevação dos mesmos parâmetros lipídicos, efeito não observado com ácido esteárico. Isso reforça a afirmação de que ácidos graxos saturados se comportam diferentemente em relação ao seu efeito sobre os lipídios plasmáticos. O ácido esteárico não eleva o colesterol do plasma porque é rapidamente convertido a ácido oleico sob ação da enzima hepática Stearoil CoA Dessaturase 1 (SCD1). ^195^

O estudo Prospective Urban Rural Epidemiology (PURE) ^196^ avaliou a dieta de 135.000 pessoas em 18 países, mostrando que o maior consumo de gorduras (35% das calorias) está relacionado com menor mortalidade, em comparação com o menor consumo (10% das calorias). Vale ressaltar que esse percentual de gorduras está dentro da faixa de recomendação (25 a 35% do valor calórico total, VCT) que vem sendo preconizada nas últimas décadas, e que a mediana do consumo variou entre 2,8 e 13,2% das calorias. Nesse estudo, foi demonstrado que o aumento do consumo de SAT associou-se à elevação do LDL-c. ^196^

Uma revisão sistemática publicada pela Biblioteca Cochrane em 2015, que avaliou os dados de estudos clínicos randomizados, totalizando 59.000, mostrou que a diminuição do consumo de saturados em relação à dieta habitual reduziu em 17% os eventos cardiovasculares. ^197^ Além disso, os resultados evidenciaram redução dos mesmos eventos em 27% quando SAT é substituído por POLI.

Se determinados ácidos graxos saturados relacionam-se com maior risco cardiovascular, outros apresentam associação inversa, como o pentadecílico (15:0) e o magárico (17:0), ^183^ bem como aqueles de cadeia muito longa, como lignocérico, behênico e araquídico. ^185^

Assim, recomenda-se consumo de até 10% das calorias na forma de ácidos graxos saturados, com limite de 7% para indivíduos hipercolesterolêmicos, de acordo com a ESC/EAS 2019 ^132^^,^^158^ e o ACC/AHA.

#### 6.2.2. Insaturados

O principal ácido graxo monoinsaturado é o ácido oleico (18:1), cujas fontes são azeite de oliva, óleo de canola e frutas e sementes oleaginosas. Os ácidos graxos poli-insaturados da série ômega 6 são: o linoleico (18:2), encontrado nos óleos de milho, girassol, soja e canola, e o araquidônico (20:4), presente em ovos, óleo de peixe e carne. ^198^ Os ácidos graxos da série ômega 3 podem ser de fonte vegetal ou animal, sendo o **α** -linolênico (18:3) encontrado nos óleos vegetais, como de linhaça, canola e soja, e o eicosapentaenoico (20:5) e docosa-hexaenoico (20:6) encontrados em peixes de águas muito frias.

Em substituição aos saturados, os MONO e POLI não elevam a concentração plasmática de colesterol e de LDL-c, um benefício que parece ser maior com POLI, uma vez que ácidos graxos monoinsaturados exibem ação neutra sobre a colesterolemia. ^158^

#### 6.2.3. Ácidos Graxos Ômega 3

Diversos estudos clínicos mostram que a suplementação com doses farmacológicas (2 a 4 g) de ácidos graxos eicosapentaenoico (EPA) e docosahexaenoico (DHA) ao dia pode diminuir as concentrações plasmáticas de triglicerídeos em até 25 a 30%, além de aumentar discretamente as de HDL-c (1 a 3%). Pode também elevar as concentrações de LDL-c em até 5 a 10%, com pouca ou nenhuma diferença sobre o colesterol total sérico. ^199^^-^^202^

No entanto, quando utilizadas formulações contendo exclusivamente ácidos graxos ômega 3 purificados, esse aumento de LDL-c pode não ocorrer, conforme demonstrado no Japan EPA Lipid Intervention Study (JELIS). ^203^^,^^204^ Pesquisas sobre o efeito do ômega 3 em pacientes com HF ainda são muito escassas, e parece que sua suplementação pode influenciar a progressão da aterosclerose nesses pacientes de alto risco, carecendo de mais investigação. ^205^^-^^207^

#### 6.2.4. Ácidos Graxos *Trans*

O consumo de ácidos graxos *Trans* presentes nos alimentos industrializados aumenta o risco cardiovascular porque induz um perfil lipídico aterogênico por elevar a concentração plasmática de LDL-c, diminuir o catabolismo de Apo B, ^208^ reduzir o HDL-c e induzir aumento do catabolismo da Apo A-I. ^209^ Além disso, os ácidos graxos *Trans* aumentam a gravidade das lesões ateroscleróticas na DAC ^209^ e induzem disfunção endotelial. ^210^

Os resultados de estudos controlados discutidos em uma meta-análise mostraram que, a cada 1% de substituição das calorias de *Trans* por saturados, poli-insaturados ou monoinsaturados, houve redução da razão Col/HDL, respectivamente, em 0,31, 0,54 e 0,67. ^211^ Já a avaliação dos estudos prospectivos da mesma pesquisa revelou que, a cada 2% de substituição de *Trans* pelos demais ácidos graxos, diminui em 17% o risco de DAC. ^211^ Posteriormente, demonstrou-se que o maior consumo de *Trans* observado em um período de 10 anos em diversos países está associado a um aumento de 4% na mortalidade por doença coronariana. ^212^ Em outro estudo, foi avaliada a taxa de mortalidade atribuída à dieta em 195 países, e observou-se considerável número de mortes por causa cardiovascular atribuídas ao consumo de ácidos graxos *Trans* . ^213^ Além dos efeitos adversos sobre o metabolismo lipídico, o *Trans* é capaz de induzir perfil pró-inflamatório, agravando ainda mais os seus efeitos deletérios. ^214^ Por todas as suas ações adversas, os ácidos graxos *Trans* utilizados em alimentos industrializados devem ser excluídos da dieta. ^53^^,^^158^^,^^208^^,^^213^^,^^215^

#### 6.2.5. Fitosteróis

Os fitosteróis, fitostanóis e seus ésteres são componentes bioativos presentes em alimentos vegetais, cuja estrutura química é muito similar à do colesterol. ^216^ Seu efeito hipocolesterolemiante está bem documentado na literatura científica. Após incorporação às micelas, os fitosteróis são transportados para o interior do enterócito via proteína transportadora NPC1-L1, e, sequencialmente, a maior parte retorna ao lúmen intestinal por meio dos transportadores ABCG5/ABCG8, ^217^ mantendo baixas as suas concentrações plasmáticas. O mecanismo de redução da concentração plasmática de colesterol total e LDL-c é explicado pela maior solubilidade dos fitosteróis nas micelas, o que desloca colesterol, promovendo sua excreção. O consumo médio de fitosterol na população é de 100 a 400 mg/dia, mas um estudo brasileiro revelou um consumo médio de 160 mg/dia. ^218^ Já se demonstrou que a suplementação diária de 2 g de fitosteróis reduz as concentrações plasmáticas de LDL-c entre 8 e 10%. ^217^^,^^219^ Apesar do seu efeito pequeno sobre a redução da colesterolemia, a diretriz atual do ESC/EAS ^53^ indica que seu uso pode ser benéfico para adultos e crianças com elevação moderada de colesterol. ^217^ Entretanto, é importante salientar que nem todos os indivíduos respondem igualmente ao uso de fitosteróis; portanto, sua eficácia deve ser avaliada individualmente. ^220^

Os fitosteróis podem ser administrados por meio de cápsulas contendo entre 650 e 900 mg ou creme vegetal; duas colheres de sopa fornecem a dose recomendada. Seu uso deve ser acompanhado de alimentação e hábitos de vida saudáveis para obtenção dos efeitos almejados. O consumo do fitosterol junto das refeições principais parece ser a melhor opção, em razão do mecanismo de ação de competição na absorção do colesterol da dieta. ^221^

Em pacientes portadores de HF, o uso de fitosterol pode auxiliar no alcance de metas para o LDL-c, quando usado em associação com estatina/ezetimiba. ^222^ Dados de meta-análise sobre o efeito dos fitoesteróis em crianças demonstraram redução do colesterol total (7 a 11%) e do LDL-c (10 a 15%). ^223^ De acordo com *o Expert Panel on Integrated Guidelines for Cardiovascular Health and Risk Reduction in Children and Adolescents* , a suplementação de fitosteróis (2 g/dia) pode ser boa opção para crianças e adolescentes com HF que ainda não podem receber tratamento farmacológico, ^224^ pois eles são bem tolerados e não apresentam efeitos adversos significativos. No Brasil, os fitosteróis são aprovados para uso pediátrico a partir dos 5 anos de idade; porém, poucos estudos testaram o seu uso na gestação e lactação, sendo recomendada cautela nesses casos. ^225^ É importante salientar que seu uso é contraindicado para os portadores de sitosterolemia.

#### 6.2.6. Fibras

O consumo alimentar de fibras parece estar associado à redução significativa do colesterol total, por meio de mecanismos que envolvem: 1) redução da absorção de colesterol induzida pela viscosidade; ^226^ 2) aumento da excreção fecal de colesterol e ácido biliar, ^227^ induzindo maior atividade da 7 **α** -hidroxilase, ^228^ enzima-chave na formação de ácidos biliares a partir de colesterol; ^229^ 3) redução da atividade da 3-hidroxi-3- metilglutaril coenzima A (HMG-CoA) redutase; ^230^ 4) redução do conteúdo hepático de colesterol; ^231^ 5) alteração da composição da microbiota intestinal, levando a maior produção de ácidos graxos de cadeia curta e aumento da excreção de esteroides neutros e ácidos biliares; ^232^ ou mesmo associação entre os mecanismos descritos.

Uma meta-análise de estudos que investigaram o efeito do consumo de fibra solúvel ( **β** -glucana) sobre lipídios plasmáticos verificou que a sua ingestão (> 3 g/dia) promoveu modesta redução das concentrações plasmáticas de colesterol total e LDL-c (11,6 e 9,6 mg/dl, respectivamente, em relação ao controle), sem efeito em HDL-c e triglicerídeos. ^233^ Revisão sistemática da Biblioteca Cochrane ^234^ avaliou o efeito das fibras na prevenção primária de DCV e observou redução de colesterol total e LDL-c (7,7 e 8,5 mg/dl, respectivamente) devido ao aumento do consumo de fibras. Todavia, os autores ressaltam que estudos de coorte randomizados, bem conduzidos e com maior tempo de duração são fundamentais para se determinar com segurança o efeito das fibras na saúde cardiovascular. ^234^

Em consonância com a atual diretriz do ESC/EAS, ^53^ recomenda-se uma dieta rica em fibras, especialmente solúveis. Elas estão presentes em legumes, frutas, verduras e grãos integrais, contemplando um padrão alimentar saudável.

#### 6.2.7. Soja

Os resultados controversos dos estudos que avaliaram a ação da suplementação de proteína de soja, livre de gorduras, com ou sem isoflavonas, sobre as concentrações plasmáticas de colesterol total e LDL-c podem, em parte, ser atribuídos às diferentes metodologias utilizadas nos estudos, à presença de outras substâncias, como fibras e fosfolipídios e à utilização de diferentes concentrações de proteína de soja ou isoflavonas. ^235^^,^^236^ As evidências sugerem ser a proteína da soja a responsável pela modesta redução alcançada (~3%) nas concentrações plasmáticas de colesterol, e não a isoflavona isolada. ^237^ De toda forma, não há evidência para a indicação de suplementos de isoflavona no tratamento da hipercolesterolemia. No entanto, produtos derivados de soja têm baixas concentrações de saturados e são ricos em fibras, vitaminas, minerais e ácidos graxos insaturados, podendo fazer parte de um plano alimentar saudável.

#### 6.2.8. Chocolate

O cacau é extraído da semente do cacaueiro (Theobromacacao L.), originário principalmente da América do Sul e Costa Oeste da África. O chocolate é obtido a partir da mistura de derivados de cacau (massa de cacau, cacau em pó ou manteiga de cacau) com outros ingredientes, como açúcar, leite, lecitina, castanhas e frutas. Desse modo, além de ter alta densidade calórica, ele é também rico em gorduras e açúcares. Cerca de 60% da gordura do cacau é constituída pelos ácidos graxos saturados esteárico e palmítico, e aproximadamente 30% estão sob a forma de ácido oleico. Os poli-insaturados compreendem em torno de 3 a 5% dos ácidos graxos presentes no cacau. ^238^ Já está descrito que o alto consumo de saturados eleva as concentrações plasmáticas de colesterol; entretanto, os resultados de duas meta-análises mostraram que o chocolate, rico em cacau (escuro), parece não exercer ação hipercolesterolêmica, ^239^^,^^240^ possivelmente pelo fato de o esteárico, principal ácido graxo do cacau, ser rapidamente convertido a ácido oleico por meio da ação da SCD1 no fígado. ^241^ Contudo, é importante ressaltar que o chocolate pode ser importante fonte de açúcar simples e calorias; por isso, seu consumo deve ser orientado com cautela, de modo que não contribua para o ganho de peso. Além disso, o chocolate é normalmente produzido com outras fontes de gorduras.

#### 6.2.9. Óleos Tropicais

##### 6.2.9.1. Óleo de Coco

O óleo de coco ( *Coco nucifera* ) é composto principalmente por gordura saturada (82%), da qual 42% estão sob a forma de ácido láurico, 16% como mirístico, 9% como palmítico, e o restante é composto por caprílico, cáprico e esteárico. ^242^ O óleo de coco contém baixa concentração de insaturados e não apresenta o ácido graxo essencial **α** -linolênico (18:3). ^181^^,^^243^ Quando comparado ao consumo de óleo de oliva ^244^ e cártamo, ^245^ o óleo de coco aumenta as concentrações plasmáticas de colesterol total e LDL-c. Estudo em indivíduos normolipidêmicos habitantes do Sri Lanka evidenciou que a substituição isocalórica do óleo de coco por óleo de soja reduziu as concentrações plasmáticas de colesterol total, LDL-c e triglicerídeos. ^246^ Resultados semelhantes foram observados em indivíduos dislipidêmicos, com substituição do óleo de coco por óleo de milho. ^247^ O aumento das concentrações de HDL-c observado com o consumo de óleo de coco foi acompanhado de elevação concomitante de LDL-c, um dos principais fatores de risco cardiovascular. ^248^

Além disso, por ser rico em ácido láurico, ^249^ o óleo de coco pode acionar vias de sinalização inflamatória, por meio da ativação de receptores ligados à resposta imune inata, os *toll-like receptors* (TLR). ^249^^-^^251^ Estudo em macrófagos observou que o ácido láurico aumentou a expressão de ciclo-oxigenase 2 (COX 2) por meio da ativação das vias NF- **κ** B/TLR 2 e 4. ^252^ Com relação à capacidade oxidativa, não foi observada diferença no metabolismo energético e oxidação lipídica ao se comparar o efeito agudo do consumo de óleo de coco ou azeite de oliva em mulheres com sobrepeso. ^253^

##### 6.2.9.2. Óleo de Palma

Apesar de se tratar de óleo vegetal, cerca de 50% do óleo de palma é composto por ácidos graxos saturados (45% de palmítico e 5% de esteárico), e o restante, por insaturados (40% oleico e 10% linoleico). Com isso, sua maior ingestão, seja adicionado ao preparo de alimentos ou por meio do consumo de produtos industrializados, eleva as concentrações de saturados na dieta. O consumo de óleo de palma, quando comparado ao de óleos vegetais ricos em insaturados, aumenta as concentrações plasmáticas de colesterol total e LDL-c. ^254^ Resultados de meta-análise que comparou o consumo de óleo de palma ao de óleos vegetais, como de canola, soja e oliva, mostraram que o óleo de palma elevou as concentrações de colesterol total, LDL-c e HDL-c, este último de forma modesta. ^255^ Isso mostrou que, em relação aos lipídios plasmáticos, o óleo de palma se comporta de maneira análoga às gorduras de origem animal, a qual é rica em saturados. ^255^^,^^256^ Sendo assim, seu consumo deve ser mantido dentro do percentual de recomendação de consumo de saturados.

Até o momento, em linha com diretrizes do ACC/AHA ^158^ e da ESC/EAS, ^53^ o uso dos óleos tropicais em substituição aos óleos vegetais ricos em ácidos graxos insaturados não é indicado.

#### 6.2.10. Lácteos

O leite e seus derivados são importantes fontes de cálcio e proteína de alto valor biológico. Contudo, o consumo dos integrais eleva a ingestão de ácidos graxos saturados, especialmente mirístico, que apresenta forte correlação com o aumento da concentração plasmática de colesterol. ^159^ Entretanto, no estudo MESA, o consumo de lácteos foi associado a redução do risco cardiovascular ^183^ e não se associou ao aumento do risco de AVE. ^257^ Mais recentemente, dados do estudo European Prospective Investigation into Cancer and Nutrition (EPIC) – Italy, conduzido em 45.009 indivíduos com seguimento de 14,9 anos, mostraram que o consumo de 160 mL a 200 mL de leite está ligado a menor mortalidade por todas as causas, mas esse benefício se perde com ingestão superior a 200 mL. ^258^ No mesmo estudo, demonstrou-se que o consumo de leite desnatado está relacionado com menor mortalidade por causa cardiovascular.

#### 6.2.11. Manteiga

Em uma porção de manteiga (10 g), cerca de 51,5% dos ácidos graxos encontram-se sob a forma de saturados, com predomínio de palmítico (24%), esteárico (10%), mirístico (8%) e láurico (2%). Já os monoinsaturados contribuem com cerca de 22% dos ácidos graxos da manteiga, e os insaturados, com apenas 1,5%. ^259^

Um estudo randomizado demonstrou que o consumo de manteiga aumentou as concentrações plasmáticas de colesterol total, LDL-c e Apo B, comparado ao consumo do mesmo percentual de insaturados. ^260^ O estudo de coorte Multi-Ethnic Study of Atherosclerosis (MESA), que acompanhou por 20 anos cerca de 6.800 indivíduos não diabéticos, sem DCV prévia, ^181^ o maior consumo de manteiga (até 5 g/dia) não se associou a DCV. Estudo conduzido em idosos mostrou resultado semelhante. ^261^ O consumo de manteiga foi ainda inversamente associado à incidência de diabetes melito 2 em estudo de coorte prospectivo. ^262^ Seu efeito foi avaliado em revisão sistemática de estudos de coorte de alto grau de evidência, o qual mostrou que o consumo médio de 14 g/dia não se associou a riscos de DCV. ^263^

O uso de manteiga deve respeitar as recomendações de consumo de saturados, permanecendo menos de 10% do VCT e ainda mais reduzido (menos de 7%) para pessoas com hipercolesterolemia (EAS/ESC 2019). ^53^ Além disso, a fim de evitar ganho de peso e desenvolvimento de obesidade, é preciso levar em consideração as calorias contidas no produto, cujo consumo deve estar inserido em padrão alimentar saudável, rico em frutas, verduras, legumes e grãos integrais. ^53^

### 6.3. Recomendações de Consumo Alimentar no Controle da Hipercolesterolemia Familiar

As recomendações de consumo alimentar em pacientes com hipercolesterolemia familiar são apresentadas na Tabela 4 .

**Tabela 4:** Recomendações de consumo alimentar no controle da hipercolesterolemia familiar

Recomendação	Grau de recomendação	Nível de evidência
**Seguimento de padrão alimentar saudável:** adequação calórica, inclusão de grãos, frutas, hortaliças, carnes magras e produtos lácteos com menor teor de gorduras	I	A
**Colesterol alimentar:** < 300 mg/dia	IIa	A
**Ácidos graxos saturados:** < 7% do VCT	I	A
**Ácidos graxos Trans:** deve ser excluído da dieta	III	A
**Chocolate:** quando rico em cacau, não está relacionado com o aumento do colesterol	I	B
**Óleos tropicais: consumo ocasional, em porções mínimas**	III	B
**Ovo:** consumo moderado, não excedendo as recomendações diárias de colesterol	IIa	A
**Fitosterol:** 2 g/dia proporciona redução moderada do colesterol (↓~10%)	I	A
**Fibras:** redução do colesterol total e LDL-C (↓~5%)	I	A

VCT: valor calórico total; LDL-c: colesterol da lipoproteína de baixa densidade.

## 7. Tratamento Farmacológico da Hipercolesterolemia Familiar Heterozigótica

O tratamento dos portadores de HF grave é um ponto crucial. Considerando-se as complicações oriundas da evolução da doença e da precocidade com que algumas delas ocorrem, deve ser iniciado o mais rápido possível após o diagnóstico. O conceito do colesterol cumulativo ao longo da vida justifica essa abordagem.

### 7.1. Metas Terapêuticas para o LDL-c

É fundamental considerar a existência de condições de alto risco, assim discriminadas: idade superior a 40 anos sem tratamento prévio, tabagismo, sexo masculino, Lp (a) maior que 50 mg/dL (> 125 nmol/L), HDL-c < 40 mg/dL, percentil do escore de cálcio coronário (CAC) calculado pelo critério do MESA.

Dessa maneira, foi proposto que as metas para tratamento do LDL-c sejam assim contempladas: para HF de alto risco, LDL-c > 400 mg/dL ou LDL-c > 310 mg/dL com uma das situações de alto risco anteriormente descritas, * ou LDL-c > 190 mg/dL com duas das condições de alto risco. ^56^ ** Dessa forma, deve-se buscar reduzir o LDL-c em pelo menos 50%, sendo ideal a meta de LDL-c < 70 mg/dL.

Assim, o risco cardiovascular e as metas lipídicas são avaliados de acordo com a presença de DASCV, fatores de risco maiores ou níveis basais de LDL-c, e classificados conforme a seguir.

#### 7.1.1. Metas Terapêuticas no Muito Alto Risco

- Quando diante de doença aterosclerótica clinicamente manifesta, assim definida quando houver infarto prévio do miocárdio, angina *pectoris* , revascularização miocárdica prévia, acidente vascular isquêmico ou transitório ou claudicação intermitente, devem ser consideradas reduções de no mínimo 50%, sendo ideal obter valores de LDL-c < 50 mg/dL. ^132^
- Na presença de doença subclínica aterosclerótica avançada, diagnosticada como CAC superior a 100 UA ou 75% do percentil para idade e sexo, ou angiotomografia computadorizada coronariana apresentando obstruções coronárias > 50% ou a presença de placas não obstrutivas em mais de um vaso, deve-se buscar reduzir o LDL-c em ao menos 50%, sendo o ideal atingir níveis < 50 mg/dL.

#### 7.1.2. Metas Terapêuticas no Alto Risco

- Na prevenção primária da HFHe com LDL-c > 400 mg/dL, mesmo sem fatores de risco, deve-se buscar reduzir o LDL-c em ao menos 50%, sendo ideal chegar a níveis < 70 mg/dL.
- Na prevenção primária da HFHe com fatores adicionais de risco, ^56^ devem ser consideradas reduções de no mínimo 50%, sendo ideais os valores de LDL-c < 70 mg/dL. ^132^

Obs.: Se LDL-c for > 310 mg/dL com uma das situações de alto risco; LDL-c > 190 mg/dL com duas das condições de alto risco. ^56^

#### 7.1.3. Metas Terapêuticas no Risco Intermediário

Na prevenção primária da HFHe sem fatores adicionais de risco, ^1^ devem ser consideradas reduções de no mínimo 50%, sendo ideais valores de LDL-c < 100 mg/dL. É preciso reavaliar periodicamente para verificar a instalação de fatores de risco.

### 7.2. Tratamento Farmacológico

#### 7.2.1. Estatinas

As estatinas – inibidores da hidroximetilglutaril-coenzima A redutase – são os fármacos de primeira escolha no tratamento da HFHe. Mesmo na ausência de evidências científicas de estudos que tenham avaliado o benefício do uso de estatinas exclusivamente em pacientes HFHe, é plenamente reconhecida a contribuição dos pacientes com HFHe para o entendimento atual do metabolismo do LDL-c e a sua expressiva participação nos grandes ensaios clínicos ^264^ (grau de recomendação: I; nível de evidência: C).

Os objetivos terapêuticos recomendados aos portadores de HFHe, enquanto pacientes classificados como de alto e muito alto risco, são reduções percentuais ≥ 50% nos níveis de LDL-c prévios ao tratamento, e o alcance das metas preconizadas de acordo com o risco estratificado. Na HFHe, as estatinas de alta intensidade, como rosuvastatina e atorvastatina, nas doses máximas toleradas são as opções preferenciais e devem ser utilizadas (grau de recomendação: I; nível de evidência: A). ^53^

São propriedades farmacológicas desejáveis das estatinas: inibição enzimática potente e reversível, seletividade pelos hepatócitos, baixa biodisponibilidade para reduzir efeitos adversos sistêmicos, meia-vida de eliminação prolongada e mínimo ou nenhum metabolismo hepático para evitar interações droga-droga. Seu mecanismo de ação se dá pela inibição enzimática, que, ao reduzir a síntese endógena do colesterol intra-hepático, promove o estímulo à síntese e expressão dos receptores de LDL-c, aumentando a captação do LDL pelos hepatócitos, diminuindo as concentrações plasmáticas. ^22^

O tratamento com estatinas demonstrou reduzir eventos isquêmicos coronários, necessidade de revascularização do miocárdio, AVE e mortalidade por causas cardiovasculares em todos os subgrupos, incluindo aqueles com aterosclerose manifesta, diabéticos, hipertensos, idosos e mulheres, diminuindo ainda a mortalidade total em pacientes de alto e muito alto risco cardiovascular (grau de recomendação: I; nível de evidência: A). Esses benefícios são atribuídos à redução dos níveis de LDL-c e considerados um efeito da classe terapêutica. ^52^

As estatinas são seguras, apresentando como efeito colateral mais frequente a mialgia, com ou sem elevação da creatinoquinase (CK). Rabdomiólise é a ação adversa mais grave e a mais rara, cujo risco se incrementa quando da associação aos fibratos. A frequência de efeitos adversos é proporcional às doses utilizadas. ^265^

Quando a eficácia não é suficientemente obtida com o uso de estatina isolada, a possibilidade de se incrementarem as reduções do LDL-c pode ser contemplada com a associação de outras terapias adjuvantes.

#### 7.2.2. Terapia Adjuvante às Estatinas

Pacientes com HF são considerados de alto risco cardiovascular, e estatinas potentes em altas doses permanecem como o pilar no tratameto da dislipidemia e na terapêutica para redução do risco destes pacientes. ^266^^-^^269^ Entretanto, a maioria dos pacientes com HF permanecerá fora das metas, apesar da máxima terapêutica tolerada com estatina. Em um estudo transversal de 1.249 pacientes com HFHe confirmada na Holanda, país com a maior taxa de diagnóstico de HF, apenas 21% apresentavam LDL-c < 100 mg/dl, embora 96% dessa amostra estivessem em uso de estatina. ^270^ Portanto, frequentemente será necessária a adição de um ou mais hipolipemiantes que não a estatina para alcançar as metas desejadas. ^271^

##### 7.2.2.1. Ezetimiba

A ezetimiba reduz seletivamente a absorção intestinal do colesterol dietético e biliar, agindo no transportador Niemann-Pick C1-Like 1 (NPC1L1) no enterócito. Após a ingestão de ezetimiba, ocorre rápida absorção (2 a 3 horas), e no fígado sofre um processo de glicuronidação, transformando-se em glicuronídeo ativo que se localiza na borda do enterócito e retorna à circulação entero-hepática (20%). O glicuronídeo conjugado é hidrolisado e absorvido e é igualmente efetivo na inibição da absorção do esterol. A reciclagem entero-hepática é responsável pela meia-vida de mais de 22 horas, inibindo especificamente a absorção intestinal do colesterol dietético e biliar, sem interferir na absorção de vitaminas lipossolúveis (A, D, E, K), ácidos graxos ou sais biliares.

A redução do afluxo do colesterol a partir do intestino para o fígado resulta em aumento compensador da expressão de receptores hepáticos de LDL e aumento da captura das partículas de LDL circulantes. A redução final de LDL-c obtida com o uso de 10 mg/dia de ezetimiba (dose única preconizada), isoladamente ou em associação com a estatina, fica em torno de 15 a 25%. ^272^ Em uma meta-análise que incluiu cinco estudos clínicos randomizados (5.039 pacientes), em que a ezetimiba foi comparada a placebo em adição à estatina já em uso, a redução média de LDL-c foi de 23,6%, sem qualquer aumento nos efeitos adversos. ^273^ Essa potencialização do efeito hipolipemiante está demonstrada também na população de pacientes com HFHe. ^274^^,^^275^ Embora os dados sejam obviamente mais escassos na literatura, a eficácia hipolipemiante da ezetimiba está também evidenciada na população com HFHo. ^276^

O uso da associação estatina e ezetimiba já se mostrou eficaz na redução de desfechos substitutos ^277^ e de eventos isquêmicos analisados como desfecho secundário. ^278^ Entretanto, a eficácia da combinação na redução de eventos cardiovasculares maiores foi demonstrada no estudo IMPROVE-IT, ^279^ que comparou a associação ezetimiba/sinvastatina à sinvastatina isolada em pacientes estáveis após episódio de síndrome coronária aguda (SCA) e com LDL-c dentro das metas preconizadas. O desfecho primário foi composto por morte cardiovascular, síndrome coronariana aguda (IAM não fatal, angina instável necessitando internamento) e acidente vascular cerebral não fatal. Os pacientes que receberam ezetimiba associada à estatina apresentaram, após um ano, uma redução de LDL-c de 24% em comparação ao grupo de estatina isolada. Em um seguimento médio de 7 anos, a redução no risco relativo no desfecho cardiovascular primário, composto por morte cardiovascular, síndrome coronariana aguda (IAM não fatal, angina instável necessitando internamento) e acidente vascular cerebral não fatal foi de 6,4%. Esta redução de risco foi proporcional à redução obtida de LDL-c e comparável à redução de risco obtida para uma redução da mesma magnitude se obtida com o uso de estatina. ^280^ Portanto, a ezetimiba deve ser utilizada como terapia adjuvante às estatinas de alta intensidade quando estas, em sua dose máxima ou na máxima dose tolerada, não são suficientes para se atingir a meta de LDL-c (recomendação: classe I; nível de evidência: B).

##### 7.2.2.2. Inibidores de PCSK9

A PCSK9 é uma protease que regula a atividade do receptor de LDL, induzindo à sua degradação lisossomal. Dessa forma, ao reduzir a quantidade de LDLR na superfície do hepatócito e diminuir a atividade desses receptores, a PCSK9 aumenta a concentração plasmática de LDL-c. ^281^ A utilização de anticorpos monoclonais para bloquear a ligação da PCSK9 com o LDLR é a forma mais avançada de inibir a atividade dessa enzima. Esses anticorpos se ligam ao sítio alostérico do LDLR e bloqueiam a ligação PCSK9-LDLR. Consequentemente, aumentam a recirculação do receptor e reduzem os níveis séricos de LDL-c. Meta-análises já publicadas apontam para uma redução consistente de cerca de 50% dos níveis séricos do LDL-c em diversos cenários clínicos, isoladamente ou em combinação com máxima terapêutica tolerada. ^282^

Estão disponíveis no mercado nacional dois anticorpos, ambos na forma de solução injetável, em “canetas” prontas para injeção, que não permitem fracionamento da dose: evolocumabe, na apresentação de 140 mg, e alirocumabe, na apresentação de 75 e 150 mg. Ambos são utilizados de maneira semelhante, com injeção subcutânea a cada duas semanas (embora evolocumabe possa ser também usado na dose de 420 mg, 1 vez ao mês). Os dois fármacos foram testados no cenário da HFHe, em adição à máxima terapêutica tolerada com estatina com ou sem outros hipolipemiantes associados, com semelhante redução de LDL-c entre 50 e 60%. ^283^^-^^285^ Na HF, 300 pacientes (106 com HFHo, incluindo adolescentes entre 14 -18 anos de idade, na inclusão) receberam evolocumabe 420 mg a cada 4 semanas por um tempo (mediana) de 4,1 anos. A redução do LDL-c do basal à semana 12 foi de 21,2% (-59.8 mg/dL) em pacientes com HFHo e de 54,9% (-104.4 mg/dL) naqueles com HFHe grave. Esse resultado se manteve com o tempo. Dos 48 pacientes com HFHo que receberam dose titulada para 420 mg a cada duas semanas, a redução de LDL-c aumentou de -19,6% na semana 12 a -29,7%, 12 semanas após a dose de 420 mg a cada duas semanas. ^285^ Evolocumabe foi também testado em pacientes com HFHo na dose de 420 mg, via subcutânea (SC), 1 vez ao mês, com redução média de LDL-c de cerca de 21%. ^286^ Por essa razão, evolocumabe é também aprovado para uso na HFHo.

A redução de desfechos cardiovasculares com inibidores de PCSK9 foi demonstrada em populações de alto risco cardiovascular, com doença aterosclerótica clínica estabelecida. No estudo FOURIER, ^287^ foram avaliados 27.564 pacientes com DCV estabelecida (doença coronariana, cerebrovascular ou doença arterial periférica), que, apesar da máxima terapêutica tolerada, permaneciam fora da meta de LDL < 70 mg/dl. Após um tempo mediano de seguimento de 2,2 anos, o uso de evolocumabe esteve associado a uma redução de 15% no desfecho primário de morte cardiovascular, IAM, AVE, hospitalização por angina instável ou necessidade de procedimento de revascularização miocárdica. Já no estudo ODYSSEY Outcomes, ^288^ que envolveu 18.924 pacientes após síndrome coronariana aguda recente (1 a 12 meses antes da inclusão no estudo) com LDL ≥ 70 mg/dl apesar da máxima terapêutica tolerada com estatina associada ou não a outros hipolipemiantes, o uso de alirocumabe reduziu em 15% o desfecho primário composto de morte coronariana, IAM não fatal, AVE fatal ou não ou angina instável com necessidade de hospitalização. O tempo mediano de seguimento foi de 2,8 anos.

Portanto, considerando a eficácia hipolipemiante nos pacientes com HF e a demonstração de redução de desfechos cardiovasculares em populações de alto risco, o uso de inibidores de PCSK9 está indicado nos pacientes que, apesar da terapêutica com estatina de alta intensidade, ou na máxima dose tolerada, preferencialmente já associada a ezetimiba, permanecem fora da meta de LDL-c (recomendação: classe I; nível de evidência: A).

##### 7.2.2.3. Colestiramina

A colestiramina é uma resina que se liga aos ácidos biliares no intestino, formando um complexo insolúvel que é excretado nas fezes. Com o aumento da sua excreção, a síntese de ácidos biliares aumenta no hepatócito à custa de uma elevação na síntese do colesterol, mas principalmente por um aumento na expressão dos LDLR que retiram LDL da circulação, reduzindo seu nível plasmático. ^289^ O efeito hipolipemiante da colestiramina é variável, podendo chegar até cerca de 30% de redução de LDL-c na dose máxima. ^290^ A colestiramina é apresentada em envelopes de 4 g, e sua posologia inicial é de 4 g ao dia, podendo-se alcançar no máximo 24 g/dia, embora posologias superiores a 16 g sejam dificilmente toleradas.

Os principais efeitos colaterais relacionam-se ao aparelho digestivo (plenitude gástrica, náuseas), interferindo na motilidade intestinal e causando obstipação e meteorismo. O fármaco diminui a absorção de vitaminas lipossolúveis (A, D, K, E) e de ácido fólico, sendo eventualmente necessária a suplementação desses elementos. A colestiramina deve ser utilizada uma hora antes ou três horas após a ingestão de outros medicamentos, para não diminuir a absorção deles. A redução de desfechos cardiovasculares com colestiramina foi demonstrada na fase pré-estatina. Ela também reduziu a incidência de infarto do miocárdio em 19% em homens hipercolesterolêmicos em prevenção primária, em 7 anos de seguimento, no estudo Lipid Research Clinics. ^291^

A colestiramina pode ser utilizada como terapia adjuvante quando a terapêutica de alta intensidade com estatina, preferencialmente já associada a ezetimiba e/ou um inibidor de PCSK9, não é suficiente para se chegar à meta de LDL-c (recomendação: classe IIa; nível de evidência: B). O fármaco pode ser especialmente útil em crianças com menos de 8 anos que ainda não podem receber estatinas e em gestantes.

## 8. Terapias Alternativas para Tratamento da Hipercolesterolemia Familiar Bypass Ileal Parcial

A cirurgia de *bypass* ileal parcial começou a ser realizada para tratamento da hipercolesterolemia na década de 1960, proporcionando redução sustentada do LDL-c por mais de 20 anos. ^292^ A operação foi avaliada no ensaio clínico randomizado *Program on the Surgical Control of the Hyperlipidemias* (POSCH), envolvendo 838 pacientes sobreviventes de IAM, com LDL-c médio de 179 mg/dl. Em relação ao grupo controle, os participantes submetidos à operação apresentaram redução do LDL-c de 38% e diminuição do desfecho combinado de morte coronariana ou IAM não fatal de 35%. ^293^

Em um pequeno estudo com 11 portadores de HFHe, a cirurgia de *bypass* ileal parcial resultou em redução do LDL-c de aproximadamente 20% após 2 anos. ^294^

O principal efeito colateral dessa cirurgia é a diarreia (média de mais de três evacuações diárias após a operação). Litíase renal e biliar também são relatadas como eventos adversos. ^292^^,^^293^

Com a introdução das estatinas na prática clínica nas décadas de 1980/1990, e posteriormente de outros medicamentos hipolipemiantes, a cirurgia de *bypass* ileal parcial deixou de ser utilizada no tratamento da hipercolesterolemia. O papel desse procedimento no manejo da HF e na prevenção da DCV na vigência da terapia farmacológica atual é desconhecido.

Vale ressaltar que algumas técnicas contemporâneas de cirurgia bariátrica que resultam em redução expressiva do peso, como a cirurgia de *bypass* gástrico em Y de Roux e a derivação biliopancreática, também promovem redução do LDL-c. ^295^

### 8.1. Recomendação

Embora a cirurgia de *bypass* ileal tenha mostrado redução do LDL-c e de eventos cardiovasculares, não se recomenda de rotina esse procedimento em portadores de HF, dada a existência de várias outras modalidades de tratamento eficazes, não invasivas e de menor risco (recomendação: classe IIB; nível de evidência: B).

### 8.2. Plasmaférese e LDL-aférese

LDL-aférese (LA) e plasmaférese são duas opções de tratamento a partir de filtração sanguínea extracorpórea. Ambas envolvem sessões com duração de 2 a 3 horas, semanais ou quinzenais. A principal diferença entre os dois procedimentos é a especificidade. No procedimento de plasmaférese, ocorre separação das células do sangue e plasma do paciente, de modo que as sanguíneas são retidas e misturadas em um fluido de reposição para retornar ao paciente, enquanto o plasma juntamente com as proteínas (incluindo o HDL-C) é descartado. Os efeitos colaterais envolvem suscetibilidade a infecções, náusea, hipertensão, hipotensão e urticária. ^296^

Já na LA, o plasma não é descartado, mas passa por um filtro de precipitação para a remoção seletiva de colesterol de LDL, VLDL e Lp(a). Os efeitos colaterais mais frequentes incluem hipotensão, anemia, náusea, rubor e cefaleia, além de intercorrências com o acesso venoso. ^297^

Existem vários métodos de aférese, incluindo adsorção de celulose sulfato de dextrano, precipitação de LDL-c extracorpórea induzida por heparina, imunoadsorção e dupla aférese de plasma de lipoproteínas. Ao se compararem os métodos disponíveis em relação à sua capacidade de redução de lipídios, apenas pequenas diferenças foram observadas. Via de regra, esses métodos seletivos reduzem os valores de LDL-c do plasma em média de 50 a 70% após tratamento único. ^298^^,^^299^ O intervalo de tempo para retorno ao nível basal de LDL-c varia entre quatro dias e três a quatro semanas.

 Pela especificidade, a LA é mais bem tolerada com menor taxa de efeitos colaterais comparada à plasmaferése (2% *versus* 12%, respectivamente) e mais eficaz em reduzir o LDL-c (60 a 65% *versus* 50%, respectivamente); porém, com menor disponibilidade e custo duas vezes maior. ^300^

### 8.3. LDL-aférese em Crianças Homozigóticas ou Heterozigóticas Compostas

Os critérios recomendados para indicação de LA ou plasmaferese após dieta associada à farmacoterapia otimizada, em pacientes pediátricos homozigóticos e heterozigóticos compostos, incluem:

- Redução menor do que 50% em relação ao LDL-c basal ou valores de LDL-c mantidos acima de 360 mg/dl: esses critérios são modificáveis de acordo com o contexto clínico de cada paciente, que envolve, dentre outros aspectos, a progressão da doença aterosclerótica. Estudos retrospectivos e de segmento longitudinais demonstram que a terapia com LA em crianças resultou em redução e/ou desaparecimento dos xantomas cutâneos, retardo da evolução de estenose valvar aórtica e supra-aórtica, e regressão de lesão coronariana. ^301^^-^^305^ A dieta e farmacoterapia devem ser continuadas com os procedimentos de aférese, já que as estatinas, quando associadas, reduzem o LDL-c em até 70% ou mais. As estatinas também retardam o efeito rebote de elevação dos níveis de LDL-c pós-aférese.
- Idade de início da LA: o prognóstico cardiovascular depende do início do tratamento; quanto mais precoce, melhor. A idade para início da LA é a partir de 5 anos, preferivelmente antes dos 8 anos de idade. Em casos mais graves, pode-se iniciar em idades mais precoces. ^303^ A partir de 10 anos, a LA não se mostrou tão benéfica nos estudos de seguimento e retrospectivos. ^303^
- Monitoramento da progressão da aterosclerose: a presença de aterosclerose progressiva é um dos critérios para escolha de tratamentos invasivos. Exames de imagem como ecocardiograma transtorácico para avaliação da presença e/ou progressão da doença valvar aórtica e arco aórtico, ultrassom de carótidas para mensurar o espessamento médio-intimal e de placas de ateroma, teste ergométrico devem ser realizados no início do tratamento e a cada 2 anos durante o seguimento. ^303^^,^^304^
- Contra-indicações para aférese: diátases hemorrágicas, resistência à adequada coagulação e hipersensibilidade à heparina.
- Efeitos colaterais: a anemia ferropriva é o efeito colateral mais frequente, hipotensão e dificuldades de acesso venoso também são reportados com menor frequência.
- Segurança: é um procedimento seguro e tolerável para crianças e adolescentes, em centros especializados. ^306^
  Numerosos estudos de casos e avaliações clínicas demonstraram que crianças submetidas à LA por muitos anos (até 20 anos) se desenvolveram normalmente. ^306^^-^^308^

### 8.4. LDL-aférese em Pacientes Adultos com HFHo ou HFHe Graves

Nos pacientes com HFHo ou HFHe graves, após 6 meses sem resposta adequada ^309^ à máxima terapêutica medicamentosa, que inclui estatinas, ezetimiba e inibidores do PCSK9, ou intolerância medicamentosa, a LA é indicada nas seguintes situações:

- HFHo funcionais com colesterol LDL-c ≥ 300 mg/dl (ou colesterol não HDL ≥ 330 mg/dl).
- HFHe funcionais com colesterol LDL-c ≥ 300 mg/dl (ou colesterol não HDL ≥ 330 mg/dl) e um ou nenhum fator de risco.
- HFHe funcionais com colesterol LDL-c ≥ 200 mg/dl (ou colesterol não HDL ≥ 230 mg/dl) e dois ou mais fatores de risco ou Lp (a) alta, ≥ 50 mg/dl.
- HFHe funcionais com colesterol LDL-c ≥ 160 mg/dl (ou não HDL colesterol ≥ 190 mg/dl) e presença de DCV clínica ou subclínica ou fatores de risco como diabetes e tabagismo).

Vários ensaios clínicos confirmaram os benefícios de LA na prevenção e redução da progressão da doença aterosclerótica. ^310^^-^^317^ Outros efeitos com comprovado benefício também são relatados, como melhora da função endotelial, ^318^ vasodilatação coronariana, ^319^ melhora no fluxo microvascular ^320^ e perfusão miocárdica. ^321^ A LA é o único tratamento que reduz consistentemente os níveis de Lp (a) em mais de 50%. ^322^^,^^323^

Apesar de a LA ser o tratamento mais viável para certos pacientes homozigóticos e heterozigóticos graves, o procedimento está disponível em poucos centros no mundo, em função do alto custo.

### 8.5. Recomendação para a Indicação de LDL-aférese em Adultos com HFHo ou HFHe Graves e Crianças com HFHo

Estudos retrospectivos ou coortes de segmento a longo prazo demonstram que pacientes com HFHo ou HFHe graves dificilmente alcançam as metas preconizadas de LDL-c, apesar das doses máximas da terapêutica otimizada associadas a sessões semanais ou quinzenais de LA. Estudos demonstram que, apesar das dificuldade na redução do colesterol, pacientes que apresentaram valores mais baixos de LDL-c tiveram melhor sobrevida e menos eventos cardiovasculares do que aqueles com níveis mais elevados. Esse benefício foi independente do tipo de tratamento realizado. Portanto, a extensão da redução lipídica, bem como seu início precoce, determinaram os resultados clínicos de modo independente. ^324^ Sendo a LA o procedimento que mais reduz os valores de LDL-c, ela é recomendada para adultos e crianças (recomendação: classe I; nível de evidência: C).

### 8.6. Transplante Hepático

O transplante hepático pode ser uma alternativa para pacientes HF refratários ao tratamento farmacológico ^325^ e está indicado especialmente para os portadores de HFHo. Entretanto, deve-se sempre discutir com pacientes e familiares para que sejam esclarecidos os riscos e os benefícios do procedimento (recomendação: classe IIB; nível de evidência: C).

## 9. Algoritmos de tratamento na Hipercolesterolemia Familiar em Adultos

**Figura 7:**
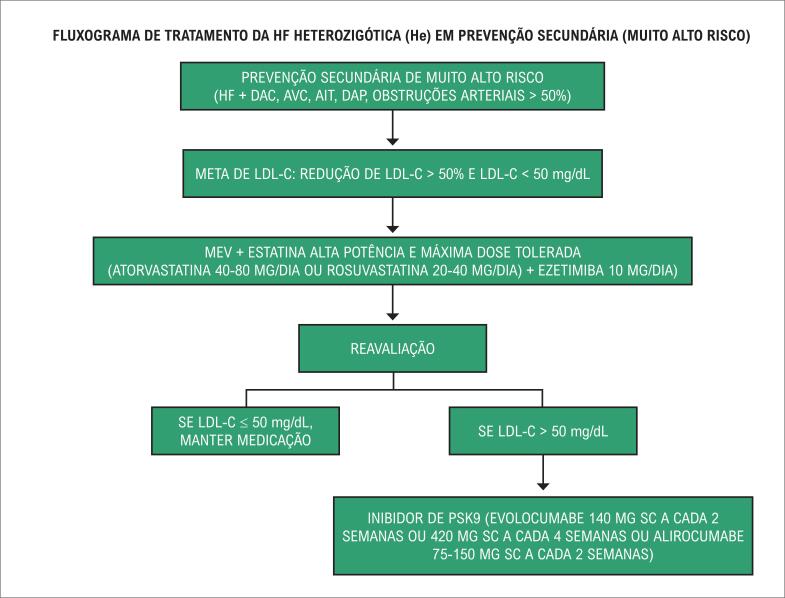
Fluxograma de tratamento da HF heterozigótica (He) em prevenção secundária (muito alto risco). AIT: ataque isquêmico transitório; AVC: acidente vascular cerebral; DAC: doença arterial coronariana; DAP: doença arterial periférica; HF: hipercolesterolemia familiar; LDL-C: colesterol da lipoproteína de baixa densidade; MEV: mudança de estilo de vida.

Visando facilitar a abordagem terapêutica da hipercolesterolemia familiar no adulto (> 18 anos) tanto na forma heterozigótica, como na homozigótica e, considerando o risco cardiovascular, cinco algoritmos de tratamento são apresentados nas Figuras 7 a 11 .

A Figura 7 mostra o fluxograma de tratamento na hipercolesterolemia familiar heterozigótica de muito alto risco, as metas e a sequência terapêutica proposta para o alcance dessas metas. Estatinas potentes, ezetimiba e inibidores de PCSK9 visando a redução de LDL-c > 50% e o alcance de LDL-c < 50 mg/dL são recomendados para o alcance de metas de forma escalonada.

**Figura 8:**
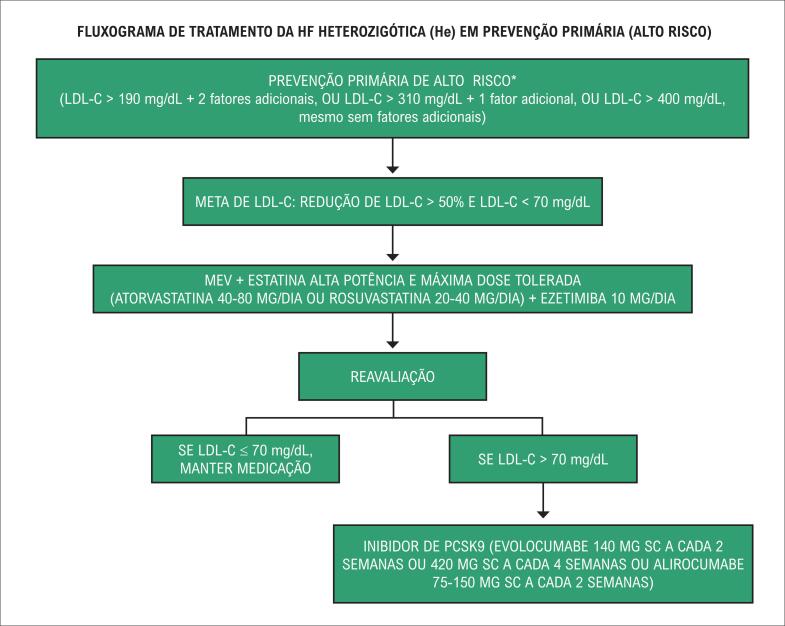
Fluxograma de tratamento da HF heterozigótica (He) em prevenção primária (alto risco). HF: hipercolesterolemia familiar; LDL-C: colesterol da lipoproteína de baixa densidade; SC: subcutâneo. *São considerados fatores de risco adicionais na HF: ^56^ idade > 40 anos e sem tratamento, tabagismo, sexo masculino, Lp(a) > 50 mg/dL, HDL-c < 40 mg/ dL, hipertensão arterial, diabetes melito, história familiar de DAC prematura em parentes de 1º grau (homens < 55 anos e mulheres < 60 anos), doença renal crônica (TFG < 60 ml/min) e IMC > 30 kg/m^2^.

Na Figura 8 observamos o fluxograma de tratamento na hipercolesterolemia familiar heterozigótica de alto risco, as metas propostas e o fluxo de tratamento para o alcance das metas. Estatinas potentes, ezetimiba e inibidores de PCSK9 visando a redução de LDL-c > 50% e o alcance de LDL-c < 70 mg/dL são recomendados para o alcance de metas de forma escalonada.

**Figura 9:**
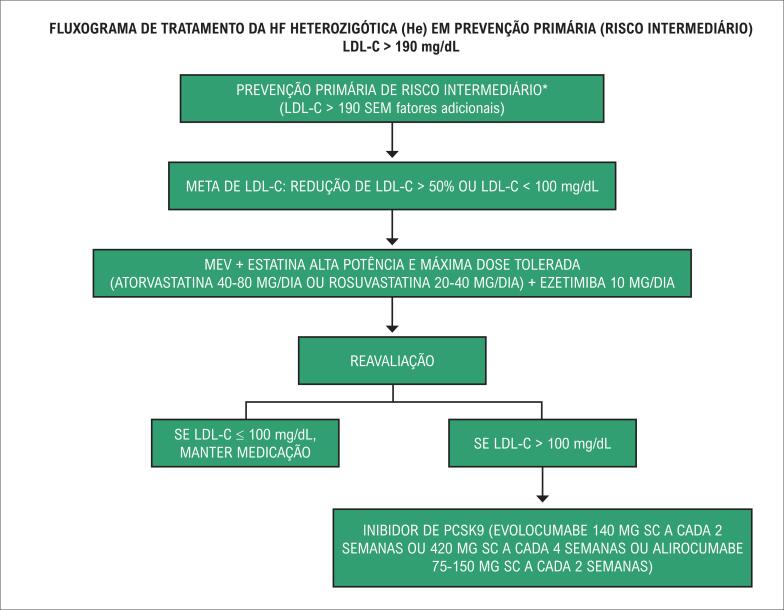
Fluxograma de tratamento da HF heterozigótica (He) em prevenção primária (risco intermediário). HF: hipercolesterolemia familiar; LDL-C: colesterol da lipoproteína de baixa densidade; SC: subcutâneo. *São considerados fatores de risco adicionais na HF: ^56^ idade > 40 anos e sem tratamento, tabagismo, sexo masculino, Lp(a) > 50 mg/dL, HDL-c < 40 mg/ dL, hipertensão arterial, diabetes melito, história familiar de DAC prematura em parentes de 1º grau (homens < 55 anos e mulheres < 60 anos), doença renal crônica (TFG < 60 ml/min) e IMC > 30 kg/m^2^.

Na Figura 9 é apresentado o fluxograma de tratamento da hipercolesterolemia familiar heterozigótica de risco intermediário, as metas propostas e o fluxo de tratamento para o alcance das metas. Estatinas potentes, ezetimiba e inibidores de PCSK9, visando a redução > 50% no LDL-c e o alcance de LDL-c < 100 mg/dL são recomendados para o alcance de metas de forma escalonada.

**Figura 10:**
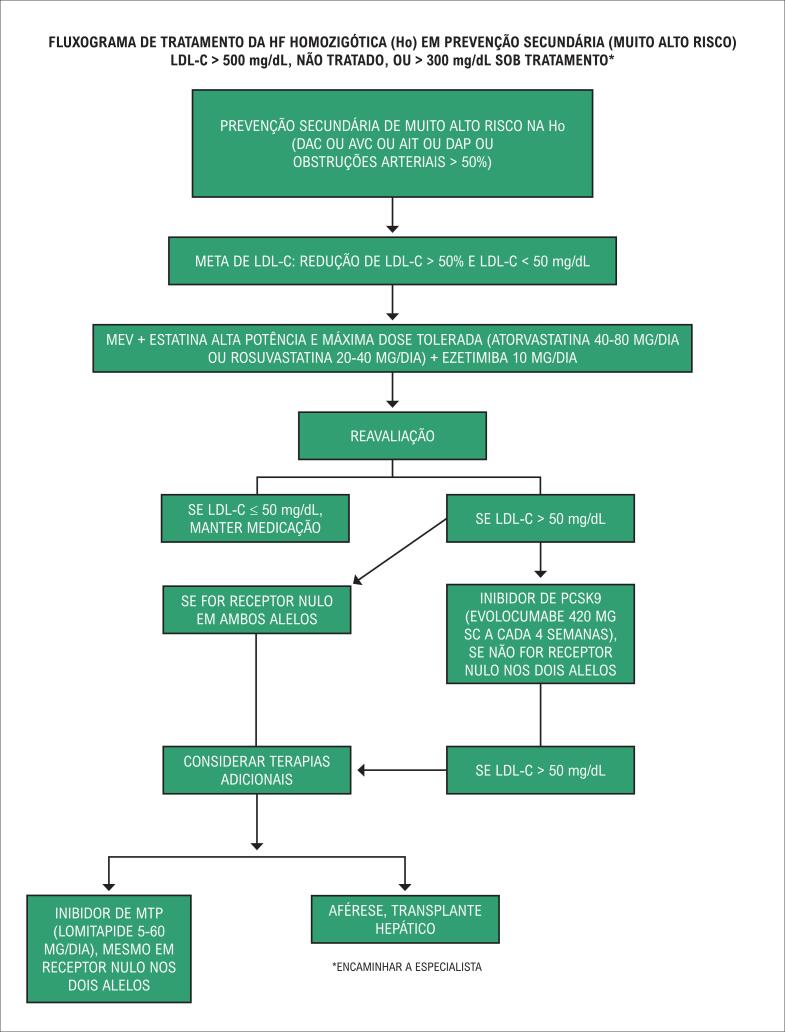
Fluxograma de tratamento da HF homozigótica (Ho) em prevenção secundária. AIT: ataque isquêmico transitório; AVC: acidente vascular cerebral; DAC: doença arterial coronariana; DAP: doença arterial periférica; HF: hipercolesterolemia familiar; LDL-C: colesterol da lipoproteína de baixa densidade; MEV: mudança de estilo de vida.

Na Figura 10 vê-se o fluxograma de tratamento na hipercolesterolemia familiar homozigótica em prevenção secundária, as metas terapêuticas e a sequência terapêutica proposta para o alcance dessas metas. O alvo é alcançar redução > 50% no LDL-c e meta de LDL-c < 50 mg/dL. Estatinas potentes, ezetimiba são recomendados como primeira linha, além de inibidores de PCSK9, se para o alcance de metas uma redução de LDL-c < 30% for suficiente e não for homozigoto para receptor de LDL nulo; se o LDL-c permanecer > 50 mg/dL ou se o paciente for homozigoto para receptor de LDL nulo, terapias adicionais como o inibidor de MTP (lomitapide), podem permitir alcance de metas com redução adicional de LDL-c de 50%. Outras terapias adicionais, como aférese e transplante hepático são incluídas no fluxograma e estes pacientes devem sempre ser encaminhados e acompanhados por especialista.

**Figura 11:**
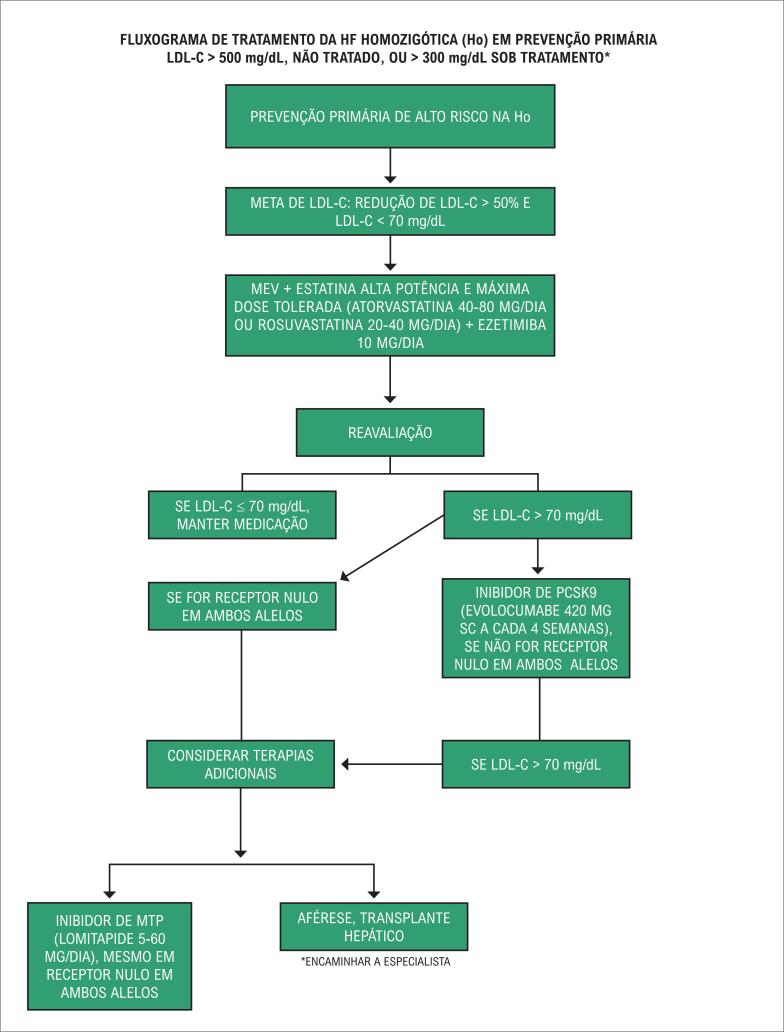
Fluxograma de tratamento da HF homozigótica (Ho) em prevenção primária. AIT: ataque isquêmico transitório; AVC: acidente vascular cerebral; DAC: doença arterial coronariana; DAP: doença arterial periférica; HF: hipercolesterolemia familiar; LDL-C: colesterol da lipoproteína de baixa densidade; MEV: mudança de estilo de vida; MTP: proteína de transferência de triglicerídeos microssomal.

Na Figura 11 vê-se o fluxograma de tratamento na hipercolesterolemia familiar homozigótica em prevenção primária, as metas terapêuticas e a sequência terapêutica proposta para o alcance dessas metas. O alvo é alcançar redução > 50% no LDL-c e meta de LDL-c < 70 mg/dL. Estatinas potentes, ezetimiba são recomendados como primeira linha, além de inibidores de PCSK9, se para o alcance de metas uma redução de LDL-c < 30% for suficiente e não for homozigoto para receptor de LDL nulo; se o LDL-c permanecer > 70 mg/dL ou se o paciente for homozigoto para receptor de LDL nulo, terapias adicionais como o inibidor de MTP (lomitapide), podem permitir alcance de metas com redução adicional de LDL-c de 50%. Outras terapias adicionais, como aférese e transplante hepático são incluídas no fluxograma e estes pacientes devem sempre ser encaminhados e acompanhados por especialista.

## 10. Hipercolesterolemia Familiarna Criança

### 10.1. Triagem

A suspeição da criança ou do adolescente para HFéde extrema importância; afinal, estima-se que no mundo, a cada minuto, nasça uma criança com HF. No entanto, o diagnóstico nesse grupoéum desafio na prática clínica. ^59^

Diversos fatores contribuem para o subdiagnóstico dessa condição. Ressalta-se o desconhecimento quantoàs recomendações da idade ideal para o rastreio laboratorial e uma tendência clínica a avaliar o perfil lipídico em crianças com sobrepeso/obesidade ou diabetes melito (crianças com HF são, em sua maioria, eutróficas e, em geral, não são portadoras de diabetes). Além disso, háfalhas na obtenção de uma anamnese detalhada desde a primeira consulta de puericultura, em busca de história de DCV prematura ou de níveis elevados de colesterol, sabidamente conhecidos, em parentes de primeiro grau.

Desse modo, as crianças assintomáticas com HFHe são geralmente identificadaspor meio do perfil lipídico sérico, que é realizadona presença de um parente próximo com diagnóstico confirmado de HF. ^57^ Nesse contexto, exceto em condições específicas na qual um rastreio precoce estárecomendado, a triagem laboratorial de crianças e adolescentes para dislipidemia, incluindo a HF,érecomendada de maneira universal a partir dos 10 anos de idade (recomendação: classe I; nível de evidência: A).

A ferramenta laboratorial inicial de rastreio neste grupoéo perfil lipídico, a partir da análise dos valores de colesterol total e do LDL-c. No entanto,éimportante ressaltar:

1. No rastreio universal, operfil lipídico pode ser realizado sem a necessidade de jejum, uma vez que o estado alimentar não influencia as concentrações de colesterol total, LDL-c e não-HDL-c.
2. Uma medida isolada de LDL-c não ésuficiente para o diagnóstico de dislipidemia em criança ou adolescente. Estudos demonstram ampla variação do LDL quando de sua repetição no período mínimo de 2 semanas. ^326^

### 10.2. Valores do Perfil Lipídico para a Suspeição da Hipercolesterolemia Familiar

**Figura 12:**
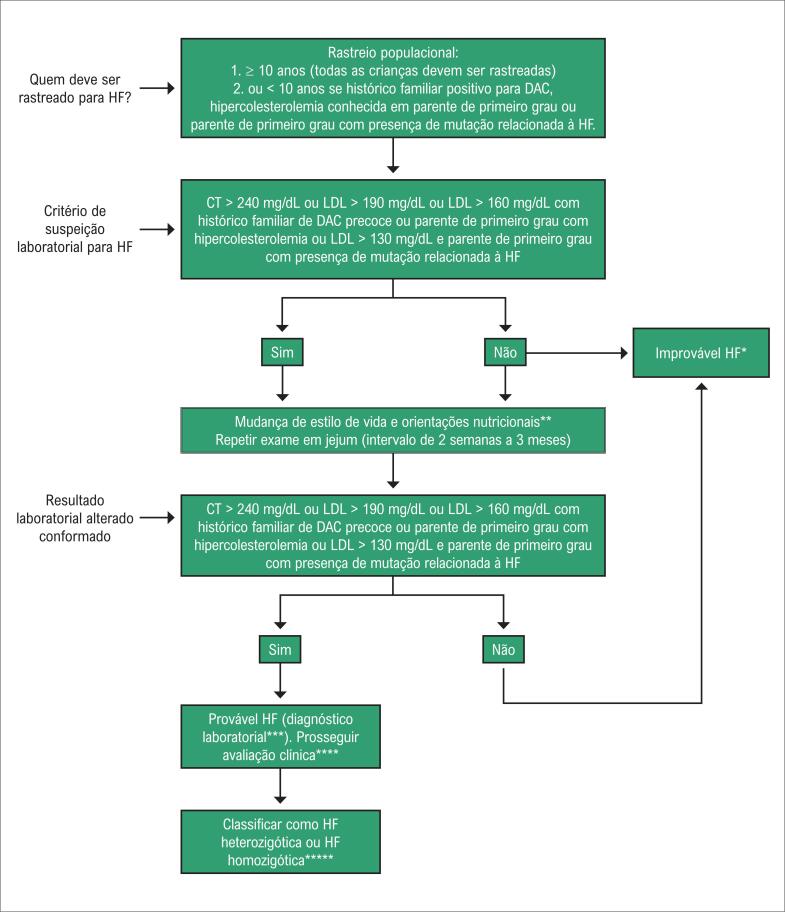
Algoritmo de triagem laboratorial de hipercolesterolemia familiar em crianças e adolescentes. Adaptado de Expert panel on integrated guidelines for cardiovascular health and risk reduction in children and adolescents; ^224^ Wiegman A, et al. ^327^ DAC: Doença arterial coronariana; HF: hipercolesterolemia familiar; CT: Colesterol total; LDL: Low-density lipoprotein. *Improvável HF: a ausência de critérios laboratoriais para HF não significa que outra dislipidemia não esteja presente. A criança ou adolescente com níveis de perfil lipídico fora do valor de referência para sua faixa etária deverá seguir com avaliação clínica. Importante avaliar causas secundárias nesta faixa etária: disfunção renal, tireoidiana, HIV, doenças auto-imunes, diabetes e obesidade, dentre outras. **Mudança de estilo de vida e orientação nutricional; vide seção terapia não farmacológica. ***Quando disponível o teste genético deve ser ofertado. ****Avaliação clínica: sinais clínicos como xantomas, xantelasma, arco corneano e espessamento de tendões devem ser avaliados. Descartar condições clínicas não HF que cursam com hipercolesterolemia. Escores de Dutch devem ser aplicados nesta etapa. *****Vide seção: Particularidades no manejo do paciente com HFo

1. O algoritmo da triagem laboratorial de crianças ou adolescentes com HF está descrito na Figura 12 (recomendação: classe I; nível de evidência: A).
2. Ressalta-se que, na vigência de níveis de LDL-c > 400 mg/dl, HFHe grave ou HFHo devem ser suspeitadas ^59^ (recomendação: classe I; nível de evidência: A).

### 10.3. Fatores Relacionados ao Aumento do Risco Cardiovascular

Em geral, pacientes com HF são considerados de alto risco cardiovascular, que pode seelevar dependendo da presença de outros fatores. ^139^^,^^328^^,^^329^ Dentre estes, destacam-se: níveis reduzidos de HDL-c, níveis elevados de triglicerídeos, níveis elevados de Lp (a) e fatores de estilo de vida, incluindo tabagismo, dietaaterogênicae sedentarismo. ^56^

Ainda nesse grupo, devem ser lembradas condições específicas relacionadas com desenvolvimento de DAC prematura, dentre eslas: diabetes melito tipos 1 e 2, doença renal crônica, transplantados cardíacos, doença de Kawasaki, doença inflamatória crônica,paciente vivendo com vírus da imunodeficiência humana (HIV), síndrome nefrótica e crianças que foram submetidas a tratamento para neoplasias. ^224^

#### 10.3.1. Triagem para o Diagnóstico de Hipercolesterolemia Familiar Além do Perfil Lipídico Laboratorial

Dada a gravidade dos pacientes e a precocidade das lesões cardíacas, há necessidade de monitoramento das complicações, idealmente a partir do diagnóstico, o que normalmente acontece após os 2 anos de idade. Consiste na determinação de marcadores da aceleração da progressão da aterosclerose, da função cardíaca e de lesões valvares e da raiz aórtica.

Em relação ao monitoramento da aterosclerose, deve-se determinar a estrutura e a função arterial periodicamente, o que pode ser utilizado na tomada de decisões do manejo medicamentoso e das metas de LDL-c. A espessura médio-intimal tem sido considerada a forma ideal e de mais fácil realização de avaliação estrutural da parede vascular em crianças portadoras de HF, especialmente nas com mais de 8 anos, utilizando-se os critérios validados internacionalmente para a idade ^330^ (recomendação: classe IIa; nivel de evidencia: B). Recentemente, tem-se demonstrado que a tomografia axial computadorizada pode ser ainda mais sensível, mas seu uso parece ser reservado para os pacientes com HFHo ^144^ (recomendação: classe IIa; nivel de evidência: C).

Existem várias formas de avaliação de função endotelial descritas para esses pacientes, entre as quais: *distensibilidade mediada por fluxo (por tonometria digital), hiperemia reativa pós-oclusivae velocidade de onda de pulso* . Ainda não há consenso de qual a forma de avaliação mais sensível e específica, sendo então sugerida a utilização do método que for mais validado localmente ^331^^,^^332^ (recomendação: classe IIa; nível de evidencia: C).

O *strain global* do ventrículo esquerdo parece estar diminuído em crianças com dislipidemias graves; por isso, deve-se avaliar periodicamente as funções ventriculares destes pacientes, utilizando-se inicialmente a ecocardiografia convencional. Também é importante realizar a avaliação das valvas cardíacas e da raiz aórtica, no intuito de afastar possibilidade de disfunções, dilatações ou calcificações. O método que tem se mostrado mais sensível para isso é também a tomografia axial computadorizada ^333^ (recomendação: classe IIa; nível de evidência: C).

### 10.4. Tratamento

**Figura 13:**
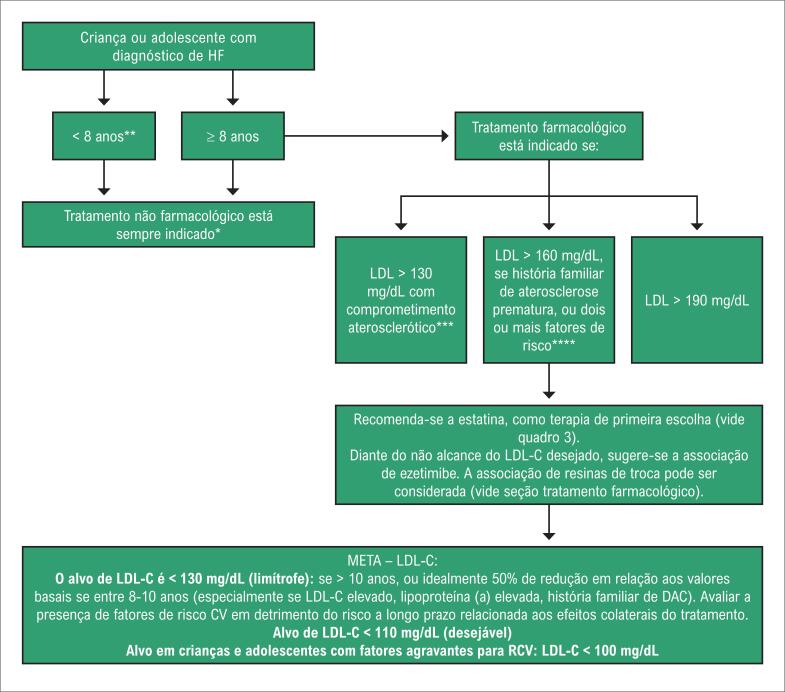
Algoritmo do Manejo da hipercolesterolemia familiar em crianças e adolescentes. Adaptado de Expert panel on integrated guidelines for cardiovascular health and risk reduction in children and adolescents; ^224^ Wiegman A, et al. ^327^ HF: hipercolesterolemia familiar; LDL: Low-density lipoprotein; DAC: Doença Arterial Coronariana; RCV: Risco Cardiovascular. *Todas as crianças devem ser submetidas a terapia não farmacológica: Vide seção terapia não farmacológica. **Em crianças menores de 8 anos a decisão por terapia farmacológica deverá ser avaliada de forma individualizada, em geral, para pacientes heterozigotos graves ou homozigotos. ***Se LDL > 130 mg/dL, sem comprometimento aterosclerótico, optar por tratamento não-farmacológico. ****Vide seção fatores relacionados ao aumento do RCV.

A indicação, o manejo e as metas a serem alcançadas com a instituição dos tratamentos não farmacológico e farmacológico estão descritos na Figura 13 (recomendação: classe II; nível de evidência: A).

#### 10.4.1. Tratamento Não Farmacológico

Mesmo em crianças com HF, a modificação do estilo de vida é parte fundamental do tratamento. O manejo da dislipidemia deve ser, preferencialmente, com o apoio de uma *equipe interdisciplinar* . Deve-se associar o combate ao fumo, primário ou passivo, além do controle intensivo de hipertensão arterial e sobrepeso ou obesidade em todos os pacientes, logo a partir do diagnóstico.

##### 10.4.1.1. Dieta

Sabe-se que, na HF, a dieta tem efeito parcial, pelas suas características fisiopatológicas. Apesar disso, recomenda-se a dieta a partir do diagnóstico. Isso porque há um efeito ainda significativo sobre as lipoproteínas e apolipoproteínas, além de haver evidência de diminuição do padrão inflamatório desses pacientes, tanto menor quanto maior a ingestão de ácidos graxos poli-instaurados, e maior quanto maior a ingestão de calorias e colesterol. ^334^^,^^335^

Como em todas as crianças da população geral, deve-se diminuir a ingestão de ácidos graxos saturados e aumentar a ingestão de ácidos graxos mono e poli-insaturados, que devem ser majoritariamente de origem vegetal, sendo o óleo de canola o de melhor resultado. Deve-se também eliminar os ácidos graxos *Trans* , aumentar a ingestão de frutas e verduras (estimulando ao máximo o consumo de fibras, mas preferencialmente as dos alimentos) e restringir a ingestão de açúcar adicionado ^334^^,^^335^ (recomendação: classe IIa; nível de evidência: B).

Mesmo na criança, a forma de tratamento dietético inicial segue as orientações denominadas dieta tipo I, sendo calculadas a partir do volume calórico total segundo volume de calorias, macro e micronutrientes para proporcionar um adequado crescimento e desenvolvimento ^334^^,^^335^ (recomendação: classe I; nível de evidência: B).

Caso não haja a resposta adequada à dieta tipo I, institui-se a dieta tipo II, recomendando-se fortemente o manejo por um especialista em nutrição (nutricionista ou nutrólogo), pois o risco de desnutrição, de macro ou micronutrientes (principalmente de vitaminas lipossolúveis), é muito grande, sendo os pacientes menores e em uso de ezetimiba ou resinas de troca os de risco máximo ^336^ (recomendação: classe I; nível de evidência: B).

##### 10.4.1.2 Atividade Física

Para crianças com idade entre6 e 17 anos, recomendam-se 60 minutos ou mais por dia de atividade aeróbica, de grauintenso a vigoroso. Sãorecomendadas também atividadesde força muscular e *muscle-strengthening and bone-loading* pelo menos três vezes por semana (recomendação: classe IIa; nível de evidência: B). ^337^^,^^338^

As crianças pré-escolares devem permanecer ativas ao longo do dia, para estimular o crescimento, o desenvolvimento e adquirir um repertório de capacidades motoras.Deve-se atingir um total de pelo menos 3 horas ativas por dia (recomendação: IIa; evidência: B).

Mesmo não havendo consenso sobre a quantidade de atividade física necessária para o controle do impacto da dislipidemia em crianças, sabe-se que o comportamento ativo melhoraa função endotelial edetermina regressão de espessamento médio-intimal ^339^ (recomendação: IIa; evidência: B).

Pacientes com dislipidemia, especialmente com HF, podem necessitar de um programa de reabilitação cardiopulmonar e metabólico de modo integrado e interdisciplinar. Mesmo na infância, por vezes é mais seguro instituir inicialmente uma atividade física supervisionada, mesmo porque se sabe que esse tipo de abordagem pode determinar melhor controle dos fatores de risco cardiovasculares e regressão da aterosclerose subclínica ^340^ (recomendação: IIa; evidência: A).

#### 10.4.2. Tratamento Farmacológico

Recomenda-se que, seguindo rigorosamente os crité rios descritos adiante e apó s mudança de estilo de vida, a terapia hipolipemiante seja iniciada apó s os 8 anos de idade. O tratamento em menores de8 anos pode ser indicado em casos graves e apó s avaliação individualizada. Tem como objetivo a redução de pelo menos 50% no LDL-c e, se possível, o alcance da meta de valores < 110 mg/dl de LDL-c (desej á vel), ou no m í nimo de 130 mg/dl (lim í trofe), além da redução de xantomatose e prevenção do aparecimento de DAC (recomendação: I; evidência: A).

##### 10.4.2.1. Estatinas

O uso de estatinas diminui significativamente colesterol total, LDL-c e Apo B, sem aparente ocorrê ncia significativa de efeitos adversos, relacionados a desenvolvimento sexual, toxicidade muscular ou hep á tica. O fármaco pode ser utilizado a partir dos8 anos de idade (em casos individualizados, pode ser prescrito em menores de8 anos). ^59^^,^^341^^,^^342^ As estatinas podem diminuir o LDL-c em cerca de 30% e aumentar o HDL-c em 5% ^343^^-^^348^ (recomendação: IIa; evidência: A).

Recentes publicações evidenciaram que as estatinas, alé m da redução de colesterol total e LDL-c nessa faixa et á ria, promoveram melhora da funçã o endotelial, diminui ção da espessura íntima-mé dia da caró tida e regressão de xantomas (recomendaçã o: IIa; evidê ncia: B). ^349^ As doses usualmente utilizadas dos hipolipemiantes em crianças e adolescentes são descritas no Quadro 3 .

**Quadro 3:** Doses de hipolipemiantes utilizados em crianças e adolescentes

Fármaco	Doses (mg)
Lovastatina	10 a 40
Pravastatina	10 a 40
Sinvastatina	10 a 40
Rosuvastatina	5 a 40
Atorvastatina	10 a 40
Colestiramina *	4 a 16
Ezetimiba	10

* Em gramas.

Doses maiores que as descritas podem ser utilizadas apó s aná lise individual do risco em crianças/adolescentes. Nesse grupo, sugere-se a utilização inicial da menor dose poss í vel de estatinas (recomendação: IIa; evidência: C). Nas situações em que a meta de LDL-c não for atingida com a dose m áxima tolerada de estatina, o uso de ezetimiba est á indicado como segunda escolha associada ao tratamento (recomendação: II; evidência: C).

##### 10.4.2.2. Inibidores da Absorção do Colesterol

O uso do ezetimiba como monoterapia diminui em cerca de 28% os valores de LDL-c em crianças com HFHe. Recomenda-se seu uso como monoterapia a partir dos 5 anos e, em associação com estatina, em crianças acima de 8 anos ^342^^,^^350^ (recomendação: IIb; evidência: C).

##### 10.4.2.3. Sequestrantes dos Ácidos Biliares

Os sequestrantes dos á cidos biliares podem ser utilizados em qualquer idade. Diminuem em mé dia, como monoterapia, cerca de 10 a 15% dos n í veis de LDL-c. Podem ser també m associados com estatinas ou ezetimiba , mas em hor á rios de administração diferentes. Pelo risco de desnutrição relacionadoà s vitaminas lipossol ú veis, recomenda-se monitoramento nutricional e suplementação segundo crité rios objetivos de deficiencia. ^351^ Para aumentar a tolerância das crianças, sugere-se associar suco de maçã ao sequestrante, para melhorar a palatabilidade (recomendação: I; evidência: B).

##### 10.4.2.4 Suplementos

Suplementa ção de 1,2 a 1,5 g de fitosteró is pode diminuir os n í veis de colesterol total e LDL-c em crianças portadoras de HFHe em cerca de 10% ^352^ (recomendação: IIb; evidência: B). É importante lembrar que suplementos à base de ômega 3, como os encontrados em derivados do óleo de peixe, não são recomendados para crianças com HF, pois podem aumentar os níveis de LDL-c ^329^ (recomendação: III; evidência: B).

#### 10.4.3. Novos Tratamentos

Neste grupo encontram-se medicamentos como lomitapide, mipomersen e inibidores da PCSK9 (alirocumabe e evolucumabe). O evolocumabe na dose de 420 mg SC a cada 4 semanas foi estudado na população pediátrica com HFHe (n=170) de 10 a 17 anos, não controlados com o tratamento padrão que incluía estatina e/ou ezetimiba. Foram 104 pacientes randomizados para evolocumabe e 53 para placebo. O evolocumabe reduziu o LDL-c na semana 24 em 44,5%, com uma redução absoluta de 77,5 mg/dL. Houve redução das demais variáveis lipídicas. ^353^ Já o alirocumabe foi estudado no Odissey kids para definição de dose, ^354^ porém os dados do estudo ainda não foram finalizados.

Apesar da aprovação de alguns desses fármacos para uso na população adulta, na população pediátrica o uso desses agentes pode ser considerado caso a caso ^59^ (recomendação: IIa; evidência: C). No entanto, até o momento, os dados de segurança de longo prazo disponíveis e publicados provêm de estudos em adultos (18 anos ou mais), sendo ainda escassos dados em crianças com HF. ^355^^-^^357^

#### 10.4.4. Particularidades no Manejo da Hipercolesterolemia Familiar Homozigótica

**Figura 14:**
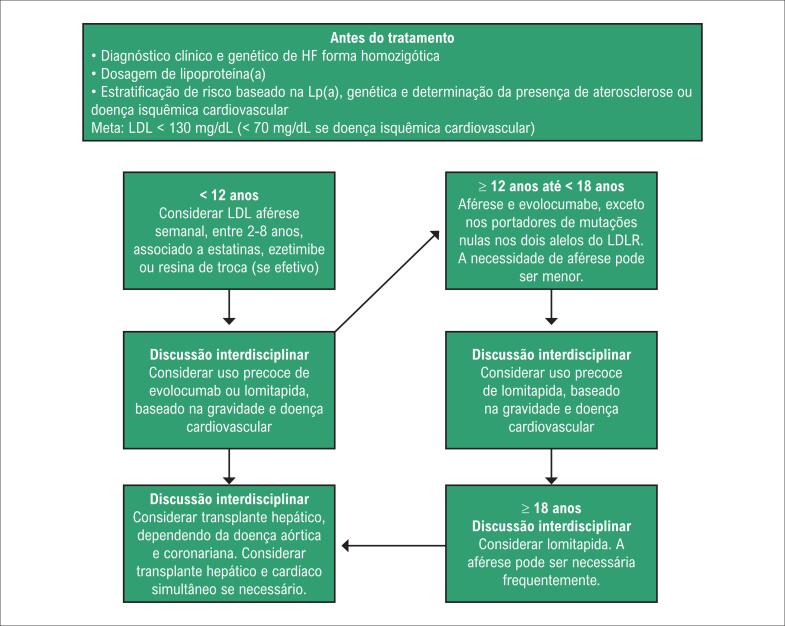
Algoritmo de tratamento de crianças e adolescentes com hipercolesterolemia familiar homozigótica. ^358^ HF: hipercolesterolemia familiar.

O grupo de maior desafio no manejo e, ao mesmo tempo, de maior impacto no tratamento desde a infância é o composto por crianças e adolescentes com HFHo. Nestes, por serem homozigotos para o gene do LDLR ou por defeitos genéticos associados, os quais fenotipicamente têm concentrações de LDL-c bastante elevadas, há necessidade de intervenção a partir do momento do diagnóstico, independentemente da idade, para que se evitem manifestações ateroscleróticas, às vezes na primeira década. Pelas características fisiopatológicas, o uso de resinas de troca frequentemente não se mostra efetivo, em especial nos pacientes homozigóticos para mutações que levam a atividade nula no LDLR. ^351^ A Figura 14 representa o algoritmo utilizado para o manejo de tais crianças e adolescentes ^358^ (recomendação: IIa; evidência: C). Esses pacientes devem ser encaminhados a especialista em lípides.

Em adolescentes graves com aterosclerose clinicamente manifesta, h á indicação de revasculariza ção do mioc árdio. ^359^^,^^360^ No caso de doenç a aó rtica decorrente de dislipidemia grave, a troca por homoenxerto pulmonar (cirurgia de Ross-Konno) ^361^ pode ser uma opção interessante para o adolescente, por sua durabilidade (recomendação: IIb; evidência: C).

### 10.5. Monitora mento do Tratamento

Recomenda-se que os fármacos isolados ou associados devem ser usados de forma contínua ^362^ (recomendação: I; evidência: C). Uma ampla revisão sistemática (incluiu apenas ensaios clínicos randomizados controlados), que avaliou o uso de estatinas em crianças e adolescentes, demonstrou que o risco de eventos adversos neste grupo foi semelhante aos observados em adultos tratados com estatina, pelo menos a curto prazo. Os estudos avaliados analisaram o efeito da terapia com estatina quanto ao impacto no desenvolvimento sexual, crescimento, nutrição, toxicidade hepática ou renal. Para a maioria desses parâmetros, não houve diferença estatisticamente significativa entre os grupos de tratamento e placebo. Não houve relatos de eventos adversos graves. Elevação das transaminases hepáticas e da enzima creatina fosfoquinase (CPK), que são particularmente preocupantes em adultos, não diferiram nos grupos estudados. ^362^

Recomenda-se a avaliação basal de CPK, transaminases (TGO, TGP) e HbA1C (pelo risco de diabetes com o uso de estatinas). O seguimento laboratorial deve ser realizado a cada 4 a 8 semanas, até a estabilização das doses utilizadas, e, em seguida, de 6 em 6 meses ^59^^,^^329^ (recomendação: I; evidência: C). Para pacientes com sintomas possivelmente relacionados às estatinas é recomendável prosseguir com avaliação laboratorial na vigência dos sintomas, o que pode auxiliar o pediatra a discernir se há de fato relação com o medicamento. Além disso, o grupo de adolescentes deve ser orientado quanto a medidas de contracepção, em virtude do potencial teratogênico das estatinas ^331^ (recomendação: I; evidência: C).

Caso anormalidades laboratoriais (níveis de transaminases elevadas mais de 3 vezes; e ou níveis de CPK > 3 a 10 vezes em pacientes assintomáticos; ou níveis de CPK > 10 vezes em pacientes assintomáticos) sejam detectadas a estatina deve ser interrompida e um novo teste realizado após 2 semanas. Diante da normalização laboratorial a estatina pode ser reiniciada com a devida monitoração ^331^ (recomendação: I; evidência: C).

### 10.6. Aspectos Psicoló gicos

O tratamento farmacológico parece n ão impactar na qualidade de vida ou na ansiedade de crianças portadoras de hipercolesterolemia familiar. Cerca de 40% das crianças sofrem por serem portadoras da condição, mas utilizar hipolipemiantes confere maior segurança em cerca de 60%. Mais de 50% fazem dieta, e 79% dos pais sofrem porque os filhos tê m HF ^363^ (recomendação: IIb; evidência: B).

## 11. Tratamento da Hipercolesterolemia Familiar na Gravidez

Durante a gravidez e a lactação, as opções terapêuticas da HF são bastante limitadas, já que estatinas, ezetimiba, inibidores de PCSK9 e ácido nicotínico não devem ser prescritos de modo a evitar potenciais efeitos adversos no feto associados ao uso desses agentes (respectivamente categorias X, C, B e C). Isso pode ser preocupante, considerando-se o aumento das taxas plasmáticas de lipídios que ocorre geralmente durante a gravidez, de 25 a 50% dos níveis de colesterol e 150 a 300% de triglicerídeos, em adição a concentrações basais de colesterol já bastante elevadas em razão da HF. ^364^

O uso de outras medicações hipolipemiantes, mais especificamente das resinas, é possível quando há necessidade clara da manutenção de terapêutica medicamentosa com provável benefício. As resinas, como o colesevelam e a colestiramina, são agentes da categoria B na gravidez e lactação e, portanto, podem ser consideradas para tratamento da HF nessas condições, desde que sob supervisão médica. ^365^ A LA é uma modalidade de tratamento que também pode ser utilizada em casos especiais, em que o risco cardiovascular da paciente, na ausência de tratamento, é muito alto, como em pacientes com HFHo ou com HFHe e doença aterosclerótica grave. ^366^

As mulheres portadoras de HF em idade fértil e que desejem engravidar devem receber aconselhamento pré-gravidez e suspender estatinas, ezetimiba e ácido nicotínico, pelo menos quatro semanas antes de interromperem o método contraceptivo utilizado. É importante destacar que o uso de anticoncepcional oral geralmente não é contraindicado para a maioria de mulheres com HF ^367^ e não interfere na eficácia das estatinas. ^368^ Mulheres com risco aumentado de eventos cardiovasculares devem discutir outros métodos contraceptivos além do anticoncepcional oral. ^367^

As pacientes que engravidaram de forma não programada devem suspender esses hipolipemiantes imediatamente e procurar acompanhamento obstétrico. Alguns poucos estudos avaliaram mulheres com HF que engravidaram em uso de estatinas, com resultados controversos, em relação à incidência de malformações fetais. Em série relatada pela Food and Drug Administration (FDA) em 2004, foram avaliados 52 casos selecionados de exposição gestacional a estatinas, entre os quais 20 tinham defeitos estruturais fetais, especialmente neurológicos e esqueléticos. ^369^

A escassez relativa de tratamentos seguros e eficazes para redução das taxas de colesterol plasmático nessas pacientes se associa à preocupação em relação a efeitos adversos pela própria hiperlipidemia. De fato, alguns trabalhos sugerem risco aumentado de prematuridade em grávidas com taxas elevadas de colesterol. ^370^^,^^371^ Trabalho recente realizado na Noruega, que avaliou 2.319 nascimentos de 1.093 mulheres com HF, não detectou diferença em relação à prematuridade entre mulheres com diagnóstico genético de HF e mulheres da população geral. ^364^ Quanto ao baixo peso ao nascer, em geral também não parece haver diferença significativa entre recém-nascidos de mulheres com ou sem o diagnóstico de HF. A frequência de malformações congênitas em fetos de mulheres com HF também não parece maior em comparação a mulheres da população geral, respectivamente, 3,3 e 3,2%. Toleikyte et al. ^364^ também não encontraram diferenças em prematuridade, baixo peso e malformações segundo diferentes tipos de mutação genética.

Embora a maioria dos estudos disponíveis não demonstre eventos adversos fetais significativos em associação à presença de HF, é recomendável o acompanhamento conjunto das gestantes portadoras da doença pelo especialista em lipídios e pelo obstetra. Deve-se estar atento à possível presença de lesões valvares, em particular de estenose aórtica, e de doença coronária prematura nessas pacientes. ^372^ Do ponto de vista obstétrico, também é importante a pesquisa de insuficiência vascular uteroplacentária. ^373^^,^^374^

### 11.1. Conclusões

O uso de medicações hipolipemiantes em gestantes com HF incluindo estatinas, ezetimiba, ácido nicotínico, fibratos, inibidores PCSK9 não é recomendado (recomendação: classe III; nível de evidência: B). Apenas as resinas (recomendação: classe IIB; nível de evidência: B) e aférese (recomendação: classe IIB; nível de evidência: B) podem ser utilizadas durante a gestação.

### 11.2. Classificação dos Agentes Quanto a Possíveis Efeitos no Feto Segundo a FDA

- Categoria A: estudos adequados e controlados não demonstraram risco ao feto no primeiro trimestre da gravidez (e não há evidência de risco nos trimestres seguintes).
- Categoria B: estudos de reprodução em animais não demonstraram risco ao feto, e não há estudos adequados e controlados em mulheres grávidas.
- Categoria C: estudos de reprodução em animais demonstraram efeito adverso ao feto, mas não há estudos adequados e controlados em mulheres grávidas.
- Categoria D: existe evidência de risco ao feto humano baseada em dados de reação adversa provenientes de estudos em humanos ou experiência de *marketing* ou investigativa. Os benefícios do uso do agente em mulheres grávidas podem ser superiores ao seu risco em algumas situações.
- Categoria X: estudos em animais ou humanos demonstraram anormalidades fetais e/ou existe evidência de risco fetal humano baseado em dados de efeitos adversos provenientes de experiência de *marketing* ou investigativa. Os riscos do uso do agente em mulheres grávidas superam claramente potenciais benefícios.

## 12. Terapias Hipolipemiantes para as Formas Graves de Hipercolesterolemia Familiar

A HF é uma patologia de difícil tratamento e que provoca eventos cardiovasculares em idade precoce. Os indivíduos acometidos podem apresentar-se de duas formas: HFHe e HFHo.

A meta de LDL-c preconizada em diretrizes não é geralmente alcançada por terapias convencionais, mesmo com a utilização de estatinas de alta potência em doses máximas, havendo necessidade da introdução de terapêutica adjuvante. Por outro lado, a redução de 50% do LDL-c, meta também preconizada, pode ser alcançada com estatinas de alta potência. ^9^ Metas individualizadas para HFHe e HFHo de acordo com as respectivas categorias de risco foram discutidas nas seções 7 e 9.

Hipolipemiantes adicionais à administração de estatinas são geralmente necessários no tratamento da HF e, apesar de seus efeitos colaterais, são empregados para a redução de LDL-c e, principalmente, de desfechos robustos cardiovasculares, tais como IAM fatal ou não, AVE fatal ou não e morte cardiovascular. Entre eles, estão:

- Anticorpos monoclonais antiPCSK9.
- Inibidor microssomal da proteína de transferência de triglicerídeos (MTP) (lomitapida).
- Oligonucleotídeo antissentido.

### 12.1. Anticorpos Monoclonais AntiPCSK9 na Hipercolesterolemia Familiar

Em estudos de genética mendeliana clássica, pesquisadores identificaram uma região no cromossomo 1 que estava ligada à presença de HF, responsável pela transcrição do gene PCSK9, cujo polimorfismo transmite a doença de forma autossômica dominante. ^16^^,^^375^ Estudos mecanísticos subsequentes demonstraram que a PCSK9 funciona como chaperona do LDLR e que, uma vez ligada ao receptor, previne a alteração conformacional necessária para prevenir a sua degradaço no lisossoma. ^3^^,^^76^ Como consequência, o LDLR que pode recircular cerca de 100 vezes é degradado precocemente, reduzindo a remoção da LDL da circulação sanguínea. Consistentemente, mutações com ganho de função da PCSK9 se associam com aumento do LDL-c e do risco cardiovascular e aquelas com perda de função com níveis reduzidos de LDL-c e com baixo risco cardiovascular. ^377^^,^^378^

Anticorpos monoclonais foram desenvolvidos para reduzir a biodisponibilidade da PCSK9, impedindo assim sua ligação ao receptor da LDL. Dentre eles, o evolocumabe e alirocumabe foram aprovados para uso clínico. Ambos são anticorpos monoclonais totalmente humanos e são administrados por via subcutânea. O evolocumabe pode ser administrado na dose de 140 mg a cada duas semanas ou em 420 mg por mês. Nas duas posologias, o evolocumabe reduz LDL-c em aproximadamente 60%, quando administrado isoladamente ou sobre o tratamento com outros hipolipemiantes. ^377^^,^^379^

Em indivíduos com HFHe, uma redução semelhante foi observada; ^283^^,^^284^ porém, em homozigotos, a redução alcançada foi de 38%. ^286^ Naturalmente, o grau de resposta dependerá do tipo e número de mutações. Nos indivíduos com dois alelos mutados defeituosos (captação de LDL de 2 a 25%), o tratamento pode reduzir LDL-c em até 47%. ^286^ Naqueles com um alelo defeituoso e um alelo negativo, ou seja, sem produção do LDLR (captação de LDL < 2%), o efeito máximo esperado é de 25%. ^286^ Por fim, nos indivíduos com dois alelos negativos (nenhuma captação de LDL), nenhuma resposta é obtida com o tratamento. ^286^ Os dados do estudo TESLA B foram amplificados pela análise interina do estudo TAUSSIG, no qual foram incluídos 106 portadores de HFHo. ^380^ Nesse estudo aberto, após 12 semanas de uso mensal de evolocumabe 420 mg, a redução média do LDL-c foi de 21% (desvio padrão 24%). Da mesma maneira como no estudo TESLA B, houve variabilidade da resposta dependente em parte do defeito genético causador do fenótipo da HF. Homozigotos que apresentavam defeitos nos genes *APOB* ou *LDLRAP1* (causador da hipercolesterolemia autossômica recessiva com fenótipo similar ao da HFHo) tiveram reduções médias de 47 e 15%, respectivamente. As respostas foram sustentadas até 4 anos de tratamento, e os eventos adversos mais frequentes foram sintomas de nasofaringite e resfriado comum.

Assim, a terapia com evolocumabe deve ser tentada em indivíduos com HFHo, exceto naqueles homozigotos para mutações nulas no LDLR. No entanto, esses pacientes provavelmente irão requerer terapias adicionais.

O alirocumabe foi testado nas doses de 75 mg e 150 mg a cada duas semanas. Na dose mais baixa é esperada uma redução de LDL-c de 45 a 50%, e na dose mais alta, 60%. ^284^^,^^381^^,^^382^ Na posologia de 300 mg mensais, o alirocumabe reduz o LDL-c em 55 a 60%. ^383^ À semelhança do evolocumabe, o alirocumabe reduz o LDL-c em pacientes com HFHe em aproximadamente 40 a 60%. ^384^ Igualmente consistente, em pacientes com HFHo, as reduções de LDL-c dependem do genótipo e variam de 7 a 64%. ^385^ O alirocumabe foi aprovado para a HFHo pelo FDA, mas aguarda a aprovação na Agência Regulatória Européia (EMA) e no Brasil (ANVISA).

Três ensaios cardiovasculares randomizados, duplo-cegos e controlados por placebo avaliaram a eficácia da terapia com inibidores da PCSK9 no risco de eventos cardiovasculares. Embora nenhum desses ensaios tenha sido dirigido a pacientes com HF, seus resultados são extrapoláveis a essa população. No estudo FOURIER, o evolocumabe foi testado contra placebo em 27.564 pacientes com DCV aterosclerótica clinicamente evidente caso apresentassem LDL-c ≥ 70 mg/dl, a despeito de terapia hipolipemiante otimizada. ^386^ De um valor basal de LDL-c de 92 mg/dl, os pacientes arrolados para evolocumabe reduziram para um nível médio de 30 mg/dl, que foi mantido ao longo do tempo (mediana de seguimento de 2,2 anos). ^287^ A terapia com evolocumabe reduziu o risco do desfecho combinado de morte cardiovascular, infarto do miocárdio, AVE, revascularização coronariana ou hospitalização por angina instável em 15% (HR 0,85, IC 95% 0,79 a 0,92). O desfecho principal secundário, composto de morte cardiovascular, infarto do miocárdio ou AVE, foi reduzido em 20% (HR 0,80, IC 95% 0,73 a 0,88) ^287^ . À semelhança dos estudos com estatinas, o benefício clínico aumentou progressivamente com o tempo de tratamento. ^287^ Em um subestudo neurocognitivo, 1.974 pacientes do estudo FOURIER foram avaliados antes e após a intervenção para que se pudesse avaliar a segurança sobre a função cognitiva. ^387^ Não foram observadas diferenças entre grupos na função cognitiva, e não houve associação entre os níveis de LDL-c e alterações cognitivas.

Nos estudos SPIRE-1 e SPIRE-2, ^388^ o bococizumabe foi administrado a 27.438 pacientes com evento cardiovascular prévio ou história de diabetes, doença renal crônica ou doença vascular periférica. O estudo foi interrompido prematuramente após a constatação de que o efeito do bococizumabe não foi sustentado devido ao desenvolvimento de anticorpos neutralizantes. ^389^ O nível basal de LDL-c foi de 109 mg/dl, e 85% dos indivíduos estavam recebendo terapia com estatina de alta intensidade. O bococizumabe diminuiu o LDL-c em 59% em 14 semanas, mas esse efeito foi atenuado para 38% em 2 anos. ^389^ No estudo SPIRE-2, que teve um seguimento mais longo, o bococizumabe reduziu em 21% (HR 0,79, IC 95% 0,65 a 0,97; p = 0,02) o risco do desfecho primário composto de morte cardiovascular, infarto do miocárdio, AVE ou revascularização coronariana. ^390^ Um subestudo do programa SPIRE mostrou que não houve heterogeneidade na redução do risco cardiovascular entre indivíduos com e sem HF com o uso do bococizumabe. ^390^

Por fim, o estudo ODYSSEY Outcomes arrolou 18.924 pacientes 1 a 12 meses após infarto do miocárdio ou angina instável para tratamento com alirocumabe ou placebo. ^391^ A dose de alirocumabe foi titulada para atingir um nível de LDL-c de 25 a 50 mg/dl, e o medicamento deveria ser interrompido se o nível de LDL-c fosse persistentemente inferior a 15 mg/dl. O alirocumabe diminuiu o LDL-c em 57% em 4 semanas, mas esse efeito foi atenuado progressivamente para 36% no final do estudo, presumivelmente devido ao esquema de titulação de doses. ^288^ O desfecho primário composto de morte cardiovascular, infarto do miocárdio, AVE isquêmico ou hospitalização por angina instável foi reduzido em 15% (HR 0,85, IC 95% 0,78 a 0,93; p = 0,003). ^288^ Foi observada uma redução de 15% (HR 0,85, IC 95% 0,73 a 0,98) na mortalidade por todas as causas com tratamento com alirocumabe em comparação com placebo. ^288^ No entanto, por não haver redução significante de dois desfechos hierarquicamente prioritários nessa análise, mortalidade cardiovascular e coronariana, o resultado foi considerado de valor exploratório, e não uma evidência.

### 12.2. Inibidor Microssomal da Proteína de Transferência de Triglicerídeos (Lomitapida)

A MTP é uma proteína transferidora de lipídios encontrada no retículo endoplasmático de hepatócitos e enterócitos, que atua na montagem de lipoproteínas que contêm Apo B. ^392^

A lomitapida é uma pequena molécula que inibe a MTP, reduzindo a formação de quilomícrons no intestino e de VLDL no fígado. É terapia adicional às estatinas, utilizada na redução das concentrações de LDL-c, principalmente em indivíduos portadores de HF.

Por ser a VLDL precursora metabólica de LDL, as concentrações plasmáticas de LDL seriam reduzidas. ^393^ Na ausência ou disfunção da MTP, semelhante ao que ocorre na hipo ou abetalipoproteinemia recessiva, não há produção de VLDL e, consequentemente, das demais lipoproteínas que contêm Apo B, tais como LDL, IDL e Lp(a). Atualmente, seu uso tem aprovação da FDA e da Agência Regulatória Europeia como terapia adjuvante em adultos com HFHo; entretanto, já está documentado seu uso em criança. ^394^ Recentemente a lomitapida foi aprovada em nosso país em 2020 pela Agência Nacional de Vigilância Sanitária (ANVISA) para uso em adultos na hipercolesterolemia familiar homozigótica.

A lomitapida é administrada por via oral, na dose inicial de 5 mg/dia e pode chegar a 60 mg/dia, sendo que a dose deve ser individualizada de acordo com as metas terapêuticas e com a resposta e tolerância individual ao tratamento. Estudo de fase 3 em pacientes com HFHo, em doses iniciais de 5 mg/dia e tituladas até 60 mg/dia, associadas à terapia de base, mostrou reduções adicionais de 50% no LDL-c e de 49% na Apo B. ^395^ Ainda não foram descritas alterações em concentrações de HDL-c e Lp(a) com a manutenção do tratamento com lomitapida após 78 semanas, exceto discretas flutuações de HDL-c. ^396^

Estudo de fase 3 com 26 semanas de tratamento com lomitapide avaliou o alcance de metas da *European Atherosclerosis Society* e da ocorrência de eventos cardiovasculares maiores (MACE). O alcance de meta de LDL-c < 100 mg/dL foi de 51% e < 70 mg/dL foi de 28% nas primeiras 26 semanas. ^397^ Na fase de extensão, nos pacientes que continuaram com lomitapide após 176 semanas (N=19), 74% alcançaram meta de LDL-c < 100 mg/dL e 58% < 70 mg/dL em pelo menos uma dosagem. O LDL-c sob tratamento com lomitapide alcançado foi de 166 mg/dL. Houve dois eventos cardiovasculares, uma morte cardíaca e uma revascularização do miocárdio, equivalente a 1,7 eventos por 1000 pacientes por mês de tratamento. Esses valores são muito menores do que os observados entre as coortes de pacientes com HF antes do uso das novas terapias. ^398^

Dados de mundo real, com 18 pacientes com HFHo em tratamento adjuvante com lomitapide em dose média de 19 mg/dia num seguimento de 32,3 ± 29,7 meses mostraram redução de LDL-c de 68,2 ± 24,8% e na visita final, 60% dos pacientes alcançaram meta de LDL-c < 100 mg/dL e, 46,6% < 70 mg/dL; 80% dos pacientes deixaram de necessitar LDL-aférese, devido aos valores alcançados de LDL-c. A redução do LDL-c foi variável (13-95%), independente do genótipo. ^392^ No seguimento, 53,3% tiveram eventos adversos, mas nenhum grave. Não houve aumento de transaminases > 5x o LSN e nenhum paciente interrompeu a medicação por eventos adversos. Cinco pacientes realizaram ultrassom hepático e fibroscan ou ressonância nuclear magnética com espectroscopia e nenhum deles apresentou indícios de dano hepático. ^399^

Dados de registro de 5 anos em pacientes com HFHo que iniciaram lomitapide (N=187) foram consistentes quanto à eficácia e segurança nos estudos de fase 3, apesar do uso de uma dose mais baixa (10 mg vs 40 mg) nos estudos de fase 3. Não houve novos achados de segurança e a incidência de eventos adversos, eventos adversos sérios, elevações de alanina aminotransferase foi menor do que nos estudos de fase 3, provavelmente relacionados à menor dose utilizada. ^400^

Os efeitos adversos mais comuns foram gastrintestinais, como náuseas, flatulência e diarreia. Eles podem ser minimizados pela redução da ingesta de gordura ou pela titulação escalonada do medicamento. ^398^ Tem sido descrito, em alguns pacientes, aumento das transaminases, em geral reversível com a redução ou a descontinuação do fármaco, ou mesmo transitório com a manutenção do tratamento.

Na maioria das vezes, não foram descritos elevação concomitante das bilirrubinas, fosfatase alcalina e surgimento de sintomas. Entretanto, o efeito adverso mais preocupante com uso da lomitapida, devido ao seu mecanismo de ação, é a esteatose hepática, que seria dose dependente e que se atenua com a redução da dosagem diária. Pode ser detectada especialmente por ressonância magnética. Outro efeito colateral relevante é o aparecimento de esteatorreia. ^397^^-^^401^

Estudos com ressonância magnética em pacientes com HFHo demonstraram acúmulo de gordura hepática. Porém, isso pode variar individualmente, sendo acentuado por consumo de álcool. Os efeitos desse acúmulo de gordura no fígado, em longo prazo, decorrentes da intervenção medicamentosa poderiam ser deletérios e até ocasionar cirrose hepática. ^399^

Pelo fato de a inibição da MTP levar não apenas à redução hepática da síntese de VLDL, mas também da produção intestinal de quilomícrons, o uso de lomitapida poderia causar redução da absorção de ácidos graxos essenciais e vitaminas lipossolúveis, em especial vitamina E, que é transportada principalmente por LDL. No entanto, esse achado não foi confirmado em portadores de HF após seu uso no tratamento. ^402^

Além disso, pesquisas não demonstraram nenhum efeito significativo do tratamento com lomitapida sobre os níveis plasmáticos de vitaminas A e D. ^399^ Contudo, com a finalidade de prevenir deficiências nutricionais, podem ser suplementados ácidos graxos essenciais e vitaminas lipossolúveis ao tratamento.

Pelo fato de a lomitapida ser amplamente metabolizada pelo CYP3A4, deve-se ter precaução com sua coadministração com inibidores do CYP3A4 (antifúngicos, diltiazem, verapamil, antibióticos como ciprofloxacino, claritromicina e eritromicina, e os inibidores de protease). O uso associado da lomitapida 60 mg/dia com a sinvastatina 40 mg/dia aumentou a exposição a sinvastatina em 1,7 vez comparada à sinvastatina isolada, aumentando o risco de efeito colateral pela sinvastatina. ^403^ Entretanto, estudos com outros hipolipemiantes não demonstraram interações significativas. Outro possível evento adverso provocado por sua abrupta interrupção seria a elevação das concentrações de LDL-c, devido ao efeito rebote na secreção de VLDL.

A lomitapida está contraindicada na gravidez, e não há, até o momento, alguma prova que o seu uso seja seguro nesta ocasião. Sem dúvida, esse questionamento deveria ser colocado antes do seu emprego, em casos de risco, caso fosse necessário. Por fim, sua eficácia hipolipemiante foi demonstrada em estudos prévios que mostraram efeito de dose-resposta na redução de LDL-c, em que 10, 25 e 50 mg diários reduziram LDL-c em 30, 55 e 70%, respectivamente. ^392^

### 12.3. Oligonucleotídeo Antissentido

#### 12.3.1. Inibidores da Síntese de Apo B (Antissentido AntiApo B)

A tecnologia do oligonucleotídeo antissentido pode ser utilizada para bloquear a síntese de determinada proteína alvo. O mipomerseno é um oligonucleotídeo antissentido de segunda geração que se liga ao RNAm que codifica a Apo B-100, levando à sua degradação por ação da enzima RNAase, reduzindo, assim, a produção de Apo B-100, ^404^ Desse modo, inibe a síntese hepática de Apo B-100 e, consequentemente, reduz as concentrações plasmáticas de VLDL, IDL, LDL e Lp(a). ^405^^-^^407^ Sua aplicação se dá por injeção SC, administrada uma vez por semana na dose de 200 mg. ^406^

Em ensaios clínicos de pacientes com HFHo ou HFHe graves, o mipomerseno reduziu o LDL-c em 25 e 28%, respectivamente. Seus eventos adversos incluem: mialgia, fadiga, lesão no local de aplicação, sintoma semelhante à gripe, além do depósito de gordura hepática (esteatose).

O elevado custo dessa tecnologia e seus efeitos colaterais foram impeditivos de seu uso de forma mais ampla; uma possível indicação terapêutica seria em condição extremamente grave e rara de dislipidemia genética, como a HFHo.

O mipomerseno foi analisado em estudo clínico randomizado fase 3, que alocou pacientes portadores de HFHo. A redução média do LDL-c foi significativamente maior no grupo mipomerseno (-24,7%, IC95% 31,6 a 17,7%) do que no grupo placebo (-3,3%, IC95% 12,1 a 5,5%; p = 0,0003). Nesse estudo, o efeito colateral mais comum foi reação local no sítio de aplicação (76% no grupo mipomerseno *versus* 24% no placebo). ^406^

Em 2013, a FDA aprovou o uso do mipomersen para tratamento da HFHo em adultos, faltando informações sobre seu uso em crianças. ^408^ Porém, em 2018 houve interrupção de sua comercialização.

Estudos fase 3 mostraram que a eficácia do produto é bastante variável, com reduções de 25 a 37%, em média, dependendo das características das populações estudadas (formas homozigóticas ou heterozigóticas da HF, hipercolesterolemias graves ou pacientes de alto risco cardiovascular).

### 12.4. RNA Pequeno de Interferência (siRNA)

Outra forma de inibir a ação da PCSK9 é reduzir sua produção tecidual. Os *small interfering RNA* (siRNA) impedem a tradução do RNA mensageiro. ^409^ O inclisiran é um siRNA sintético que inibe a síntese hepática da PCSK9. O fármaco foi testado no programa ORION, administrado por via subcutânea como primeiro na classe, avaliando sua eficácia e segurança na redução do LDL-c. Estudos de fase II e fase III demonstraram redução de LDL-c de cerca de 50% com uma dose infrequente, uma aplicação a cada seis meses, em pacientes com doença aterosclerótica cardiovascular estabelecida, ou de alto risco cardiovascular, incluindo pacientes com HF. Estudos de fase III em andamento irão determinar evidências de segurança e eficácia em longo prazo e, na HFHo. Além disso, o estudo ORION-4 irá avaliar o impacto do inclisiran nos eventos cardiovasculares. Sua eficácia já foi demonstrada (em estudo fase 1 e confirmada em estudo fase 2), com redução de 52.6% do LDL-c. ^410^ No estudo ORION-9, um estudo de fase III com pacientes portadores de HFHe, o inclisiran na dose de 300 mg reduziu em 39,7% as concentrações de LDL-c no dia 510 (vs. aumento de 8,2% no grupo placebo) e entre os dias 90 e 540, houve redução de 38,1%. As reduções foram robustas em todos os genótipos encontrados. ^411^ A medicação ainda não foi aprovada para uso.

### 12.5. Anticorpo Monoclonal Anti-ANGPTL3

Outra terapia promissora especialmente para as formas refratárias de HF e para os portadores de HFHo envolve a inibição da ANGPTL3 ( *angiopoietin-like 3 peptide* ), com o anticorpo monoclonal anti-ANGPTL3, o evinacumabe. Na HFHO o evinacumabe reduziu em 47% o LDL-c administrado por via endovenosa na dose de 15 mg/kg de peso a cada 4 semanas. ^412^ O evinacumabe foi efetivo tanto nos pacientes com mutações nulas para o *LDLR* (redução de LDL-c de 43,4%), como para as outras variantes (redução de LDL-c de 49,1%).

Nas formas graves e refratárias de hipercolesterolemia o evinacumabe foi testado por via subcutânea e endovenosa em várias doses e intervalos posológicos, mostrando redução de LDL-c superior a 50%. ^413^ O evinacumab foi recentemente aprovado para uso em pacientes com HFHo pelo FDA.

### 12.6. Ácido Bempedóico

O ácido bempedóico é uma pequena molécula que atua na redução do LDL-c pela inibição de uma enzima-chave na via de biossíntese do colesterol, a ATP citrato liase, que atua um passo antes da 3-hydroxy-3-metilglutaril-coenzima A. Diferente das estatinas, tem ação específica no fígado, sem interferência nos músculos esqueléticos. ^414^ Num estudo randomizado de fase III, um total de 779 pacientes com doença aterosclerótica cardiovascular, hipercolesterolemia familiar heterozigótica ou ambos, e com LDL-c tratado > 70 mg/dL foram incluídos. O ácido bempedóico reduziu o LDL-c em 15,1%, além de reduzir o colesterol não-HDL, colesterol total, apoB e proteína C-reativa de alta sensibilidade, em comparação ao placebo. ^415^ Já no estudo usando combinação fixa de ácido bempedóico 180 mg e ezetimiba 10 mg em pacientes com hipercolesterolemia recebendo dose máxima tolerada de estatina e de alto risco cardiovascular, a redução de LDL-c foi de 36%, comparada à ezetimibe isolada (-23,2%) e ácido bempedóico isolado (-17,2%). ^416^ A redução com o ácido bempedóico / ezetimiba foi semelhante entre os grupos recebendo estatina de alta ou média intensidade e naqueles sem estatina. Os outros lípides e a PCR-us também tiveram redução maior com a terapia combinada.

Esta classe de fármacos é associada a elevações modestas no nível de ácido úrico, explicada pela competição do metabólito do medicamento e do ácido úrico para os mesmos transportadores renais envolvidos na excreção desses compostos. Dados de segurança com exposição prolongada, bem como de desfechos cardiovasculares, estão sendo avaliados em estudo de fase 3, ainda sem resultados publicados.

## 13. Custo-efetividade do Rastreamento e Tratamento da Hipercolesterolemia Familiar

### 13.1. Introdução

Na atualidade, os recursos para a saúde são escassos para atender a todas as demandas da sociedade. A medicina contemporânea se depara com uma avalanche de terapias que comprovadamente acrescentam um benefício clínico em relação às terapias já incorporadas, mas geralmente vem associadas a um determinado custo incremental.

Tradicionalmente, quando se avaliam as diversas intervenções médicas, tanto do ponto de vista clínico quanto de política de saúde, a preocupação é estabelecer a eficácia e segurança de uma intervenção, que pode ser alcançada quando aplicada em condições ideais (ensaios clínicos controlados e randomizados). A efetividade mostrará o real efeito da intervenção quando utilizada nas circunstâncias usuais (estudos de mundo real). Entretanto, há ainda o conceito de eficiência das intervenções, que considera não apenas a efetividade de cada intervenção, mas também os recursos necessários para que a mesma seja implementada.

Esse panorama tem despertado o interesse da comunidade e seus diversos segmentos na busca de soluções. A Economia da Saúde é uma área do conhecimento interdisciplinar que pode auxiliar médicos, gestores e formuladores de políticas de saúde na difícil tarefa de tomar decisões em ambiente de escassez de recursos.

### 13.2. Estudo de Custo da Doença

Para análise do impacto de uma doença ou um tratamento específico em um cenário (p. ex., país, sistema de saúde, hospital), há necessidade do conhecimento do custo da doença. Esse tipo de estudo econômico é um método descritivo que associado aos dados de prevalência, incidência, morbidade e mortalidade, auxilia na mensuração do impacto para a sociedade de uma doença específica.

O estudo do custo da doença não é categorizado como análise econômica, pois não compara intervenções e não avalia desfechos em saúde. O objetivo é estimar a carga ou o impacto de uma doença para priorizar a alocação de recursos em políticas públicas de saúde, orientar fundos para pesquisa e identificar as doenças que mais comprometem o orçamento da saúde, além de fornecer informações para análises econômicas.

### 13.3. Análises Econômicas em Saúde

As análises econômicas comparam as opções para a alocação dos recursos escassos destinados à área de saúde, entre alternativas que competem pelo seu uso. Todas as formas de análise econômica envolvem o uso de recursos e os benefícios de saúde de intervenções terapêuticas, preventivas ou mesmo de programas de saúde. Essas análises proporcionam a comparação entre as alternativas e facilitam o processo de escolha do uso apropriado dos recursos de saúde.

A análise de custo-efetividade (ACE) é o tipo de análise mais utilizado em saúde e mensura o custo em unidades monetárias dividido por uma unidade não monetária, chamada *unidade natural* , como, por exemplo, anos de sobrevida ou eventos evitados após uma determinada intervenção em saúde. ^417^

Uma intervenção em saúde é dita custo-efetiva se produz um benefício clínico justificável para o seu custo. A determinação do quanto a efetividade adicional justifica o custo extra é tomada pela sociedade e depende de valores sociais e da disponibilidade de recursos. A OMS recomenda o valor de um a três vezes o produto interno bruto (PIB) *per capita* do país onde a análise foi realizada, como limite de custo-efetividade justificável para aquele contexto. ^418^ No cenário brasileiro, não foi definido um valor explícito do limiar de custo-efetividade para o Sistema Único de Saúde (SUS) ou sistema suplementar de saúde, ou seja, a definição do valor monetário a partir do qual se considera uma intervenção custo-efetiva (limiar de custo-efetividade). A definição desse valor é contexto-específica, depende da riqueza local, das características do sistema de saúde, da disponibilidade e capacidade de pagar, bem como das preferências sociais. Além disso, o uso desse limiar deverá sempre ser feito em conjunto com outros critérios que agreguem “valor” e tem sido discutido nos últimos anos.

### 13.4. Custos da Hipercolesterolemia Familiar

As DCV são as principais causas de morte e de custos da população adulta no Brasil, e provavelmente esse impacto aumentará com o aumento da expectativa de vida. A HF é um importante fator de risco para a doença aterosclerótica, aumentando o risco de eventos precoces.

Siqueira et al., ^419^ estimaram os custos das DCV no Brasil na perspectiva do SUS durante 5 anos, incluindo os custos diretos com hospitalizações, atendimentos ambulatoriais e benefícios concedidos pela previdência, além dos custos indiretos com a perda de renda causada pela mortalidade da DCV. Esta representou 28% do total de óbitos ocorridos no Brasil e atingiu 38% dos óbitos na faixa etária produtiva (18 a 65 anos). Os custos estimados por DCV foram de R$ 37,1 bilhões no ano de 2015, com um aumento percentual de 17% no período de 2010 a 2015. Os custos estimados pela morte prematura por DCV representam 61% do total de custo por DCV; os custos diretos com internações e consultas foram de 22%, e os custos pela perda da produtividade relacionados à doença foram de 15% do total. Os gastos com saúde no Brasil foram estimados em 9,5% do PIB, e o custo médio das DCV foi estimado em 0,7% do PIB.

Bahia et al. ^420^ estimaram os custos das hospitalizações por DAC atribuíveis à presença de HF na população adulta brasileira na perspectiva do SUS no período de 2012 a 2014. Utilizando dados da literatura internacional sobre a prevalência de HF e o risco relativo de eventos, foram calculadas as frações atribuíveis populacionais (FAP) e aplicadas sobre os custos das hospitalizações em todas as unidades de saúde do SUS por meio da base de dados SIH-SUS. Um total de 245.981 hospitalizações foram registradas em um ano, sendo que, entre 7.249 (2,9%) e 12.915 (5,2%), seriam atribuíveis a presença de HF, dependendo da prevalência considerada. O custo total dessas hospitalizações foi de R$ 985.919.299, sendo que R$ 29.053.500 a R$ 51.764.175 seriam atribuíveis à presença de HF e poderiam ser minimizados caso houvesse um controle adequado desse fator de risco na população com tratamento adequado.

### 13.5. Custo-efetividade do Rastreamento e Tratamento da Hipercolesterolemia Familiar

Estima-se que menos de 25% dos indivíduos com HF tenham diagnóstico, com a maioria sendo incorretamente tratada. ^421^ As implicações clínicas e econômicas desse baixo número de diagnósticos são significativas, já que uma importante parcela da população com HF não tratada precocemente irá evoluir para DCV aterosclerótica. Com isso, um importante ponto de discussão é a custo-efetividade do rastreamento populacional da HF para um diagnóstico e tratamento precoces. Rosso et al. ^422^ realizaram uma revisão sistemática de análises econômicas do rastreamento genético da HF (custo-efetividade, custo-utilidade, custo-benefício e custo-minimização). Sete avaliações econômicas foram realizadas na Europa entre 2002 e 2015 com foco nos parentes de casos índice com diagnóstico genético ou clínico de HF (rastreamento em cascata), mas não comparando com uma estratégia de não rastreamento. Na perspectiva do sistema de saúde pagador, apenas os custos diretos foram analisados, o que evidencia estimativas de impacto conservadoras, pois não levaram em consideração os custos indiretos da DCV (absenteísmo, licenças, aposentadorias e mortes precoces). Em todos os contextos, o rastreamento em cascata foi custo-efetivo, exceto em estudo norte-americano que não mostrou evidência de benefício econômico do rastreamento genético em comparação com a recomendação vigente de manejo das dislipidemias. ^423^

A efetividade e custo-efetividade do tratamento com estatinas de indivíduos com HF já foi claramente demonstrada em diferentes contextos para prevenção secundária. ^424^^,^^425^ Para a prevenção primária, a custo-efetividade dependerá do risco cardiovascular e idade da população, com resultados que suportam o valor do tratamento mais agressivo em indivíduos de maior risco. ^426^ No Brasil, uma análise de custo-efetividade de três esquemas de dosagem (baixa, intermediária e alta intensidade) das estatinas foi realizada na perspectiva do SUS. Com base nos preços de aquisição governamental, o esquema terapêutico com estatinas de potência intermediária (p. ex., atorvastatina 10 mg e sinvastatina 40 mg) foi o que apresentou melhor relação de custo-efetividade e foi sugerida como a abordagem econômica mais atrativa para o SUS tanto na prevenção primária (população de alto risco CV), quanto secundária. ^427^

As novas abordagens de tratamento com os inibidores da PCSK-9 necessitam de análises econômicas contextualizadas, já que são medicamentos que demonstram eficácia adicional ao tratamento de indivíduos de alto risco com risco residual, porém com um custo de tratamento muito elevado. A relação custo-efetividade dessas novas terapias é importante à medida que os custos dos serviços de saúde aumentam significativamente, e informações precisas sobre o valor e comparação entre os benefícios potenciais entre as terapias são essenciais para análises de impacto orçamentário.

Nos EUA, três estudos de modelagem demonstraram que o tratamento com inibidores de PCSK9 em indivíduos com DCV estabelecida excedeu os limites de custo-efetividade geralmente aceitos e poderiam aumentar significativamente os custos da assistência médica. ^428^^-^^430^ O custo médio de tratamento anual considerado nos modelos foi de US$ 14.000 a 15.000, e os autores sugerem que a redução do custo deveria ser de mais de 50% por ano para que a terapia fosse considerada custo-efetiva para a redução de eventos cardiovasculares.

### 13.6. Considerações Finais

A contribuição dos estudos de custo da doença e das análises econômicas é instrumentalizar os médicos e gestores para o processo de tomada de decisão, momento no qual se decide o que, quanto, para quem, a que custo, e qual o benefício da ação que está sendo produzido, possibilitando guiar o processo de decisão de modo racional e com vistas a alcançar o maior benefício coletivo com os recursos disponíveis.

Constantemente, são feitas escolhas entre tratamentos, com custos e efetividades diferentes, para uma mesma finalidade clínica. É importante, portanto, procurar a “melhor evidência” para o tratamento da enfermidade em questão e analisar o benefício proporcionado ao paciente, idealmente aquele que proporcione a melhor relação custo-efetividade. Na prática clínica do mundo real, onde existe escassez de recursos e desigualdade no acesso à saúde, o uso dos estudos da área de economia da saúde pode auxiliar muito nos processos decisórios.

## Footnotes

* No caso de doença subclínica aterosclerótica avançada diagnosticada como CAC superior a 100 UA ou 75% do percentil para idade e sexo, ou angiotomografia computadorizada coronariana apresentando obstruções coronárias em mais de 50%, ou presença de placas não obstrutivas em mais de um vaso, deve-se buscar reduzir o LDL-c em ao menos 50%, sendo ideal chegar a níveis < 50 mg/dL.

** São considerados fatores de risco adicionais na HF: ^56^ idade > 40 anos e sem tratamento, tabagismo, sexo masculino, Lp(a) > 50 mg/dL, HDL-c < 40 mg/dL, hipertensão arterial, diabetes melito, história familiar de DAC prematura em parentes de 1º grau (homens < 55 anos e mulheres < 60 anos), doença renal crônica (TFG < 60 ml/min) e IMC > 30 kg/m^2^.
