# Supplementary material for: Atualização da Diretriz Brasileira de Hipercolesterolemia Familiar – 2021
Source: Arq Bras Cardiol. 2021 Oct 6;117(4):782–844. [Article in Portuguese] doi: 10.36660/abc.20210788 (PMC8528358; doi:10.36660/abc.20210788)
Supplement: Translation

# Translation: Update of the Brazilian Guideline for Familial Hypercholesterolemia – 2021

**Authors:** Maria Cristina de Oliveira Izar, Viviane Zorzanelli Rocha Giraldez, Adriana Bertolami, Raul Dias dos Santos, Ana Maria Lottenberg, Marcelo Heitor Vieira Assad, José Francisco Kerr Saraiva, Ana Paula M. Chacra, Tania L. R. Martinez, Luciana Ribeiro Bahia, Francisco Antonio Helfenstein Fonseca, Andre Arpad Faludi, Andrei C. Sposito, Antônio Carlos Palandri Chagas, Cinthia Elim Jannes, Cristiane Kovacs Amaral, Daniel Branco de Araújo, Dennys Esper Cintra, Elaine dos Reis Coutinho, Fernando Cesena, Hermes Toros Xavier, Isabela Cardoso Pimentel Mota, Isabela de Carlos Back Giuliano, José Rocha Faria, Juliana Tieko Kato, Marcelo Chiara Bertolami, Marcio Hiroshi Miname, Maria Helane Costa Gurgel Castelo, Maria Sílvia Ferrari Lavrador, Roberta Marcondes Machado, Patrícia Guedes de Souza, Renato Jorge Alves, Valeria Arruda Machado, Wilson Salgado

**Affiliations:** 1 São PauloSP Brazil Universidade Federal de São Paulo (UNIFESP), São Paulo, SP – Brazil; 2 São PauloSP Brazil Instituto do Coração (InCor) da Faculdade de Medicina da Universidade de São Paulo (FMUSP), São Paulo, SP – Brazil; 3 São PauloSP Brazil Grupo Fleury, São Paulo, SP – Brazil; 4 São PauloSP Brazil Instituto Dante Pazzanese de Cardiologia, São Paulo, SP – Brazil; 5 São PauloSP Brazil Universidade de São Paulo, São Paulo, SP – Brazil; 6 São PauloSP Brazil Hospital Israelita Albert Einstein (HIAE) – Faculdade Israelita de Ciências da Saúde Albert Einstein (FICSAE), São Paulo, SP – Brazil; 7 São PauloSP Brazil Faculdade de Medicina da Universidade de São Paulo, Laboratório de Lípides (LIM10), São Paulo, São Paulo, SP – Brazil; 8 Rio de JaneiroRJ Brazil Instituto Nacional de Cardiologia, Rio de Janeiro, RJ – Brazil; 9 CampinasSP Brazil Sociedade Campineira de Educação e Instrução, Campinas, SP – Brazil; 10 São PauloSP Brazil Hospital Beneficência Portuguesa de São Paulo, São Paulo, SP – Brazil; 11 Rio de JaneiroRJ Brazil Universidade do Estado do Rio de Janeiro, Rio de Janeiro, RJ – Brazil; 12 CampinasSP Brazil Universidade Estadual de Campinas (UNICAMP), Campinas, SP – Brazil; 13 São PauloSP Brazil Faculdade de Medicina do ABC (FMABC), São Paulo, SP – Brazil; 14 CampinasSP Brazil Pontifícia Universidade Católica de Campinas, Campinas, SP – Brazil; 15 São PauloSP Brazil Hospital Israelita Albert Einstein (HIAE), São Paulo, SP – Brazil; 16 SantosSP Brazil Cardio-Point Serviços Médicos e Assessoria LTDA, Santos, SP – Brazil; 17 FlorianópolisSC Brazil Universidade Federal de Santa Catarina (UFSC), Florianópolis, SC – Brazil; 18 CuritibaPR Brazil Pontifícia Universidade Católica do Paraná, Curitiba, PR – Brazil; 19 FortalezaCE Brazil Universidade Federal do Ceará (UFC), Fortaleza, CE – Brazil; 20 FortalezaCE Brazil Hospital do Coração de Messejana, Fortaleza, CE – Brazil; 21 FortalezaCE Brazil Professora da Faculdade Unichristus, Fortaleza, CE – Brazil; 22 SalvadorBA Brazil Hospital Universitário Professor Edgard Santos da Universidade Federal da Bahia (UFBA), Salvador, BA – Brazil; 23 São PauloSP Brazil Santa Casa de Misericórdia da São Paulo, São Paulo, SP – Brazil

**Table:** 

Update of the Brazilian Guideline for Familial Hypercholesterolemia – 2021	
The report below lists declarations of interest as reported to the SBC by the experts during the period of the development of these statement, 2020/2021.	
Expert	Type of relationship with industry
Adriana Bertolami	Nothing to be declared
Ana Maria Lottenberg	Financial declaration
	Nothing to be declared
	Ana Paula M. Chacra	Nothing to be declared
Andre Arpad Faludi	Nothing to be declared
Andrei C. Sposito	Other relationships
	Funding of continuing medical education activities, including travel, accommodation and registration in conferences and courses, from the brazilian or international pharmaceutical, orthosis, prosthesis, equipment and implants industry:
	- Sanofi: Medical class.
Antônio Carlos Palandri Chagas	Financial declaration
	A - Economically relevant payments of any kind made to (i) you, (ii) your spouse/partner or any other person living with you, (iii) any legal person in which any of these is either a direct or indirect controlling owner, business partner, shareholder or participant; any payments received for lectures, lessons, training instruction, compensation, fees paid for participation in advisory boards, investigative boards or other committees, etc. From the brazilian or international pharmaceutical, orthosis, prosthesis, equipment and implants industry:
	- Instituto de Vita: Membro do conselho científico; Novo Nordisk: Diabetes/obesidade; Pfizer/ Upjohn: Hipolipemiante.
Cinthia Elim Jannes	Nothing to be declared
Cristiane Kovacs Amaral	Nothing to be declared
Daniel Branco de Araújo	Financial declaration
	A - Economically relevant payments of any kind made to (i) you, (ii) your spouse/partner or any other person living with you, (iii) any legal person in which any of these is either a direct or indirect controlling owner, business partner, shareholder or participant; any payments received for lectures, lessons, training instruction, compensation, fees paid for participation in advisory boards, investigative boards or other committees, etc. From the brazilian or international pharmaceutical, orthosis, prosthesis, equipment and implants industry:
	- Novo Nordisk/ Merk do Brasil: Diabetes; Novartis: Dyslipidemia.
	Other relationships
	Funding of continuing medical education activities, including travel, accommodation and registration in conferences and courses, from the brazilian or international pharmaceutical, orthosis, prosthesis, equipment and implants industry:
	- Sanofi: Dyslipidemia.
Dennys Esper Cintra	Nothing to be declared
Elaine dos Reis Coutinho	Financial declaration
	A - Economically relevant payments of any kind made to (i) you, (ii) your spouse/partner or any other person living with you, (iii) any legal person in which any of these is either a direct or indirect controlling owner, business partner, shareholder or participant; any payments received for lectures, lessons, training instruction, compensation, fees paid for participation in advisory boards, investigative boards or other committees, etc. From the brazilian or international pharmaceutical, orthosis, prosthesis, equipment and implants industry:
	- Novartis: Non-Promotional Activity.
Fernando Cesena	Financial declaration
	A - Economically relevant payments of any kind made to (i) you, (ii) your spouse/partner or any other person living with you, (iii) any legal person in which any of these is either a direct or indirect controlling owner, business partner, shareholder or participant; any payments received for lectures, lessons, training instruction, compensation, fees paid for participation in advisory boards, investigative boards or other committees, etc. From the brazilian or international pharmaceutical, orthosis, prosthesis, equipment and implants industry:
	- Libbs/ Novartis: Dislipidemia; Abbott/ Novo Nordisk: Diabetes; Pfizer: Anticoagulation.
	Other relationships
	Funding of continuing medical education activities, including travel, accommodation and registration in conferences and courses, from the brazilian or international pharmaceutical, orthosis, prosthesis, equipment and implants industry:
	- Novartis; Novo Nordisk: Diabetes.
Francisco Antonio Helfenstein Fonseca	Financial declaration
	A - Economically relevant payments of any kind made to (i) you, (ii) your spouse/partner or any other person living with you, (iii) any legal person in which any of these is either a direct or indirect controlling owner, business partner, shareholder or participant; any payments received for lectures, lessons, training instruction, compensation, fees paid for participation in advisory boards, investigative boards or other committees, etc. From the brazilian or international pharmaceutical, orthosis, prosthesis, equipment and implants industry:
	- Novo Nordisk: Semaglutida; AstraZeneca: Rosuvastatina, Metoprolol, Candesartana; Libbs: Rosuvastatina, Ezetimiba; Sanofi.
	Other relationships
	Funding of continuing medical education activities, including travel, accommodation and registration in conferences and courses, from the brazilian or international pharmaceutical, orthosis, prosthesis, equipment and implants industry:
	- AstraZeneca/ Novo Nordisk: Virtual congress.
Hermes Toros Xavier	Financial declaration
	A - Economically relevant payments of any kind made to (i) you, (ii) your spouse/partner or any other person living with you, (iii) any legal person in which any of these is either a direct or indirect controlling owner, business partner, shareholder or participant; any payments received for lectures, lessons, training instruction, compensation, fees paid for participation in advisory boards, investigative boards or other committees, etc. From the brazilian or international pharmaceutical, orthosis, prosthesis, equipment and implants industry:
	- Torrent do Brasil: Cholesterol; Bayer: Atherothrombosis.
	Other relationships
	Funding of continuing medical education activities, including travel, accommodation and registration in conferences and courses, from the brazilian or international pharmaceutical, orthosis, prosthesis, equipment and implants industry:
	- Torrent do Brasil: Cholesterol.
Isabela Cardoso Pimentel Mota	Financial declaration
	A - Economically relevant payments of any kind made to (i) you, (ii) your spouse/partner or any other person living with you, (iii) any legal person in which any of these is either a direct or indirect controlling owner, business partner, shareholder or participant; any payments received for lectures, lessons, training instruction, compensation, fees paid for participation in advisory boards, investigative boards or other committees, etc. From the brazilian or international pharmaceutical, orthosis, prosthesis, equipment and implants industry:
	- PTC Therapheutics: Rare deseases.
Isabela de Carlos Back Giuliano	Financial declaration
	B - Research funding under your direct/personal responsibility (directed to the department or institution) from the brazilian or international pharmaceutical, orthosis, prosthesis, equipment and implants industry:
	- Genzyme do Brasil: Mipomersen.
José Francisco Kerr Saraiva	Nothing to be declared
José Rocha Faria Neto	Financial declaration
	A - Economically relevant payments of any kind made to (i) you, (ii) your spouse/partner or any other person living with you, (iii) any legal person in which any of these is either a direct or indirect controlling owner, business partner, shareholder or participant; any payments received for lectures, lessons, training instruction, compensation, fees paid for participation in advisory boards, investigative boards or other committees, etc. From the brazilian or international pharmaceutical, orthosis, prosthesis, equipment and implants industry:
	- AstraZeneca: Diabetes; Boehringer Ingelheim: Diabetes, Atrial Fibrillation; Sanofi/Medley: Dyslipidemia; Novartis.
	Other relationships
	Funding of continuing medical education activities, including travel, accommodation and registration in conferences and courses, from the brazilian or international pharmaceutical, orthosis, prosthesis, equipment and implants industry:
	- Sanofi/ Medley: Dyslipidemia.
Juliana Tieko Kato	Nothing to be declared
Luciana Ribeiro Bahia	Financial declaration
	A - Economically relevant payments of any kind made to (i) you, (ii) your spouse/partner or any other person living with you, (iii) any legal person in which any of these is either a direct or indirect controlling owner, business partner, shareholder or participant; any payments received for lectures, lessons, training instruction, compensation, fees paid for participation in advisory boards, investigative boards or other committees, etc. From the brazilian or international pharmaceutical, orthosis, prosthesis, equipment and implants industry:
	- Novo Nordisk: Obesity; AstraZeneca: Diabetes.
	Other relationships
	Funding of continuing medical education activities, including travel, accommodation and registration in conferences and courses, from the brazilian or international pharmaceutical, orthosis, prosthesis, equipment and implants industry:
	- Novo Nordisk: Obesity.
Marcelo Chiara Bertolami	Financial declaration
	A - Economically relevant payments of any kind made to (i) you, (ii) your spouse/partner or any other person living with you, (iii) any legal person in which any of these is either a direct or indirect controlling owner, business partner, shareholder or participant; any payments received for lectures, lessons, training instruction, compensation, fees paid for participation in advisory boards, investigative boards or other committees, etc. From the brazilian or international pharmaceutical, orthosis, prosthesis, equipment and implants industry:
	- Abbott: Lipidil; Sanofi: Zinpass e Zinpass eze, Libbs: Plenance eze.
	Other relationships
	Funding of continuing medical education activities, including travel, accommodation and registration in conferences and courses, from the brazilian or international pharmaceutical, orthosis, prosthesis, equipment and implants industry:
	- Novo Nordisk: Ozempic; EMS: Hipolipemiantes; Aché: Trezate.
Marcelo Heitor Vieira Assad	Financial declaration
	A - Economically relevant payments of any kind made to (i) you, (ii) your spouse/partner or any other person living with you, (iii) any legal person in which any of these is either a direct or indirect controlling owner, business partner, shareholder or participant; any payments received for lectures, lessons, training instruction, compensation, fees paid for participation in advisory boards, investigative boards or other committees, etc. From the brazilian or international pharmaceutical, orthosis, prosthesis, equipment and implants industry:
	- AstraZeneca: Cardiovascular prevention; Boehringer: Diabetes/anticoagulation; Novo Nordisk: Diabetes.
	Other relationships
	Funding of continuing medical education activities, including travel, accommodation and registration in conferences and courses, from the brazilian or international pharmaceutical, orthosis, prosthesis, equipment and implants industry:
	- Boehringer; Novo Nordisk: Diabetes
Marcio Hiroshi Miname	Financial declaration
	A - Economically relevant payments of any kind made to (i) you, (ii) your spouse/partner or any other person living with you, (iii) any legal person in which any of these is either a direct or indirect controlling owner, business partner, shareholder or participant; any payments received for lectures, lessons, training instruction, compensation, fees paid for participation in advisory boards, investigative boards or other committees, etc. From the brazilian or international pharmaceutical, orthosis, prosthesis, equipment and implants industry:
	- Amgen: Repatha; Novo Nordisk: Ozempic; Libbs.
	B - Research funding under your direct/personal responsibility (directed to the department or institution) from the brazilian or international pharmaceutical, orthosis, prosthesis, equipment and implants industry:
	- Kowa: Pemafibrato.
	Other relationships
	Funding of continuing medical education activities, including travel, accommodation and registration in conferences and courses, from the brazilian or international pharmaceutical, orthosis, prosthesis, equipment and implants industry:
	- Novo Nordisk: Ozempic.
Maria Cristina de Oliveira Izar	Financial declaration
	A - Economically relevant payments of any kind made to (i) you, (ii) your spouse/partner or any other person living with you, (iii) any legal person in which any of these is either a direct or indirect controlling owner, business partner, shareholder or participant; any payments received for lectures, lessons, training instruction, compensation, fees paid for participation in advisory boards, investigative boards or other committees, etc. From the brazilian or international pharmaceutical, orthosis, prosthesis, equipment and implants industry:
	- Amgen: Evolocumabe; Amryt: Lomitapide; Aché: Rosuvastatina, Ezetimiba; Libbs.
	B - Research funding under your direct/personal responsibility (directed to the department or institution) from the brazilian or international pharmaceutical, orthosis, prosthesis, equipment and implants industry:
	- Novartis; PTC Bio; Amgen: Dyslipidemia.
	C - Personal research funding paid by the brazilian or international pharmaceutical, orthosis, prosthesis, equipment and implants industry:
	- PTC Bio; Amgen: Dyslipidemia.
Maria Helane Costa Gurgel Castelo	Financial declaration
	A - Economically relevant payments of any kind made to (i) you, (ii) your spouse/partner or any other person living with you, (iii) any legal person in which any of these is either a direct or indirect controlling owner, business partner, shareholder or participant; any payments received for lectures, lessons, training instruction, compensation, fees paid for participation in advisory boards, investigative boards or other committees, etc. From the brazilian or international pharmaceutical, orthosis, prosthesis, equipment and implants industry:
	- PTC: Palestra e Advisory board sobre SQF; Amgen: Estuco clinico nos últimos 2 anos - Houser;
	B - Research funding under your direct/personal responsibility (directed to the department or institution) from the brazilian or international pharmaceutical, orthosis, prosthesis, equipment and implants industry:
	- Amgen: Estudo Houser; Novertis: Estudo Orion.
Maria Sílvia Ferrari Lavrador	Nothing to be declared
Patrícia Guedes de Souza	Nothing to be declared
Raul Dias dos Santos Filho	Financial declaration
	A - Economically relevant payments of any kind made to (i) you, (ii) your spouse/partner or any other person living with you, (iii) any legal person in which any of these is either a direct or indirect controlling owner, business partner, shareholder or participant; any payments received for lectures, lessons, training instruction, compensation, fees paid for participation in advisory boards, investigative boards or other committees, etc. From the brazilian or international pharmaceutical, orthosis, prosthesis, equipment and implants industry:
	- Abbott; Amgen: Dyslipidemia; AstraZeneca: Diabetes; EMS; GETZ Pharma; Kowa; Merck; MSD; Novo Nordisk; Novartis; PTC; Pfizer; Hypera; Sanofi.
	B - Research funding under your direct/personal responsibility (directed to the department or institution) from the brazilian or international pharmaceutical, orthosis, prosthesis, equipment and implants industry:
	- Amgen, Sanofi; Esperion: Dyslipidemia; Kowa.
Renato Jorge Alves	Financial declaration
	A - Economically relevant payments of any kind made to (i) you, (ii) your spouse/partner or any other person living with you, (iii) any legal person in which any of these is either a direct or indirect controlling owner, business partner, shareholder or participant; any payments received for lectures, lessons, training instruction, compensation, fees paid for participation in advisory boards, investigative boards or other committees, etc. From the brazilian or international pharmaceutical, orthosis, prosthesis, equipment and implants industry:
	- Amgen: Evolocumabe; PTC: Volanesorsen; Pfizer: Apixaban.
Roberta Marcondes Machado	Financial declaration
	Nothing to be declared
	Tania L. R. Martinez	Nothing to be declared
	Valeria Arruda Machado	Nothing to be declared
	Viviane Zorzanelli Rocha Giraldez	Financial declaration
		A - Economically relevant payments of any kind made to (i) you, (ii) your spouse/partner or any other person living with you, (iii) any legal person in which any of these is either a direct or indirect controlling owner, business partner, shareholder or participant; any payments received for lectures, lessons, training instruction, compensation, fees paid for participation in advisory boards, investigative boards or other committees, etc. From the brazilian or international pharmaceutical, orthosis, prosthesis, equipment and implants industry:
- Novo Nordisk: GLP-1 Receptor Agonist; AstraZeneca: Dapagliflozina; Amgen: PCSK9 inhibitors.	
Other relationships	
Funding of continuing medical education activities, including travel, accommodation and registration in conferences and courses, from the brazilian or international pharmaceutical, orthosis, prosthesis, equipment and implants industry:	
- Novo Nordisk: GLP-1 Receptor Agonist.	
Wilson Salgado Filho	Nothing to be declared

**Definition of Grades of Recommendation and Levels of Evidence**

**Classes (grades) of recommendation:**

Class I – Conditions for which there is conclusive evidence or, if not, a consensus that the procedure is safe and useful/effective.

Class II – Conditions for which there is conflicting evidence and/or divergence of opinions on the safety and usefulness/efficacy of the procedure.

Class IIA – Evidence or opinion in favor of the procedure. The majority agrees.

Class IIB – Safety and usefulness/efficacy are less well established, and there is no predominance of opinions in favor of the procedure.

Class III – Conditions for which there is evidence and/or a consensus that the procedure is not useful/effective, and in some cases may be harmful.

**Levels of evidence:**

Level A – Data obtained from several large randomized studies showing concurring results and/or a robust meta-analysis of randomized controlled trials.

Level B – Data obtained from a less robust meta-analysis, a single randomized study, or from nonrandomized (observational) studies.

Level C – Data obtained from consensual expert opinions.

## Introduction

Familial hypercholesterolemia (FH) is a common genetic cause of premature coronary artery disease (CAD), especially myocardial infarction, related to lifetime exposure to high concentrations of low-density lipoprotein cholesterol (LDL-C). It is a severe form of genetic dyslipidemia in which approximately 85% of men and 50% of women may experience a coronary event before 65 years of age if not properly treated.

FH is considered a public health problem because of the high prevalence (approximately 1:200-300 in the general population) and the association with early CAD. Reduced life expectancy was also observed in several families. In addition, nearly 200,000 people die each year worldwide from early heart attacks due to the disease, which could be prevented with appropriate treatments. If not treated, men and women with heterozygous FH will develop CAD before the ages of 55 and 60 years, respectively. Homozygotes, in turn, commonly develop CAD very early in life and, if not treated, may die before the age of 20. However, once the diagnosis is made and the treatment is initiated, the natural history of atherosclerotic disease can be changed.

A key step is early diagnosis, which allows early initiation of lipid-lowering medication and may change the natural history of the disease. Diagnosis should be supported by guidelines and algorithms. Identifying more severe cases and promoting integrated care are strategies to minimize the impact of FH on cardiovascular disease (CVD) outcomes. Diagnostic approaches, nutritional measures, and potent drugs, such as high-intensity statins, combined medications, and new lipid-lowering agents, can modify the natural history of the disease in patients.

Another important aspect is the recognition that FH is an autosomal codominant genetic condition, and thus cascade screening of patients’ family members is imperative. This is a cost-effective measure that allows early detection and the initiation of therapies aimed at delaying or preventing the onset of atherosclerotic disease. Special attention is given to children and adolescents, pregnant women, and patients with severe FH in different sections of this guideline.

The Department of Atherosclerosis of the Brazilian Society of Cardiology and Brazil’s leading experts met with the purpose of gathering the best available data and presenting them clearly and objectively to improve clinical practice in Brazil. This guideline can now be used to prevent and treat premature cardiovascular atherosclerotic disease, thus reassuring the families affected by this condition.

Yours sincerely,

Prof. Maria Cristina de Oliveira Izar, MD, PhD

## 1. Natural History of Familial Hypercholesterolemia

### 1.1. Definition

FH is a genetic disease with an autosomal codominant mode of inheritance that affects lipoprotein metabolism. It is characterized by highly elevated levels of LDL-C and specific clinical signs, such as tendon xanthomata, corneal arcus, and atherosclerotic cardiovascular disease (ASCVD) before the age of 45 years. ^1^^,^^2^

The disease was first described by the pathologist Francis Harbitz in the mid-18th century, when he reported cases of sudden death in patients with xanthomas. In 1938, Müller ^3^ described FH as a clinical entity. He identified that the combination of hypercholesterolemia, xanthomas, and CAD manifestations was a common finding in some families, inherited as a dominant trait. Approximately 50 years later, Brown and Goldstein ^4^^-^^6^ elucidated the complex pathway of endogenous cholesterol synthesis and identified a defect in the internalization of receptor-bound low-density lipoprotein (LDL) by studying patients and cell cultures. In 1983, the LDL receptor gene was cloned and mapped on the short arm of chromosome 19 ^7^ , being named the LDL receptor ( *LDLR* ) gene in 1989. ^8^

The estimated historical prevalence of FH was 1:500 people with the heterozygous form and 1:1,000,000 people with the homozygous form. ^9^^,^^10^ Khachadurian ^10^ was the first to discriminate one presentation from the other. However, recent studies based on clinical and molecular criteria suggest that the prevalence of FH is actually higher: 1:200-300 in those with heterozygous FH (HeFH) and 1:160,000-300,000 in those with homozygous FH (HoFH). ^11^^,^^12^

LDL-C plasma concentrations are, in general, 2 to 3 times higher in people with HeFH than in those without the disease, and those with HeFH are more likely to develop premature ASCVD in the second or third decades of life. People with HoFH, in turn, have LDL-C concentrations approximately 6 to 8 times higher and develop ASCVD very early in life, often dying by the age of 20 years if the disease is not treated. ^9^^,^^13^

The clinical phenotype of FH is usually caused by defects in the *LDLR* gene, which encodes the LDLR, ^5^^,^^10^ with more than 2,251 mutations described to date. ^14^ Point mutations, or single base-pair substitutions (single nucleotide polymorphism, SNP), account for over 84% of mutations, and major rearrangements occur in 16% of all mutations described in the *LDLR* gene.

The clinical phenotype of FH may also be secondary to defects in the apolipoprotein-B ( *APOB* ) gene, which encodes apolipoprotein B-100 (Apo B-100) ^15^ – when defective, it has lower affinity for the LDLR. It may also occur in case of accelerated LDLR catabolism due to gain-of-function mutations in the proprotein convertase subtilisin/kexin type 9 ( *PCSK9* ) gene, which encodes NARC-1, ^16^ a protein involved in LDLR catabolism.

In most cases, FH is caused by mutations in genes encoding proteins involved in LDLR uptake and catabolism. The *LDLR* , *APOB* , and *PCSK9* genes are linked to the development of FH, causing defective homeostasis of LDL particles and, consequently, elevated plasma concentrations of LDL-C. Thus, patients with a molecular diagnosis of FH frequently have pathogenic variants in the *LDLR* gene, ^17^ while mutations in the *APOB* and *PCSK9* genes account for a lower proportion of cases of FH in its autosomal codominant form. ^18^ Autosomal recessive FH (ARH), conversely, is rare and occurs when pathogenic mutations are inherited in both copies of the low-density lipoprotein adaptor protein 1 ( *LDLRAP1* ) gene, which encodes the LDLR adaptor protein. ^19^ However, FH may also be caused by pathogenic mutations in unidentified genes or in several genes, known as polygenic FH. ^20^

The clinical phenotype is remarkably similar among the most common forms of FH; however, *APOB* gene defects are more common among some European populations (1:300-700 in Central Europe), while *PCSK9* gene mutations do not have an established frequency (generally ~1%). FH has high penetrance and, ^20^^-^^22^ therefore, most carriers of FH-causing mutations have the clinical phenotype. Because of its autosomal codominant mode of inheritance, half of the first-degree relatives of a person with FH will carry the genetic defect and show high LDL-C levels at birth and over the lifespan. Men and women are equally affected. ^9^^,^^22^

In heterozygotes, only half of the LDLRs are functional, while in homozygotes, due to a defect in the *LDLR* , both receptors have loss of function or null function. ^23^ Genetic diagnosis is important because patients are often unaware of clinical/laboratory criteria, which makes diagnostic confirmation more difficult.

According to a recent statement, ^23^ FH comprises multiple clinical phenotypes because of different underlying molecular etiologies and additional genetic factors. LDL-C levels, number of mutations, and additional protective or pathogenic factors determine the risk of CAD; therefore, people at risk due to family history and those with the FH phenotype should be genotyped. The test results indicate three categories of individuals:

1. Genotype-positive, phenotype-negative
2. Genotype-positive, phenotype-positive
3. Genotype-negative, phenotype-positive

In some cases, alternative molecular etiologies should be explored, ^23^ such as mutations in the *APOE* gene or in the *LIPA* gene, which encodes lysosomal acid lipase, as well as the polygenic form. Carriers of pathogenic mutations are at higher risk of CAD in any LDL-C level when compared to noncarriers. Those with causative mutations and LDL-C levels > 190 mg/dL have a 3-fold higher risk of CAD compared with noncarriers with the same LDL-C levels. This is probably due to lifetime exposure to very high levels of LDL-C. ^24^

FH is considered a public health problem because of the high prevalence of early coronary heart disease and reduced life expectancy observed in several families. If the disease is not properly treated, approximately 85% of men and 50% of women may experience a coronary event before the age of 65 years. Studies have shown that approximately 200,000 people die each year worldwide from early heart attacks due to FH, which could be prevented with appropriate treatments. ^20^

### 1.2. Epidemiology of Atherosclerotic Cardiovascular Disease

ASCVD and its complications are a serious public health problem in Brazil and worldwide. According to data from the Information Technology Department of the Brazilian Unified Health System (DATASUS), CVDs are the leading cause of death in the country, accounting for approximately 27.65% of all deaths. ^25^ Regarding the specific mortality rate for circulatory system diseases, ischemic heart diseases account for 32% of deaths. ^25^ According to Ribeiro et al., ^26^ the Brazilian public health system funded 940,323 hospitalizations for CVD in 2012. From 2008 to 2012, the rates of hospitalization for congestive heart failure and hypertension decreased, whereas the rates of hospitalization for angioplasty and acute myocardial infarction (AMI) increased. ^26^

Ischemic heart diseases and stroke are, respectively, the first and second leading causes of death worldwide, accounting for more than 15.2 million deaths. These conditions have remained the global leading causes of death for the past 15 years. ^27^ A study conducted in the United States (US) from 1989 to 1998 found that 51% of women and 41% of men with sudden cardiac death died out of hospital. Acute coronary syndrome (ACS) accounted for 27% of these deaths. ^28^

Most deaths from AMI occur in the first hours of manifestations. Forty to 65% of cases occur in the first hour, while approximately 80% occur in the first 24 hours. ^29^ Among survivors, 19% on average progress to heart failure, which is a major cause of hospitalization and morbidity. ^30^^,^^31^

Although known cardiovascular risk factors are responsible for most cases of ASCVD and its complications, ^32^^-^^36^ some clinical conditions increase the risk of ASCVD and accelerate its onset, such as FH. ^37^^-^^40^

### 1.3. Epidemiological Aspects of Familial Hypercholesterolemia Worldwide and In Brazil

The estimated historical prevalence of FH in the general population is 1:500. ^22^ However, according to the Copenhagen General Population Study, the estimated current prevalence of FH is 1:223 by clinical criteria ^37^ and 1:217 by genetic testing. ^38^ A Danish government report concluded that, with a prevalence of 1:200-250, only 11 to 13% of HeFH carriers would be identified (failure to diagnose is particularly significant in children).

The estimated prevalence of HoFH was 1:1,000,000. However, current data suggest that HoFH affects 1:300,000 people, although the number can be higher (1:160,000) when there is a founder effect. This means that HoFH may be more prevalent in some populations, such as South Africans (1:100,000), Lebanese (1:170,000), French Canadians (1:270,000), and Finns, because of consanguineous marriage. ^13^^,^^14^

Therefore, according to the Danish study, an HeFH prevalence of 1:220 translates into an allele frequency of 1:440, with an assumed HoFH frequency of 1:193,600. Based on these estimates, the predicted number of cases of HoFH in Denmark is approximately 28. However, the fact is that very few cases are diagnosed ^38^^,^^39^ and, in most countries, the condition remains underdiagnosed (less than 1% in Brazil). ^14^ The estimated number of people with FH worldwide is over 34,000,000. ^9^^,^^14^ However, less than 10% are diagnosed, and less than 25% receive lipid-lowering medication. ^38^ If the same prevalence is assumed in Brazil, approximately 1,033 cases of HoFH are estimated in the country.

There are no objective data on the prevalence of FH in Brazil. Based on clinical and laboratory data and family history of the adult population of participating institutions in the ELSA-Brazil study and according to the Dutch Lipid Clinic Network (DLCN) criteria, the estimated prevalence of FH is 1:263, which corresponds to 766,000 people living with FH in Brazil. ^41^ The prevalence varies according to gender (0.38% in women and 0.30% in men), race (0.25% in White people, 0.47% in multiethnic people, and 0.67% in Black people), and age (0.10% in 35-45 years, 0.42% in 46-55 years, 0.60% in 56-65 years, and 0.26% in 66-75 years). ^41^ Data from a recent meta-analysis showed that the global prevalence of FH in the general population is 1:311, being 18-fold higher among those with ASCVD. ^42^ Another meta-analysis showed a higher prevalence of FH among those with ischemic heart disease (10-fold), premature ischemic heart disease (20-fold), and severe hypercholesterolemia (23-fold). ^43^

### 1.4. Impact of Familial Hypercholesterolemia on Atherosclerotic Cardiovascular Disease

FH is a common genetic cause of premature coronary disease, especially AMI and angina pectoris, related to lifetime exposure to elevated LDL-C concentrations. ^44^^,^^45^ If not treated, men and women with HeFH and total cholesterol levels between 310 and 580 mg/dL will develop CAD before the ages of 55 and 60 years, respectively. Homozygotes with total cholesterol levels between 460 and 1,160 mg/dL usually develop CAD very early in life and, if not treated, may die before the age of 20 years. However, when the diagnosis is made and the treatment is initiated, the natural history of atherosclerotic disease can be changed. ^46^

Although there are no data on the risks of ASCVD or the rates of lipid-lowering treatment in FH, the prevalence of CAD among those with a probable or definite diagnosis of FH (according to DLCN criteria) in a large sample of the general population in Copenhagen, Denmark, was 33%, ^37^ of which only 48% received statins. Those with probable or definite FH who did not receive statins had a 13-fold increase in the risk of CAD (95% confidence interval [CI] 10- to 17-fold). Similar results were found in other FH cohorts. ^47^

Conversely, FH patients receiving statins had a 10-fold higher risk of ASCVD (95% CI 8- to 14-fold), which suggests that the statin treatment was insufficient to lower lipid levels or was introduced late in life, when atherosclerosis was already severe. Similar treatment data were reported in other studies. ^48^^-^^50^

The risk of premature ASCVD in FH is very high, and 5 to 10% of coronary events occur before the age of 50 years. ^47^^,^^51^ If not treated, young patients with FH have a 90-fold increase in the risk of death. ^47^^,^^51^ FH also accounts for a significant number of hospitalizations and loss of productivity due to the high incidence of ASCVD. ^47^

Therefore, a key step is early diagnosis, which allows early initiation of lipid-lowering medication and may change the natural history of the disease. Diagnosis should be supported by guidelines ^52^^-^^54^ and algorithms. ^55^ In addition, identifying more severe cases ^56^^,^^57^ and promoting integrated care ^58^ are strategies to minimize the impact of FH on CVD outcomes.

## 2. Lipid Metabolism in Familial Hypercholesterolemia

The amount of circulating cholesterol depends, on the one hand, on the balance between hepatic synthesis and intestinal absorption and, on the other hand, on excretion, especially via biliary tract. When this process is unbalanced, as is the case with FH, cholesterol levels can increase significantly and form deposits such as xanthomas and early atherosclerosis ^22^ . Body cholesterol input and output are regulated by a feedback system in which increased cholesterol absorption from diet leads to reduced hepatic synthesis. Unlike dietary fats, which are almost completely absorbed by the intestine, cholesterol is only partially absorbed. When the amount of cholesterol from diet increases, absorption decreases proportionally. In men, cholesterol is mostly transported by LDLs. These particles are produced by metabolism of very low-density lipoproteins (VLDLs), which are rich in triglycerides but also provide, especially as remnants (intermediate-density lipoproteins, IDLs), cholesterol for the formation of atheromatous plaques. In addition, when LDLs are delipidated in triglyceride content, they originate smaller and denser LDLs, which are very atherogenic. LDLs are removed from circulation and transported into cells by cell membrane receptors that recognize Apo B-100, the only protein found in LDL. Remnants and IDL are also removed by these receptors, but much faster than LDL. This happens because these particles, in addition to Apo B-100, also have apolipoprotein E (Apo E) on their surface, which has higher affinity for receptors than Apo B-100.

In FH, genetic defects also affect the LDLR, leading to decreased lipoprotein endocytosis. ^59^ Receptor-mediated LDL endocytosis and defects causing impaired receptor function and hypercholesterolemia were described by Brown and Goldstein in the 1970s. The several hundred polymorphisms in the receptor gene can affect the structure of the receptor that binds to Apo B-100 in LDL particle, other protein domains, and the recirculation of receptors that are normally recycled back to the cell membrane after endocytosis. However, only some *LDLR* gene polymorphisms are associated with the FH phenotype. Apo B defects and those related to gain-of-function of PCSK9, participating in LDLR catabolism, account for approximately 5% and < 1% of cases, respectively. ^2^

A much rarer possibility is a homozygous defect in the *LDLRAP1* , since this type of defect is recessive. However, according to estimates, between 5 and 30% of patients with the FH phenotype do not have an identified causative gene, which suggests that there were mutations in unidentified genes or by combination (polygenic mutations). Thus, FH results from the inability to efficiently remove cholesterol from LDLs, causing elevated plasma concentrations and deposits in vessels and tissues. ^59^

In general, FH is genetically transmitted by one of the parents, with an autosomal codominant monogenic inheritance pattern, which most frequently characterizes the heterozygous form of the disease. The estimated prevalence of HeFH is 1:200-250 in Europe and approximately 1:250 in Brazil. However, a concomitant increase in Lp(a) or a concomitant defect in triglyceride metabolism is not uncommon, indicating even more severe dyslipidemia.

The occurrence of xanthomas during childhood or adolescence together with very high LDL-C levels (> 500 mg/dL), premature atherosclerotic disease, and aortic valve stenosis are suggestive of HoFH, which is much more severe and difficult to treat. ^13^ In this case, most patients’ parents have HeFH, usually due to mutations in the *LDLR* gene, but mutations may also occur in other genes ( *APOB* or *PCSK9* ). There may also be a combination of polymorphisms from different genes ( *LDLR* , *APOB* , *PCSK9* , or *LDLRAP-1* ). In the homozygous form, concomitantly low levels of high-density lipoprotein cholesterol (HDL-C) are also frequent, possibly due to accelerated removal of apolipoprotein A1 (Apo A-I) or a defect in cholesterol efflux. Homozygous manifestations should also be suspected in case of less markedly elevated LDL-C levels (> 300 mg/dL) occurring together with xanthomas before 10 years of age. ^13^

## 3. Clinical Diagnosis of Familial Hypercholesterolemia

The clinical and laboratory criteria for the diagnosis of FH are arbitrary and based on the following:

- Clinical signs of extravascular cholesterol deposits
- Elevated plasma LDL-C or total cholesterol levels
- Family history of hypercholesterolemia and/or premature atherosclerotic disease
- Identification of mutations and genetic polymorphisms favoring the development of FH.

Some criteria have been proposed to standardize and formalize the diagnosis of FH, such as the US MEDPED criteria from the US Make Early Diagnosis to Prevent Early Deaths Program, ^60^ the Dutch MEDPED criteria from the DLCN (see Table 1 ), ^61^ and the Simon Broome Register Group criteria. ^62^ The Dutch MEDPED criteria are used in Brazil.

**Table 1:** Diagnostic criteria for heterozygous familial hypercholesterolemia according to the Dutch Lipid Clinic Network (Dutch MEDPED criteria) ^61^

Parameter	Score
**Family history**	
First-degree relative with premature coronary and vascular disease (men aged < 55 years, women aged < 60 years) OR Adult relative with total cholesterol > 290 mg/dL *	1
First-degree relative with tendon xanthomas and/or corneal arcus OR First-degree relative aged < 16 years with total cholesterol > 260 mg/dL *	2
**Clinical history**	
Patient with premature CAD (men aged < 55 years, women aged < 60 years)	2
Patient with premature cerebral or peripheral vascular disease (men aged < 55 years, women aged < 60 years)	1
**Physical examination**	
Tendon xanthomas	6
Corneal arcus < 45 years of age	4
**LDL cholesterol levels (mg/dL)**	
≥ 330	8
250 to 329	5
190 to 249	3
155 to 189	1
**DNA analysis**	
Presence of functional mutation in the LDL receptor gene, Apo B-100, or PCSK9 *	8
**FH diagnosis**	
Definite if	> 8
Probable if	6 to 8
Possible if	3 to 5

* Adapted from the Dutch Lipid Clinic Network with one criterion from the Simon Broome Register Group. ^62^ CAD: coronary artery disease; DNA: deoxyribonucleic acid; FH: familial hypercholesterolemia; LDL: low-density lipoprotein.

The Dutch MEDPED criteria are simple and recommended for diagnostic suspicion of FH and for decision-making regarding treatment initiation (see below). An algorithm based on the Dutch MEDPED criteria can be used to improve diagnostic accuracy, although validation for the Brazilian population is not yet available.

### 3.1. Medical History

Given the high prevalence of FH in the general population and its great impact on CVD and mortality rates, medical history should include information about family history of hypercholesterolemia, use of lipid-lowering medication, and premature atherosclerotic disease, including the age of onset. Patients with a family history of FH and/or premature atherosclerotic disease are more likely to have FH.

### 3.2. Physical Examination

The investigation for clinical signs of FH (xanthomas, xanthelasmas, and corneal arcus) should be part of routine physical examination and can be complemented by additional tests, such as tendon ultrasound, in specific cases. The clinical signs are not overly sensitive but can be highly specific; thus, although FH diagnosis does not depend on clinical signs, their presence strongly suggests this etiology.

Tendon xanthomas ( Figure 1 ) are most commonly found in the Achilles tendon and in the extensor tendons of the fingers but may also be found in the patellar and triceps tendons. They should be assessed by both visual inspection and palpation. These xanthomas are almost pathognomonic for FH but occur in less than 50% of cases. ^63^ Intertriginous planar xanthomas may also occur, especially in HoFH ( Figure 2 ).

**Figure 1:** Tendon xanthoma in the Achilles tendon.

**Figure 2:** Planar xanthoma.

Yellow-orange tuberous xanthomas ( Figures 3 and 4 ) and eyelid xanthelasmas are not specific to FH and should be carefully assessed when found in patients aged 20 to 25 years. The presence of partial or total corneal arcus suggests FH when found in patients under 45 years of age ( Figure 5 ). HoFH carriers may also have systolic ejection murmur due to stenosis in the aortic valve and supra-aortic region.

**Figure 3 (A e B):** Tuberous xanthomas on the knees.

**Figure 4:** Tuberous xanthomas on the hands.

**Figure 5:** Corneal arcus.

### 3.3. Screening and Lipid Levels

The collection of blood samples to measure LDL-C and total cholesterol levels for FH screening is extremely important to diagnose as many cases as possible and, consequently, reduce the impact of the disease on cardiovascular morbidity and mortality in the general population. The screening process can be based on two methods: universal screening and cascade screening. ^23^^,^^52^

#### 3.3.1. Universal Screening

All those aged 10 years or over should undergo lipid profile testing. ^52^ Plasma lipid measurement should also be considered in children aged 2 years or over in the following cases: ^52^

1. When there is a family history of premature atherosclerotic disease (men aged < 55 years or women aged < 65 years) and/or dyslipidemia.
2. When the child has xanthomas or corneal arcus, risk factors (hypertension, diabetes mellitus, obesity), or atherosclerotic disease.

The recommended frequency for plasma lipid measurement is still open to debate. In general, if the lipid profile is normal, but other criteria suggest FH, such as family history of early atherosclerotic disease or significant hypercholesterolemia, the test can be repeated after 1 year. In the absence of these factors, the test can be repeated within 5 years. Data such as age, presence of other risk factors for atherosclerosis, degree of control of risk factors, lifestyle habits, and occasional use of medications that may affect lipid metabolism can be considered to individualize the frequency of lipid measurements.

A positive FH diagnosis should always be suspected in adults (≥ 20 years) with LDL-C levels ≥ 190 mg/dL. In the general population, people aged ≥ 30 years with LDL-C ≥ 250 mg/dL, those aged 20-29 years with LDL-C ≥ 220 mg/dL, and those aged < 20 years with LDL-C ≥ 190 mg/dL are 80% more likely to have FH. ^61^ FH is also more likely to occur in patients with LDL-C ≥ 190 mg/dL whose families are characterized by a bimodal distribution of LDL-C, in which some members have typically low levels (LDL-C < 130 mg/dL), while others (those with FH) have typically high levels (≥ 190 mg/dL). ^62^

Before the diagnosis of FH is made, however, secondary causes of hypercholesterolemia, including hypothyroidism and nephrotic syndrome, should be ruled out. Importantly, the presence of hypertriglyceridemia does not exclude the diagnosis of FH.

Since 2017, total cholesterol levels ≥ 310 mg/dL in adults and ≥ 230 mg/dL in children and adolescents have been considered suggestive of FH by laboratory reports in Brazil. ^64^

Finally, it should be noted that lipid profile measurement is subject to a series of variations related to the method and procedures used as well as to patient-specific factors, such as lifestyle, use of medications, and associated diseases. Therefore, diagnostic accuracy can be increased by laboratory analysis of new samples ideally collected at least 1 week after the first collection.

#### 3.3.2. Cascade Screening

Cascade screening consists of measuring the lipid profiles of all first-degree relatives (father, mother, and siblings) of patients diagnosed with FH. The chances of identifying other FH carriers from an index case are 50% in first-degree relatives, 25% in second-degree relatives, and 12.5% in third-degree relatives. ^63^ As new cases are identified, additional relatives are recommended for screening. Cascade screening is considered the most cost-effective method for identifying FH carriers.

##### 3.3.2.1. Cascade Genetic Screening

Genetic screening is cost-effective and can be used in all FH patients and their first-degree relatives. The most cost-effective cascade screening method is the one that uses genetic information from people who have been identified with an FH-causing mutation. ^63^

##### 3.3.2.2. Reverse Cascade Screening

Reverse cascade screening consists of reversely testing first-, second-, and third-degree relatives of a child who has been identified as an index case. Children with FH are often the first to be diagnosed by a pediatrician, and their parents are unaware if they are also FH carriers. Therefore, it is an opportunity to identify and treat asymptomatic parents who have never used any medication for the disease. ^23^

##### 3.3.2.3. Opportunistic Diagnosis

Opportunistic diagnosis occurs when the lipid profile is measured at the time of immunization. Although this is not a common practice in Brazil, the method is an opportunity for early diagnosis of asymptomatic children. ^23^^,^^59^

#### 3.3.3. Homozygous Familial Hypercholesterolemia

The estimated historical prevalence of HoFH in the general population worldwide (1:1,000,000) is very low. However, higher prevalence rates are currently recorded in the general population, ranging from 1:160,000 to 1:300,000. ^13^^,^^52^ The diagnostic criteria for HoFH are shown in Chart 1 .

**Chart 1:** Diagnostic criteria for homozygous familial hypercholesterolemia (HoFH).

1. Genetic confirmation of two mutant alleles at the *LDLR* , *APOB* , and *PCSK9* genes or at the *LDLRAP1* gene locus OR
2. Untreated LDL-C > 500 mg/dL or treated LDL-C > 300 mg/dL together with:
either cutaneous or tendon xanthomas before the age of 10 years OR elevated LDL-C levels consistent with heterozygous FH in both parents *

* Except for autosomal recessive hypercholesterolemia. FH: familial hypercholesterolemia; LDL-C: low-density lipoprotein cholesterol. The LDL-C levels are only suggestive of homozygous FH, but lower levels should be considered for the diagnosis of compound or double heterozygotes in the presence of additional criteria.

### 3.4. Recommendations

1. Clinical signs of FH and family history of early atherosclerotic disease and/or dyslipidemia should be investigated in all patients (grade of recommendation: I; level of evidence: C).
2. The lipid profile of all patients over 10 years of age should be measured (grade of recommendation: I; level of evidence: C).
3. Lipid profile measurement should be considered in those aged 2 years or over in the presence of risk factors, clinical signs of FH, and atherosclerotic disease, as well as in the case of a family history of premature atherosclerotic disease and/or dyslipidemia (grade of recommendation: I; level of evidence: C).
4. The lipid profile of all first-degree relatives of patients diagnosed with FH should be measured (grade of recommendation: I; level of evidence: C).

## 4. Genetic Testing for Familial Hypercholesterolemia

FH is an autosomal codominant disease. It is primarily caused by loss-of-function mutations in the *LDLR* and *APOB* and, less frequently, by gain-of-function mutations in *PCSK9* , which is responsible for LDLR degradation.

### 4.1. LDLR, APOB, PCSK9, and Removal of Circulating LDL

The LDLR is located on the surface of hepatocytes and cells from other organs, binding to LDL via Apo B, which leads to LDLR uptake by a mechanism of internalization and endocytosis of the LDL/Apo B/LDLR complex. This process is mediated by LDLRAP1 present in the clathrin-coated pits. After internalization, the LDL particle separates from the LDLR in the endosome, and the LDLR will either undergo lysosomal degradation facilitated by PCSK9 or be transferred back to the cell surface. Cholesterol is then released into the cell for metabolism or elimination. Alternatively, the LDLR can be degraded by exogenous PCSK9 binding to the LDLR on the cell surface, where it is internalized and processed for lysosomal degradation. ^16^ When the LDLRs have any genetic mutation affecting their structure or function, the level of LDL removal from the plasma decreases and, consequently, the plasma LDL-C level increases inversely to the number of functional receptors. ^65^

### 4.2. Autosomal Dominant Inheritance

FH is classically caused by pathogenic mutations in the *LDLR* , *APOB* , and *PCSK9* genes. The LDLR-coding gene ( *LDLR* ), located on chromosome 19, consists of approximately 45,000 DNA base pairs and is formed by 18 exons and 17 introns. The LDLR is a protein composed of 839 amino acids, including a 21-amino-acid signal peptide with several functional domains.

The analysis of the mutations identified in the *LDLR* gene demonstrates that there are no mutation hot spots in the gene. ^66^^,^^67^ Nonetheless, mutations in exon 4, which is responsible for binding LDLR via Apo B, seem to correlate with more severe phenotypes. ^66^^-^^70^ Interestingly, de novo mutations in the *LDLR* gene seem to be rare. ^71^ Production is finely regulated by a sophisticated feedback mechanism controlling the transcription of the *LDLR* gene in response to variations in intracellular sterol content and cellular cholesterol demand. ^72^

There are approximately 2,900 genetic variants associated with FH, ^73^ and approximately 85 to 90% occur in the *LDLR* gene. FH is most commonly attributed to mutations in the *LDLR* gene (including missense, nonsense, insertions, and deletions), resulting in partial-to-total functional reductions in LDLR ability to remove LDL from circulation. Depending on the impact of the mutation on the resulting protein, the patient may express little or no LDLR (receptor-negative) or LDLR isoforms with reduced affinity for LDL on the surface of hepatocytes (receptor-defective). ^70^^,^^74^^-^^77^

Heterozygotes inherit an allele with a pathogenic variant from one parent and a normal allele from the other. Since two functional alleles are needed to maintain a normal plasma LDL-C level, the absence of a functional allele may cause a 2-fold increase from normal LDL levels during childhood. ^72^ Homozygotes inherit two alleles with pathogenic variants; consequently, the LDLR function is very reduced, and patients develop very severe hypercholesterolemia (400 to 1,000 mg/dL). ^72^

There are five main classes of *LDLR* gene mutations: ^70^^,^^76^

- Class I (null mutations): these mutations affect the promoter or coding regions of the gene, leading to the complete absence of LDLR synthesis or in the synthesis of a nonfunctional receptor.
- Class II: these mutations are caused by defects in post-translational processing or failure to transport LDLR from the endoplasmic reticulum to the Golgi complex, leading to lower LDLR expression on the cell surface.
- Class III: LDL does not bind correctly to the LDLR on the cell surface because of a defect in the ligand-binding domain or in the epidermal growth factor (EGF) precursor homologous domain of the LDLR.
- Class IV: the LDLR binds normally to LDL, but the latter is not efficiently internalized by the mechanism of endocytosis via clathrin-coated pits.
- Class V: the LDLR is not recycled back to the cell surface.

The *APOB* gene spans 42 kilobases (kb), consists of 29 exons and 28 introns, and yields two protein isoforms: a small one, named Apo B-48, and a large one, named Apo B-100. Apo B-48 is produced in the intestine and found in chylomicrons and their remnants, while Apo B-100 is produced in the liver and found in several lipoproteins, such as VLDL, IDL, LDL, and lipoprotein(a) (Lp(a)). Hypercholesterolemia due to a mutation in the *APOB* gene results in a clinical phenotype of FH similar to that caused by mutations in other genes and was classically referred to as familial defective Apo B-100 (FDB). ^15^ However, it is worth noting that FDB is currently considered a type of FH, and its distinction is made only from an academic perspective.

Contrasting with the findings regarding the *LDLR* gene, only 353 variants have been identified in the *APOB* gene, ^80^ and most of them are in exon 26. ^78^^-^^80^ The most common mutation in the *APOB* gene is the Arg3500Gln substitution, which causes disruption of the protein structure. The variant accounts for 5 to 10% of FH cases in northern European populations but is rare in other populations. ^79^^,^^80^

Another condition that may cause the FH phenotype is increased PCSK9 activity, also known as FH3, in which gain-of-function mutations lead to further LDLR degradation. ^16^^,^^80^^,^^81^ This is the least common cause of FH, accounting for 1 to 3% of clinically diagnosed cases. ^80^^,^^81^ The *PCSK9* gene spans 25 kb, consists of 12 exons, and yields a 692-amino acid protein.

### 4.3. Autosomal Recessive Hypercholesterolemia

In addition to the previously described genes, mutations in *LDLRAP1* have also been considered to cause the HoFH phenotype. Unlike classic FH, these disorders have an autosomal recessive mode of inheritance. In ARH, reduced LDLRAP1 expression hinders LDLR binding to clathrin-coated pits on the cell surface, ^82^^,^^83^ which thus reduces or prevents the internalization of the LDL/LDLR complex in the hepatocytes. The *LDLRAP1* gene spans 25 kb, consists of 9 exons, and yields a 308-amino acid protein. Only patients with homozygous or compound heterozygous gene mutations are affected; simple heterozygotes are only carriers, as they usually do not have hypercholesterolemia. However, cases of ARH carriers with LDL-C levels higher than other noncarrier family members have been described in the literature. ^84^

### 4.4. Other Candidate Genes

In addition to the previously mentioned genes, there are other candidate FH-causing genes: *APOE* , *IDOL (MYLIP)* , *HCHOLA4* , *STAP1* , and *LIPA* . ^85^

Rare forms of ARH (also known as FH phenocopies) include sitosterolemia and phytosterolemia, which occur due to mutations in two adjacent and oppositely oriented genes ( *ABCG5* and *ABCG8* ). These genes encode ATP-binding cassette transporter proteins, sterolin-1 and sterolin-2, ^86^ which are involved in the elimination of plant sterols, which cannot be used by human cells, and in cholesterol 7-alpha hydroxylase (CYP7A1) deficiency. CYP7A1 is an enzyme involved in the first step in bile acid synthesis, and its deficiency results in increased intrahepatic cholesterol and reduced LDLR expression on the surface of the hepatocyte. CYP7A1 deficiency is the least common autosomal recessive disorder possibly causing severe hypercholesterolemia. ^87^

### 4.5. Phenotype Variability in Familial Hypercholesterolemia

Current studies show that FH encompasses a spectrum of clinical phenotypes partly based on the range of pathogenic variants. Therefore, people with more than one variant in the same gene and in different alleles (compound heterozygotes in *trans* , usually in the *LDLR* gene) may have a phenotype similar to that of a true homozygote (same variant in two alleles). ^23^^,^^88^ Table 2 shows the variability in the distribution of pre-treatment LDL-C levels for different FH genotypes. ^23^

**Table 2:** Variability in familial hypercholesterolemia phenotype in descending order of LDL-C concentrations

LDL-C levels	Possible genotypes
400 to 1,000 mg/dL	Homozygous pathogenic variants
	Null homozygous for *LDLR*
	True homozygous for *LDLR*
	Compound heterozygous for *LDLR*
130 to 450 mg/dL	Heterozygous pathogenic variants
	Null *LDLR*
	Defective *LDLR*
	Gain-of-function *PCSK9*
	APOB
	Polygenic forms (multiple LDL-C-raising SNPs)
	High lipoprotein(a)
130 to 200 mg/dL	Common hypercholesterolemia

Adapted from Sturm et al. ^23^ APOB: apolipoprotein B; LDL-C: low-density lipoprotein cholesterol; LDLR: LDL receptor; PCSK9: proprotein convertase subtilisin/kexin type 9; SNP: single nucleotide polymorphism.

Importantly, normal LDL-C levels have been described in patients with pathogenic variants in families with FH, and a pathogenic variant is not always identified in people with the phenotype. Thus, the presence of a phenotype compatible with FH without an identified pathogenic variant in the classical *LDLR* , *APOB* , and *PCSK9* genes may be linked to polygenic inheritance. Talmud et al. ^89^ described sets of 12 polymorphisms in different genes in hypercholesterolemic patients without an identified causative mutation. ^89^ According to the authors, in the absence of classical monogenic causes, polygenic inheritance could explain up to 88% of cases of general hypercholesterolemia and approximately 20% of those with the FH phenotype. ^90^

### 4.6. Rationale for Cascade Screening

Cascade genetic screening has been used as a tool to identify new FH patients. Depending on the inclusion criteria and the sensitivity of the methods used for FH screening, pathogenic variants correlated to the disease can be identified in 30 to 80% of patients. ^91^^,^^92^ The technique consists of sequencing variants in first-degree relatives of those diagnosed with FH. ^93^ In screening rounds, first-degree relatives identified with the condition become index cases, and their respective relatives are screened successively ( Figure 6 ).

**Figure 6:** Example of cascade genetic screening.

Cascade genetic screening is the most cost-effective strategy for identifying FH carriers. ^93^^-^^95^ Marks et al. ^93^ analyzed the cost-effectiveness of the strategy and determined an incremental cost per life-years gained (LYG) of £3,300. It was the most cost-effective program in Denmark, with a cost per LYG of US$8,700.00, which demonstrates a lower cost estimate than the expenditures on secondary prevention of non-FH carriers. ^96^

Studies have shown that very few FH carriers are diagnosed. In general, according to estimates, approximately 20% of patients are diagnosed, and less than 10% receive appropriate treatment. ^10^ In view of that, cascade screening increases the number of diagnoses and decreases the age at diagnosis, thereby providing a greater chance of early treatment and reduction in global cardiovascular risk for patients.

Genetic testing is not usually necessary for the diagnosis or clinical treatment of an index case, but it can be useful when diagnosis is uncertain or when family members need to be assessed. The cascade screening method has been used in several countries, including Spain, ^96^ England, Netherlands, ^97^ Portugal, ^98^ and more recently in Brazil, ^99^ as a highly cost-effective tool for identifying new FH carriers.

A genetics consortium study ^24^ identified the presence of a FH-causing pathogenic variant in 2% of severe cases (LDL-C > 190 mg/dL detected in approximately 7% of the study population). Participants with the FH-causing monogenic variant had a 22-fold higher risk of cardiovascular events compared with normolipidemic participants without genetic mutations and a 4-fold higher risk compared with hypercholesterolemic participants without mutations. ^24^ The increase was mainly due to the exposure of FH patients to high cholesterol levels from birth, unlike polygenic hypercholesterolemia, which may manifest later in life. These data strongly suggest that the presence of a FH-causing pathogenic genetic variant has prognostic implications.

The identification of a causative mutation may provide additional motivation for some patients to initiate appropriate treatment, and genetic testing is the gold standard for a definite diagnosis of FH. It can be particularly useful in cases of family members with a misdiagnosis or with LDL-C level as the only indicator of the disease. Genetic testing may also be important to identify a causative mutation in families with a strong suspicion or recent diagnosis of FH. Furthermore, once a mutation is identified, the test provides a simple and definite answer for the diagnosis of FH, becoming an indisputable tool for identifying the disease as a family trait. ^23^

However, genetic testing has limitations. Among hypercholesterolemic patients with a possible FH diagnosis, the rate of identification of a causative mutation by genetic testing is 50% or less, while the rate of identification in patients with a definite FH diagnosis according to clinical criteria may be as high as 86%. ^23^^,^^100^ Therefore, a negative genetic test does not exclude FH diagnosis. Also, people with elevated LDL-C levels remain at high cardiovascular risk and should be treated according to established guidelines regardless of genetic test results.

### 4.7. Methods for Genetic Diagnosis

Defects in the genes causing the FH phenotype – *LDLR* , *APOB* , *PCSK9* , or *LDLRAP1* , in addition to the rarer ones previously mentioned – cannot be clinically detected, thus genetic testing is required for confirmation. Thus, because of gene variability and the large number of possible mutations, the method of genetic diagnosis should include the sequencing of the coding region of all genes possibly linked to the etiology of the disease. ^101^

To achieve large-scale sequencing, so that a group of genes can be sequenced (targeted gene panels), next-generation sequencing (NGS) should be used. In this technique, a panel is designed with all the genes to be sequenced, which are placed on a chip. A broader approach is exome sequencing, which allows the determination of the coding region sequence of virtually every gene in the genome in question. However, although this approach provides extensive coverage of the genome, many genes may not be perfectly sequenced. Therefore, in specific cases of monogenetic diseases, such as FH, targeted gene panels are a more cost-effective alternative in addition to being more accurate.

NGS technology has many advantages over Sanger sequencing, which is considered the gold-standard sequencing method. Some of these advantages include the promptness of sequencing results, the amount of material required for the reaction, the cost of per-base sequencing, the amount of output data, and the accuracy of sequencing results. Briefly, for genetic testing, peripheral blood is collected in a tube containing ethylenediaminetetraacetic acid (EDTA), and the genomic DNA of leukocytes is extracted. The first step in preparing the material consists of generating a library of DNA fragments flanked by specific adaptors. The regions of interest of the genes under study are amplified by large-scale polymerase chain reaction in multiplex reactions, with hundreds of pairs of oligonucleotides in the same reaction tube. From these reactions, libraries with barcodes are created to identify each patient analyzed. The generated fragments are clonally amplified on beads by polymerase chain reaction, and the beads are then placed on a chip and inserted in the NGS device. Once generated, the data are transferred to a platform, in which the readings are mapped to the human genome (hg19/GRCh37) and the variants are interpreted.

Approximately 10% of genetic variants in the *LDLR* gene are not point mutations, ^99^ but rather large deletions or duplications of *LDLR* exons. Therefore, if no variants are identified by NGS, the multiplex ligation-dependent probe amplification ^102^ (MLPA) (MCR-Holland) technique should be used to identify probable deletions or duplications.

Cascade screening is cost-effective and should be used in all patients and first-degree relatives of those diagnosed with FH. The most cost-effective method is the one that uses genetic information from people with an identified FH-causing mutation. Clinical/biochemical screening should be performed even when genetic testing is not feasible. ^103^^-^^105^

The benefits and limitations of cascade genetic testing are summarized in Chart 2 , adapted from Sturm et al. ^23^

**Chart 2:** Benefits and limitations of cascade genetic testing. ^23^

**Benefits**
1. Provides a definite diagnosis for FH.
2. Provides prognostic information and the ability to perform refined risk stratification because the detection of a pathogenic variant indicates higher cardiovascular risk.
3. Positive genetic test results have been shown to improve initiation of lipid-lowering therapy, adherence to therapy, and reductions in LDL-C levels.
4. Early detection provides the opportunity for earlier treatment and lifestyle changes.
5. When the genetic test result of a proband is positive, this leads to cascade genetic testing in at-risk family members, with high sensitivity and specificity.
6.May exclude FH in at-risk family members who did not inherit the pathogenic variant(s).
7. Genetic testing provides discrimination at the molecular level between HeFH, compound HeFH, double HeFH, HoFH, ARH, and those without an identifiable pathogenic variant but with the FH phenotype. The recurrence risks to relatives and the implications for family planning differ between these scenarios.
8. Genetic testing allows the potential identification of FH “phenocopies” that may require specific therapies and have inheritance patterns different from those of FH.
9. May provide additional motivation for patients to properly adhere to prescribed medications.
10. Provides an explanation for failure of diet and exercise management to control elevated lipid levels.
11. Provides a helpful explanation for a family history of premature heart disease and difficult-to-treat LDL-C levels.
**Limitations**
1. FH genetic testing is not completely sensitive or specific.
2. Not all patients with a clinical diagnosis of FH will have an identifiable pathogenic variant (or variants).
3. Some patients will have a variant of uncertain significance identified, which may be reclassified as pathogenic or benign over time as more information is obtained.
**Cost**
1. Patients may want to undergo genetic testing, but the cost may be a limiting factor.

Adapted from Sturm et al. ^23^

### 4.8. Recommendation

1. Laboratory screening: All patients with suspected FH (index case) should have their first-degree relatives tested for hypercholesterolemia. If the result is positive, other family members (second- and third-degree relatives) should undergo cascade screening. Grade of recommendation: I; level of evidence: A.
2. Genetic screening: Genetic testing should be provided for the index case; if positive, first-degree relatives should be tested. If their test results are also positive, other family members (second- and third-degree relatives) should undergo cascade screening. Grade of recommendation: II; level of evidence: A.

## 5. Cardiovascular Risk Stratification

### 5.1. Epidemiology of Cardiovascular Risk in Familial Hypercholesterolemia

The association between HeFH and CAD is well established, ^51^^,^^105^ as the absence of lipid-lowering therapy implies a cumulative risk of fatal CAD of approximately 50% in men and 33% in women of up to 60 years of age. ^51^ A study conducted by the Simon Broome Register group from 1980 to 1995 identified a 125-fold increase in the relative risk of a fatal cardiovascular event in women with FH aged 20 to 39 years (annual mortality: 0.17%), despite treatment, compared with the general population of England and Wales. Men with FH aged 20 to 39 years had a 48-fold increase in relative risk (annual mortality: 0.46%). ^4^

More recent studies confirmed the increased risk of CAD among people with FH (LDL-C ≥ 190 mg/dL) of either monogenic or polygenic causes. Khera et al. ^24^ found an increased risk of cardiovascular events among participants with LDL-C levels ≥ 190 mg/dL, even in those without an identified FH mutation, compared with those with normal cholesterol levels. ^24^ Of 1,386 participants with LDL-C ≥ 190 mg/dL (6.7% of the total), only 24 (1.7%) had a detected mutation. Those with LDL-C levels ≥ 190 mg/dL and no mutation had a 6-fold higher risk of CAD compared with the control group (LDL-C < 130 mg/dL and no mutation), while those with LDL-C levels ≥ 190 mg/ dL and some mutation had a 22-fold higher risk. ^24^

Another recent study identified that, among patients with clinically diagnosed FH, the presence of a monogenic cause for the disease was associated with a significantly increased cardiovascular risk (adjusted hazard ratio [HR] 1.96; 95% CI 1.24 to 3.12; p = 0.004), while there was no difference in cardiovascular risk between patients with polygenic hypercholesterolemia and those without an identified genetic cause. However, the presence of a polygenic score in participants with monogenic FH further increased their cardiovascular risk (adjusted HR 3.06; 95% CI 1.56 to 5.99; p = 0.001). ^106^

Despite the increased risk of CAD observed in FH patients, statin treatment is associated with a significant reduction in the risk of cardiovascular events. Versmissen et al. ^46^ showed that patients treated with statins had a 76% reduction (HR 0.24; 95% CI 0.18 to 0.30; p < 0.001) in the risk of coronary events compared with “untreated” patients (ie, those with delayed initiation of treatment). However, although lipid-lowering therapy significantly decreases the chances of cardiovascular events, recent studies have shown a residual risk of events. In a study of 821 FH patients (median age: 47.4; 35.3 to 58.3) treated with lipid-lowering therapy for 9.5 years (5.1 to 14.2), 102 (12%) patients developed CVD. Patients more likely to develop CVD had a previous history of cardiovascular events, a family history of premature CVD, and hypertension, in addition to higher on-treatment LDL-C, lower on-treatment HDL-C, and higher smoking rates than patients without cardiovascular events. ^107^

The CASCADE FH registry evaluated cardiovascular outcomes in FH patients in the US. In a cohort of 1,900 people whose mean age was 56 ± 15 years, with a mean follow-up of 20 ± 11 years and a prevalence of previous atherosclerotic CVD of 37%, only 48% participants achieved LDL-C < 100 mg/dL and 22% achieved LDL-C < 70 mg/dL, despite the use of lipid-lowering therapy in 92.8%. A total of 107 atherosclerotic events occurred in 69 (3.6%) participants during follow-up, corresponding to an annual incidence of events of 2.2/100 patient-years. ^108^ Thus, FH carriers are at increased risk of atherosclerotic events even if on lipid-lowering treatment. The risk varies according to LDL-C control and the presence of several other factors, which indicates the importance of stratifying FH patients.

#### 5.1.1. Recommendations for Risk Stratification in Familial Hypercholesterolemia

Cardiovascular risk in FH is increased. However, although increased, it varies according to the presence of several risk factors. Therefore, risk stratification is recommended in FH patients (grade of recommendation: I; level of evidence: B)

### 5.2. Role of Risk Factors in Familial Hypercholesterolemia

Classical risk factors for CAD in FH are of great importance in the stratification of this population. For example, as is the case with the general population, cardiovascular risk among FH patients is higher in men than in women, as demonstrated in different studies. In a Dutch cohort study conducted by Jansen et al., ^109^ the risk of a cardiovascular event was almost 3 times higher in men than in women (relative risk [RR] 2.82; 95% CI 2.37 to 3.36). ^109^ A recent meta-analysis of 27 studies including 41,831 participants quantified the association between several risk factors and CVD in people with FH. The risk for men with CVD was almost 2 times higher (odds ratio [OR] 1.95; 95% CI 1.68 to 2.23). ^110^ In this meta-analysis as well as in other studies, smoking was strongly associated with the development of CAD in patients with FH, with a risk approximately 1.7 to 1.8 times higher than in nonsmokers. ^109^^-^^111^

Diabetes mellitus is an important cardiovascular risk factor in the general population. A meta-analysis of 12 prospective studies conducted by the Emerging Risk Factors Collaboration found that FH was associated with a 2-fold increase in CVD risk regardless of other risk factors. ^112^ As expected, diabetic FH patients are also at increased risk compared with nondiabetic FH patients. A meta-analysis conducted by Akioyamen et al. ^110^ found that diabetes (OR 1.95; 95% CI 1.33 to 2.57) and hypertension (OR 2.11; 95% CI 1.64 to 2.58) increased the CVD risk in FH patients by 2 times. Smoking, hypertension, and diabetes accounted for more than 1/4 of cardiovascular risk in those with FH. ^110^

In addition to traditional risk factors, other factors increase the risk of events in FH patients, such as a family history of CVD, which has been shown to be associated with a higher risk of CVD in those with FH. Akioyamen et al. ^110^ found that participants with a family history of CVD had an almost 2-fold higher CVD risk (OR 1.83; 95% CI 1.58 to 2.07).

Some previous studies failed to demonstrate an association between LDL-C and CVD in FH, although high LDL-C is the main feature of this condition. However, there are several explanations for this. For example, a comparison between participants with high LDL-C levels and those with similarly elevated LDL-C levels may not be effective in showing the effects of LDL-C differences, especially when the comparison involves a small number of participants. Furthermore, patients with higher LDL-C levels are generally treated more aggressively, which introduces a confounding factor in the analyses. However, in the recent meta-analysis conducted by Akioyamen et al., ^110^ meta-regression analyses showed that higher levels of untreated LDL-C and total cholesterol were associated with a higher risk of CVD (51% increase in CVD risk for every 1 mmol/L increase in cholesterol). Low levels of HDL-C (< 1 mmol/L) were also associated with increased cardiovascular risk in FH patients, unlike serum triglycerides and apolipoproteins A-I and B.

#### 5.2.1. Recommendation on the Role of Risk Factors in FH

Several factors play an important role in the cardiovascular risk of patients with FH and should be actively investigated in this population (grade of recommendation: I; level of evidence: A).

### 5.3. Role of Other Factors in Cardiovascular Risk in Familial Hypercholesterolemia: Lipoprotein(a), Achilles Tendon Xanthoma, and C-Reactive Protein

Lp(a) is a lipoprotein composed of an LDL-like particle whose Apo B is covalently linked to an apolipoprotein(a).

According to evidence gathered over several years, high Lp(a) is an independent cardiovascular risk factor in the general population, with causal implication. ^112^^-^^114^ In FH patients, the condition is also considered an additional risk factor, which is an extremely relevant observation in this group given the potential for high Lp(a) levels in this population.

#### 5.3.1. Recommendation

Lp(a) measurement should be considered in those with FH (grade of recommendation: IIa; level of evidence: B).

Achilles tendon xanthoma is a peculiar sign of FH and is included in the diagnostic criteria. Approximately 30 to 50% of patients genetically diagnosed with HeFH have tendinous xanthoma. Civeira et al. ^115^ showed that FH carriers with xanthomas had a higher prevalence of premature CVD compared with those without xanthomas (36.7% vs 13.8%, p = 0.001). ^115^ A meta-analysis conducted by Oosterveer et al. ^116^ of patients with genetically confirmed FH identified a 3-fold higher risk of CVD among those with tendinous xanthoma. ^116^

A more recent Brazilian study also evaluated the association of Achilles tendon xanthomas with the presence and burden of subclinical atherosclerosis in HeFH patients. Participants with xanthomas (21%) had higher concentrations of LDL-C and Lp(a), as well as a higher calcium score. Furthermore, the association of xanthoma with calcium score remained positive and independent after adjustments for age, sex, smoking, hypertension, previous statin use, HDL-C, LDL-C, and Lp(a). ^117^

Despite the positive association between xanthomas and CVD found in previous studies, the recent meta-analysis conducted by Akioyamen et al. ^110^ did not report tendon xanthomas as risk factors in FH.

Other recommendations include the following:

1. Achilles tendon xanthoma seems to be associated with a higher cardiovascular risk in FH. Because xanthomas are often investigated only during physical examination, this should be encouraged (grade of recommendation: IIA; level of evidence: B).
2. The association between C-reactive protein and CVD in FH is based on small studies of subclinical atherosclerosis with controversial results. ^118^^,^^119^
3. There is no evidence to support routine C-reactive protein measurement in FH (grade of recommendation: IIB; level of evidence: C).

### 5.4. Cardiovascular Risk Stratification in Familial Hypercholesterolemia: Use of Clinical Scores for Risk Stratification

Conventional risk stratification with widely used clinical scores, such as the Framingham score, the Framingham Global score, the American Heart Association/American College of Cardiology (AHA/ACC) score, and others, was not designed for patients with FH. ^22^ Indeed, a person with long-term exposure to high cholesterol levels over time (cholesterol-years score) cannot be approached as being at possibly low cardiovascular risk as in a traditional score.

Therefore, current studies on risk stratification in FH should include, if possible, a prospective design, molecular diagnosis, and the attenuating effects of previous statin therapy. In this context, Paquette et al. ^120^ developed the Montreal-FH-SCORE by evaluating 670 participants with a confirmed molecular diagnosis of FH and who had undergone statin treatment. ^120^ Being male, age, hypertension, and smoking independently associated with incidence of atherosclerotic CVD. More recently, the authors validated their risk equation in a different population of 718 patients with a molecular diagnosis of FH with good statistical discrimination; ^121^ however, these studies are considered limited by their retrospective design and relatively small incidence of events.

Using the prospective Spanish Familial Hypercholesterolemia Registry (SAFEHEART), Perez de Isla et al. ^122^ developed a new equation by adding the following to the risk markers defined in the Montreal-FH-SCORE: previous atherosclerotic cardiovascular event, high body mass index (BMI) (> 30 kg/m^2^), high residual LDL-C concentrations (> 100 or > 160 mg/dL), and Lp(a) levels > 50 mg/dL. ^122^ These parameters were found to be independently associated with first or recurrent atherosclerotic cardiovascular event. Indeed, the SAFEHEART score had a good discrimination index (0.85 overall and 0.81 in primary prevention), with an excellent calibration for both primary and secondary preventions. However, the score was limited by the relatively low incidence of events (5.6%), a possible confounding effect, by previous statin therapy, and by a relatively short follow-up. Finally, similar to the Montreal-FH-score, the SAFEHEART registry equation was limited by the lack of validation in other FH populations. ^123^

#### 5.4.1. Recommendation

1. Montreal-FH-SCORE: grade of recommendation, IIb; level of evidence, B.
2. SAFEHEART score: grade of recommendation, IIa; level of evidence, B.
3. Framingham score or other clinical FH scores: grade of recommendation, III; level of evidence, B.

### 5.5. Coronary Artery Calcium Score

The coronary artery calcium (CAC) score is a tool for quantifying the total burden of coronary artery atherosclerotic plaque: the higher the CAC score, the greater the plaque burden in the patient. The most common method to assess the CAC score is using the Agatston score, which corresponds to the weighted sum of lesions with a density above 130 Hounsfield units (HU). Then, the calcium area is multiplied by a factor related to maximum plaque attenuation: factor 1 – if maximum attenuation < 200 HU; factor 2 – if maximum attenuation between 200 and 300 HU; factor 3 – if maximum attenuation between 300 and 400 HU; or factor 4 – if maximum attenuation ≥ 400 HU. ^124^

Several studies have demonstrated the association of high CAC scores with coronary events. ^125^^,^^126^ A 2004 meta-analysis conducted by Pletcher et al. ^127^ shows a linear association between CAC and the risk of coronary events. Those with a CAC score > 400 Agatston units (AU) were at higher risk of cardiovascular events. The most relevant studies regarding the association of CAC with predicted CAD are the Multi-Ethnic Study of Atherosclerosis (MESA) ^125^ and the Heinz Nixdorf Recall Study (HNR), ^126^ which showed that CAC is an independent marker of death and myocardial infarction. Not only did the score add discriminative power but also improved risk reclassification for CAD compared with classical risk factors. In the MESA study, a prospective evaluation of 6,814 patients followed-up for a mean period of 3.8 years showed that coronary event risk rates were increased by 7.73 for those with a CAC score between 101 and 300 AU and by 9.67 for those with a CAC score ≥ 300 AU (p < 0.001) compared with those with no CAC score. ^125^ Also, a CAC score of zero is associated with low rates of coronary events even in the medium term (11 years). ^128^

No randomized studies have addressed the management of lipid profile guided by CAC, but observational studies have showed that those with higher CAC scores benefit the most from statin therapy. ^129^^,^^130^ The 2017 Brazilian guideline for dyslipidemia recommends that primary prevention patients with high CAC scores (> 100 AU) be considered at high cardiovascular risk and be treated according to appropriate lipid goals. ^52^ The 2018 AHA/ACC guideline goes further and proposes postponing statin treatment in primary prevention patients aged 40 to 75 years without diabetes mellitus, with LDL-C levels between 70 and 189 mg/dL, and with a CAC score of zero. ^53^

FH carriers have higher CAC scores than noncarriers matched for age and sex. ^118^ Coronary artery calcification determinants in FH are the classical risk factors for atherosclerosis. Indeed, Martinez et al. ^118^ showed that the LDL-C exposure burden corresponding to the LDL-C-years score (LDL-C multiplied by age), the Framingham score, and being male were associated with CAC. A French study also showed an association between CAC and cholesterol-years score (total cholesterol multiplied by age). ^131^

Guidelines for the treatment of dyslipidemia consider FH patients as being at least at high cardiovascular risk for having high LDL-C since childhood. ^52^^,^^132^ In this context, questions remain regarding the usefulness of the CAC score for risk stratification in FH when dealing with a population at high cardiovascular risk. A MESA study subanalysis showed an association between CAC and CVD even when LDL-C is high (LDL-C > 190 mg/dL). Patients with a CAC score of zero had low rates of cardiovascular events (10-year risk: 3.7%, per-year risk: 0.4%) when compared with those with a CAC score > 0 (10-year risk: 20%; per-year risk: 2.0%). The factors associated with a CAC score of zero were: age < 65 years, being female, and absence of diabetes mellitus. The association of CAC with cardiovascular events was analyzed in a Brazilian cohort of patients with confirmed molecular diagnosis of FH and in primary prevention. The study showed an association between CAC and cardiovascular events in FH despite high-potency statin treatment. ^133^ The mean age was 45 years, and 15 cardiovascular events were reported. The annual rates of events per 1,000 patients for CAC scores of 0, 1 to 100, and > 100 were, respectively, 0; 26.4 (95% CI 12.9 to 51.8); and 44.1 (95% CI 26.0 to 104.1). Despite the small sample size (n = 206) and the relatively short follow-up (median time: 3.7 years), the study demonstrated that a CAC score of zero can also be used as a marker of good prognosis in the FH population. ^133^

Thus, the CAC score is an additional tool in the risk stratification of HeFH patients in primary prevention (grade of recommendation: IIb; level of evidence: B).

### 5.6. Coronary Computed Tomography Angiography

Compared to the CAC score, coronary computed tomography (CT) angiography has some advantages such as visualization of noncalcified plaques and estimation of the degree of luminal stenosis. ^134^ Conversely, the disadvantages include the need for intravenous contrast infusion, higher costs, and higher radiation doses.

The use of coronary CT angiography has clear benefits in symptomatic patients at low risk for CAD both in the emergency department and in the outpatient setting as it rules out the disease as the cause of symptoms. ^135^ However, the benefits of this method over the CAC score in the risk stratification of asymptomatic patients are controversial. For example, the COronary CT Angiography EvaluatioN For Clinical Outcomes InteRnational Multicenter (CONFIRM) registry did not show any advantage for coronary CT angiography over the CAC score in asymptomatic individuals. ^136^ However, two CONFIRM substudies showed that coronary CT angiography could improve stratification compared with the CAC score in patients at higher risk, particularly older patients ^137^ and those with intermediate-level CAC. ^138^

Previous studies have shown that FH carriers have a greater atherosclerotic plaque burden in coronary CT angiography, which is represented by a greater number of patients with plaque, luminal stenosis, and plaque segments compared with normolipidemic controls. ^139^^,^^140^ The condition is associated with a higher risk of cardiovascular outcomes in a noncarrier population. ^141^ The question, then, is about the usefulness of coronary CT angiography in asymptomatic FH patients in primary prevention. A small Japanese study including 101 FH carriers showed that a tomographic score based on segments with stenosis on coronary CT angiography was associated with major cardiac events. ^142^ However, the study was not able to provide a definite answer because of its small sample size, retrospective design, and the fact that many events occurred soon after the examination, which could mean that several revascularization events were “caused” by the examination. The only randomized clinical trial assessing the influence of coronary CT angiography on clinical outcomes in asymptomatic patients was conducted in a diabetic population and showed no benefit. ^143^ Therefore, coronary CT angiography is not currently recommended for asymptomatic HeFH patients in primary prevention (grade of recommendation: III; level of evidence: B).

However, the usefulness of coronary CT angiography in HoFH should be analyzed from a different perspective, since the disease is associated with accelerated atherosclerosis, and cardiovascular events and supravalvular aortic stenosis may be developed very early. ^13^ There are studies with small sample sizes demonstrating that HoFH patients may have coronary atherosclerosis and atherosclerotic involvement of the aortic root detected by coronary CT angiography at an early age. ^144^^,^^145^

The examination can be performed even in asymptomatic HoFH patients at diagnosis for a better assessment of cardiovascular risk profile. It can be repeated at the clinician’s discretion (grade of recommendation: IIa; level of evidence: B).

### 5.7. Carotid Intima-Media Thickness

Carotid intima-media thickness (IMT) is defined as the distance between the lumen-intima interface and the media-adventitia interface. It is related to cardiovascular risk factors, prevalence and incidence of CVD, and degree of atherosclerosis at different arterial sites. The progression of carotid IMT can be reversed or attenuated by interventions in risk factors, which is associated with a reduction in cardiovascular events. ^146^ These findings suggest that carotid IMT is a potential surrogate marker of atherosclerosis. Martinez et al. analyzed carotid IMT in FH patients ^118^ and found increased measures in the FH group compared with the control group.

Carotid IMT was used as a surrogate marker of atherosclerosis in an FH population to assess the progression of atherosclerosis with lipid-lowering medication. ^147^^,^^148^ Increased carotid IMT was found in children with FH compared with those without FH. Also, a previous study of people aged 8 to 18 years showed a trend toward regression of carotid IMT with pravastatin, whereas a trend toward progression was observed in the placebo group. ^149^ Rosuvastatin treatment was able to decelerate the progression of carotid IMT in FH children aged ≥ 6 years compared with unaffected children, with no between-group difference after 2 years of treatment. ^150^

However, the potential use of carotid IMT in clinical practice is hampered by the variability in the methods of measurement, including measurement site, influence of cardiac cycle (no standardization on whether it should be measured in systole or diastole in different studies), use of mean or maximum measurements, definition of abnormal carotid IMT, and other difficulties in standardizing the method. ^151^ Also, the MESA study compared risk reclassification between different biomarkers in intermediate-risk patients and found that carotid IMT had low reclassification power compared with CAC: net reclassification improvement (NRI) was 0.060 for carotid IMT and 0.406 for CAC, which demonstrates the superiority of the latter. ^152^

A carotid ultrasound can also indicate the presence of carotid plaques. The relative risk of carotid plaques varies widely, ranging from 1.16 to 6.71 in different studies, possibly because of different definitions: carotid IMT > 1.2 mm; carotid IMT > 1.0 mm with protrusion into the lumen; subjective analysis; focal thickening > 50% around the carotid IMT or > 1.5 mm; and others. Furthermore, considering only the presence or absence of carotid plaque may be too simplistic given the diversity of plaque phenotypes (calcified, noncalcified, focal, etc.). ^153^ Therefore, the use of carotid Doppler ultrasound in asymptomatic FH patients for assessment of carotid IMT and carotid plaque can optimize cardiovascular risk stratification (grade of recommendation: IIb; level of evidence: B).

### 5.8. Investigation of Myocardial Ischemia

The investigation of myocardial ischemia with exercise testing is recommended for those who are planning to start high-intensity and/or competitive sports activities. A previous study of 639 FH patients diagnosed by clinical criteria found that 9% of tests were positive for myocardial ischemia. The study also showed that exercise testing parameters such as decreased exercise capacity, delayed decrease in heart rate during the first minute of graded exercise, and increased peak pulse pressure were predictors of coronary events. ^154^ A different study of 194 FH patients detected a rate of 21% of positive exercise tests. ^155^

Although there are no randomized studies of exercise testing in FH, and the few existing ones use clinical diagnostic criteria, periodic performance of exercise testing may be useful for asymptomatic HeFH patients who wish to start recreational or competitive physical activity, as well as for those with additional risk factors for coronary heart disease or delayed initiation of lipid-lowering treatment. The test can be repeated every 3 to 5 years (grade of recommendation: IIb; level of evidence: C). Additional risk factors or markers in patients with FH may be considered according to Table 3 .

**Table 3:** Risk factors/markers of cardiovascular risk in familial hypercholesterolemia.

Risk factor/marker	Grade of recommendation	Level of evidence
Diabetes mellitus	I	B
Hypertension	I	B
Smoking	I	B
Family history of premature CAD in first-degree relatives (men aged < 55 years and women aged < 60 years)	I	B
Initiation of lipid-lowering treatment after 40 years of age	IIa	B
HDL-C < 40 mg/dL	I	B
Lipoprotein(a) > 50 mg/dL (or > 125 nmol/L)	IIa	B
Achilles tendon xanthoma	IIb	B
Calcium score > 100 AU or > 75th percentile	IIa	B
Presence of atherosclerotic plaque with obstruction > 50% at any arterial site	IIa	C

CAD: coronary artery disease; HDL-C: high-density lipoprotein cholesterol.

### 5.9. How to Perform Cardiovascular Risk Stratification of Patients with Familial Hypercholesterolemia in Clinical Practice

As previously discussed, patients with FH are at high risk of cardiovascular events in primary prevention compared with those without FH. However, the presence of classical risk factors for CAD further increases the risk, which contributes to risk heterogeneity. Even in the context of secondary prevention, there is evidence that the risk of recurrence after an index cardiovascular event in patients with FH is at least 2-fold higher than in non-FH patients. ^156^

Santos et al. ^56^ defined FH carriers according to the presence of the following **additional risk factors**: age > 40 years and no treatment; smoking; being male; Lp(a) > 50 mg/dL (> 125 nmol/L); HDL-C < 40 mg/dL; hypertension; diabetes mellitus; family history of premature CVD in first-degree relatives (men aged < 55 years and women aged < 60 years); chronic kidney disease (glomerular filtration rate [GFR] < 60 mL/min); and BMI > 30 kg/m^2^. All these **additional risk factors** characterize higher risk situations.

Based on the presence of significant manifest or subclinical atherosclerotic disease, **additional risk factors** , and very high LDL-C levels, FH patients can be classified into the following three risk categories. ^132^

#### 5.9.1. Very High-Risk

- Patients with clinically manifest ASCVD, defined as previous myocardial infarction, angina pectoris, previous myocardial revascularization, stroke or transient ischemic attack, and intermittent claudication.
- Patients with advanced subclinical atherosclerotic disease diagnosed by CAC score > 100 AU or 75th percentile for age and sex, or coronary CT angiography showing coronary obstructions > 50% or presence of nonobstructive plaques in more than one vessel.

#### 5.9.2. High-Risk

- In primary prevention of HeFH, those with LDL-C > 400 mg/dL, even with no **additional risk factors** .
- In primary prevention of HeFH, those with **additional risk factors** .

Note: If LDL-C > 310 mg/dL with one high-risk feature; if LDL-C > 190 mg/dL with two high-risk features ( **additional risk factors)** .

#### 5.9.3. Intermediate-Risk

- In primary prevention of HeFH, those with no **additional risk factors** .

## 6. Nutritional Recommendations

Following a healthy eating pattern is crucial for the treatment of FH because a poor diet may increase the established cardiovascular risk in FH patients. ^157^ The latest guideline for dietary treatment of FH, developed jointly by the American College of Cardiology (ACC), the American Heart Association (AHA), and other US societies, ^53^ is based on a previous guideline published by the societies in 2013. ^158^ The document recommends following healthy eating patterns with adequate energy intake, removing *trans* fatty acids, adjusting the intake of saturated fatty acids (SFAs), and encouraging an adequate consumption of monounsaturated fatty acids (MUFAs) and polyunsaturated fatty acids (PUFAs). ^158^ How these fatty acids act on plasma cholesterol has been exhaustively evaluated in several experimental, clinical, and epidemiological studies. ^158^^,^^159^ Many results were controversial because of differences between the studies regarding duration, sample size, and type of nutrient used for comparison purposes. ^132^ Also, different fatty acids are obtained from different food sources, such as meat, milk, oils, or processed foods, and may thus induce different effects on plasma lipids. ^160^

In recent years, the type of food has been more highly valued than the type of nutrient alone. Thus, eating patterns such as the Mediterranean diet ^161^^,^^162^ and the DASH diet, ^163^ which are based on the consumption of grains, fruits, vegetables, lean meats, dairy products with reduced fat content, and oleaginous fruits (walnuts and chestnuts), were included in the recommendations of major international guidelines. Also, moderate use of vegetable oils rich in PUFAs (omega-3 and omega-6 acids) and MUFAs (omega-9 acids) is indicated for food preparation. ^53^ This recommendation is based on two important studies. The recent Cohorts for Heart and Aging Research in Genomic Epidemiology (CHARGE) study showed that plasma and tissue concentrations of omega-6 fatty acid biomarkers were associated with decreased cardiovascular events. ^164^ Similar results were observed with plasma concentrations of omega-3 and omega-6 fatty acid biomarkers, which were associated with decreased cardiovascular risk. ^165^

The guideline developed jointly by the European Society of Cardiology (ESC) and the European Atherosclerosis Society (EAS) ^132^ reinforces the previously mentioned recommendations and warns that foods such as palm oil, coconut oil, bacon, cookies, high-fat bakery products, and full-fat dairy products should be consumed only occasionally and in minimal amounts by FH patients. ^53^

### 6.1. Dietary Cholesterol

In recent years, the AHA guidelines ^53^^,^^158^ and the Dietary Guidelines for Americans (2015-2020) ^166^ removed an upper limit for cholesterol intake because of limited evidence relating it to atherosclerosis ^167^ and CAD. ^168^ Nevertheless, the Dietary Guidelines for Americans suggest that considering dietary cholesterol when adopting healthy eating patterns is important, which is consistent with the Institute of Medicine recommendations ^169^ about the benefits of low cholesterol intake.

The hypercholesterolemic action of dietary cholesterol is lower than that of saturated fats, which is why dietary guidelines have emphasized the importance of reducing the consumption of saturated fatty acids. Thus, reducing the intake of saturated fats from animal sources ensures that cholesterol levels are lower, since both are found in the same foods. In general, the balance between cholesterol intake and endogenous cholesterol synthesis is responsible for cholesterol homeostasis; ^170^ however, increased intake may significantly contribute to high plasma concentrations of LDL-C, ^171^ a response that is subject to great interpersonal variability and dependent on metabolic and genetic factors. ^170^^,^^172^^,^^173^

Indeed, the 2018 AHA/ACC guideline on the management of blood cholesterol, which is based on a document first published in 2014, found no evidence to determine whether reducing dietary cholesterol also lowers LDL-C. ^158^

Observational studies and meta-analyses evaluating the influence of dietary cholesterol on the risk of developing type 2 diabetes, CAD, and stroke are inconclusive ^168^^,^^171^^,^^173^^-^^177^ or show no association with CVD and mortality. ^178^ However, a study evaluating the databases of Atherosclerosis Risk in Communities (ARIC), Coronary Artery Risk Development in Young Adults (CARDIA), Framingham Heart Study (FHS), Framingham Offspring Study (FOS), Jackson Heart Study (JHS), and MESA studies showed that increased cholesterol intake is dose-dependently related to increased CVD and overall mortality, ^179^ possibly because of the impact on LDL-C concentrations. ^180^

Eggs, despite being a source of cholesterol, are also highly nutritious and have an excellent profile of high biological value proteins, vitamins, and minerals, in addition to being affordable. Given such nutritional qualities, eggs should be included in the diet as long as they are part of a healthy eating pattern.

An increase in mortality and cardiovascular events due to a higher consumption of total cholesterol and from egg is independent of the quality of the diet. Therefore, moderate consumption of eggs and other sources of cholesterol is recommended, ^179^ especially among those with higher plasma lipid concentrations and those who are hyperresponsive to cholesterol intake.

### 6.2. Action of Fatty Acids on Cholesterolemia

#### 6.2.1. Saturated Fatty Acids

Fatty acids are classified as SFAs, MUFAs, PUFAs, or *trans* fatty acids, and each category has a different influence on plasma concentrations of total cholesterol and LDL-C. Among the main SFAs found in foods, we have the lauric acid (12:0), myristic acid (14:0), palmitic acid (16:0), and stearic acid (18:0), but only palmitic acid is abundant in nature. Coconut oil is an exuberant source of lauric and myristic acids; meat has a high content of palmitic acid; and milk is rich in stearic and myristic acids. Foods from plant sources, such as palm oil and cocoa, also have high contents of palmitic and stearic fatty acids, respectively. ^181^^,^^182^ Pentadecylic (15:0) and margaric (17:0) fatty acids are found in small amounts in dairy products, and their plasma concentration is a marker of consumption. ^183^^,^^184^ Foods also provide longer-chain fatty acids such as arachidic acid (20:0), behenic acid (22:0), and lignoceric acid (24:0), which are found in oleaginous fruits such as peanuts and macadamia nuts. ^185^^,^^186^

Several clinical and epidemiological studies have shown that cardiovascular risk increases with a higher consumption of SFAs ^186^^-^^188^ because they induce greater plasma concentrations of total cholesterol and LDL-C. ^159^ Several mechanisms have been proposed to explain this action, including: (a) SFAs have straight carbon chains and are closely packed in the core of lipoproteins, which allows the accommodation of a greater amount of cholesterol; ^189^ (b) in association with cholesterol, SFAs reduce LDLR activity, protein, and mRNA levels, ^190^^-^^192^ a change that decreases the metabolism of LDL particles. ^193^^,^^194^

The World Health Organization (WHO) published a systematic review of clinical studies (mean SFA intake: 9.8%) and showed that isocaloric replacement of SFAs with PUFAs or MUFAs reduced plasma concentrations of total cholesterol and LDL-C. ^159^ Furthermore, replacement of carbohydrates with palmitic, myristic, or lauric acids was also found to induce an increase in those lipid parameters, an effect that was not observed with stearic acid. This reinforces the idea that SFAs behave differently with regard to their effect on plasma lipids. Stearic acid does not elevate plasma cholesterol because it is rapidly converted to oleic acid by the action of the liver enzyme stearoyl-CoA desaturase-1 (SCD1). ^195^

The Prospective Urban Rural Epidemiology (PURE) ^196^ study evaluated the diets of 135,000 people in 18 countries and showed that higher fat consumption (35% of energy) is related to lower mortality compared with lower fat consumption (10% of energy). It is worth noting that this amount of fat is within the recommended range (25 to 35% of total energy intake, TEI) reported in recent decades, and that median consumption ranged from 2.8 to 13.2% of energy. That study showed that increased SFA consumption was associated with higher LDL-C. ^196^

A 2015 Cochrane Library systematic review evaluating data from randomized clinical trials (59,000 participants in total) showed that decreased consumption of saturated fat from the usual diet reduced cardiovascular events by 17%. ^197^ Also, the results showed that cardiovascular events reduce by 27% when SFAs are replaced with PUFAs.

While some SFAs are related to greater cardiovascular risk, others are inversely associated, such as pentadecylic (15:0) and margaric (17:0) acids, ^183^ as well as those with a very long chain, such as lignoceric, behenic, and arachidic acids. ^185^

Thus, consumption of up to 10% of energy for SFAs is recommended, with a 7% limit for those with hypercholesterolemia, according to 2019 ESC/EAS ^132^^,^^158^ and ACC/AHA guidelines.

#### 6.2.2. Unsaturated Fats

Oleic acid (18:1) is the main MUFA, found in olive oil, canola oil, oilseeds, and oleaginous fruits. Omega-6 PUFAs consist of linoleic acid (18:2), found in corn, sunflower, soybean, and canola oils, and arachidonic acid (20:4), found in eggs, fish oil, and meat. ^198^ Omega-3 fatty acids can be from both plant and animal sources; *α* -linolenic acid (18:3) is found in linseed, canola, and soybean oils, and eicosapentaenoic acid (20:5) and docosahexaenoic acid (20:6) are found in very cold water fish.

Contrasting with saturated fats, MUFAs and PUFAs do not elevate plasma concentrations of cholesterol and LDL-C, a benefit that seems to be greater with PUFAs, since MUFAs have a neutral action on cholesterolemia. ^158^

#### 6.2.3. Omega-3 Fatty Acids

Several clinical studies have shown that supplementation with pharmacological doses (2 to 4 g) of eicosapentaenoic acid (EPA) and docosahexaenoic acid (DHA) per day can decrease plasma concentrations of triglycerides by up to 25 to 30%, in addition to slightly increasing those of HDL-C (1 to 3%). It can also elevate LDL-C concentrations by up to 5 to 10%, with little or no difference in serum total cholesterol. ^199^^-^^202^

However, in the case of formulations containing only purified omega-3 fatty acids, LDL-C increase may not occur, as demonstrated in the Japan EPA Lipid Intervention Study (JELIS). ^203^^,^^204^ Research on the effect of omega-3 fatty acids on FH patients remains incipient, and supplementation seems to be able to influence the progression of atherosclerosis in high-risk patients, but this requires further investigation. ^205^^-^^207^

#### 6.2.4. Trans Fatty Acids

The consumption of *trans* fatty acids present in processed foods increases cardiovascular risk because it induces an atherogenic lipid profile by elevating plasma LDL-C concentrations, decreasing Apo B catabolism, ^208^ reducing HDL-C, and inducing increased Apo A-I catabolism. ^209^ Also, *trans* fatty acids increase the severity of atherosclerotic lesions in CAD ^209^ and induce endothelial dysfunction. ^210^

The results of controlled studies discussed in a meta-analysis showed that for every 1% energy replacement of *trans* fats with saturated, polyunsaturated, or monounsaturated fats, total cholesterol/HDL-C ratio reduced by 0.31, 0.54, and 0.67, respectively. ^211^ The evaluation of prospective studies in another meta-analysis revealed that for every 2% energy replacement of *trans* fats with other fatty acids, the risk of CAD decreases by 17%. ^211^ Subsequently, a higher consumption of *trans* fat observed over a 10-year period in several countries was found to be associated with a 4% increase in deaths from coronary heart disease. ^212^ A study evaluating diet-related mortality rate in 195 countries found a considerable number of cardiovascular deaths attributed to the consumption of *trans* fatty acids. ^213^ In addition to adverse effects on lipid metabolism, *trans* fat can induce a proinflammatory profile, which further intensifies its deleterious effects. ^214^ For all their adverse reactions, *trans* fatty acids used in processed foods should be excluded from diet. ^53^^,^^158^^,^^208^^,^^213^^,^^215^

#### 6.2.5. Phytosterols

Phytosterols, phytostanols, and their esters are bioactive components present in plant foods, and their chemical structure is highly similar to that of cholesterol. ^216^ Their hypocholesterolemic effect is well documented in the scientific literature. After being incorporated to micelles, phytosterols are transported into the enterocyte via NPC1L1 transporter, and then most return to the intestinal lumen via ABCG5/ABCG8 transporters, ^217^ which keeps their plasma concentrations low. The mechanism for reducing plasma concentrations of total cholesterol and LDL-C is explained by the greater solubility of phytosterols in micelles, which displaces cholesterol and promotes its excretion. The mean consumption of phytosterol in the population is 100 to 400 mg/day, and a Brazilian study revealed that the mean consumption was 160 mg/day. ^218^ A daily supplementation of 2 g of phytosterols was shown to reduce plasma LDL-C concentrations by 8 to 10%. ^217^^,^^219^ Despite the small lowering effect on cholesterolemia, the current ESC/EAS guideline ^53^ indicates that phytosterols may be beneficial for adults and children with moderately high cholesterol levels. ^217^ However, because responses to the use of phytosterols may vary, efficacy should be evaluated individually. ^220^

Phytosterols can be administered in capsules containing 650 to 900 mg or spread (two tablespoons provide the recommended dose). Their use should be accompanied by healthy dietary and lifestyle habits to lead to the desired effects. Phytosterol consumption during the main meals seems to be the best option because of the mechanism of action of competition in the absorption of dietary cholesterol. ^221^

In FH patients, phytosterol can help achieve LDL-C goals when used in combination with statin/ezetimibe. ^222^ Meta-analysis data on the effect of phytosterols on children demonstrated reductions in total cholesterol (7 to 11%) and LDL-C (10 to 15%). ^223^ According to the Expert Panel on Integrated Guidelines for Cardiovascular Health and Risk Reduction in Children and Adolescents, phytosterol supplementation (2 g/day) can be a good option for children and adolescents with FH who are not yet eligible for pharmacological treatment, ^224^ since phytosterols are well tolerated and do not have significant adverse effects. In Brazil, phytosterols are approved for use in children aged 5 years or over; however, few studies have tested their use during pregnancy and lactation, and caution is recommended in such cases. ^225^ Importantly, their use is contraindicated for patients with sitosterolemia.

#### 6.2.6. Fiber

Dietary fiber intake seems to be associated with a significant reduction in total cholesterol because of mechanisms involving: 1) reduced cholesterol absorption induced by viscosity; ^226^ 2) increased fecal excretion of cholesterol and bile acid, ^227^ inducing increased activity of 7 **α** -hydroxylase, ^228^ a key enzyme in the formation of bile acids from cholesterol; ^229^ 3) reduced activity of 3-hydroxy-3-methylglutaryl-coenzyme A (HMG-CoA) reductase; ^230^ 4) reduced hepatic cholesterol content; ^231^ 5) altered composition of intestinal microbiota, leading to higher production of short-chain fatty acids and increased excretion of neutral steroids and bile acids; ^232^ or even a combination of the described mechanisms.

A meta-analysis of studies investigating the effect of soluble fiber ( **β** -glucan) on plasma lipids found that an intake > 3 g/day led to a modest reduction in plasma concentrations of total cholesterol and LDL-C (11.6 and 9.6 mg/dL, respectively, relative to control), with no effect on HDL-C and triglycerides. ^233^ A Cochrane Library systematic review ^234^ evaluated the effects of fiber on primary prevention of CVD and found reduced total cholesterol and LDL-C levels (7.7 and 8.5 mg/dL, respectively) associated with increased fiber intake. However, the authors highlight that randomized, well-conducted, and long-term cohort studies are necessary to reliably determine the effect of fiber on cardiovascular health. ^234^

In line with the current ESC/EAS guideline, ^53^ following a diet rich in fiber, especially soluble fiber, is recommended. This is found in vegetables, fruits, and whole grains, which should be part of a healthy eating pattern.

#### 6.2.7. Soybean

The controversial results of studies evaluating the action of fat-free soy protein supplementation with or without isoflavones on plasma concentrations of total cholesterol and LDL-C can be partly attributed to the different methods used in the studies, the presence of other substances such as fiber and phospholipids, and the use of different concentrations of soy protein or isoflavones. ^235^^,^^236^ Evidence suggests that soy protein is responsible for a modest reduction (~3%) in plasma cholesterol concentrations, and not isoflavone alone. ^237^ Nevertheless, there is no evidence for the indication of isoflavone supplementation in the treatment of hypercholesterolemia. However, soy-based products have low concentrations of saturated fat and are rich in fiber, vitamins, minerals, and unsaturated fatty acids, thus they can be part of a healthy eating plan.

#### 6.2.8. Chocolate

Cocoa beans are extracted from the cocoa tree (Theobroma cacao L.), which is native especially to South America and the west coast of Africa. Chocolate is obtained by mixing cocoa products (cocoa mass, cocoa powder, or cocoa butter) with additional ingredients such as sugar, milk, lecithin, nuts, and fruits. Thus, in addition to having a high energy density, chocolate is also rich in fat and sugar. Approximately 60% of cocoa fat consists of SFAs (stearic and palmitic acids), and approximately 30% is oleic acid. Polyunsaturated fats are 3 to 5% of all fatty acids present in cocoa. ^238^ High consumption of saturated fats has been shown to elevate plasma cholesterol concentrations; however, the results of two meta-analyses showed that cocoa-rich (dark) chocolate seems to have no hypercholesterolemic action, ^239^^,^^240^ possibly because stearic acid, the main fatty acid in cocoa, is rapidly converted to oleic acid through the action of SCD1 in the liver. ^241^ However, it is worth noting that chocolate can be an important source of simple sugar and energy; therefore, it should be consumed in moderation, so that it does not contribute to weight gain. Also, chocolate is usually made from other sources of fat.

#### 6.2.9. Tropical Oils

##### 6.2.9.1. Coconut Oil

Coconut oil ( *Coco nucifera* ) consists primarily of saturated fat (82%), of which 42% is lauric acid, 16% is myristic acid, 9% is palmitic acid, and the rest is caprylic, capric, and stearic acids. ^242^ Coconut oil has a low concentration of unsaturated fats and lacks an essential fatty acid, **α** -linolenic acid (18:3). ^181^^,^^243^ When compared with consumption of olive oil ^244^ and safflower oil, ^245^ coconut oil consumption increases plasma concentrations of total cholesterol and LDL-C. A study of normolipidemic men living in Sri Lanka showed that isocaloric replacement of coconut oil with soybean oil reduced plasma concentrations of total cholesterol, LDL-C, and triglycerides. ^246^ Similar results were found in dyslipidemic adults after replacement of coconut oil with corn oil. ^247^ Increased HDL-C concentrations with coconut oil consumption were accompanied by high LDL-C levels, which are a major cardiovascular risk factor. ^248^

Furthermore, because it is rich in lauric acid, ^249^ coconut oil can trigger inflammatory signaling pathways by activating receptors related to the innate immune response, named toll-like receptors (TLRs). ^249^^-^^251^ A study of macrophages found that lauric acid increased the expression of cyclooxygenase-2 (COX-2) by activating the NF- **κ** B/TLR 2 and 4 pathways. ^252^ Regarding oxidative capacity, no difference was found in energy metabolism and lipid oxidation when the acute effects of coconut oil and olive oil consumption were compared in overweight women. ^253^

##### 6.2.9.2. Palm Oil

Despite being a vegetable oil, palm oil is approximately 50% SFAs (45% palmitic acid and 5% stearic acid) and 50% unsaturated fats (40% oleic acid and 10% linoleic acid). Thus, greater palm oil intake, either added to food preparation or consumed in processed foods, increases the dietary concentrations of saturated fat. Consumption of palm oil, when compared with that of vegetable oils rich in unsaturated fat, increases plasma concentrations of total cholesterol and LDL-C. ^254^ The results of a meta-analysis comparing palm oil to vegetable oils (eg, canola oil, soybean oil, and olive oil) showed that palm oil consumption elevated the concentrations of total cholesterol, LDL-C, and HDL-C, the latter modestly. ^255^ This showed that, regarding plasma lipids, palm oil is similar to animal fats, which are rich in saturated fat. ^255^^,^^256^ Therefore, consumption should be kept within the recommended amount of saturated fat.

To date, in line with the ACC/AHA ^158^ and ESC/EAS ^53^ guidelines, the use of tropical oils to replace vegetable oils rich in unsaturated fatty acids is not indicated.

#### 6.2.10. Dairy

Milk and its products are an important source of calcium and high biological value protein. However, whole milk consumption increases the intake of SFAs, especially myristic acid, which strongly correlates with increased plasma cholesterol concentration. ^159^ However, in the MESA study, dairy consumption was associated with reduced cardiovascular risk ^183^ but not with increased risk of stroke. ^257^ More recently, the European Prospective Investigation into Cancer and Nutrition (EPIC) – Italy study, which included 45,009 participants followed-up for 14.9 years, showed that consumption of 160 mL to 200 mL of milk is related to lower all-cause mortality, but this benefit is lost with an intake greater than 200 mL. ^258^ That study also demonstrated that skim milk consumption is related to lower cardiovascular mortality.

#### 6.2.11. Butter

In a serving of butter (10 g), approximately 51.5% of fatty acids are saturated, with a predominance of palmitic (24%), stearic (10%), myristic (8%), and lauric (2%) acids. Monounsaturated fats are roughly 22% of all fatty acids in butter, while unsaturated fats are only 1.5%. ^259^

A randomized study demonstrated that butter consumption increased plasma concentrations of total cholesterol, LDL-C, and Apo B compared with the consumption of the same amount of unsaturated fat. ^260^ The MESA cohort study, which followed-up for 20 years approximately 6,800 nondiabetic participants with no prior CVD, ^181^ found that highest consumption of butter (up to 5 g/day) was not associated with CVD. Another study showed similar results in older adults. ^261^ Butter consumption was also inversely associated with incidence of type 2 diabetes in a prospective cohort study. ^262^ A systematic review of high-level evidence cohort studies evaluated the effect of butter consumption and showed that a mean intake of 14 g/day was not associated with CVD risks. ^263^

Butter should be consumed according to the recommendations for saturated fat, ie, less than 10% of TEI and even more reduced (less than 7%) for those with hypercholesterolemia (2019 EAS/ESC guideline). ^53^ Also, to avoid gain weight and obesity development, the calories in each product should be considered and consumption should be part of a healthy eating pattern, rich in fruits, vegetables, and whole grains. ^53^

### 6.3. Recommendations for Food Consumption to Control Familial Hypercholesterolemia

The recommendations for food consumption in patients with familial hypercholesterolemia are presented in Table 4 .

**Table 4:** Dietary recommendations for management of familial hypercholesterolemia.

Recommendation	Grade of recommendation	Level of evidence
**Follow a healthy eating pattern:** adjustment of energy intake, inclusion of grains, fruits, vegetables, lean meats, and dairy products with reduced fat content	I	A
**Dietary cholesterol:** < 300 mg/day	IIa	A
**Saturated fatty acids:** < 7% of TEI	I	A
**Trans fatty acids:** should be excluded from diet	III	A
**Chocolate:** if rich in cocoa, it is not related to increased cholesterol	I	B
**Tropical oils:** occasional consumption in minimal amounts	III	B
**Eggs:** moderate consumption, not exceeding daily cholesterol recommendations	IIa	A
**Phytosterols:** 2 g/day provides moderately reduced cholesterol (↓~10%)	I	A
**Fiber:** provides reduction of total and LDL-cholesterol (↓~5%)	I	A

## 7. Pharmacological Treatment of Heterozygous Familial Hypercholesterolemia

The treatment of patients with severe FH is a crucial topic. Because of the complications arising from disease progression and the early development of some of them, treatment should be initiated as soon as possible after diagnosis. The concept of cumulative cholesterol over a lifetime justifies this approach.

### 7.1. Therapeutic Goals for LDL-C

Some high-risk features must be considered: over 40 years of age without prior treatment, smoking, being male, Lp(a) greater than 50 mg/dL (> 125 nmol/L), HDL-C < 40 mg/dL, and percentile of coronary artery calcium (CAC) score calculated by the MESA criteria.

Thus, the proposed goals for treatment of LDL-C are the following: for high-risk FH, LDL-C > 400 mg/dL or LDL-C > 310 mg/dL with one previously described high-risk feature, * or LDL-C > 190 mg/dL with two high-risk features. ^56^ ** Thus, LDL-C should be reduced by at least 50%, with < 70 mg/dL being the ideal level.

Thus, cardiovascular risk and lipid goals are assessed according to presence of ASCVD, major risk factors, or baseline LDL-C levels, and are classified as follows.

#### 7.1.1. Therapeutic Goals in Very High-Risk Patients

- In the case of clinically manifest atherosclerotic disease, defined as previous myocardial infarction, angina pectoris, previous coronary artery bypass graft, ischemic or transient stroke, or intermittent claudication, LDL-C should be reduced by at least 50%, with < 50 mg/dL being the ideal level. ^132^
- In the presence of advanced subclinical atherosclerotic disease diagnosed with a CAC score greater than 100 AU or 75th percentile for age and sex, or coronary CT angiography showing coronary obstructions > 50% or presence of nonobstructive plaques in more than one vessel, LDL-C should be reduced by at least 50%, with < 50 mg/dL being the ideal level.

#### 7.1.2. Therapeutic Goals in High-Risk Patients

- In primary prevention of HeFH in patients with LDL-C > 400 mg/dL, even with no risk factors, LDL-C should be reduced by at least 50%, with < 70 mg/dL being the ideal level.
- In primary prevention of HeFH in patients with additional risk factors, ^56^ LDL-C should be reduced by at least 50%, with < 70 mg/dL being the ideal level. ^132^

Note: If LDL-C > 310 mg/dL with one high-risk feature; if LDL-C > 190 mg/dL with two high-risk features. ^56^

#### 7.1.3. Therapeutic Goals in Intermediate-Risk Patients

In primary prevention of HeFH in patients with no additional risk factors, ^1^ LDL-C should be reduced by at least 50%, with < 100 mg/dL being the ideal level. Periodic reassessment is required to monitor the onset of risk factors.

### 7.2. Pharmacological Treatment

#### 7.2.1. Statins

Statins – hydroxymethylglutaryl-coenzyme A reductase inhibitors – are the first-choice drugs for the treatment of HeFH. Even in the absence of scientific evidence from studies evaluating the benefits of statins used exclusively in HeFH patients, the contribution of HeFH patients to the current understanding of LDL-C metabolism and their significant participation in large clinical trials are fully recognized ^264^ (grade of recommendation: I; level of evidence: C).

The therapeutic goals recommended for HeFH patients, when classified as being at high and very high risk, are ≥ 50% reductions in LDL-C levels from baseline and achievement of the recommended goals according to stratified risk groups. In HeFH, high-intensity statins such as rosuvastatin and atorvastatin at maximum tolerated doses are the preferred options (grade of recommendation: I; level of evidence: A). ^53^

Relevant pharmacological properties of statins are the following: potent and reversible enzyme inhibition, selectivity in hepatocytes, low bioavailability to reduce systemic adverse effects, prolonged elimination half-life, and minimal or no hepatic metabolism to prevent drug-drug interactions. The mechanism of action is based on enzyme inhibition, which, by reducing the endogenous synthesis of intrahepatic cholesterol, stimulates the synthesis and expression of LDL-C receptors, thus increasing the uptake of LDL in hepatocytes and reducing plasma concentrations. ^22^

Statin treatment has been shown to reduce coronary ischemic events, need for coronary artery bypass graft, stroke, and cardiovascular mortality in all subgroups, including those with manifest atherosclerosis, diabetes, or hypertension, older adults, and women, while also reducing total mortality in patients at high and very high cardiovascular risk (grade of recommendation: I; level of evidence: A). The benefits are attributed to reduced LDL-C levels and defined as a therapeutic class effect. ^52^

Statins are safe, and the most frequent adverse effect is myalgia with or without increased creatine kinase (CK). Rhabdomyolysis is the most severe and rarest adverse reaction, whose risk increases when statins are combined with fibrates. The frequency of adverse effects is proportional to the doses used. ^265^

When efficacy is not sufficiently obtained with statin therapy alone, further LDL-C reductions may be attempted by adding adjuvant therapies.

#### 7.2.2. Adjuvant Therapy to Statins

FH patients are at high cardiovascular risk, and high doses of potent statins remain the mainstay of treatment of dyslipidemia for reduction of risk in these patients. ^266^^-^^269^ However, most FH patients will not achieve the goals, despite the use of maximum tolerated statin therapy. In a cross-sectional study of 1,249 patients with confirmed HeFH in the Netherlands, the country with highest rates of FH diagnosis, only 21% had LDL-C < 100 mg/dL, although 96% were on statins. ^270^ Therefore, adding one or more lipid-lowering drugs other than statins will be frequently required to achieve the desired goals. ^271^

##### 7.2.2.1. Ezetimibe

Ezetimibe selectively reduces the intestinal absorption of dietary and biliary cholesterol by acting on the Niemann-Pick C1-Like 1 (NPC1L1) transporter in the enterocyte. After oral administration, ezetimibe is rapidly absorbed (2 to 3 hours) and then undergoes glucuronidation in the liver. This produces an active glucuronide located at the border of the enterocyte that returns to the enterohepatic circulation (20%). The glucuronide conjugate is hydrolyzed and absorbed and is equally effective in inhibiting sterol absorption. This enterohepatic recycling is responsible for a half-life of more than 22 hours, and the drug specifically inhibits the intestinal absorption of dietary and biliary cholesterol but does not affect the absorption of fat-soluble vitamins (A, D, E, K), fatty acids, or bile salts.

Reduced cholesterol influx from the intestine to the liver leads to a compensating increase in the expression of hepatic LDLRs and increased uptake of circulating LDL particles. The final LDL-C reduction obtained with ezetimibe 10 mg/day (single dose recommended), alone or combined with a statin, is 15 to 25%. ^272^ In a meta-analysis of five randomized clinical trials (5,039 patients) in which ezetimibe added to ongoing statin therapy was compared with placebo, the mean LDL-C reduction was 23.6%, with no increase in adverse effects. ^273^ This potentiated lipid-lowering effect is also demonstrated in the population of HeFH patients. ^274^^,^^275^ Although the concerning literature is obviously more limited, the lipid-lowering efficacy of ezetimibe has also been demonstrated in the HoFH population. ^276^

The combined use of statin and ezetimibe was shown to be effective in reducing surrogate outcomes ^277^ and ischemic events that were analyzed as a secondary outcome. ^278^ However, the efficacy of the combination in reducing major cardiovascular events was demonstrated in the IMPROVE-IT study, ^279^ which compared the ezetimibe-simvastatin combination with simvastatin alone in stable patients after an episode of ACS and with LDL-C within the recommended goals. The primary outcome was a composite of cardiovascular death, ACS (nonfatal AMI, unstable angina requiring hospitalization), and nonfatal stroke. Patients receiving ezetimibe plus statin had, after 1 year, a 24% reduction in LDL-C compared with those receiving statin alone. In a mean follow-up of 7 years, the relative risk reduction in the primary cardiovascular outcome, a composite of cardiovascular death, ACS (nonfatal AMI, unstable angina requiring hospitalization), and nonfatal stroke, was 6.4%. This risk reduction was proportional to the LDL-C reduction and was comparable to the risk reduction obtained for a reduction of the same magnitude with statin therapy. ^280^ Therefore, ezetimibe should be used as an adjuvant therapy to high-intensity statins when the latter, at their maximum dose or at the maximum tolerated dose, are not sufficient to achieve the LDL-C goal (grade of recommendation: I; level of evidence: B).

##### 7.2.2.2. PCSK9 Inhibitors

PCSK9 is a protease that regulates the activity of the LDLR and induces it for lysosomal degradation. Thus, by reducing the amount of LDLR on the surface of the hepatocyte and decreasing the activity of these receptors, PCSK9 increases the plasma concentration of LDL-C. ^281^ The use of monoclonal antibodies to prevent PCSK9 from binding to the LDLR is the most effective way of inhibiting the activity of this enzyme. These antibodies bind to the allosteric site of the LDLR and block PCSK9-LDLR binding. Consequently, they increase LDLR recirculation and reduce serum LDL-C levels. Published meta-analyses report a consistent reduction of approximately 50% in serum LDL-C levels in different clinical settings with the antibodies, used alone or combined with the maximum tolerated therapy. ^282^

Two antibodies are available on the Brazilian market, both consisting of a solution in a “pen” ready for injection, which does not allow fractionated doses: evolocumab 140 mg and alirocumab 75 and 150 mg. Both are used similarly, with a subcutaneous injection every two weeks (although evolocumab can also be administered at a dose of 420 mg once monthly). Both drugs were tested in an HeFH setting, in addition to the maximum tolerated statin therapy, combined or not with other lipid-lowering drugs, and showed a similar LDL-C reduction ranging from 50 to 60%. ^283^^-^^285^ Three-hundred FH patients (106 with HoFH, including adolescents aged 14-18 years at inclusion) received evolocumab 420 mg every 4 weeks for a median time of 4.1 years. The LDL-C reduction from baseline to week 12 was 21.2% (-59.8 mg/dL) in patients with HoFH and 54.9% (-104.4 mg/dL) in those with severe HeFH. These results were sustained over time. Of 48 patients with HoFH receiving a dose up-titrated to 420 mg every two weeks, the LDL-C reduction improved from -19.6% at week 12 to -29.7% after 12 weeks of 420 mg every two weeks. ^285^ Evolocumab was also tested in HoFH patients receiving 420 mg subcutaneously (SC) once monthly, with a mean LDL-C reduction of approximately 21%. ^286^ For such reason, evolocumab is also approved for use in HoFH.

PCSK9 inhibitors have been shown to reduce cardiovascular outcomes in high cardiovascular risk populations with established clinical atherosclerotic disease. The FOURIER study ^287^ evaluated 27,564 patients with established CVD (coronary heart disease, cerebrovascular disease, or peripheral artery disease) who, despite being on maximum tolerated therapy, failed to reach the LDL-C goal (< 70 mg/dL). After a median follow-up of 2.2 years, evolocumab was associated with a 15% reduction in the primary outcome, a composite of cardiovascular death, AMI, stroke, unstable angina requiring hospitalization, or need for coronary artery bypass graft. In the ODYSSEY Outcomes study, ^288^ which included 18,924 patients with a recent ACS (1 to 12 months before inclusion in the study) and LDL-C ≥ 70 mg/dL despite being on maximum tolerated dose of statin therapy combined or not with other lipid-lowering drugs, alirocumab reduced the primary composite outcome of death from coronary heart disease, nonfatal AMI, fatal or nonfatal stroke, or unstable angina requiring hospitalization by 15%. The median duration of follow-up was 2.8 years.

Therefore, because of the lipid-lowering efficacy in FH patients and the reduced cardiovascular outcomes in high-risk populations, PCSK9 inhibitors are indicated for patients who, despite being on high-intensity statin therapy or maximum tolerated dose, preferably already combined with ezetimibe, failed to reach the LDL-C goal (grade of recommendation: I; level of evidence: A).

##### 7.2.2.3. Cholestyramine

Cholestyramine is an anion exchange resin that binds to bile acids in the intestine and forms an insoluble complex which is excreted in the feces. With increased excretion, the synthesis of bile acids increases in the hepatocyte at the expense of an elevation in cholesterol synthesis but mainly by an increased expression of LDLRs. This removes LDL-C from circulation, thus reducing plasma LDL-C levels. ^289^ The lipid-lowering effect of cholestyramine may vary, reaching up to a 30% reduction in LDL-C at maximum doses. ^290^ Cholestyramine is available as 4-g packets, and the initial dosage is 4 g daily, with a maximum of 24 g/day, although doses greater than 16 g are hardly tolerated.

The main adverse effects of cholestyramine refer to the digestive system (gastric fullness, nausea), since it may affect intestinal motility and cause constipation and meteorism. The drug reduces the absorption of fat-soluble vitamins (A, D, K, E) and folic acid, sometimes requiring vitamin supplementation. Cholestyramine should be taken 1 hour before or 3 hours after administration of other medications, so that their absorption is not reduced. Cholestyramine was shown to reduce cardiovascular outcomes when used prior to statin therapy. It also reduced the incidence of myocardial infarction by 19% in hypercholesterolemic men over a 7-year follow-up in the Lipid Research Clinics primary prevention study. ^291^

Cholestyramine can be used as an adjuvant therapy when high-intensity statin therapy, preferably already combined with ezetimibe and/or a PCSK9 inhibitor, is not sufficient to achieve the LDL-C goal (grade of recommendation: IIa; level of evidence: B). The drug may be especially useful in children under 8 years of age who are not yet eligible to receive statin therapy and in pregnant women.

## 8. Alternative Therapies for Treatment of Familial Hypercholesterolemia: Partial Ileal Bypass

Partial ileal bypass surgery was first performed for the treatment of hypercholesterolemia in the 1960s and provided a sustained LDL-C reduction for more than 20 years. ^292^ The procedure was evaluated in the Program on the Surgical Control of the Hyperlipidemias (POSCH) trial, which included 838 AMI survivors with a mean LDL-C of 179 mg/dL. Compared with the control group, the participants undergoing surgery showed a 38% reduction in LDL-C and a 35% decrease in the composite outcome of death from coronary heart disease or nonfatal AMI. ^293^

In a small study of 11 patients with HeFH, partial ileal bypass surgery reduced LDL-C by approximately 20% after 2 years. ^294^

The main adverse effect of this surgery is diarrhea (an average of more than three bowel movements per day after the procedure). Renal and biliary lithiasis is another reported adverse event. ^292^^,^^293^

With the introduction of statins in clinical practice in the 1980s-1990s and then of other lipid-lowering drugs, partial ileal bypass surgery was no longer used in the treatment of hypercholesterolemia. The role of this procedure in the management of FH and prevention of CVD under current pharmacological therapy is unknown.

It is worth noting that some contemporary bariatric surgery techniques leading to significant weight reductions, such as Roux-en-Y gastric bypass and biliopancreatic diversion, also provide LDL-C reductions. ^295^

### 8.1. Recommendation

Although partial ileal bypass surgery has been shown to reduce LDL-C and cardiovascular events, this procedure is not routinely recommended for FH patients, given the existence of several other effective, noninvasive, and lower-risk treatment modalities (grade of recommendation: IIB; level of evidence: B).

### 8.2. Plasmapheresis and LDL apheresis

LDL apheresis (LDL-A) and plasmapheresis are two treatment options based on extracorporeal blood filtration. Both involve sessions lasting 2 to 3 hours weekly or fortnightly. The main difference between the two procedures is specificity. In plasmapheresis procedures, there is separation of the patient’s plasma and blood cells, so that the blood cells are retained and mixed in a replacement fluid to return to the patient, while plasma together with proteins (including HDL-C) is discarded. Adverse effects include susceptibility to infections, nausea, hypertension, hypotension, and urticaria. ^296^

In LDL-A procedures, in turn, plasma is not discarded; it passes through a precipitation filter for selective removal of LDL, VLDL, and Lp(a) cholesterol. The most frequent adverse effects include hypotension, anemia, nausea, flushing, headache, and venous access problems. ^297^

There are several methods of apheresis, including dextran sulfate cellulose adsorption, heparin-induced extracorporeal LDL-C precipitation, immunoadsorption, and double filtration plasmapheresis. In a comparison of the available methods, only small differences were observed in their ability to reduce lipid levels. As a rule, these selective methods reduce plasma LDL-C levels by a mean of 50 to 70% after a single treatment. ^298^^,^^299^ The time to return to baseline LDL-C levels ranges from 4 days to 3 to 4 weeks.

Because of its specificity, LDL-A is better tolerated than plasmapheresis; it has a lower rate of adverse effects (2% versus 12%, respectively) and is more effective in reducing LDL-C (60 to 65% versus 50%, respectively). However, LDL-A is less commonly available and costs twice as much. ^300^

### 8.3. LDL Apheresis in Homozygous or Compound Heterozygous Children

The recommended criteria for indication of LDL-A or plasmapheresis following diet combined with optimized pharmacotherapy in homozygous or compound heterozygous children include the following:

- A reduction below 50% from baseline LDL-C or LDL-C levels kept above 360 mg/dL: these criteria are modifiable according to the clinical status of each patient, including but not limited to the progression of atherosclerotic disease. Retrospective and longitudinal follow-up studies demonstrate that LDL-A therapy in children led to a reduction and/or disappearance of cutaneous xanthomas, delayed progression of aortic and supra-aortic valve stenosis, and regression of coronary lesions. ^301^^-^^305^ Diet and pharmacotherapy should be maintained concomitantly with apheresis procedures, since combined statin therapy reduces LDL-C by up to 70%. Statins also delay the rebound effect of increased LDL-C levels post apheresis.
- Age at initiation of LDL-A: cardiovascular prognosis depends on timing of treatment; the earlier the better. The age for initiation of LDL-A is 5 years or over, preferably before 8 years of age. In more severe cases, it can be initiated at an earlier age. ^303^ After 10 years of age, LDL-A has not been shown to be as beneficial in follow-up and retrospective studies. ^303^
- Monitoring the progression of atherosclerosis: the presence of progressive atherosclerosis is one of the criteria for choosing invasive treatments. Imaging tests, such as transthoracic echocardiogram to assess the presence and/or progression of aortic valve disease and aortic arch disease, carotid ultrasound to measure intima-media thickness and atheromatous plaques, and exercise tests should be done at treatment initiation and every 2 years during follow-up. ^303^^,^^304^
- Contraindications for apheresis: bleeding diathesis, resistance to adequate coagulation, and hypersensitivity to heparin.
- Adverse effects: iron deficiency anemia is the most frequent; hypotension and venous access problems are also reported but less frequently.
- Safety: it is a safe and tolerable procedure for children and adolescents in specialized centers. ^306^

Many case studies and clinical evaluations have demonstrated that children who had undergone LDL-A for many years (up to 20 years) developed normally. ^306^^-^^308^

### 8.4. LDL Apheresis in Adult Patients with HoFH or Severe HeFH

For patients with HoFH or severe HeFH, after 6 months with no adequate response ^309^ to maximum drug therapy, which includes statins, ezetimibe, and PCSK9 inhibitors, or drug intolerance, LDL-A is indicated in the following situations:

1. Functional HoFH with LDL-C ≥ 300 mg/dL (or non-HDL-C ≥ 330 mg/dL).
2. Functional HeFH with LDL-C ≥ 300 mg/dL (or non-HDL-C ≥ 330 mg/dL) and one or no risk factors.
3. Functional HeFH with LDL-C ≥ 200 mg/dL (or non-HDL-C ≥ 230 mg/dL) and at least two risk factors or high Lp(a) (≥ 50 mg/dL).
4. Functional HeFH with LDL-C ≥ 160 mg/dL (or non-HDL-C ≥ 190 mg/dL) and presence of clinical or subclinical CVD or risk factors such as diabetes and smoking.

Several clinical trials have confirmed the benefits of LDL-A in preventing and reducing the progression of atherosclerotic disease. ^310^^-^^317^ Other beneficial effects have also been reported, such as improved endothelial function, ^318^ coronary artery vasodilation, ^319^ improved microvascular flow, ^320^ and myocardial perfusion. ^321^ LDL-A is the only treatment that consistently reduces Lp(a) levels by more than 50%. ^322^^,^^323^

Although LDL-A is the most feasible treatment for some severe homozygous and heterozygous patients, the procedure is available in few centers worldwide because of its high cost.

### 8.5. Recommendation for Indication of LDL Apheresis in Adults with HoFH or Severe HeFH and Children with HoFH

Retrospective studies or long-term follow-up cohorts demonstrate that patients with severe HoFH or HeFH hardly achieve the recommended LDL-C goals, despite the maximum doses of optimized therapy combined with weekly or fortnightly LDL-A sessions. Studies demonstrated that, although reducing cholesterol was difficult, patients with lower LDL-C levels had higher survival rates and fewer cardiovascular events than those with higher levels. These benefits were independent of the type of treatment. Therefore, the degree of lipid lowering, as well as its early onset, independently determined clinical outcomes. ^324^ Because LDL-A is the procedure that most reduces LDL-C levels, it is recommended for adults with HoFH or severe HeFH and children with HoFH (grade of recommendation: I; level of evidence: C).

### 8.6. Liver Transplantation

Liver transplantation can be an option for patients with FH refractory to pharmacological treatment ^325^ and is especially indicated for patients with HoFH. However, patients and family members should always be included in the discussion so that the risks and benefits of the procedure are explained (grade of recommendation: IIB; level of evidence: C).

## 9. Treatment Algorithms for Familial Hypercholesterolemia in Adults

To facilitate the therapeutic approach to both heterozygous and homozygous FH in adults (> 18 years), with attention to cardiovascular risk, five treatment algorithms are shown in Figures 7 - 11 .

Figure 7 shows a treatment flowchart for very high-risk HeFH, including goals and proposed therapeutic sequence to achieve these goals. Potent statins, ezetimibe, and PCSK9 inhibitors aimed at lowering LDL-C by > 50% and achieving LDL-C < 50 mg/dL are recommended for attaining goals gradually.

**Figure 7:** Treatment flowchart for heterozygous familial hypercholesterolemia in secondary prevention (very high-risk patients). CAD: coronary artery disease; FH: familial hypercholesterolemia; LDL-C: low-density lipoprotein cholesterol; PAD: peripheral artery disease; SC: subcutaneously; TIA: transient ischemic attack.

In Figure 8 , there is a treatment flowchart for high-risk HeFH with proposed goals and therapeutic sequence to achieve these goals. Potent statins, ezetimibe, and PCSK9 inhibitors aimed at lowering LDL-C by > 50% and achieving LDL-C < 70 mg/dL are recommended for attaining goals gradually.

**Figure 8:** Treatment flowchart for heterozygous familial hypercholesterolemia in primary prevention (high-risk patients). LDL-C: low-density lipoprotein cholesterol; SC: subcutaneously. *Additional risk factors in FH are the following: ^56^ age > 40 years and no treatment, smoking, being male, Lp(a) > 50 mg/dL, HDL-C < 40 mg/dL, hypertension, diabetes mellitus, family history of premature CAD in rst-degree relatives (men aged < 55 years and women aged < 60 years), chronic kidney disease (GFR < 60 mL/min), and BMI > 30 kg/m^2^.

In Figure 9 , a treatment flowchart for intermediate-risk HeFH is shown, including proposed goals and therapeutic sequence to achieve these goals. Potent statins, ezetimibe, and PCSK9 inhibitors aimed at lowering LDL-C by > 50% and achieving LDL-C < 100 mg/dL are recommended for attaining goals gradually.

**Figure 9:** Treatment flowchart for heterozygous familial hypercholesterolemia in primary prevention (intermediate-risk patients). LDL-C: low-density lipoprotein cholesterol; SC: subcutaneously. *Additional risk factors in FH are the following: ^56^ age > 40 years and no treatment, smoking, being male, Lp(a) > 50 mg/dL, HDL-C < 40 mg/dL, hypertension, diabetes mellitus, family history of premature CAD in rst-degree relatives (men aged < 55 years and women aged < 60 years), chronic kidney disease (GFR < 60 mL/min), and BMI > 30 kg/m^2^.

Figure 10 shows a treatment flowchart for HoFH in secondary prevention, including therapeutic goals and proposed therapeutic sequence to attain these goals. The purpose is to achieve an LDL-C reduction > 50% and an LDL-C goal < 50 mg/dL. Potent statins and ezetimibe are recommended as first-line therapy, in addition to PCSK9 inhibitors, if an LDL-C reduction < 30% is sufficient to achieve goals and the patient does not carry LDLR-null mutations in both alleles; if LDL-C remains > 50 mg/dL or if the patient carries LDLR-null mutations in both alleles, additional therapies such as microsomal triglyceride transfer protein (MTP) inhibitor (lomitapide) may allow achieving goals with an additional 50% reduction in LDL-C. Other additional therapies such as apheresis and liver transplantation are included in the flowchart. Patients should always be referred to and followed-up by a specialist.

**Figure 10:** Treatment flowchart for homozygous familial hypercholesterolemia in secondary prevention. CAD: coronary artery disease; HoFH: homozygous familial hypercholesterolemia; LDL-C: low-density lipoprotein cholesterol; MTP: microsomal triglyceride transfer protein; PAD: peripheral artery disease; SC: subcutaneously; TIA: transient ischemic attack.

Figure 11 shows a treatment flowchart for HoFH in primary prevention, with therapeutic goals and a proposed therapeutic sequence to achieve these goals. The purpose is to achieve an LDL-C reduction > 50% and an LDL-C goal < 70 mg/dL. Potent statins and ezetimibe are recommended as first-line therapy, in addition to PCSK9 inhibitors, if an LDL-C reduction < 30% is sufficient to achieve goals and the patient does not carry LDLR-null mutations in both alleles; if LDL-C remains > 70 mg/dL or if the patient carries LDLR-null mutations in both alleles, additional therapies such as MTP inhibitor (lomitapide) may allow achieving goals with an additional 50% reduction in LDL-C. Other additional therapies such as apheresis and liver transplantation are included in the flowchart. Patients should always be referred to and followed-up by a specialist.

**Figure 11:** Treatment flowchart for homozygous familial hypercholesterolemia in primary prevention. CAD: coronary artery disease; HoFH: homozygous familial hypercholesterolemia; LDL-C: low-density lipoprotein cholesterol; MTP: microsomal triglyceride transfer protein; PAD: peripheral artery disease; SC: subcutaneously; TIA: transient ischemic attack.

## 10. Familial Hypercholesterolemia in Children

### 10.1. Screening

Suspecting that a child or adolescent has FH is extremely important because, according to estimates, one child is born with FH every minute worldwide. However, diagnosing the disorder in this group is a challenge in clinical practice. ^59^

Several factors contribute to underdiagnosis. Notably, there is a lack of knowledge regarding ideal age recommendations for laboratory screening as well as a clinical tendency to assess lipid profiles in overweight/obese or diabetic children (FH children are mostly healthy weight and generally do not have diabetes). Also, there are failures in the process of obtaining a detailed history during the first childcare visit, which should include a history of premature CVD or known high cholesterol levels in first-degree relatives.

Thus, HeFH in asymptomatic children is generally detected with a serum lipid profile test, when there is a close relative with a confirmed diagnosis of FH. ^57^ In this context, except for specific conditions in which early screening is recommended, laboratory screening for dyslipidemia, including FH, is universally recommended in children and adolescents aged 10 years or over (grade of recommendation: I; level of evidence: A).

A lipid profile test is the initial laboratory screening tool in this group, based on the analysis of total cholesterol and LDL-C levels. However, it should be noted that:

1. In universal screening, a lipid profile test does not require fasting because dietary status has no influence on concentrations of total cholesterol, LDL-C, and non-HDL-C.
2. An isolated measure of LDL-C is not sufficient for diagnosing dyslipidemia in children or adolescents. Studies demonstrate a wide variation in LDL-C when measurement is repeated after at least 2 weeks. ^326^

### 10.2. Lipid Profile Levels for Suspected Familial Hypercholesterolemia

1. A laboratory screening algorithm for children or adolescents with FH is described in Figure 12 (grade of recommendation: I; level of evidence: A).
2. It should be noted that, if LDL-C levels are > 400 mg/dL, severe HeFH or HoFH should be suspected ^59^ (grade of recommendation: I; level of evidence: A).

**Figure 12:** Laboratory screening algorithm for familial hypercholesterolemia in children and adolescents. Adapted from Expert panel on integrated guidelines for cardiovascular health and risk reduction in children and adolescents; ^224^ Wiegman A, et al. ^327^ CAD: coronary artery disease; FH: familial hypercholesterolemia; LDL-C: low-density lipoprotein cholesterol; TC: total cholesterol. *Improbable FH: absence of laboratory criteria for FH does not mean that a different dyslipidemia is not present. Children or adolescents with lipid profile levels outside the reference range for their age group should undergo a clinical assessment. Secondary causes should be assessed in this age group: kidney or thyroid dysfunction, HIV, autoimmune diseases, diabetes, and obesity, among others. **Lifestyle changes and nutritional guidance; see nonpharmacological therapy section. ***When available, genetic testing should be provided. ****Clinical assessment: clinical signs such as xanthomas, xanthelasma, corneal arcus, and thickened tendon should be assessed. Rule out non-FH clinical conditions that co-occur with hypercholesterolemia. Dutch scores should be used at this stage. *****See peculiarities in the management of patients with HoFH section

### 10.3. Factors Related to Increased Cardiovascular Risk

FH patients are generally considered to be at high cardiovascular risk, and this risk may increase depending on the presence of additional factors. ^139^^,^^328^^,^^329^ These include the following: reduced HDL-C levels, high triglyceride levels, high Lp(a) levels, and lifestyle factors such as smoking, atherogenic diet, and sedentary behavior. ^56^

Also, specific conditions related to premature CAD should be considered in the pediatric group, including type 1 and type 2 diabetes, chronic kidney disease, heart transplant, Kawasaki disease, chronic inflammatory disease, human immunodeficiency virus (HIV), nephrotic syndrome, and previous cancer treatment. ^224^

#### 10.3.1. Screening Tools for Diagnosis of Familial Hypercholesterolemia in Addition to Laboratory Lipid Profile

Given the severity of cases and the early development of cardiac lesions, there is a need to monitor complications, ideally at diagnosis, which usually happens after the age of 2 years. It consists of determining markers of accelerated progression of atherosclerosis, cardiac function, valvular and aortic root lesions.

Monitoring of atherosclerosis aims to determine the arterial structure and function periodically, which can be used for decision-making regarding drug management and LDL-C goals. IMT has been considered the ideal and easiest method to conduct a structural assessment of the vessel wall in FH children, especially those over 8 years of age, based on internationally validated criteria for age ^330^ (grade of recommendation: IIa; level of evidence: B). Recently, computerized axial tomography has been shown to be even more sensitive, but this procedure seems to be reserved for patients with HoFH ^144^ (grade of recommendation: IIa; level of evidence: C).

Several methods have been described to assess endothelial function in pediatric patients, including flow-mediated distensibility (with digital tonometry), post-occlusive reactive hyperemia, and pulse wave velocity. There is no consensus on which of them is more sensitive and specific, and use of the locally validated method is suggested ^331^^,^^332^ (grade of recommendation: IIa; level of evidence: C).

Global longitudinal strain of the left ventricle seems to be decreased in children with severe dyslipidemias; for this reason, their ventricular function should be periodically assessed, initially with conventional echocardiography. Assessing cardiac valves and aortic root is also important to rule out the possibility of dysfunctions, dilations, or calcifications. Computerized axial tomography has been shown to be the most sensitive method ^333^ (grade of recommendation: IIa; level of evidence: C).

### 10.4. Treatment

Indication, management, and goals to be achieved with the introduction of nonpharmacological and pharmacological treatments are described in Figure 13 (grade of recommendation: II; level of evidence: A).

**Figure 13:** Management algorithm for familial hypercholesterolemia in children and adolescents. Adapted from Expert panel on integrated guidelines for cardiovascular health and risk reduction in children and adolescents; ^224^ Wiegman A, et al. ^327^ CAD: coronary artery disease; CV: cardiovascular; FH: familial hypercholesterolemia; LDL-C: low-density lipoprotein cholesterol. *All children should undergo a nonpharmacological therapy. See nonpharmacological therapy section. **In children under 8 years of age, the decision on drug therapy should be individualized, e.g. for cases of severe heterozygous or homozygous FH. ***If LDL-C > 130 mg/dL with no atherosclerotic involvement, choose nonpharmacological treatment. ****See factors related to increased cardiovascular risk section.

#### 10.4.1. Nonpharmacological Treatment

Lifestyle changes are a key step in the treatment of children with FH. Management of dyslipidemia should preferably be supported by an interdisciplinary team. Combating both active and passive smoking should be combined with close monitoring of hypertension and overweight or obesity in all patients, right from diagnosis.

##### 10.4.1.1. Diet

Diet is known to have a partial effect on FH because of pathophysiological characteristics. Nonetheless, diet is recommended at diagnosis because it has a significant effect on lipoproteins and apolipoproteins, and also there is evidence that diet decreases the inflammatory pattern of these patients : the higher the intake of PUFAs, the lower the inflammation, and the higher the intake of energy and cholesterol, the higher the inflammation. ^334^^,^^335^

As is the case with all children in the general population, intake of SFAs should be reduced and intake of MUFAs and PUFAs should be increased, mostly from plants (canola oil has the best results). Other recommendations are elimination of trans fatty acids, increased intake of fruits and vegetables (maximizing fiber consumption, but preferably from food sources), and restriction of intake of added sugar ^334^^,^^335^ (grade of recommendation: IIa; level of evidence: B).

Even in children, initial dietary treatment should follow type I diet guidelines, with TEI calculations according to amounts of calories, macronutrients, and micronutrients required to allow adequate growth and development ^334^^,^^335^ (grade of recommendation: I; level of evidence: B).

If the response to type I diet is inadequate, a type II diet is introduced, and management by a nutrition expert (a dietitian or a physician nutrition specialist) is strongly recommended, since the risk of malnutrition (lack of macronutrients or micronutrients, especially fat-soluble vitamins) is remarkably high, especially in younger patients on ezetimibe or anion exchange resins, who are at the highest risk ^336^ (grade of recommendation: I; level of evidence: B).

##### 10.4.1.2. Physical Activity

For children aged 6 to 17 years, vigorous-intensity aerobic activity for at least 60 minutes daily is recommended. Muscle-strengthening and bone-building activities at least three times weekly are also recommended (grade of recommendation: IIa; level of evidence: B). ^337^^,^^338^

Preschoolers should remain active throughout the day to have better conditions of growing and developing a repertoire of motor skills. Being active for at least 3 hours daily should be aimed (grade of recommendation: IIa; level of evidence: B).

Although there is no consensus on the level of physical activity required to control the impact of dyslipidemia on children, active behavior is known to improve endothelial function and reduce IMT ^339^ (grade of recommendation: IIa; level of evidence: B).

Patients with dyslipidemia, especially FH, may need a comprehensive, interdisciplinary cardiopulmonary and metabolic rehabilitation program. In children, initial supervised physical activity is safer because this type of approach is known to provide better control of cardiovascular risk factors and regression of subclinical atherosclerosis ^340^ (grade of recommendation: IIa; level of evidence: A).

#### 10.4.2. Pharmacological Treatment

Based on strict fulfillment of the criteria described below and after lifestyle changes, lipid-lowering therapy should be initiated at the age of 8 years. The treatment in children under 8 years of age may be indicated if the case is severe and after individual assessment. The aim is to reduce LDL-C by at least 50% and, if possible, achieve < 110 mg/dL (desired) or at least 130 mg/dL (borderline), in addition to reducing xanthomatosis and preventing the onset of CAD (grade of recommendation: I; level of evidence: A).

##### 10.4.2.1. Statins

Statins significantly decrease total cholesterol, LDL-C, and Apo B, with no apparent significant occurrence of adverse effects related to sexual development and muscle or liver toxicity. The drugs can be used in children aged 8 years or over (in individual cases, they can be prescribed for those under 8 years of age). ^59^^,^^341^^,^^342^ Statins can decrease LDL-C by approximately 30% and increase HDL-C by 5% ^343^^-^^348^ (grade of recommendation: IIa; level of evidence: A).

Recent publications have shown that statins, in addition to reducing total cholesterol and LDL-C in children, improved endothelial function, decreased carotid IMT, and led to regression of xanthomas (grade of recommendation: IIa; level of evidence: B). ^349^ The usual doses of lipid-lowering drugs for children and adolescents are described in Chart 3 .

**Chart 3:** Doses of lipid-lowering drugs for children and adolescents

Drug	Doses (mg)
Lovastatin	10 to 40
Pravastatin	10 to 40
Simvastatin	10 to 40
Rosuvastatin	5 to 40
Atorvastatin	10 to 40
Cholestyramine *	4 to 16 *
Ezetimibe	10

* In grams.

Doses higher than those described below can be used after individual risk assessment in children and adolescents. In this group, initial use of the lowest possible statin dose is suggested (grade of recommendation: IIa; level of evidence: C). When the LDL-C goal is not achieved with the maximum tolerated statin dose, ezetimibe is indicated as a second choice for combined treatment (grade of recommendation: I; level of evidence: B).

##### 10.4.2.2. Cholesterol Absorption Inhibitors

Ezetimibe monotherapy lowers LDL-C levels by approximately 28% in children with HeFH. Monotherapy is recommended for those aged 5 years or over, while combined therapy with a statin is indicated for those over 8 years of age ^342^^,^^350^ (grade of recommendation: IIb; level of evidence: C).

##### 10.4.2.3. Bile Acid Sequestrants

Bile acid sequestrants can be used at any age. Sequestrants used as monotherapy lower LDL-C levels by approximately 10 to 15%. They can also be combined with statins or ezetimibe, but at different times of administration. Because of the risk of malnutrition related to fat-soluble vitamins, nutritional monitoring and supplementation according to objective deficiency criteria are recommended. ^351^ To improve palatability and increase children’s tolerance, apple juice can be given together with the sequestrant (grade of recommendation: I; level of evidence: B).

##### 10.4.2.4. Supplements

Supplementation with 1.2 to 1.5 g of phytosterols can lower total cholesterol and LDL-C levels in HeFH children by approximately 10% ^352^ (grade of recommendation: IIb; level of evidence: B). Importantly, omega-3 supplements, such as those found in fish oil products, are not recommended for FH children because they may increase LDL-C levels ^329^ (grade of recommendation: III; level of evidence: B).

#### 10.4.3. New Treatments

This group consists of medications such as lomitapide, mipomersen, and PCSK9 inhibitors (alirocumab and evolocumab). Evolocumab 420 mg SC every 4 weeks was studied in a pediatric population with HeFH (n = 170) aged 10 to 17 years, not controlled for standard treatment including statin and/or ezetimibe. Patients were randomly allocated to receive evolocumab (n = 104) or placebo (n = 53). Evolocumab reduced LDL-C at week 24 by 44.5%, with an absolute reduction of 77.5 mg/dL. There were reductions in other lipid variables. ^353^ Alirocumab was evaluated in the Odissey Kids dose-finding study, ^354^ but the study data are yet to be completed.

Although some of these drugs have been approved for the adult population, their use in the pediatric population should be considered on a case-by-case basis ^59^ (grade of recommendation: IIa; level of evidence: C). To date, however, published long-term safety data are based on adult samples (age of 18 years or over), with limited evidence on children with FH. ^355^^-^^357^

#### 10.4.4. Particularities in the Management of Homozygous Familial Hypercholesterolemia

The subgroup representing the greatest management challenge and also with the greatest impact on treatment during childhood consists of children and adolescents with HoFH. Because they have a homozygous mutation in the *LDLR* gene or additional genetic defects, phenotypically characterized by very high LDL-C concentrations, intervention is needed at diagnosis, regardless of age, to prevent atherosclerotic manifestations, which sometimes appear in the first decade. Because of pathophysiological characteristics, anion exchange resins are often ineffective, especially in homozygous patients with LDLR-null mutations in both alleles. ^351^ Figure 14 shows the algorithm used to manage such children and adolescents ^358^ (grade of recommendation: IIa; level of evidence: C). These patients should be referred to a lipid specialist.

**Figure 14:** Treatment algorithm for homozygous familial hypercholesterolemia in children and adolescents. ^358^ FH: familial hypercholesterolemia.

In adolescents with severe, clinically manifest atherosclerosis, coronary artery bypass graft is indicated. ^359^^,^^360^ In case of aortic disease due to severe dyslipidemia, pulmonary autograft (Ross-Konno procedure) ^361^ may be an interesting substitute in adolescents because of its durability (grade of recommendation: IIb; level of evidence: C).

## 10.5. Treatment Monitoring

Continuous use of single or combined drugs is recommended ^362^ (grade of recommendation: I; level of evidence: C). A large systematic review of randomized controlled trials evaluating the use of statins in children and adolescents demonstrated that the risk of adverse events in this group was similar to that in adults treated with statins, at least in the short term. The reviewed studies analyzed the effect of statin therapy on sexual development, growth, nutrition, and liver or kidney toxicity. In most parameters, there was no statistically significant difference between the treatment and placebo groups. No severe adverse events were reported. Elevations in liver transaminases and creatine phosphokinase (CPK), which are of particular concern in adults, did not differ in the study groups. ^362^

Baseline assessment of CPK, transaminases (TGO, TGP), and HbA1C (for the risk of diabetes associated with statins) is recommended. Laboratory monitoring should be conducted every 4 to 8 weeks, until the measurements have stabilized, and then every 6 months ^59^^,^^329^ (grade of recommendation: I; level of evidence: C). For patients with symptoms possibly related to statins, laboratory monitoring is recommended while symptoms are present, which can help the pediatrician decide whether there is indeed a relationship with the medication. Also, adolescents should be oriented about contraceptive methods because of the potential teratogenicity of statins ^331^ (grade of recommendation: I; level of evidence: C).

If laboratory abnormalities (transaminase levels > 3-fold higher; and/or CPK levels > 3- to 10-fold higher in asymptomatic patients; or CPK levels > 10-fold higher in asymptomatic patients) are detected, statin therapy should be discontinued, and a new test should be done after 2 weeks. With normal laboratory levels, statin therapy can be resumed with proper monitoring ^331^ (grade of recommendation: I; level of evidence: C).

## 10.6. Psychological Aspects

Pharmacological treatment does not seem to impact quality of life or anxiety in children with FH. Approximately 40% of children suffer because they have the condition, but lipid-lowering drugs provide greater safety in around 60%. Over 50% are on a diet, and 79% of parents suffer because their children have FH ^363^ (grade of recommendation: IIb; level of evidence: B).

## 11. Treatment of Familial Hypercholesterolemia during Pregnancy

During pregnancy and lactation, the therapeutic options for FH are quite limited, since statins, ezetimibe, PCSK9 inhibitors, and nicotinic acid should not be prescribed in order to prevent the potential adverse fetal effects which have been associated with these agents (categories X, C, B, and C, respectively). This may be a cause for concern because plasma lipid levels are usually increased during pregnancy (increases of 25 to 50% in cholesterol and 150 to 300% in triglycerides), in addition to quite high baseline cholesterol concentrations due to FH. ^364^

Other lipid-lowering medications, specifically resins, may be used when there is a clear need to maintain drug therapy because of their potential benefits. Resins, such as colesevelam and cholestyramine, are category B agents in pregnancy and lactation and, therefore, can be considered for the treatment of FH in these situations, provided they are used under medical supervision. ^365^ LDL-A is a treatment modality which can also be used in special cases, when the patient’s cardiovascular risk, in the absence of treatment, is very high, such as in patients with HoFH or HeFH and severe atherosclerotic disease. ^366^

Women with FH of childbearing potential who wish to become pregnant should receive pre-pregnancy counseling and discontinue statins, ezetimibe, and nicotinic acid for at least four weeks before stopping the contraceptive method. Importantly, oral contraceptives are generally not contraindicated for most women with FH ^367^ and do not affect statin efficacy. ^368^ Women at increased risk of cardiovascular events should discuss about contraceptive methods other than oral contraceptives. ^367^

In the case of unplanned pregnancy, patients should discontinue lipid-lowering drugs immediately and search for obstetric care. Few studies have evaluated women with FH who became pregnant while on statin therapy, with controversial results regarding the incidence of fetal malformations. In a case series reported by the Food and Drug Administration (FDA) in 2004, 52 selected cases of gestational exposure to statins were evaluated, and 20 consisted of structural birth defects, especially neurological and skeletal defects. ^369^

The relative lack of safe and effective treatments to reduce plasma cholesterol levels in these pregnant patients is associated with concerns about adverse effects caused by hyperlipidemia itself. Indeed, some studies suggest that pregnant women with high cholesterol levels are at increased risk of preterm birth. ^370^^,^^371^ A recent Norwegian study evaluating 2,319 births of 1,093 women with FH detected no differences in preterm births between women genetically diagnosed with FH and women in the general population. ^364^ Concerning low birth weight, there also seems to be no significant difference between newborns of women with or without FH. The frequency of congenital malformations in newborns of women with FH does not seem to be higher compared with that of women in the general population (3.3 and 3.2%, respectively). Toleikyte et al. ^364^ also found no differences in preterm birth, low birth weight, and malformations according to different types of genetic mutation.

Although most available studies do not demonstrate significant associations between adverse fetal events and FH, pregnant women with the disease should be monitored jointly by a lipid specialist and an obstetrician. Attention should be given to possible valve diseases, particularly aortic stenosis, and early coronary heart disease in these patients. ^372^ From an obstetric point of view, the investigation of uteroplacental vascular insufficiency is also important. ^373^^,^^374^

### 11.1. Conclusions

Lipid-lowering medications in pregnant women with FH including statins, ezetimibe, nicotinic acid, fibrates, and PCSK9 inhibitors are not recommended (grade of recommendation: III; level of evidence: B). Resins (grade of recommendation: IIB; level of evidence: B) and apheresis (grade of recommendation: IIB; level of evidence: B) can be used during pregnancy.

### 11.2. FDA Drug Classification for Possible Fetal Effects

- Category A: adequate, well-controlled studies have shown no risk to the fetus in the first trimester of pregnancy (and there is no evidence of risk in the following trimesters).
- Category B: animal studies have shown no risk to the fetus, and there are no adequate, well-controlled studies in pregnant women.
- Category C: animal studies have shown adverse effects on the fetus, but there are no adequate, well-controlled studies in pregnant women.
- Category D: there is evidence of risk to the fetus based on adverse reaction data from human studies or marketing or investigative experience. The benefits of using the drug in pregnant women may outweigh the risks in some situations.
- Category X: studies in animals or humans have demonstrated fetal abnormalities, and/or there is evidence of fetal risk based on adverse effect data from marketing or investigative experience. The risks of using the drug in pregnant women clearly outweigh the potential benefits.

## 12. Lipid-lowering Therapies for Severe Forms of Familial Hypercholesterolemia

FH is a difficult-to-treat condition that causes cardiovascular events at an early age. There are two possible presentations: HeFH and HoFH.

The LDL-C goal recommended in guidelines is generally not achieved with conventional therapies, despite the maximum doses of high-potency statins, requiring then the introduction of adjuvant therapy. Conversely, a 50% reduction in LDL-C, another recommended goal, can be achieved with high-potency statins. ^9^ Individualized goals for HeFH and HoFH according to the respective risk categories were discussed in sections 7 and 9.

Lipid-lowering drugs added to statins are generally required in the treatment of FH. Despite their adverse effects, they are used to reduce LDL-C and, especially, robust cardiovascular outcomes, such as fatal or nonfatal AMI, fatal or nonfatal stroke, and cardiovascular death. These drugs include:

- Anti-PCSK9 monoclonal antibodies.
- MTP inhibitor (lomitapide).
- Antisense oligonucleotide.

### 12.1. Anti-PCSK9 Monoclonal Antibodies in Familial Hypercholesterolemia

In classical Mendelian genetic studies, researchers found a region on chromosome 1 that was linked to the presence of FH, responsible for the transcription of the *PCSK9* gene, whose polymorphism transmits the autosomal codominant form of the disease. ^16^^,^^375^ Subsequent mechanistic studies demonstrated that PCSK9 acts as a chaperone for the LDLR and that, once bound to the receptor, it blocks the conformational change required to prevent its degradation in the lysosome. ^376^ Consequently, the LDLR, which can recirculate approximately 100 times, is degraded early, and this reduces LDL removal from the bloodstream. Consistently, PCSK9 gain-of-function mutations are associated with increased LDL-C and cardiovascular risk, while loss-of-function mutations are associated with reduced LDL-C and cardiovascular risk. ^377^^,^^378^

Monoclonal antibodies were developed to reduce the bioavailability of PCSK9, thus preventing it from binding to the LDLR. Evolocumab and alirocumab have been approved for clinical use. Both are fully human monoclonal antibodies administered SC. Evolocumab can be administered at a dose of 140 mg every two weeks or 420 mg once monthly. In both dosing regimens, evolocumab reduces LDL-C by approximately 60%, when administered alone or combined with other lipid-lowering drugs. ^377^^,^^379^

A similar reduction was observed in HeFH patients; ^283^^,^^284^ however, the reduction achieved in HoFH patients was 38%. ^286^ The degree of response will naturally depend on the type and number of mutations. In those with two defective (mutant) alleles (LDL uptake: 2 to 25%), the treatment can reduce LDL-C by up to 47%. ^286^ In those with one defective allele and one negative allele, ie, with no LDLR production (LDL uptake < 2%), the maximum expected effect is 25%. ^286^ Finally, in those with two negative alleles (no LDL uptake), no response is obtained with treatment. ^286^ Data from the TESLA B study were expanded by an interim analysis of the TAUSSIG study, which included 106 patients with HoFH. ^380^ In this open study, after 12 weeks of monthly use of evolocumab 420 mg, the mean reduction in LDL-C was 21% (standard deviation: 24%). Similar to the TESLA B study, response variability was partially dependent on the genetic defect causing the FH phenotype. Homozygotes with defects in the *APOB* or *LDLRAP1* genes (causing autosomal recessive hypercholesterolemia with a phenotype similar to that of HoFH) had mean reductions of 47 and 15%, respectively. Responses were sustained for up to 4 years of treatment, and the most frequent adverse events were symptoms of nasopharyngitis and common flu.

Therefore, evolocumab therapy should be attempted in HoFH patients, except in those homozygous for LDLR-null alleles. However, they will likely require additional therapies.

Alirocumab was tested at the doses of 75 mg and 150 mg every two weeks. The expected LDL-C reductions are 45 to 50% with the lowest dose and 60% with the highest dose. ^284^^,^^381^^,^^382^ With the 300-mg monthly regimen, alirocumab reduces LDL-C by 55 to 60%. ^383^ Similar to evolocumab, alirocumab reduces LDL-C in HeFH patients by approximately 40 to 60%. ^384^ Equally consistent, LDL-C reductions in HoFH patients are genotype-dependent and range from 7 to 64%. ^385^ Alirocumab is approved for use in HoFH by the FDA, and needs approval for use in the European Medicine Agencies (EMA) and Brazil (ANVISA).

Three randomized, double-blind, placebo-controlled cardiovascular trials evaluated the efficacy of PCSK9 inhibitor therapy for the risk of cardiovascular events. Although none of these trials targeted FH patients, their results can be extrapolated to this population. In the FOURIER trial, evolocumab was compared with placebo in 27,564 patients with clinically manifest atherosclerotic CVD and LDL-C ≥ 70 mg/dL despite optimized lipid-lowering therapy. ^386^ From a baseline LDL-C level of 92 mg/dL, patients receiving evolocumab had a reduction to a mean level of 30 mg/dL, which was maintained over time (median follow-up: 2.2 years). ^287^ Evolocumab therapy reduced the risk of the composite outcome of cardiovascular death, myocardial infarction, stroke, coronary revascularization, or unstable angina requiring hospitalization by 15% (HR 0.85; 95% CI 0.79 to 0.92). The main secondary outcome, a composite of cardiovascular death, myocardial infarction, or stroke, was reduced by 20% (HR 0.80; 95% CI 0.73 to 0.88). ^287^ Similar to studies of statin therapy, clinical benefits progressively increased with time of treatment. ^287^ In a neurocognitive substudy, 1,974 patients from the FOURIER study were evaluated before and after the intervention to assess safety for cognitive function. ^387^ No differences were observed in cognitive function between groups, and there was no association between LDL-C levels and cognitive changes.

In the SPIRE-1 and SPIRE-2 studies, ^388^ bococizumab was administered to 27,438 patients with a previous cardiovascular event or a history of diabetes, chronic kidney disease, or peripheral vascular disease. The study was terminated prematurely after the investigators found that the effect of bococizumab was not sustained because of the development of neutralizing antibodies. ^389^ The baseline LDL-C level was 109 mg/dL, and 85% of participants were receiving high-intensity statin therapy. Bococizumab lowered LDL-C by 59% at 14 weeks, but this effect reduced to 38% at 2 years. ^389^ In the SPIRE-2 study, which had a longer follow-up, bococizumab reduced by 21% (HR 0.79; 95% CI 0.65 to 0.97; p = 0.02) the risk of the primary composite outcome of cardiovascular death, myocardial infarction, stroke, or coronary revascularization. ^390^ A SPIRE substudy showed that the reduction in cardiovascular risk among participants with and without FH was not heterogeneous with the use of bococizumab. ^390^

Finally, the ODYSSEY Outcomes study enrolled 18,924 patients 1 to 12 months after myocardial infarction or unstable angina for alirocumab treatment or placebo. ^391^ Alirocumab dose was up-titrated to provide an LDL-C level of 25 to 50 mg/dL, and the drug was discontinued if LDL-C level was persistently lower than 15 mg/dL. Alirocumab lowered LDL-C by 57% within 4 weeks, but this effect progressively reduced to 36% by the end of the study, presumably because of the drug titration regimen. ^288^ The primary composite outcome of cardiovascular death, myocardial infarction, ischemic stroke, or unstable angina requiring hospitalization was reduced by 15% (HR 0.85; 95% CI 0.78 to 0.93; p = 0.003). ^288^ A 15% reduction was observed (HR 0.85; 95% CI 0.73 to 0.98) in all-cause mortality with alirocumab treatment compared with placebo. ^288^ However, as there was no significant reduction in two hierarchically important outcomes in this analysis, ie, death from cardiovascular causes and death from coronary heart disease, the results were found to be of exploratory value, not evidence.

### 12.2. Microsomal Triglyceride Transfer Protein Inhibitor (Lomitapide)

MTP is a lipid transfer protein found in the endoplasmic reticulum of hepatocytes and enterocytes which acts on the assembly of lipoproteins containing Apo B. ^392^

Lomitapide is a small molecule that inhibits MTP, thus reducing the formation of chylomicrons in the intestine and VLDL in the liver. It is used in combination with statin therapy to reduce LDL-C concentrations, especially in FH patients.

Because VLDL is the metabolic precursor of LDL, plasma LDL concentrations are reduced. ^393^ In the absence or dysfunction of MTP, similar to what occurs in recessive hypobetalipoproteinemia or abetalipoproteinemia, there is no production of VLDL and, consequently, of other lipoproteins containing Apo B, such as LDL, IDL, and Lp(a). Lomitapide is currently approved by the FDA and the European Medicines Agency (EMA) for use as an adjuvant therapy in adults with HoFH; however, use in children has been documented. ^394^ Recently, lomitapide was approved in Brazil in 2020 by the National Health Surveillance Agency (Agência Nacional de Vigilância Sanitária, ANVISA) for use in adults with HoFH.

Lomitapide is administered orally at an initial dose of 5 mg/day and may reach 60 mg/day. The dose should be individualized according to therapeutic goals and individual response and tolerance to treatment. A phase 3 study of HoFH patients treated at initial doses of 5 mg/day and up-titrated to 60 mg/day, combined with the current therapy, showed additional reductions of 50% in LDL-C and 49% in Apo B. ^395^ Changes in HDL-C and Lp(a) concentrations have not been described with maintenance of lomitapide treatment after 78 weeks, except for slight variations in HDL-C. ^396^

A phase 3 study consisting of 26 weeks of lomitapide treatment evaluated the achievement of European Atherosclerosis Society goals and the occurrence of major adverse cardiovascular events. The LDL-C goals < 100 mg/dL and < 70 mg/dL were achieved by 51% and 28% of participants, respectively, within 26 weeks. ^397^ In the extension study of patients who remained on lomitapide after 176 weeks (N = 19), 74% achieved LDL-C < 100 mg/dL and 58% achieved LDL-C < 70 mg/dL in at least one measurement. The LDL-C level achieved with lomitapide treatment was 166 mg/dL. There were two cardiovascular events, ie, one cardiac death and one coronary artery bypass graft, equivalent to 1.7 events per 1,000 patient-months of treatment. These numbers are much lower than those observed in the cohorts of FH patients before the use of new therapies. ^398^

A real-world study of 18 patients with HoFH undergoing adjuvant lomitapide treatment at a mean dose of 19 mg/day in a follow-up of 32.3 ± 29.7 months showed an LDL-C reduction of 68.2 ± 24.8%. At the final visit, 60% of patients achieved LDL-C < 100 mg/dL and 46.6% achieved LDL-C < 70 mg/dL; 80% of patients no longer needed LDL-A because of their achievements. LDL-C reduction varied considerably (13-95%), regardless of genotype. ^392^ During follow-up, 53.3% of patients had adverse events, but none were severe. There was no increase in transaminases > 5 times the ULN, and no patient discontinued the medication because of adverse events. Five patients underwent liver ultrasound and fibroscan or nuclear magnetic resonance imaging with spectroscopy, and none had evidence of liver damage. ^399^

Five-year registry data on HoFH patients receiving lomitapide (N = 187) were consistent for efficacy and safety in phase 3 studies, despite the use of a lower dose (10 mg vs 40 mg) in phase 3 studies. There were no new safety findings, and the incidence of adverse events, serious adverse events, and alanine aminotransferase elevations was lower than that in phase 3 studies, which is probably related to the lower dose used. ^400^

The most common adverse effects involved the gastrointestinal tract, such as nausea, flatulence, and diarrhea. They can be minimized by reducing fat intake or by using drug titration. ^398^ Increased transaminase levels have been described in some patients, but they are generally reversible with drug reduction or discontinuation, or even transient with maintenance of treatment.

In most cases, concomitant elevation of bilirubin and alkaline phosphatase and onset of symptoms have not been described. However, the most concerning adverse effect of lomitapide, because of its mechanism of action, is hepatic steatosis, which is dose-dependent and attenuates with a reduction in daily dose. It can be detected especially by magnetic resonance imaging. Another relevant adverse effect is steatorrhea. ^397^^-^^401^

Studies using magnetic resonance imaging in HoFH patients have shown accumulation of liver fat. However, this may vary individually and is increased by alcohol consumption. The long-term effects of this accumulation of fat in the liver resulting from drug intervention could be deleterious and even cause liver cirrhosis. ^399^

Because MTP inhibition leads to reductions not only in hepatic VLDL synthesis but also in intestinal chylomicron production, lomitapide could cause reduced absorption of essential fatty acids and fat-soluble vitamins, especially vitamin E, which is transported primarily by LDL. However, this finding has not been confirmed in FH patients after use of lomitapide. ^402^

Furthermore, research has not shown any significant effect of lomitapide treatment on plasma levels of vitamins A and D. ^399^ However, to prevent nutritional deficiencies, essential fatty acids and fat-soluble vitamins may be supplemented.

Because lomitapide is extensively metabolized by CYP3A4, caution is required for coadministration with CYP3A4 inhibitors (antifungals, diltiazem, verapamil, antibiotics such as ciprofloxacin, clarithromycin, and erythromycin, and protease inhibitors). The combined use of lomitapide 60 mg/day with simvastatin 40 mg/day increased the exposure to simvastatin by 1.7 times compared with simvastatin alone, increasing the risk of adverse effects from simvastatin. ^403^ However, studies of other lipid-lowering drugs showed no significant interactions. Another possible adverse event caused by sudden discontinuation is elevation of LDL-C concentrations because of the rebound effect on VLDL secretion.

Lomitapide is contraindicated during pregnancy, and currently there is no evidence of safety. Undoubtedly, this issue should be addressed before indication for high-risk cases. Finally, the lipid-lowering efficacy of lomitapide was demonstrated in previous studies showing a dose-response effect on LDL-C, in which 10, 25, and 50 mg daily reduced LDL-C by 30, 55, and 70%, respectively. ^392^

### 12.3. Antisense Oligonucleotide

#### 12.3.1. Inhibitors of Apo B synthesis (antisense Apo B inhibition)

Antisense oligonucleotide technology can be used to block the synthesis of a particular protein target. Mipomersen is a second-generation antisense oligonucleotide that binds to the mRNA encoding Apo B-100, thus leading to its degradation by the action of Rnase and reducing the production of Apo B-100. ^404^ Hence, mipomersen inhibits the hepatic synthesis of Apo B-100 and then reduces the plasma concentrations of VLDL, IDL, LDL, and Lp(a). ^405^^-^^407^ A subcutaneous injection of 200 mg is administered once weekly. ^406^

In clinical trials of patients with severe HoFH or HeFH, mipomersen reduced LDL-C by 25 and 28%, respectively. Adverse events include myalgia, fatigue, injection-site reactions, flu-like symptoms, and liver fat deposition (steatosis).

The high cost of this technology and its adverse effects prevented wider use; a possible therapeutic indication is for an extremely severe and rare condition of genetic dyslipidemia, such as HoFH.

Mipomersen was evaluated in a randomized phase 3 trial of HoFH patients. The mean LDL-C reduction was significantly greater in the mipomersen group (-24.7%; 95% CI 31.6 to 17.7%) than in the placebo group (-3.3%; 95% CI 12.1 to 5.5%; p = 0.0003). Injection-site reactions were the most common adverse effects (76% in the mipomersen group vs 24% in the placebo group). ^406^

In 2013, the FDA approved mipomersen for the treatment of HoFH in adults, but data on children were lacking. ^408^ However, marketing was discontinued in 2018.

Phase 3 studies showed that the efficacy of the drug varies considerably, with mean reductions of 25 to 37%, depending on the characteristics of the populations of interest (homozygous or heterozygous forms of FH, severe hypercholesterolemias, or patients at high cardiovascular risk).

### 12.4. Small Interfering RNA (siRNA)

Another method to inhibit the action of PCSK9 is to reduce its production in tissue. Small interfering RNAs (siRNAs) block the translation of messenger RNA. ^409^ Inclisiran is a synthetic siRNA inhibiting hepatic PCSK9 synthesis. The drug was administered SC as first in class in the ORION program, which evaluated its efficacy and safety in reducing LDL-C. Phase 2 and phase 3 studies have shown LDL-C reductions of approximately 50% with an infrequent dose, one administration every six months, in patients with established atherosclerotic cardiovascular disease or at high cardiovascular risk, including FH patients. Ongoing phase 3 studies will provide evidence of long-term safety and efficacy in HoFH patients. Also, the ORION-4 study will evaluate the impact of inclisiran on cardiovascular events. Inclisiran efficacy was demonstrated in a phase 1 study and then confirmed in a phase 2 study, with a 52.6% reduction in LDL-C. ^410^ In the ORION-9 study, a phase 3 study of HeFH patients, inclisiran at a dose of 300 mg reduced LDL-C concentrations by 39.7% on day 510 (vs. 8.2% increase in the placebo group), and between days 90 and 540, there was a 38.1% reduction. The reductions were robust across all genotypes. ^411^ The medication has not yet been approved for use.

### 12.5. Anti-ANGPTL3 Monoclonal Antibody

Another promising therapy, especially for refractory forms of FH and for HoFH, is based on angiopoietin-like 3 peptide (ANGPTL3) inhibition with evinacumab, an anti-ANGPTL3 monoclonal antibody. In HoFH patients, evinacumab reduced LDL-C by 47% when administered intravenously at a dose of 15 mg/kg body weight every 4 weeks. ^412^ Evinacumab was effective in patients with *LDLR* -null mutations (43.4% reduction in LDL-C) and in those with other variants (49.1% reduction in LDL-C).

In severe and refractory forms of hypercholesterolemia, evinacumab was tested subcutaneously and intravenously at different doses and dosing intervals, leading to an LDL-C reduction greater than 50%. ^413^ Evinacumab has been recently approved for HoFH by the FDA.

### 12.6. Bempedoic Acid

Bempedoic acid is a small molecule that reduces LDL-C by inhibiting a key enzyme in the cholesterol biosynthesis pathway, named ATP citrate lyase, which acts upstream of 3-hydroxy-3-methylglutaryl-coenzyme A. Unlike statins, bempedoic acid has a specific action on the liver, not affecting skeletal muscles. ^414^ A randomized phase 3 study enrolled 779 patients with atherosclerotic cardiovascular disease, HeFH, or both, and with treated LDL-C > 70 mg/dL. Bempedoic acid reduced LDL-C by 15.1%, in addition to lowering non-HDL-C, total cholesterol, Apo B, and high-sensitivity C-reactive protein, compared with placebo. ^415^ In a study using a fixed-dose combination of bempedoic acid 180 mg and ezetimibe 10 mg in patients with hypercholesterolemia receiving the maximum tolerated statin dose and at high cardiovascular risk, a 36% reduction in LDL-C was obtained, superior to ezetimibe alone (-23.2%) and bempedoic acid alone (-17.2%). ^416^ The reduction obtained with bempedoic acid plus ezetimibe was similar between the groups receiving high- or moderate-intensity statin and those receiving no statins. Other lipids and high-sensitivity C-reactive protein also showed a greater reduction with the combined therapy.

This drug class is associated with modest elevations in uric acid level, explained by the competition between the drug metabolite and uric acid for the same renal transporters involved in the excretion of the compounds. Data on prolonged exposure safety and cardiovascular outcomes are being evaluated in a phase 3 study whose results are yet to be published. Bempedoic acid and its combination with ezetimibe received approval of the FDA for patients with HeFH and atherosclerotic cardiovascular disease (ASCVD).

## 13. Cost-Effectiveness of Screening and Treatment of Familial Hypercholesterolemia

### 13.1. Introduction

Health resources are currently insufficient to meet all the demands of society. Contemporary medicine is faced with an avalanche of therapies that have been proven to add clinical benefits to incorporated therapies but are usually associated with an incremental cost.

Traditionally, when different medical interventions are evaluated from both clinical and health policy perspectives, the purpose is to determine the efficacy and safety that can be achieved under ideal conditions (randomized controlled trials). Effectiveness will show the real effect of the intervention when used in usual circumstances (real-world studies). Another concept is that of intervention efficiency, which considers not only the effectiveness of each intervention but also the resources needed for implementation.

This panorama has aroused the interest of different segments of the community in the search for solutions. Health economics is a field of interdisciplinary knowledge that can help physicians, managers, and health policy-makers in the difficult task of making decisions in a resource-limited setting.

### 13.2. Cost-of-Illness Study

To analyze the impact of a disease or a treatment in a specific setting (eg, a country, a health care system, a hospital), knowledge of the cost of illness is needed. This type of economic study is based on a descriptive method that, combined with prevalence, incidence, morbidity, and mortality data, helps measure the impact of a specific disease on society.

A cost-of-illness study is not categorized as an economic analysis because it does not compare interventions and does not assess health outcomes. The aim is to estimate the burden or impact of a disease to prioritize the allocation of resources in public health policies, guide research funds, and identify the most expensive diseases for the health budget, in addition to providing data for economic analyses.

### 13.3. Health Economic Analyses

Economic analyses compare different options competing for the allocation of limited resources intended for use in health care. All forms of economic analysis address the use of resources and the health benefits of therapeutic or preventive interventions or even health programs. The analyses compare alternatives and facilitate the process of making decisions for the appropriate use of health resources.

Cost-effectiveness analysis (CEA) is the most widely used type of health economic evaluation and measures the cost in monetary units divided by a nonmonetary unit, named natural unit, such as life years of survival or events prevented after a given health intervention. ^417^

A health intervention is said to be cost-effective if it produces a clinical benefit that justifies the cost. The determination of how much the additional effectiveness justifies the extra cost is based on social values and the availability of resources. WHO recommends using an amount corresponding to 1 to 3 times the gross domestic product (GDP) per capita of the country where the analysis was performed as a justifiable cost-effectiveness threshold for that context. ^418^ In Brazil, an explicit cost-effectiveness threshold was not determined for the Unified Health System (Sistema Único de Saúde, SUS) or the private health insurance system, ie, the amount from which an intervention is considered to be cost-effective was not defined. The definition of this amount is context-specific and depends on local wealth, characteristics of the health system, availability and ability to pay, as well as social preferences. Furthermore, this threshold should always be used together with other value-adding criteria and has been discussed in recent years.

### 13.4. Costs of Familial Hypercholesterolemia

CVDs are the main causes of death and costs for the adult population in Brazil, and this impact will probably grow with increases in life expectancy. FH is an important risk factor for atherosclerotic disease and increases the risk of early events.

Siqueira et al. ^419^ estimated the costs of CVD in Brazil from a SUS perspective over 5 years, including direct costs of hospitalizations, outpatient care visits, and social security benefits, in addition to indirect costs of loss of income caused by CVD mortality. CVD accounted for 28% of all deaths in Brazil and reached 38% of deaths in the working-age population (18 to 65 years old). The estimated CVD costs were R$37.1 billion in 2015, with a 17% increase in the years between 2010 and 2015. The estimated costs of premature death from CVD accounted for 61% of the total CVD cost; direct costs of hospitalizations and consultations were 22%, and costs of illness-related lost productivity were 15%. Estimated health expenditures in Brazil were 9.5% of GDP, and the estimated mean CVD cost was 0.7% of GDP.

Bahia et al. ^420^ estimated the costs of hospitalizations for CAD attributable to FH in the Brazilian adult population from a SUS perspective between 2012 and 2014. With data from the international literature on the prevalence of FH and the relative risk of events, population attributable fractions were calculated and applied to hospitalization costs in all SUS health care units with the SIH-SUS database. In total, 245,981 hospitalizations were recorded over 1 year, and 7,249 (2.9%) to 12,915 (5.2%) were attributable to FH, according to the prevalence used. The total hospitalization cost was R$985,919,299, of which R$29,053,500 to R$51,764,175 were attributable to FH and could be minimized if there was a proper control of this risk factor in the population with adequate treatment.

### 13.5. Cost-Effectiveness of Screening and Treatment of Familial Hypercholesterolemia

Less than 25% of people with FH are diagnosed, according to estimates, and most are incorrectly treated. ^421^ The clinical and economic implications of the low number of diagnoses are significant as an important share of the population with FH which was not treated early will develop atherosclerotic CVD. Thus, an important topic of discussion refers to the cost-effectiveness of population screening for FH for early diagnosis and treatment purposes. Rosso et al. ^422^ conducted a systematic review of economic analyses of genetic screening for FH (cost-effectiveness, cost-utility, cost-benefit, and cost-minimization analyses). Seven economic evaluations were conducted in Europe between 2002 and 2015 focusing on relatives of index patients with a genetic or clinical diagnosis of FH (cascade screening strategy), but this was not compared with a no-screening strategy. Only direct costs were analyzed in studies adopting a health care payer perspective, which means that estimates were conservative as they did not include indirect CVD costs (absenteeism, leaves, retirement, and early deaths). In all contexts, cascade screening was cost-effective, except in a North American study that showed no evidence of economic benefit of genetic screening compared with the currently recommended management of dyslipidemias. ^423^

The effectiveness and cost-effectiveness of statin treatment in FH patients has been clearly demonstrated in different secondary prevention contexts. ^424^^,^^425^ For primary prevention, cost-effectiveness will depend on cardiovascular risk and age of the population, with results supporting a more aggressive treatment in those at higher risk. ^426^ In Brazil, a cost-effectiveness analysis of three statin dosing regimens (low-, intermediate-, and high-intensity statins) was performed from a SUS perspective. Based on government procurement prices, the therapeutic regimen of intermediate-intensity statins (eg, atorvastatin 10 mg and simvastatin 40 mg) was found to be the most cost-effective and was suggested as the most attractive economic approach for SUS in both primary prevention (high CV risk population) and secondary prevention. ^427^

New treatment approaches with PCSK-9 inhibitors require context-specific economic analyses as they are drugs that have shown additional efficacy in the treatment of high-risk patients with residual risk, but treatment costs are remarkably high. Cost-effectiveness of new therapies is important because the costs of health care services are increasing significantly, and accurate data on costs and potential benefits of different therapies are crucial for budget impact analyses.

In the US, three modeling studies demonstrated that PCSK9 inhibitor treatment in patients with established CVD exceeded generally accepted cost-effectiveness thresholds and could significantly increase health care costs. ^428^^-^^430^ The mean annual treatment cost observed in the US models ranged from $14,000 to 15,000, and the authors suggest that cost reduction should be greater than 50% per year for the therapy to be considered cost-effective for reducing cardiovascular events.

### 13.6. Final Considerations

The contribution of cost-of-illness studies and economic analyses consists of supporting physicians and managers in the decision-making process, when they analyze several aspects, including the costs (what, how much, to whom, at what cost) and the benefits, of the action being produced. This will rationally guide decision-making with the purpose of achieving the greatest collective benefit with the available resources.

Choices are frequently made between treatments that have different costs and effectiveness levels but used for the same clinical purpose. Therefore, the best evidence on the treatment of the illness in question should be obtained, and the benefits provided to the patient should be analyzed, with a preference for the intervention with the best cost-effectiveness ratio. In real-world clinical practice, where there is a lack of resources and inequality in health care access, the use of health economic studies can be of great help in decision-making.

## Footnotes

* In the case of advanced subclinical atherosclerotic disease diagnosed with a CAC score greater than 100 AU or 75th percentile for age and sex, or coronary CT angiography showing coronary obstructions greater than 50% or presence of nonobstructive plaques in more than one vessel, LDL-C should be reduced by at least 50%, with < 50 mg/dL being the ideal level.

** Additional risk factors in FH are the following: ^56^ age > 40 years and no treatment, smoking, being male, Lp(a) > 50 mg/dL, HDL-C < 40 mg/dL, hypertension, diabetes mellitus, family history of premature CAD in first-degree relatives (men aged < 55 years and women aged < 60 years), chronic kidney disease (GFR < 60 mL/min), and BMI > 30 kg/m ^2^ .
